# 3-*O*-Carbamoyl-5,7,20-*O*-trimethylsilybins: Synthesis and Preliminary Antiproliferative Evaluation

**DOI:** 10.3390/molecules26216421

**Published:** 2021-10-24

**Authors:** Sitong Wu, Guanglin Chen, Qiang Zhang, Guangdi Wang, Qiao-Hong Chen

**Affiliations:** 1 Department of Chemistry and Biochemistry, California State University, Fresno, CA 93740, USA; sitong@mail.fresnostate.edu (S.W.); chen.bmc@gmail.com (G.C.); 2 Department of Chemistry and RCMI Cancer Research Center, Xavier University of Louisiana, New Orleans, LA 70125, USA; qzhang1@xula.edu (Q.Z.); gwang@xula.edu (G.W.)

**Keywords:** silibinin, carbamoylation, prostate cancer, cell proliferation, androgen receptor

## Abstract

To search for novel androgen receptor (AR) modulators for the potential treatment of castration-resistant prostate cancer (CRPC), naturally occurring silibinin was sought after as a lead compound because it possesses a moderate potency towards AR-positive prostate cancer cells and its chemical scaffold is dissimilar to all currently marketed AR antagonists. On the basis of the structure–activity relationships that we have explored, this study aims to incorporate carbamoyl groups to the alcoholic hydroxyl groups of silibinin to improve its capability in selectively suppressing AR-positive prostate cancer cell proliferation together with water solubility. To this end, a feasible approach was developed to regioselectively introduce a carbamoyl group to the secondary alcoholic hydroxyl group at C-3 without causing the undesired oxidation at C2–C3, providing an avenue for achieving 3-*O*-carbamoyl-5,7,20-*O*-trimethylsilybins. The application of the synthetic method can be extended to the synthesis of 3-*O*-carbamoyl-3′,4′,5,7-*O*-tetramethyltaxifolins. The antiproliferative potency of 5,7,20-*O*-trimethylsilybin and its nine 3-carbamoyl derivatives were assessed in an AR-positive LNCaP prostate cancer cell line and two AR-null prostate cancer cell lines (PC-3 and DU145). Our preliminary bioassay data imply that 5,7,20-*O*-trimethylsilybin and four 3-*O*-carbamoyl-5,7,20-*O*-trimethylsilybins emerge as very promising lead compounds due to the fact that they can selectively suppress AR-positive LNCaP cell proliferation. The IC_50_ values of these five 5,7,20-*O*-trimethylsilybins against the LNCaP cells fall into the range of 0.11–0.83 µM, which exhibit up to 660 times greater in vitro antiproliferative potency than silibinin. Our findings suggest that carbamoylated 5,7,20-*O*-trimethylsilybins could serve as a natural product-based scaffold for new antiandrogens for lethal castration-resistant prostate cancer.

## 1. Introduction

**Figure 1:**
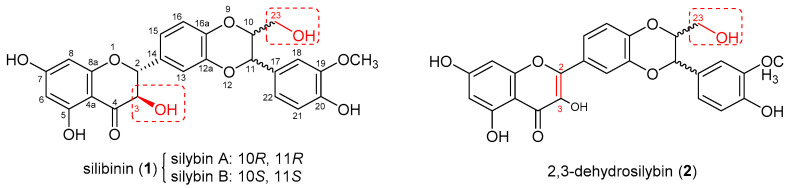
Chemical structures of silibinin (**1**) and 2,3-dehydrosilybin (**2**).

Castration-resistant prostate cancer (CRPC) is a lethal version of prostate cancer that continues to deprive the lives of over 30,000 American men per year [1]. The androgen receptor (AR)-regulated gene expression still holds the foremost impetus for the progression of CRPC [2]. In addition to acquiring drug resistance with the time of treatment, a considerable portion of patients with CRPC are primarily resistant to the current FDA-approved treatments targeting the AR-signaling axis as evidenced by their hazard ratios for the primary end points in the phase III trials [3]. Consequently, novel potential therapeutic strategies for CRPC, including poly(adenosine diphosphate (ADP)-ribose)polymerase inhibitors [4,5], androgen receptor degraders [6,7], and CAR-T cell therapy [8], are emerging. As part of our ongoing project to discover new AR modulators with dissimilar chemical scaffolds for the potential treatment of CRPC, this study picks up naturally occurring silibinin (**1**, also known as silybin, Figure 1) as a lead compound because it has a chemical scaffold completely different from all marketed AR antagonists and possesses moderate potency towards AR-positive prostate cancer cells. Silibinin (**1**) is the most abundant flavonolignan of silymarin, a well-known traditional European medicine and the crude extract from milk thistle (*Silybum marianum*) [9]. Its initial name is silybin because it was considered as a single compound in 1968 [9] and later silibinin was recommended as an alternative name to highlight the fact that it is a diastereomeric mixture of silybin A and silybin B [10]. Throughout this paper, silibinin is used to stand for the commercially available mixture of silybin A and silybin B. In addition to having appreciable potential in treating prostate cancer on the ground of its in vitro and in vivo experimental data [11,12,13], silibinin (**1**) acts as an anti-prostate cancer agent with mechanisms of action that are associated with the AR-signaling axis as summarized in our review article [14]. Specifically, silibinin can inhibit the secretion of prostate-specific antigens (PSA) [11,15,16,17], lower the AR level, prevent AR nuclear localization, and promote AR degradation [11,16,18]. Its non-toxic profiles, which have been corroborated by its long-term dietary application along with its clinical trial data, make it even more alluring as a lead compound [19]. However, its moderate potency, poor selectivity, and poor bioavailability pose significant impediments to clinical applications. Chemical manipulations have been verified by us and others to be a viable strategy to boost its potency [20,21,22,23,24] and to improve its pharmacokinetic profiles [25,26].

The earlier studies on the structure–activity relationships of silibinin (**1**) and 2,3-dehydrosilybin (**2**) from our laboratories revealed that chemical modification on silibinin (**1**) led to significantly higher selectivity in the proliferation inhibition of AR-positive LNCaP cells vs. AR-null PC-3 and DU145 cells when compared with the same chemical modification on 2,3-dehydrosilybin (**2**) [20,21,22]. Our recent investigation suggests that modification of the alcoholic hydroxyl group at C-23 of 3,5,7,20-*O*-tetramethyl-2,3-dehydrosilybin resulted in appreciably higher selectivity in suppressing AR-positive LNCaP prostate cancer cell proliferation compared with modification of the phenolic hydroxyl groups [27]. These results prompted us to aim for the appropriately designed substituents at alcoholic hydroxyl groups at C-3 and C-23 for 5,7,20-*O*-trimethylsilybin (**3**) in hopes to enhance the antiproliferative potency and selectivity towards AR-positive prostate cancer cells. Structure manipulations on the alcoholic hydroxyl groups at C-3 and C-23 have been reported to yield silybin 3,23-bis-*O*-hemisuccinate and 23-phosphodiester silybin with improved pharmacokinetic profiles compared with silibinin [25,26]. However, these derivatives do not show significant improvement in antiproliferative potency against prostate cancer cells (both LNCaP and PC-3 cell lines). The carbamate-incorporated compounds have been proven to possess sufficient water solubility and improved biological activity [28,29]. The carbamate derivatives of 5,7,20-*O*-trimethylsilybin (**3**) have thus been designed to fine-tune the alcoholic hydroxyl groups at C-23 and C-3 in hopes to simultaneously increase the potency, selectivity, and aqueous solubility. In this paper, a regioselective synthesis of 3-*O*-carbamoyl-5,7,20-*O*-trimethylsilybins, along with the antiproliferative potency towards AR-containing and AR-null prostate cancer cell lines, are presented.

## 2. Results and Discussion

### 2.1. Synthesis

**Scheme 1:**
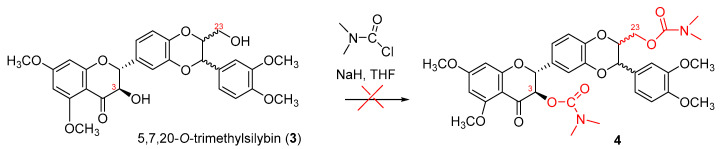
Original attempt to synthesize dicarbamoylsilybin **4.**

So far, no 3-*O*-carbamoyl derivative of flavanonol-based flavonolignans has yet been reported. The synthesis of 3-*O*-carbamoyl derivatives of flavanonol-based flavonolignans could be a challenge due to the fact that an oxidation at the C-2, C-3 position can readily occur under the basic conditions [30] and the typical bases employed for the carbamoylation of the alcoholic hydroxyl groups are NaH or KH [31]. Encouraged by the successful preparation of 7-*O*-benzylsilybin and 5,7,20-*O*-trimethylsilybin mediated by potassium carbonate under strictly anaerobic conditions [21], we initially attempted to synthesize 3,23-*O*-dicarbamoyl-5,7,20-*O*-trimethylsilybin (**4**) by treating 5,7,20-*O*-trimethylsilybin (**3**) [21] with *N*,*N*-dimethylcarbamoyl chloride using NaH as a base under strictly anaerobic conditions (Scheme 1). Unfortunately, the TLC plates from our several trials showed this reaction was very messy and did not yield the desired carbamate **4**, suggesting the starting material **3** (5,7,20-*O*-trimethylsilybin) was decomposed under the strong basic conditions [32].

**Scheme 2:**
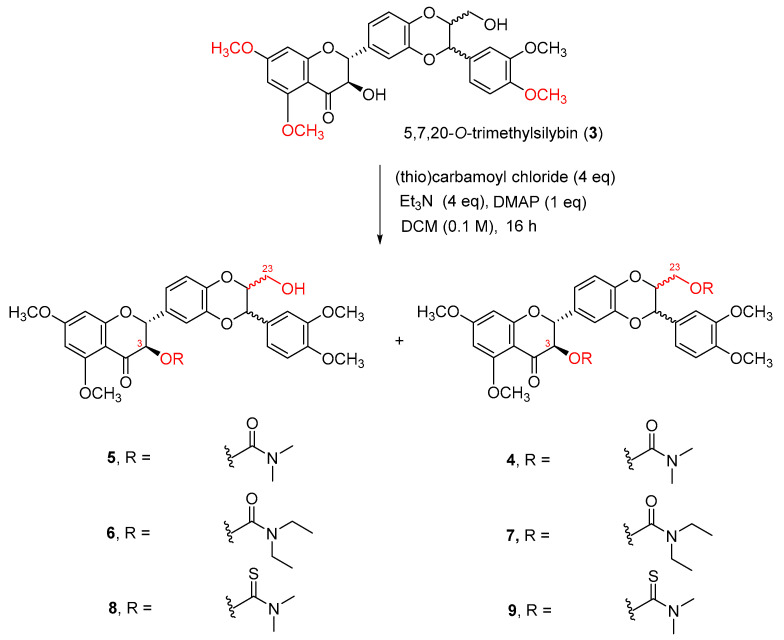
Carbamoylation of 5,7,20-*O*-trimethylsilybin.

At this point, we revisited the literature and searched for weaker organic bases for this carbamoylation. It has been reported that carbamates could be achieved in good yield by refluxing alcohols with *N*,*N*-dimethylcarbamoyl chloride in pyridine [33]. These conditions are not appropriate for the synthesis of 3-*O*-carbamoylsilybin because heating silibinin at 80−90 °C in dry pyridine in the presence of air leads to the oxidation at C2–C3 [34]. A secondary benzylic alcohol was reported to be converted to the corresponding carbamate by treating it with a bulky carbamoyl chloride mediated by Et_3_N in DCM [35,36]. By integrating these two carbamoylation methods, we decided to prepare 3,23-*O*-dicarbamoyl-5,7,20-*O*-trimethylsilybin by treating 5,7,20-*O*-trimethylsilybin with *N*,*N*-dimethylcarbamoyl chloride (4 eq) in DCM (0.1 M) using triethylamine (4 eq) and 4-(*N*,*N*-dimethylamino)pyridine (DMAP,1 eq) at room temperature for 16 h (Scheme 2). Surprisingly, the carbamoylation regioselectively occurs at the secondary alcohol at C-3 in the presence of the primary alcohol at C-23 when treating 5,7,20-*O*-trimethylsilybin (**3**) with *N*,*N*-dimethylcarbamoyl chloride using triethylamine and DMAP as bases. This unexpected regioselective reaction has been repeated over forty times by two co-authors, and the structure of the 3-*O*-carbamoyl-5,7,20-*O*-trimethylsilybin (**5**) has been confirmed by 2D-NMR data and HRMS (see Structure Determination). Removal of DMAP resulted in no carbamoylation reaction, and the minimum amount of DMAP required for the completion of *N*,*N*-dimethylcarbamoylation of 5,7,20-*O*-trimethylsilybin (**3**) was 0.5 equivalents, suggesting an appropriate amount of DMAP is crucial for facilitating the carbamoylation.

In addition to obtaining the major 3-*O*-carbamoyl-5,7,20-*O*-trimethylsilybin (**5**) in 79% yield, a small amount of 5,7,20-*O*-trimethyl-3,23-*O*-di-(*N*,*N*-dimethylcarbamoyl)silybin (**4**) was also isolated in 13% yield (Table 1, entry 1). Adding more triethyl amine and DMAP, prolonging reaction time, or increasing reaction temperature can slightly increase the yields for 3,23-*O*-dicarbamoylsilbybin (**4**), but cannot promote the completion of carbamoylation at the primary alcoholic hydroxyl group at C-23 (Table 1, entries 4 and 7). This regioselective carbamoylation at the secondary alcoholic hydroxyl group at C-3 can be extended to other carbamoyl chlorides. A similar tendency was observed when reacting 5,7,20-*O*-trimethylsilybin (**3**) with *N*,*N*-diethylcarbamoyl chloride (Table 1, entries 5 and 8) or *N*,*N*-dimethylthiocarbamoyl chloride (Table 1, entries 6 and 9). It is worth noting that the bulky carbamoyl chlorides (i.e., *N*,*N*-diethylcarbamoyl chloride and *N*,*N*-dimethylthiocarbamoyl chloride) significantly decrease the conversion rates when compared with *N*,*N*-dimethylcarbamoyl chloride under the same set of carbamoylation conditions (Table 1).

### 2.2. Structure Determination of 3-O-Carbamoyl-5,7,20-O-trimethylsilybin ***5***

**Figure 2:**
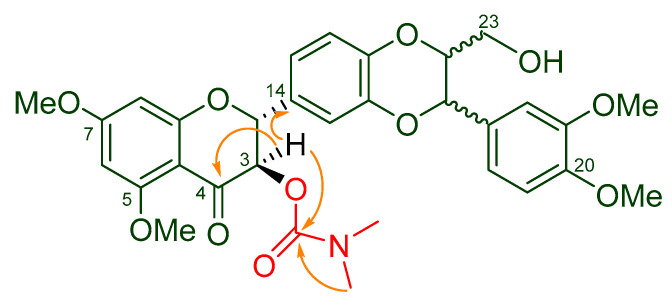
Key HMBC correlations (from H to C) in **5**.

The structure of 5,7,20-*O*-trimethyl-3-*O*-(*N*,*N*-dimethylcarbamoyl)-silybin (**5**) was elucidated by interpreting its 1D- and 2D-NMR data (Table 2), as well as high resolution MS and IR data. The structure of **5** was characterized by the existence of one signal at 2.85 ppm representing 6 protons in its ^1^H NMR spectrum (Supplementary Materials) and at 36.75 (36.08) and 155.28 in its ^13^C NMR spectrum for an additional dimethylcarbamoyl group when compared with the starting material 5,7,20-*O*-trimethylsilybin (**3**), which was corroborated by the HRMS data. The dimethylcarbamoyl group in **5** was assigned to 3-OH based on the fact that the H-3 signal is downshifted from 4.42 ppm to 5.51 (5.49) ppm relative to 5,7,20-*O*-trimethylsilybin (**3**) [21]. The carbamoylation at 3-OH was further confirmed by the critical HMBC correlations from the signal at *δ*_H_ 5.51 (5.49) (H-3) to the signal at *δ*_C_ 155.28 (carbonyl carbon from the dimethylcarbamoyl group, Figure 2). It is worth noting that the starting material silibinin (**1**) (purchased from Fisher Scientific) was a nearly equimolar diastereoisomeric mixture of silybin A and silybin B. The carbamoyled derivatives of 5,7,20-*O*-trimethylsilybin are thus diastereoisomeric mixtures as well, and some NMR signals of these derivatives therefore appear as a pair (see Table 2 and Materials and Methods).

### 2.3. Scope of the Reaction Approach

**Scheme 3:**
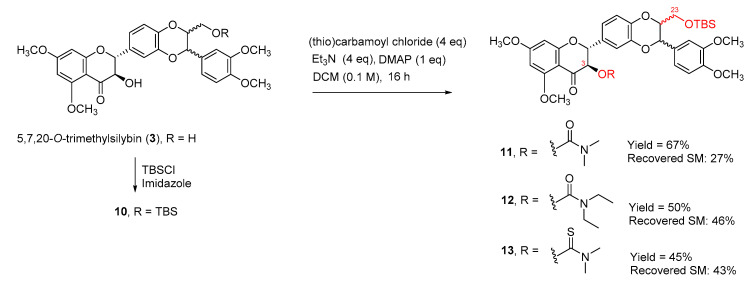
Synthesis of 3-*O*-carbamoyl-23-*O*-TBS-5,7,20-trimethylsilybins.

**Scheme 4:**
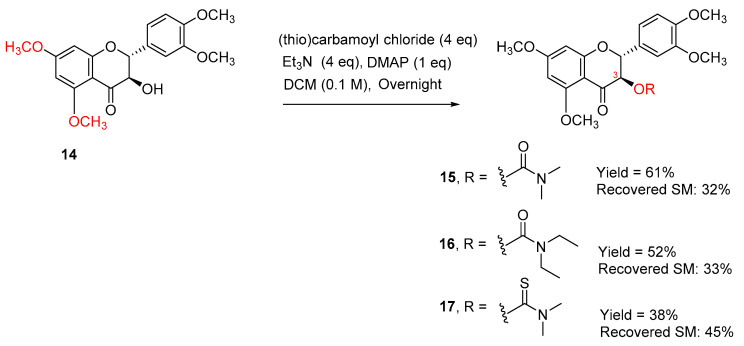
Synthesis of 3-*O*-carbamoyltaxifolins.

The selectivity may be caused by the hydrogen bonding between the hydrogen of the alpha-OH and the 4-carbonyl oxygen atom. To further confirm that the secondary 3-OH in 5,7,20-*O*-trimethylsilybin can be carbamoyled by using triethylamine and DMAP as bases, 5,7,20-*O*-trimethyl-23-*O*-TBS-silybin (**10**) was exposed to the same set of reaction conditions, resulting in three 3-*O*-carbamoyl-5,7,20-*O*-trimethyl-23-*O*-TBS-silybins (**11**, **12**, and **13**) in 45–67% yields together with the recovered starting material **10** (27–46%), as shown in Scheme 3. To further explore the applicability of the new carbamoylation method to flavanonols, the application of the same set of reaction conditions to 3′,4′,5,7-*O*-tetramethyltaxifolin (**14**) led to the formation of three desired taxifolin derivatives, **15**, **16**, and **17**, in 38–61% yields along with the recovered starting material **14** (32–45%) (Scheme 4). This implies that the carbamoylation provides a feasible approach to not only 3-*O*-carbamoylated flavonolignans but also 3-*O*-carbamoylated flavanonols.

**Scheme 5:**
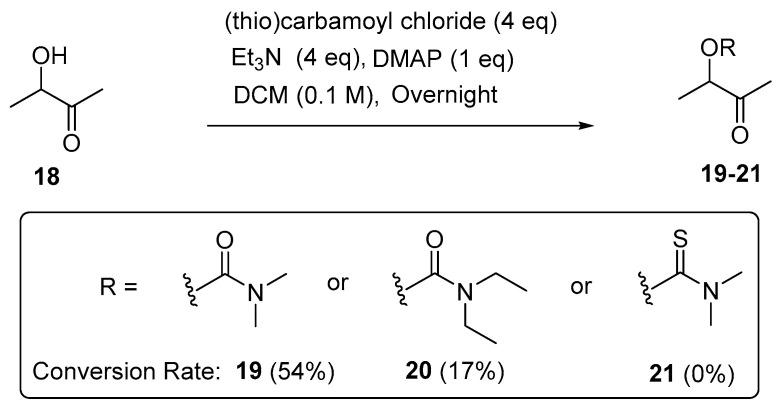
Carbamoylation of open-chain α-hydroxyl ketones.

**Figure 3:**
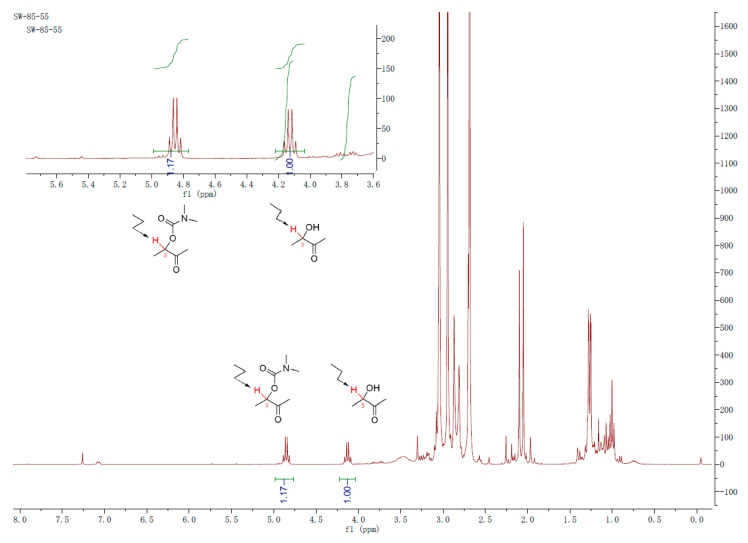
^1^H NMR spectrum for the crude product from reaction of *α*-hydroxyl ketones with *N*,*N*-dimethylcarbamoyl chloride.

The applicability of the method was also evaluated in the open-chain α-hydroxyl ketones. Acyclic 3-hydroxy-2-butanone (**18**) was selected and treated with the respective (thio)carbamoyl chloride (4 eq) in the presence of triethylamine (4 eq) and DMPA (1 eq) in DCM (0.1 M). The ^1^H NMR analysis (Figure 3) of the characteristic H-3 in the crude product indicated that 54% and 17% of 3-hydroxy-2-butanone (**18**) can be converted to the corresponding *N*,*N*-dimethylcarbamoyl product (**19**) and *N*,*N*-diethylcarbamoyl product (**20**), respectively (Scheme 5). The overall conversion rates for the open-chain α-hydroxyl ketones are lower than those for the cyclic α-hydroxyl ketones, and no carbamoylation reaction of 3-hydroxy-2-butanone with *N*,*N*-dimethylthiocarbamoyl halide under the above-mentioned conditions was observed.

### 2.4. Preliminary Anti-Proliferative Activity towards AR-Positive and AR-Null Prostate Cancer Cell Lines

The preliminary in vitro antiproliferative potency of 5,7,20-*O*-trimethylsilybin (**3**) and its nine carbamoyled derivatives towards the AR-positive LNCaP prostate cancer cell line was assessed using the WST-1 cell proliferation assay according to the procedure described in the Materials and Methods section. The AR-null DU145 and PC-3 prostate cancer cell models were used to evaluate the inhibitory selectivity towards AR-positive cells over AR-null ones. Enzalutamide, a current FDA-approved second-generation AR antagonist for CRPC, and silibinin were used as positive controls for comparison. The IC_50_ values were calculated based on the dose-response curves and summarized in Table 3. The IC_50_ values for 5,7,20-*O*-trimethylsilybin (**3**) and four derivatives (**4**, **5**, **6**, and **8**) against the LNCaP cells are 0.11–0.83 µM, which exhibit up to 660 times greater in vitro antiproliferative potency than silibinin. All these 5,7,20-*O*-trimethylsilybins are also significantly more potent than enzalutamide in the AR-positive LNCaP cell model. These five 5,7,20-*O*-trimethylsilybins are clearly more potent in suppressing cell proliferation of AR-positive LNCaP prostate cancer cells than AR-negative PC-3 and DU145 cells by comparing their IC_50_ values.

On the grounds of our preliminary bioassay data, 5,7,20-*O*-trimethylsilybin (**3**) and its four 3-substituted derivatives (**4**, **5**, **6**, and **8**) emerge as very promising lead compounds due to the fact that they can selectively suppress AR-positive LNCaP cell proliferation with IC_50_ values of 0.11−0.83 µM and have more efficacy than the current FDA-approved second-generation AR antagonist enzalutamide (Table 3). Our findings suggest that 3-*O*-substituted-5,7,20-*O*-trimethylsilybin may serve as a natural product-based scaffold for new antiandrogens for lethal castration-resistant prostate cancer.

## 3. Materials and Methods

### 3.1. General Procedures

IR spectra were recorded on a Nicolet Nexus 470 FTIR spectrophotometer (Waltham, MA, USA). HRMS were obtained on an Orbitrap mass spectrometer with electrospray ionization (ESI). NMR spectra were obtained on a Bruker Fourier 300 spectrometer (Billerica, MA, USA) in CDCl_3_. The chemical shifts are given in ppm referenced to the respective solvent peak, and coupling constants are reported in Hz. The value of the central peak of the solvent was calibrated as *δ* = 7.26 ppm for the ^1^H NMR spectrum and as *δ* = 77.16 ppm for the ^13^C NMR spectrum, respectively. THF and dichloromethane were purified by the PureSolv MD 7 Solvent Purification System from Innovative Technologies (MB-SPS-800) (Herndon, VA, USA). All other reagents and solvents were purchased from commercial sources and were used without further purification. Silica gel column chromatography was performed using silica gel (32–63 µm). Preparative thin-layer chromatography (PTLC) separations were carried out on thin-layer chromatography plates loaded with silica gel 60 GF254 (EMD Millipore Corporation) (Berlington, MA, USA). 5,7,20-*O*-Trimethylsilybin (**3**) was synthesized from silibinin (>98%, purchased from Fisher Scientific (Portland, OR, USA)) using the procedure previously described by us [21]. The HPLC purity analyses were performed on an Agilent Hewlett-Packard 1100 series HPLC DAD system (Santa Clara, CA, USA) using a 5 µM C18 reversed phase column (4.6 × 250 mm) and a diode array detector. Solvent A was methanol and solvent B was 5% methanol in DI water. All testing samples were run for 30 min of 35−100% A in B, with a 20 min gradient. The flow rate was 1 mL/min.

### 3.2. General Procedure for the Synthesis of Carbamoyled Derivatives of 5,7,20-Trimethylsilybin

Triethylamine (56 µL, 0.4 mmoL) and 4-(*N*,*N*-dimethylamino)pyridine (12 mg, 0.1 mmol) were sequentially added to a solution of 5,7,20-*O*-trimethylsilybin (**3**, 52.5 mg, 0.1 mmol) in DCM (1 mL, 0.1 M). The subsequent mixture was stirred for 10 min at room temperature, to which the respective (thio)carbamoyl chloride (0.4 mmol) was added. The reaction was allowed to proceed at room temperature with stirring overnight under argon prior to being quenched with brine (50 mL). The resulting mixture was extracted with ethyl acetate (30 mL × 3), the combined organic extracts were dried over anhydrous sodium sulfate, and the organic solvents were removed. The crude product was subjected to PTLC purification eluting with DCM:MeOH (95:5, *v*/*v*) to afford the respective 3-carbomoyled derivative and 3,23-dicarbomoyled derivative. Their physical and spectral data are summarized below.

#### 3.2.1. 5,7,20-*O*-Trimethyl-3-*O*-(*N*,*N*-dimethylcarbamoyl)silybin (**5**)

Yield, 79%; white solid; m.p. 113−115 °C. ^1^H NMR (300 MHz, CDCl_3_) and ^13^C NMR (75 MHz, CDCl_3_): see Table 2. HRMS (ESI): *m*/*z* calculated for C_31_H_34_NO_11_ [M+H]^+^: 596.2132. Found: 596.2128. IR (film) *ν*_max_: 3361, 2943, 2833, 1689, 1610, 1572, 1509 cm^−1^. HPLC purity 100% (two very close signals were observed for the diastereomers).

#### 3.2.2. 5,7,20-*O*-Trimethyl-3,23-*O*-di(*N*,*N*-dimethylcarbamoyl)silybin (**4**)

Yield, 13%; colorless syrup. ^1^H NMR (300 MHz, CDCl_3_) *δ* 7.15 (d, *J* = 1.2 Hz, 1H, H-13), 6.09–7.01 (overlapped, 2H, H-15 and H-16), 6.94 (dt, *J* = 8.4, 1.5 Hz, 1H, H-22), 6.90–6.87 (overlapped, 2H, H-18 and H-22), 6.11 (d, *J* = 2.1 Hz, 1H, H-6), 6.09 (d, *J* = 2.4 Hz, 1H, H-8), 5.53 (5.51) (d, *J* = 11.7 Hz, 1H, H-3), 5.31 (5.30) (d, *J* = 11.7 Hz, 1H, H-2), 4.91 (4.90) (d, *J* = 7.8 Hz, 1H, H-11), 4.34 (dd, *J* = 11.9, 3.0 Hz, 1H, H-23), 4.27 (ddd, *J* = 7.8, 4.2, 3.0 Hz, 1H, H-10), 3.97 (dd, *J* = 11.9, 4.2 Hz, 1H, H-23), 3.91 (s, 3H, OCH_3_), 3.90 (s, 3H, OCH_3_), 3.86 (s, 3H, OCH_3_), 3.81 (s, 3H, OCH_3_), 2.88 (s, 12H, 2 × *N(*CH_3_)_2_). ^13^C NMR (75 MHz, CDCl_3_) *δ* 186.41 (C-4), 166.48, 164.24, 162.48, 155.90, 155.34, 149.87, 149.57 (149.51), 143.98, 143.86 (143.83), 129.63 (129.52), 128.34, 121.05, 120.22, 117.31 (117.21), 116.50 (116.39), 111.43 (111.35), 110.18 (110.02), 104.38 (104.34), 93.48, 81.03 (80.94), 76.87, 76.12, 74.75 (74.62), 63.94, 56.19 (56.13), 56.10, 56.05 (56.00), 55.77 (55.72), 36.79, 36.14. HRMS (ESI): *m*/*z* calculated for C_34_H_39_N_2_O_12_ [M+H]^+^: 667.2503. Found: 667.2498. IR (film) *ν*_max_: 2935, 2839, 1701, 1608, 1573, 1539, 1508 cm^−1^. HPLC purity 96.9% (only one signal was observed for the diastereomers).

#### 3.2.3. 3-*O*-(*N*,*N*-Diethylcarbamoyl)-5,7,20-*O*-trimethylsilybin (**6**)

Yield, 34%; slight yellow solid; 114–116 °C. ^1^H NMR (300 MHz, CDCl_3_) *δ* 7.16 (7.13) (d, *J* = 1.5 Hz, 1H, H-13), 7.03–6.92 (overlapped, 4H, H-15, H-16, H-18, H-22), 6.89 (d, *J* = 8.1 Hz, 1H, H-21), 6.11 (d, *J* = 2.1 Hz, 1H, H-6), 6.08 (d, *J* = 2.1 Hz, 1H, H-8), 5.54 (d, *J* = 11.7 Hz, 1H, H-3), 5.30 (5.28) (d, *J* = 11.7 Hz, 1H, H-2), 4.95 (4.94) (d, *J* = 8.1 Hz, 1H, H-11), 4.02 (ddd, *J* = 8.1, 5.4, 3.6 Hz, 1H, H-10), 3.91 (s, 3H, OCH_3_), 3.89 (s, 3H, OCH_3_), 3.85 (s, 3H, OCH_3_), 3.79 (3.78) (s, 3H, OCH_3_), 3.82−3.77 (overlapped, 1H, H-23), 3.53 (dd, *J* = 12.3, 3.6 Hz, 1H, H-23), 3.33–3.10 (m, 4H, 2 × CH_2_CH_3_), 1.11–0.97 (m, 6H, 2 × CH_2_CH_3_). ^13^C NMR (75 MHz, CDCl_3_) *δ* 186.47, 166.38, 164.18, 162.41, 154.51 (154.44), 149.72, 149.49 (149.43), 143.99 (143.92), 129.66 (129.58), 128.61 (128.58), 120.92 (120.89), 120.22 (120.18), 117.12 (117.00), 116.55, 111.33 (111.27), 110.19, 110.12 (110.07), 104.42 (104.38), 93.52 (93.47), 93.44 (93.39), 81.13 (81.05), 78.37, 76.34 (76.24), 74.31, 61.70, 56.21 (56.15), 56.07, 56.01 (55.96), 55.75 (55.69), 42.40 (41.55), 14.00 (13.44). HRMS (ESI): *m*/*z* calculated for C_33_H_38_NO_11_ [M+H]^+^: 624.2445. Found: 624.2443. IR (film) *ν*_max_: 3468, 2964, 2933, 2838, 1686, 1607, 1571, 1508 cm^−1^. HPLC purity 97.8% (two very close signals were observed for the diastereomers).

#### 3.2.4. 3,23-*O*-Di(*N*,*N*-diethylcarbamoyl)-5,7,20-*O*-trimethylsilybin (**7**)

Yield, 9%; colorless wax. ^1^H NMR (300 MHz, CDCl_3_) *δ* 7.17 (7.13) (d, *J* = 1.8 Hz, 1H, H-13), 7.01–6.87 (overlapped, 5H, H-15, H-16, H-18, H-21, H-22), 6.11 (d, *J* = 2.1 Hz, 1H, aromatic H-6), 6.09 (d, *J* = 2.1 Hz, 1H, H-8), 5.55 (d, *J* = 11.7 Hz, 1H, H-3), 5.30 (5.29) (d, *J* = 11.7 Hz, 1H, H-2), 4.89 (4.87) (d, *J* = 7.2 Hz, 1H, H-11), 4.32 (dd, *J* = 12.0, 5.1 Hz, 1H, H-23), 4.25 (ddd, *J* = 7.2, 5.1, 4.5 Hz, 1H, H-10), 3.98 (dd, *J* = 12.0, 4.5 Hz, 1H, H-23), 3.90 (3.88) (s, 3H, OCH_3_), 3.89 (s, 3H, OCH_3_), 3.86 (s, 3H, OCH_3_), 3.80 (3.79) (s, 3H, OCH_3_), 3.34–3.12 (m, 8H, 4 × CH_2_CH_3_), 1.18–0.96 (m, 12H, 4 × CH_2_CH_3_). ^13^C NMR (75 MHz, CDCl_3_) *δ* 186.43, 166.35, 164.17, 162.42, 155.30, 154.50 (154.45), 149.83, 149.54 (149.47), 143.96 (143.93), 143.80 (143.77), 129.58 (129.51), 128.32 (128.30), 121.14 (120.97), 120.16, 117.25 (117.12), 116.57 (116.43), 111.37 (111.29), 110.08 (109.93), 104.43 (104.39), 93.52 (93.43), 93.43 (93.35), 81.15 (81.04), 76.15, 74.33, 69.59, 63.46, 56.29 (56.14), 56.08 (56.03), 55.94 (55.82), 55.69 (55.63), 42.05, 41.48, 14.18, 13.54. HRMS (ESI): *m*/*z* calculated for C_38_H_47_N_2_O_12_ [M+H]^+^: 723.3129. Found: 723.3129. IR (film) ν_max_: 2971, 2933, 1698, 1609, 1574, 1509 cm^−1^. HPLC purity 100% (only one signal was observed for the diastereomers).

#### 3.2.5. 5,7,20-*O*-Trimethyl-3-*O*-(*N*,*N*-dimethylthiocarbamoyl)silybin (**8**)

Yield, 25%; slight yellow solid; 124–125 °C. ^1^H NMR (300 MHz, CDCl_3_) *δ* 7.17 (7.16) (d, *J* = 1.8 Hz, 1H, H-13), 7.08 (7.07) (dd, *J* = 8.4, 1.8 Hz, 1H, H-15), 7.02–6.98 (overlapped, 2H, H-16 and H-22), 6.94 (6.93) (d, *J* = 2.1 Hz, 1H, H-18), 6.90 (d, *J* = 8.1 Hz, 1H, H-21), 6.61 (6.57) (d, *J* = 11.6 Hz, 1H, H-3), 6.12 (6.11) (d, *J* = 2.3 Hz, 1H, H-6), 6.09 (d, *J* = 2.3 Hz, 1H, H-8), 5.37 (5.36) (d, *J* = 11.6 Hz, 1H, H-2), 4.97 (d, *J* = 8.4 Hz, 1H, H-11), 4.05 (ddd, *J* = 8.4, 3.9, 3.0 Hz, 1H, H-10), 3.91 (s, 3H, OCH_3_), 3.90 (s, 3H, OCH_3_), 3.86 (s, 3H, OCH_3_), 3.81 (s, 3H, OCH_3_), 3.80 (dd, *J* = 12.6, 3.0 Hz, 1H, H-23), 3.54 (dd, *J* = 12.6, 3.9 Hz, 1H, H-23), 3.33 (s, 3H, *N*CH_3_), 3.10 (3.09) (s, 3H, *N*CH_3_). ^13^C NMR (75 MHz, CDCl_3_) *δ* 187.46, 187.42, 166.49, 163.95, 162.40, 149.76, 149.50 (149.46), 144.17 (144.13), 144.00 (143.90), 128.99 (128.92), 128.55 (128.52), 121.26 (121.04), 120.25, 117.24, 116.96, 111.35, 110.23, 104.36, 93.56 (93.50), 93.46 (93.39), 81.06, 79.31 (79.06), 78.45 (78.30), 76.30, 61.73, 56.14, 55.99, 55.83, 55.70, 43.39, 38.33. HRMS (ESI): *m*/*z* calculated for C_31_H_34_NO_10_S [M+H]^+^: 612.1904. Found: 612.1920. IR (film) *ν*_max_: 3481, 2929, 2838, 1684, 1653, 1607, 1571, 1539, 1507 cm^−1^. HPLC purity 100% (only one signal was observed for the diastereomers).

#### 3.2.6. 5,7,20-*O*-Trimethyl-3,23-*O*-(*N*,*N*-dimethylthiocarbamoyl)silybin (**9**)

Yield, 7%; colorless wax. ^1^H NMR (300 MHz, CDCl_3_) *δ* 7.17 (7.16) (d, *J* = 1.8 Hz, 1H, H-13), 7.07 (d, *J* = 8.1 Hz, 1H, H-16), 6.99 (dd, *J* = 8.4, 2.1 Hz, 1H, H-22), 6.93 (dd, *J* = 8.1, 1.8 Hz, 1H, H-15), 6.90–6.87 (overlapped, 2H, H-18 and H-21), 6.60 (6.56) (d, *J* = 11.6 Hz, 1H, H-3), 6.12 (d, *J* = 2.7 Hz, 1H, H-8), 6.09 (d, *J* = 2.7 Hz, 1H, H-6), 5.37 (d, *J* = 11.6 Hz, 1H, H-2), 4.92 (4.90) (d, *J* = 7.8 Hz, 1H, H-11), 4.67 (dd, *J* = 11.4, 2.4 Hz, 1H, H-23), 4.40–4.30 (overlapped, 2H, H-10, H-23), 3.89 (s, 6H, 2 × OCH_3_), 3.86 (s, 3H, OCH_3_), 3.81 (s, 3H, OCH_3_), 3.34 (s, 6H, 2 × *N*CH_3_), 3.11 (3.10, 3.08) (s, 6H, 2 × *N*CH_3_). ^13^C NMR (75 MHz, CDCl_3_) *δ* 187.69, 187.60, 187.49, 166.56, 164.06, 162.48, 150.00, 149.64 (149.61), 144.01 (143.98), 143.85 (143.77), 129.10 (129.01), 128.15, 121.34 (121.25), 120.30 (120.25), 117.39, 116.88, 111.46, 110.14 (110.03), 104.40, 93.51, 93.49, 81.09, 79.06, 76.87, 75.84, 69.60, 56.22, 56.15, 56.08, 55.81 (55.75), 43.36, 43.01, 38.30, 38.00. HRMS (ESI): *m*/*z* calculated for C_34_H_39_N_2_O_10_S_2_ [M+H]^+^: 699.2046. Found: 699.2050. IR (film) *ν*_max_: 2937, 1688, 1606, 1672, 1508 cm^−1^. HPLC purity 97.9% (only one signal was observed for the diastereomers).

### 3.3. Synthesis of 23-O-TBS-5,7,20-O-Trimethylsilybin (***10***)

Imidazole (36 mg, 0.53 mmol) and TBSCl (57 mg, 0.38 mmol) were added to a solution of 5,7,20-*O*-trimethylsilybin (**3**, 200 mg, 0.31 mmol, 1.0 eq) in DCM (3.1 mL). The reaction was stirred at room temperature for 2 h prior to being quenched with saturated aqueous solution of ammonium chloride (20 mL). The subsequent mixture was extracted with ethyl acetate (10 mL × 3). The combined organic layers were dried over anhydrous sodium sulfate. Two PTLC plates were used to purify the crude product eluting with DCM:MeOH (97:3, *v*/*v*) to yield **10** as a white foam-like solid in 76% yield; 87−89 °C. ^1^H NMR (300 MHz, CDCl_3_) *δ* 7.22 (7.18) (d, *J* = 1.8 Hz, 1H, H-13), 7.11–6.97 (overlapped, 4H, H-15, H-16, H-18, H-22), 6.89 (d, *J* = 8.1 Hz, 1H, H-21), 6.11 (d, *J* = 2.1 Hz, 1H, H-8), 6.10 (d, *J* = 2.1 Hz, 1H, H-6), 5.02 (d, *J* = 8.1 Hz, 1H, H-11), 4.94 (d, *J* = 12.2 Hz, 1H, H-2), 4.45 (4.43) (d, *J* = 12.2 Hz, 1H, H-3), 3.96 (ddd, *J* = 8.1, 4.2, 2.4 Hz, 1H, H-10), 3.90 (s, 3H, OCH_3_), 3.89 (s, 3H, OCH_3_), 3.88 (s, 3H, OCH_3_), 3.89–3.81 (overlapped, 1H, H-23), 3.80 (3.79) (s, 3H, OCH_3_), 3.55 (dd, *J* = 11.7, 2.7 Hz, 1H, H-23), 0.90 (s, 9H, TBS), 0.07 (0.06) (s, 6H, TBS). ^13^C NMR (75 MHz, CDCl_3_) *δ* 190.97, 167.12, 165.06, 162.24, 149.46, 149.19, 144.76 (144.69), 144.05 (143.97), 129.40 (129.35), 129.21, 121.32 (120.86), 120.19, 117.37 (117.21), 116.67 (116.33), 111.18, 110.45, 103.01, 93.79, 93.43, 83.12, 78.67, 76.17, 72.73 (72.64), 62.40, 56.42, 56.07, 55.92, 55.77, 25.94, 18.41, −4.95, −5.34. HRMS (ESI): *m*/*z* calculated for C_24_H_43_O_10_Si [M+H]^+^: 639.2626. Found: 639.2623. IR (film) *ν*_max_: 3446, 2951, 2929, 2882, 2854, 1716, 1675, 1606, 1573, 1540, 1507 cm^−1^.

### 3.4. General Procedure for the Synthesis of Carbamoyled Derivatives of 23-O-TBS-5,7,20-O-Trimethylsilybin

Triethylamine (56 µL, 0.4 mmol) and 4-dimethylaminopyridine (12 mg, 0.1 mmol) were sequentially added to a solution of 23-*O*-TBS-5,7,20-*O*-trimethylsilybin (**10**) (63.8 mg, 0.1 mmol) in DCM (1 mL, 0.1 M). The subsequent mixture was stirred for 10 min at room temperature, to which the respective (thio)carbamoyl chloride (0.4 mmol) was added. The reaction was allowed to proceed at room temperature overnight under argon prior to being quenched with brine (50 mL). The resulting mixture was extracted with ethyl acetate (30 mL × 3), the combined organic extracts were dried over anhydrous sodium sulfate, and the organic solvents were removed. The crude product was subjected to PTLC purification eluting with DCM/MeOH (95:5, *v*/*v*) to afford the respective 3-carbomoyled derivative. Their physical and spectral data are summarized below.

#### 3.4.1. 23-*O*-TBS-5,7,20-*O*-Trimethyl-3-*O*-(*N*,*N*-dimethylcarbamoyl)silybin (**11**)

Yield, 67%; white solid; 81−82 °C. ^1^H NMR (300 MHz, CDCl_3_) *δ* 7.15 (7.11) (d, *J* = 1.5 Hz, 1H, H-13), 7.01–6.93 (overlapped, 4H, H-15, H-16, H18, H-22), 6.87 (d, *J* = 8.1 Hz, 1H, H-21), 6.10 (6.09) (d, *J* = 2.3 Hz, 1H, H-8), 6.08 (d, *J* = 2.3 Hz, 1H, H-6), 5.53 (5.51) (d, *J* = 11.7 Hz, 1H, H-3), 5.30 (5.29) (d, *J* = 11.7 Hz, 1H, H-2), 5.00 (4.99) (d, *J* = 8.1 Hz, 1H, H-11), 3.97 (ddd, *J* = 8.1, 3.0, 2.3 Hz, 1H, H-10), 3.91 (s, 3H, OCH_3_), 3.91 (3.87) (s, 3H, OCH_3_), 3.84 (s, 3H, OCH_3_), 3.79 (3.78) (s, 3H, OCH_3_), 3.85 (dd, *J* = 11.7, 2.3 Hz, 1H, H-23), 3.55 (dd, *J* = 11.7, 3.0 Hz, 1H, H-23), 2.87 (s, 6H, 2 × *N*CH_3_), 0.89 (s, 9H, TBS), 0.06 (s, 3H, TBS), 0.057 (0.070) (s, 3H, TBS). ^13^C NMR (75 MHz, CDCl_3_) *δ* 186.44, 166.44, 164.20, 162.41, 155.30, 149.67, 149.44 (149.38), 149.15 (149.10), 144.54, 144.04 (144.00), 143.96 (143.90), 129.60 (129.47), 129.09 (129.05), 129.01 (128.89), 128.55 (128.52), 121.00 (120.80), 120.20, 117.13 (117.03), 116.55 (116.46), 111.25 (111.19), 110.15 (110.00), 104.30, 93.52 (93.42), 93.42 (93.35), 80.99 (80.90), 78.46 (78.28), 76.26, 74.69 (74.54), 61.68, 56.19 (56.10), 56.04, 55.95, 55.79 (55.66), 36.79, 36.12, 25.95 (25.89), 25.76 (25.70), 18.38 (18.05), −3.47 (−3.56), −5.30 (−5.39). HRMS (ESI): *m*/*z* calculated for C_37_H_48_NO_11_Si [M+H]^+^: 710.2997. Found: 710.3006. IR (film) *ν*_max_: 2928, 2855, 1713, 1693, 1607, 1573, 1508 cm^−1^. HPLC purity 94.2% (only one signal was observed for the diastereomers).

#### 3.4.2. 23-*O*-TBS-3-*O*-(*N*,*N*-diethylcarbamoyl)-5,7,20-*O*-trimethylsilybin (**12**)

Yield, 50%; white solid; 79−81 °C. ^1^H NMR (300 MHz, CDCl_3_) *δ* 7.15 (7.11) (d, *J* = 1.8 Hz, 1H, H-13), 7.02–6.93 (overlapped, 4H, H-15, H-16, H-18, H-22), 6.89 (d, *J* = 8.1 Hz, 1H, H-21), 6.11 (6.10 (d, *J* = 2.3 Hz, 1H, H-8), 6.09 (6.08) (d, *J* = 2.3 Hz, 1H, H-6), 5.57 (5.55) (d, *J* = 11.9 Hz, 1H, H-3), 5.29 (5.28) (d, *J* = 11.9 Hz, 1H, H-2), 5.00 (4.98) (d, *J* = 7.8 Hz, 1H, H-11), 3.97 (ddd, *J* = 7.8, 3.0, 2.1 Hz, 1H, H-10), 3.89 (s, 3H, OCH_3_), 3.89 (3.88) (s, 3H, OCH_3_), 3.85 (s, 3H, OCH_3_), 3.91−3.82 (overlapped, 1H, H-23), 3.79 (3.78) (s, 3H, OCH_3_), 3.55 (dd, *J* = 11.7, 3.0 Hz, 1H, H-23), 3.30–3.09 (m, 4H, 2 × CH_2_CH_3_), 1.04 (br.s, 6H, 2 × CH_2_CH_3_), 0.89 (s, 9H, TBS), 0.06 (s, 6H, TBS). ^13^C NMR (75 MHz, CDCl_3_) *δ* 186.53, 166.30, 164.20, 162.37, 154.46 (154.40), 149.40, 149.15 (149.09), 144.48, 143.89 (143.85), 129.13 (129.09), 129.03 (128.94), 120.91 (120.78), 120.14 (120.08), 117.04 (116.88), 116.54 (116.36), 111.12 (111.07), 110.43 (110.27), 104.42 (104.38), 93.43, 93.36, 81.25 (81.15), 78.66, 76.09 (76.02), 74.35 (74.27), 62.35, 56.19 (56.17), 56.02 (55.99), 55.94, 55.70 (55.67), 42.15 (41.49), 25.90, 18.37, 13.89 (13.43), −5.07, −5.34. HRMS (ESI): *m*/*z* calculated for C_39_H_52_NO_11_Si [M+H]^+^: 738.3310. Found: 738.3329. IR (film) *ν*_max_: 2930, 2854, 1693, 1607, 1573, 1508 cm^−1^. HPLC purity 100% (only one signal was observed for the diastereomers).

#### 3.4.3. 23-*O*-TBS-5,7,20-*O*-Trimethyl-3-*O*-(*N*,*N*-dimethylthiocarbamoyl)silybin (**13**)

This derivative was achieved by PTLC purification using EtOAc/hexane (7:3, *v*/*v*) followed by hexane/EtOAc (1:1, *v*/*v*). Yield, 45%; slight yellow solid; 83–85 °C. ^1^H NMR (300 MHz, CDCl_3_) *δ* 7.16 (7.13) (d, *J* = 2.1 Hz, 1H, H-13), 7.09–6.95 (overlapped, 4H, H-15, H-16, H-18, H-22), 6.89 (d, *J* = 8.4 Hz, 1H, H-21), 6.60 (6.57) (d, *J* = 11.6 Hz, 1H, H-3), 6.12 (6.11) (d, *J* = 2.3 Hz, 1H, H-8), 6.09 (d, *J* = 2.3 Hz, 1H, H-6), 5.36 (d, *J* = 11.6 Hz, 1H, H-2), 5.01 (5.00) (d, *J* = 7.8 Hz, 1H, H-11), 3.98 (ddd, *J* = 7.8, 4.2, 2.7 Hz, 1H, H-10), 3.90 (s, 3H, OCH_3_), 3.90 (3.89) (s, 3H, OCH_3_), 3.86 (s, 3H, OCH_3_), 3.90–3.82 (overlapped, 1H, H-23), 3.80 (s, 3H, OCH_3_), 3.55 (dd, *J* = 11.7, 3.0 Hz, 1H, H-23), 3.34 (s, 3H, *N*CH_3_), 3.10 (s, 3H, *N*CH_3_), 0.90 (s, 9H, TBS), 0.06 (s, 3H, TBS), 0.06 (0.05) (s, 3H, TBS). ^13^C NMR (75 MHz, CDCl_3_) *δ* 187.53, 187.49, 166.46, 164.02, 162.40, 149.49, 149.17, 144.74, 143.98, 129.14 (129.09), 128.42 (128.33), 121.05, 120.20, 117.29 (117.17), 116.93 (116.80), 111.18 (111.15), 110.53 (110.38), 104.37, 93.48, 93.46, 81.17, 78.68 (78.64), 77.4, 76.22 (76.16), 62.41, 56.20, 56.06, 56.02, 55.79, 43.37, 38.33, 25.98, 18.44, −5.00, −5.29. HRMS (ESI): *m*/*z* calculated for C_37_H_48_NO_10_SSi [M+H]^+^: 726.2769. Found: 726.2796. IR (film) *ν*_max_: 2929, 2854, 1690, 1607, 1573, 1508 cm^−1^. HPLC purity 96.7% (only one signal was observed for the diastereomers).

### 3.5. Synthesis of Tetramethyltaxifolin (***14***)

A reaction flask was charged with taxifolin (20 mg, 0.066 mmol) and potassium carbonate (82 mg, 0.59 mmol), which was vacuumed three times under argon before the addition of anhydrous acetone (0.47 mL, 0.14 M). Dimethylsulfate (75 µL, 0.79 mmol) was then added into the reaction flask through a long needle. The reaction solution was refluxed overnight and cooled down to room temperature before the aqueous solution of ammonium chloride (20 mL) was added to quench the reaction. The subsequent mixture was extracted with ethyl acetate (10 mL × 3), the combined organic extracts were dried over anhydrous sodium sulfate, and the organic solvents were removed under vacuum. The crude product was purified over preparative TLC plate eluting with ethyl acetate/hexane (5:1, *v*/*v*) and ethyl acetate/hexane (7:3, *v*/*v*), sequentially, to furnish the desired **14** as a slight yellow solid in 86% yield; 149–151 °C; in [37], 165–166 °C; in [38], 153–155 °C. ^1^H NMR (300 MHz, CDCl_3_) *δ* 7.11 (dd, *J* = 8.3, 1.9 Hz, 1H, H-6′), 7.07 (d, *J* = 1.9 Hz, 1H, H-2′), 6.93 (d, *J* = 8.3 Hz, 1H, H-5′), 6.13 (d, *J* = 2.1 Hz, 1H, H-6), 6.11 (d, *J* = 2.1 Hz, 1H, H-8), 4.97 (d, *J* = 12.3 Hz, 1H, H-2), 4.45 (d, *J* = 12.3 Hz, 1H, H-3), 3.93 (s, 3H, OCH_3_), 3.92 (s, 3H, OCH_3_), 3.90 (s, 3H, OCH_3_), 3.82 (s, 3H, OCH_3_). ^13^C NMR (75 MHz, CDCl_3_) *δ* 190.93, 167.13, 165.06, 162.25, 149.91, 149.28, 129.02, 120.51, 111.23, 110.39, 103.02, 93.80, 93.43, 83.42, 72.72, 56.32, 56.11, 56.02, 55.89. HRMS (ESI): *m*/*z* calculated for C_19_H_21_O_7_ [M+H]^+^: 361.1287. Found: 361.1281. IR (film) *ν*_max_: 3446, 2939, 2838, 1673, 1606, 1572, 1516 cm^−1^. The NMR data are mostly consistent with those reported in the literature [39]. Only one exception is that the chemical shift for H-5′ was reported as 7.08 ppm in the literature [39] rather than at 6.93 ppm, without showing the original ^1^H NMR spectrum. Our chemical shift at H-5′ is closer to the value reported in another work [40].

### 3.6. General Procedure for the Synthesis of 3-Carbamoyled Derivatives of Tetramethyltaxifolin

Triethylamine (56 µL, 0.4 mmoL) and 4-dimethylaminopyridine (12 mg, 0.1 mmol) were sequentially added to a solution of tetramethyltoxifolin (**14**) (36 mg, 0.1 mmol) in DCM (1 mL, 0.1 M). The subsequent mixture was stirred for 10 min at room temperature, to which the respective (thio)carbamoyl chloride (0.4 mmol) was added. The reaction was allowed to proceed at room temperature with stirring overnight under argon prior to being quenched with brine (50 mL). The resulting mixture was extracted with ethyl acetate (30 mL × 3), the combined organic extracts were dried over anhydrous sodium sulfate, and the organic solvents were removed. The crude product was subjected to PTLC purification eluting with hexane/EtOAc (3:7, *v*/*v*) to afford the respective 3-carbomoyled derivative. Their physical and spectral data are summarized below.

#### 3.6.1. 3′,4′,5,7-*O*-Tetramethyl-3-*O*-(*N*,*N*-dimethylcarbamoyl)taxifolin (**15**)

Yield, 61%; white solid; 153–155 °C. ^1^H NMR (300 MHz, CDCl_3_) *δ* 7.04 (dd, *J* = 8.4, 1.8 Hz, 1H, H-6′), 7.03 (s, 1H, H-2′), 6.89 (d, *J* = 8.4 Hz, 1H, H-5′), 6.12 (d, *J* = 2.1 Hz, 1H, H-8), 6.10 (d, *J* = 2.1 Hz, 1H, H-6), 5.63 (d, *J* = 12.3 Hz, 1H, H-3), 5.32 (d, *J* = 12.3 Hz, 1H, H-2), 3.91 (s, 3H, OCH_3_), 3.90 (s, 3H, OCH_3_), 3.87 (s, 3H, OCH_3_), 3.81 (s, 3H, OCH_3_), 2.86 (s, 6H, 2 × *N*CH_3_). ^13^C NMR (75 MHz, CDCl_3_) *δ* 186.69, 166.47, 164.34, 162.51, 155.27, 149.84, 149.17, 128.46, 120.74, 111.08, 110.42, 104.47, 93.59, 93.51, 81.51, 74.34, 56.19, 56.08, 56.05, 55.78, 36.76, 36.16. HRMS (ESI): *m*/*z* calculated for C_22_H_26_NO_8_ [M+H]^+^: 432.1659. Found: 432.1657. IR (film) *ν*_max_: 2937, 2839, 1708, 1694, 1607, 1595, 1572, 1517 cm^−1^.

#### 3.6.2. 3-*O*-(*N*,*N*-Diethylcarbamoyl)-3′,4′,5,7-*O*-tetramethyltaxifolin (**16**)

Yield, 52%; colorless syrup. ^1^H NMR (300 MHz, CDCl_3_) *δ* 7.06 (d, *J* = 8.1, 2.1 Hz, 1H, H-6′), 7.03 (s, 1H, H-2′), 6.88 (d, *J* = 8.1 Hz, 1H, H-5′), 6.13 (d, *J* = 2.1 Hz, 1H, H-6 or H-8), 6.11 (d, *J* = 2.4 Hz, 1H, H-8 or H-6), 5.69 (d, *J* = 12.2 Hz, 1H, H-3), 5.31 (d, *J* = 12.2 Hz, 1H, H-2), 3.91 (s, 3H, OCH_3_), 3.90 (s, 3H, OCH_3_), 3.88 (s, 3H, OCH_3_), 3.81 (s, 3H, OCH_3_), 3.30–3.06 (m, 4H, 2 × CH_2_CH_3_), 1.02 (brs, 6H, 2 × CH_2_CH_3_). ^13^C NMR (75 MHz, CDCl_3_) *δ* 186.81, 166.37, 164.31, 162.47, 154.46, 149.80, 149.12, 128.45, 120.87, 111.02, 110.41, 104.53, 93.55, 93.50, 81.64, 73.91, 56.32, 56.13, 55.99, 55.71, 42.40, 41.63, 13.96, 13.47. HRMS (ESI): *m*/*z* calculated for C_24_H_30_NO_8_ [M+H]^+^: 460.1972. Found: 460.1970. IR (film) *ν*_max_: 2965, 2934, 2839, 1697, 1595, 1571, 1518 cm^−1^.

#### 3.6.3. 3′,4′,5,7-*O*-Tetramethyl-3-*O*-(*N*,*N*-dimethylthiocarbamoyl)taxifolin (**17**)

Yield, 38%; white solid; 152–154 °C. ^1^H NMR (300 MHz, CDCl_3_) *δ* 7.15 (d, *J* = 1.8 Hz, 1H, H-2′), 7.06 (dd, *J* = 8.1, 1.8 Hz, 1H, H-6′), 6.88 (d, *J* = 8.1 Hz, 1H, H-5′), 6.79 (d, *J* = 12.0 Hz, 1H, H-3), 6.12 (d, *J* = 2.4 Hz, 1H, H-8), 6.10 (d, *J* = 2.4 Hz, 1H, H-6), 5.36 (d, *J* = 12.0 Hz, 1H, H-2), 3.92 (s, 3H, OCH_3_), 3.89 (s, 3H, OCH_3_), 3.87 (s, 3H, OCH_3_), 3.81 (s, 3H, OCH_3_), 3.31 (s, 3H, *N*CH_3_), 3.06 (s, 3H, *N*CH_3_). ^13^C NMR (75 MHz, CDCl_3_) *δ* 187.53, 187.49, 166.47, 164.10, 162.41, 149.99, 149.21, 127.67, 121.47, 110.94, 110.81, 104.42, 93.59, 93.50, 81.56, 78.74, 56.30, 56.15, 55.94, 55.88, 43.41, 38.31. HRMS (ESI): *m*/*z* calculated for C_22_H_26_NO_7_S [M+H]^+^: 448.1430. Found: 448.1431. IR (film) *ν*_max_: 2937, 2895, 2838, 1687, 1607, 1572, 1518 cm^−1^.

### 3.7. Cell Culture

The LNCaP, DU145, and PC-3 prostate cancer cell lines were originally purchased from American Type Culture Collection (ATCC, Manassas, VA, USA). The PC-3 and LNCaP cell lines were routinely cultured in RPMI-1640 medium supplemented with 10% FBS and 1% penicillin/streptomycin. Cultures were maintained in a high humidity environment supplemented with 5% carbon dioxide at a temperature of 37 °C. The DU-145 prostate cancer cells were routinely cultured in Eagle’s Minimum Essential Medium (EMEM) supplemented with 10% FBS and 1% penicillin/streptomycin.

### 3.8. WST-1 Cell Proliferation Assay

The LNCaP, DU-145, or PC-3 prostate cancer cells were plated in 96-well plates at a density of 3200 in each well in 200 µL of culture medium. The cells were then treated with positive reference, or synthesized mimics separately at different doses for 3 days, while equal treatment volumes of DMSO were used as the vehicle control. The cells were cultured in a CO_2_ incubator at 37 °C for three days. An amount of 10 µL of the premixed WST-1 cell proliferation reagent (Clontech) was added to each well. After mixing gently for 1 min on an orbital shaker, the cells were incubated for an additional 3 h at 37 °C. To ensure homogeneous distribution of color, it was important to mix gently on an orbital shaker for 1 min. The absorbance of each well was measured using a microplate-reader (Synergy HT, BioTek) at a wavelength of 430 nm. The IC_50_ value was the concentration of each compound that inhibits cell proliferation by 50% under the experimental conditions and was the average from triplicate determinations that were reproducible and statistically significant. For calculating the IC_50_ values, a linear proliferative inhibition was made based on at least five dosages for each compound.

### 3.9. Statistical Analysis

All data are represented as the mean ± standard deviation (S.D.) for the number of experiments indicated. Other differences between treated and control groups were analyzed using the Student’s t-test. A *p*-value < 0.05 was considered statistically significant.

## 4. Conclusions

Collectively, the regioselective carbamoylation at the secondary alcoholic hydroxyl group at C-3 of flavanonols and flavanonol-type flavonolignans was developed for the first time. This approach avoids the oxidation of the C2–C3, offering a practical approach to the synthesis of 3-*O*-carbamoyl derivatives of flavanonols and flavanonol-type flavonolignans. Our preliminary WST cell proliferation assay data exhibit that 5,7,20-*O*-trimethylsilybin and four 3-*O*-carbamoyl-5,7,20-*O*-trimethylsilybins bear high selectivity and promising potency towards the AR-positive LNCaP prostate cancer cell line. These findings advocate that 3-*O*-substituted-5,7,20-*O*-trimethylsilybin may serve as a natural product-based scaffold for new antiandrogens for lethal castration-resistant prostate cancer. Further evaluation of their (including their optically pure versions) promising potency and selectivity in other AR-positive prostate cancer cell lines, as well as their AR modulating capability, are in progress and will be reported in due course.

## Figures and Tables

**Table 1:** Reaction conditions and yields for the carbamoylation of 5,7,20-*O*-trimethylsilybin (**3**).

Entry	(Thio)carbamoyl Chloride	Reaction Conditions					SM (%) ^a^	3-Carbamoyl- product (%) ^a^	Dicarbamoyl- product (%) ^a^
		Et_3_N	DMAP	DCM	Temp.	Time			
1	Me_2_NCOCl (4 eq)	4 eq	1 eq	0.1 M	r.t.	16 h	**3** (0%)	**5** (79%)	**4** (13%)
2	Et_2_NCOCl (4 eq)	4 eq	1 eq	0.1 M	r.t.	16 h	**3** (50%)	**6** (34%)	**7** (9%)
3	Me_2_NCSCl (4 eq)	4 eq	1 eq	0.1 M	r.t.	16 h	**3** (59%)	**8** (25%)	**9** (7%)
4	Me_2_NCOCl (4 eq)	4 eq	1 eq	0.1 M	50 °C	16 h	**3** (0%)	**5** (73%)	**4** (23%)
5	Et_2_NCOCl (4 eq)	4 eq	1 eq	0.1 M	50 °C	16 h	**3** (29%)	**6** (46%)	**7** (19%)
6	Me_2_NCSCl (4 eq)	4 eq	1 eq	0.1 M	50 °C	16 h	**3** (40%)	**8** (42%)	**9** (14%)
7	Me_2_NCOCl (6 eq)	6 eq	3 eq	0.2 M	50 °C	16 h	**3** (0%)	**5** (58%)	**4** (38%)
8	Et_2_NCOC (6 eq)	6 eq	3 eq	0.2 M	50 °C	16 h	**3** (0%)	**6** (69%)	**7** (24%)
9	Me_2_NCSCl (6 eq)	6 eq	3 eq	0.2 M	50 °C	16 h	**3** (7%)	**8** (58%)	**9** (25%)

^a^ Isolated yields.

**Table 2:** NMR data for **5** (^1^H NMR: 300 MHz; ^13^C NMR: 75 MHz).

Position	5 (CDCl_3_)				
	δ_C_,	Type	δ_H_, (*J* in Hz)		HMBC (H→C)
2	80.96 (80.89),	CH	5.29 (5.27)	d (11.8)	C-4, C-13, C-14, C-15
3	74.67 (74.52),	CH	5.51 (5.49)	d (11.8)	C-4, C-14, 3-OC(O)*N*-
4	186.40,	C	-		-
4a	104.24,	C	-		-
5	164.18,	C	-		-
6	93.47,	CH	6.09–6.06, overlapped		C-4a, C-7, C-8
7	166.43,	C	-		-
8	93.38,	CH	6.09–6.06, overlapped		C-6, C-7, C-8a
8a	162.39,	C	-		-
10	78.36,	CH	4.02, ddd (8.1, 5.4, 3.9)		C-11
11	76.28 (76.20),	CH	4.95 (4.93), d (8.1)		C-17, C-18, C-22
12a	144.03 (144.00),	C	-		-
13	116.47,	CH	7.13, d	(2.4)	C-12a, C-14, C-15
14	129.56 (129.43),	C	-		-
15	120.95 (120.78),	CH	7.00–6.91, overlapped		C-14, C-15a
16	117.11 (117.02),	CH	7.00–6.91, overlapped		C-14, C-16a
16a	144.03 (144.00),	C	-		-
17	128.55 (128.52),	C	-		-
18	110.13 (109.99),	CH	7.00–6.91, overlapped		C-11, C-19, C-22
19	149.64,	C	-		-
20	149.41 (149.35),	C	-		-
21	111.24 (111.18),	CH	6.87, d (8.1)		C-17, C-20
22	120.19,	CH	7.00–6.91, overlapped		C-17, C-18
23	61.64,	CH_2_	3.87–3.75, overlapped 3.51, dd (12.6, 3.9)		C-11
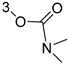	155.28,	C	-		-
	36.75 (36.08),	CH_3_	2.85, s		3-*O*-C(O)*N*(CH_3_)_2_
5-OMe	56.09, CH_3_		3.83, s		C-5
7-OMe	55.70, CH_3_		3.78, s		C-7
19-OMe	56.01, CH_3_		3.87, s		C-19
20-OMe	56.01, CH_3_		3.89, s		C-20

**Table 3:** Seventy-two hours of antiproliferative activity of carbamoyled 5,7,20-*O*-trimethylsilybins.

Compounds	IC_50_, µM ^a^	IC_50_, µM ^a^	IC_50_, µM ^a^
	PC-3	DU145	LNCaP
**Enzalutamide**	>25	>25	40–60% inhibition at 1–25 µM
**Silibinin**	72.65 ± 3.15	93.34 ± 13.67	43.73 ± 10.90
**3**	7.41 ± 1.30	22.41 ± 0.87	0.41 ± 0.08
**4**	17.83 ± 1.09	>25	0.25 ± 0.04
**5**	12.49 ± 1.91	>25	0.83 ± 0.04
**6**	>25	>25	0.11± 0.03
**7**	>25	>25	3.76 ± 0.71
**8**	>25	>25	0.82 ± 0.03
**9**	>25	>25	15.49 ± 2.96
**11**	>25	>25	29.41 ± 2.25
**12**	>25	>25	1.95 ± 0.19
**13**	>25	>25	17.66 ± 2.41

^a^ IC_50_ is the compound concentration effective in suppressing 50% of the cell viability measured by WST-1 cell proliferation assay after a 3-day exposure. The data were presented as the mean ± SD from triplicate determinations.

## Supplementary Materials

The following are available online. Figures S1–S5, S8, S9, S12, S13, S16, S17, S20, S21, S24, S25, S28, S29, S31, S32, S34, S35, S38, S39, S42, S43, S45, S46, S48, S49, S51 and S52: NMR spectra, Figures S6, S10, S14, S18, S22, S26, S30, S33, S36, S40, S44, S47, S50, & S53: High resolution mass spectra, Figures S7, S11, S15, S19, S23, S27, S33, S37 and S41: HPLC chromatograms, Figure S54: The concentration-effect curves.

## Sample Availability

Samples of the compounds **4**–**17** are available from the authors.
