# Supplementary material for: 3-O-Carbamoyl-5,7,20-O-trimethylsilybins: Synthesis and Preliminary Antiproliferative Evaluation
Source: Molecules. 2021 Oct 24;26(21):6421. doi: 10.3390/molecules26216421 (PMC8588252; doi:10.3390/molecules26216421)
Supplement: Supplementary file 1 [file molecules-26-06421-s001.zip › molecules-1416461-supplementary.pdf]

Electronic Supplementary Information

3-*O*-Carbamoyl-5,7,20-*O*-trimethylsilybins: Synthesis and preliminary antiproliferative evaluation

Sitong Wu[a], Guanglin Chen[a], Qiang Zhang[b,c], Guangdi Wang[b,c], and Qiao-Hong Chen*[a]

[a]Department of Chemistry and Biochemistry, California State University, Fresno, 2555 E. San Ramon Avenue, M/S SB70, Fresno, CA 93740, USA

[b]Department of Chemistry and [c]RCMI Cancer Research Center, Xavier University of Louisiana, 1 Drexel Drive, New Orleans, LA 70125, USA

List of Contents:

Figure S1: ^{1}H NMR spectrum of **5** in $CDCl_3$……S3
Figure S2: ^{13}C NMR spectrum of **5** in $CDCl_3$……S4
Figure S3: COSY spectrum of **5** in $CDCl_3$……S5
Figure S4: HSQC spectrum of **5** in $CDCl_3$……S6
Figure S5: HMBC spectrum of **5** in $CDCl_3$……S7
Figure S6: High resolution mass spectrum of **5** ……S8
Figure S7: HPLC chromatogram of **5**……S9
Figure S8: ^{1}H NMR spectrum of **4** in $CDCl_3$……S10
Figure S9: ^{13}C NMR spectrum of **4** in $CDCl_3$……S11
Figure S10: High resolution mass spectrum of **4** ……S12
Figure S11: HPLC chromatogram of **4**……S13
Figure S12: ^{1}H NMR spectrum of **6** in $CDCl_3$……S14
Figure S13: ^{13}C NMR spectrum of **6** in $CDCl_3$……S15
Figure S14: High resolution mass spectrum of **6** ……S16
Figure S15: HPLC chromatogram of **6**……S17
Figure S16: ^{1}H NMR spectrum of **7** in $CDCl_3$……S18
Figure S17: ^{13}C NMR spectrum of **7** in $CDCl_3$……S19
Figure S18: High resolution mass spectrum of **7** ……S20
Figure S19: HPLC chromatogram of **7**……S21
Figure S20: ^{1}H NMR spectrum of **8** in $CDCl_3$……S22
Figure S21: ^{13}C NMR spectrum of **8** in $CDCl_3$……S23

Figure S22: High resolution mass spectrum of **8** ……………………………………………………………………........S24
Figure S23: HPLC chromatogram of **8**………………………………………………………………………………..S25
Figure S24: ^{1}H NMR spectrum of **9** in $CDCl_3$………………………………………………………………………S26
Figure S25: ^{13}C NMR spectrum of **9** in $CDCl_3$……………………………………………………………………...S27
Figure S26: High resolution mass spectrum of **9** ……………………………………………………………………...... S28
Figure S27: HPLC chromatogram of **9**………………………………………………………………………………...S29
Figure S28: ^{1}H NMR spectrum of **10** in $CDCl_3$……………………………………………………………………..S30
Figure S29: ^{13}C NMR spectrum of **10** in $CDCl_3$……………………………………………………………………S31
Figure S30: High resolution mass spectrum of **10** ……………………………………………………………….... S32
Figure S31: ^{1}H NMR spectrum of **11** in $CDCl_3$……………………………………………………………………..S33
Figure S32: ^{13}C NMR spectrum of **11** in $CDCl_3$……………………………………………………………………S34
Figure S33: High resolution mass spectrum of **11** ……………………………………………………………….... S35
Figure S33: HPLC chromatogram of **11**………………………………………………………………………………S36
Figure S34: ^{1}H NMR spectrum of **12** in $CDCl_3$……………………………………………………………………..S37
Figure S35: ^{13}C NMR spectrum of **12** in $CDCl_3$……………………………………………………………………S38
Figure S36: High resolution mass spectrum of **12** ……………………………………………………………….... S39
Figure S37: HPLC chromatogram of **12**………………………………………………………………………………S40
Figure S38: ^{1}H NMR spectrum of **13** in $CDCl_3$……………………………………………………………………..S41
Figure S39: ^{13}C NMR spectrum of **13** in $CDCl_3$……………………………………………………………………S42
Figure S40: High resolution mass spectrum of **13** ……………………………………………………………….....S43
Figure S41: HPLC chromatogram of **13**………………………………………………………………………………S44
Figure S42: ^{1}H NMR spectrum of **14** in $CDCl_3$……………………………………………………………………..S45
Figure S43: ^{13}C NMR spectrum of **14** in $CDCl_3$……………………………………………………………………S46
Figure S44: High resolution mass spectrum of **14** ……………………………………………………………………S47
Figure S45: ^{1}H NMR spectrum of **15** in $CDCl_3$……………………………………………………………………..S48
Figure S46: ^{13}C NMR spectrum of **15** in $CDCl_3$……………………………………………………………………S49
Figure S47: High resolution mass spectrum of **15** ……………………………………………………………………S50
Figure S48: ^{1}H NMR spectrum of **16** in $CDCl_3$……………………………………………………………………..S51
Figure S49: ^{13}C NMR spectrum of **16** in $CDCl_3$……………………………………………………………………S52
Figure S50: High resolution mass spectrum of **16** ……………………………………………………………………S53
Figure S51: ^{1}H NMR spectrum of **17** in $CDCl_3$……………………………………………………………………..S54
Figure S52: ^{13}C NMR spectrum of **17** in $CDCl_3$……………………………………………………………………S55
Figure S53: High resolution mass spectrum of **17** ……………………………………………………………………S56
Figure S54: The concentration-effect curves …………………………………………………………………S57-S101

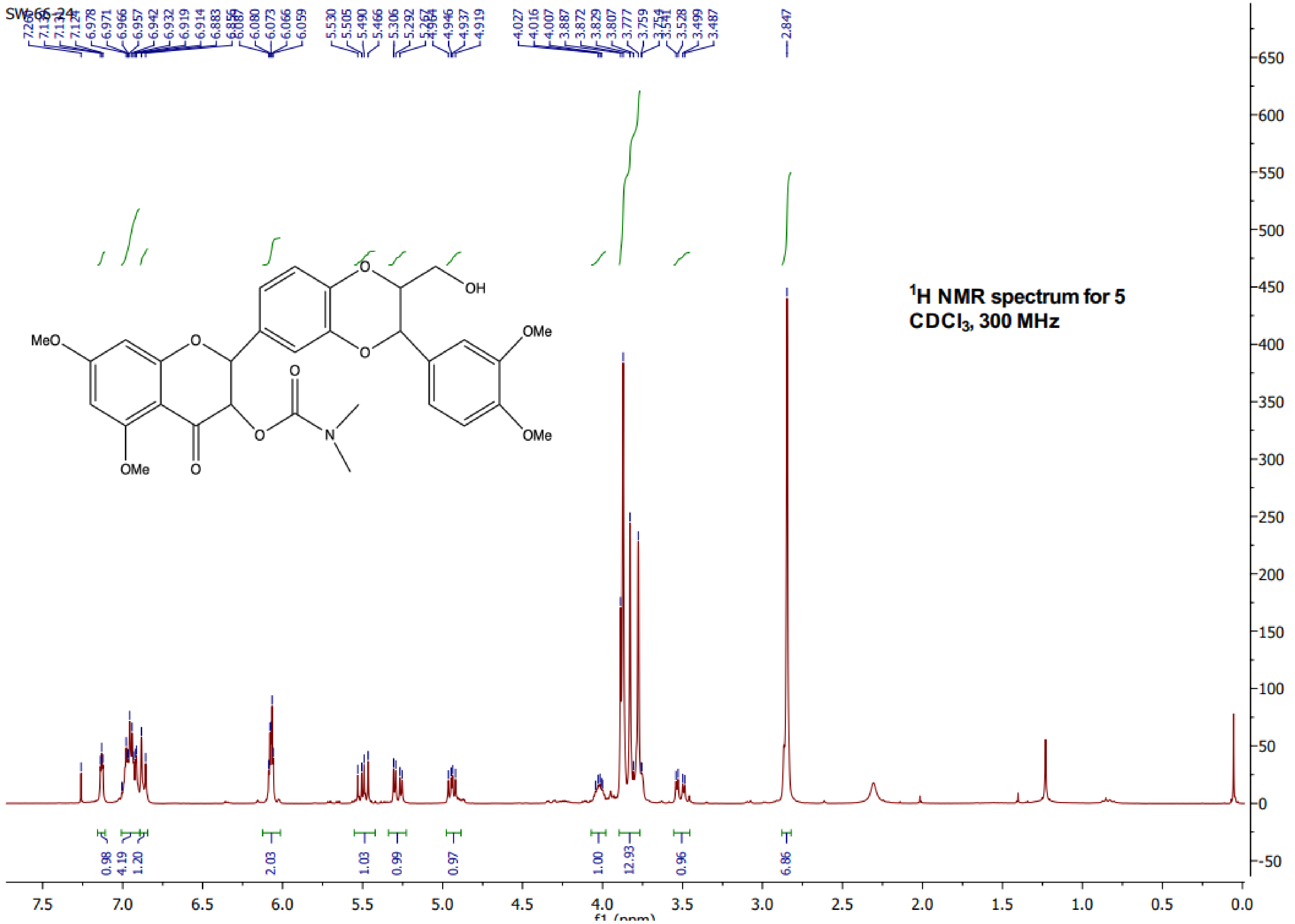

Figure S1: ^{1}H NMR spectrum of **5** in $CDCl_3$

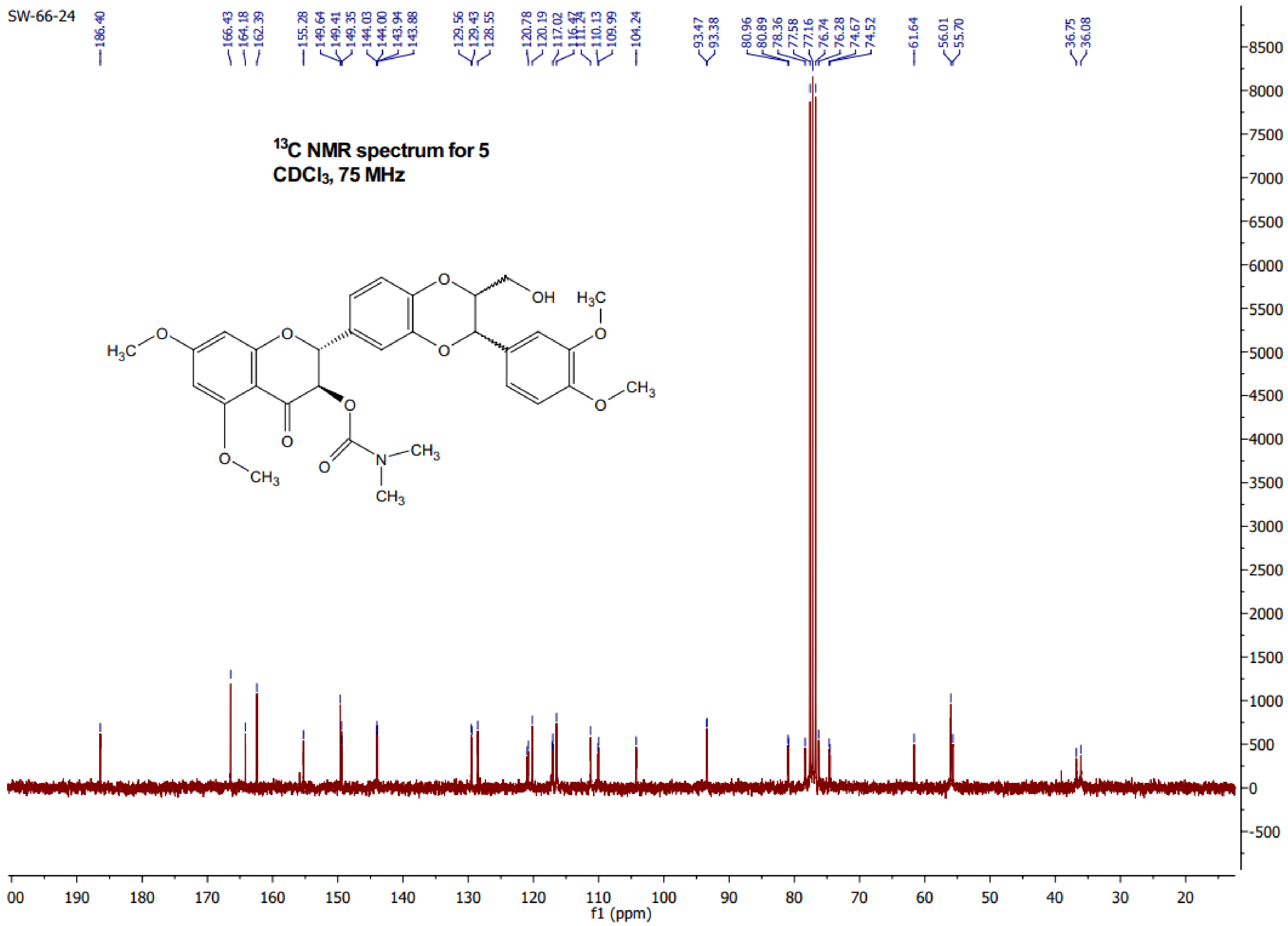

Figure S2: ^{13}C NMR spectrum of **5** in $CDCl_3$

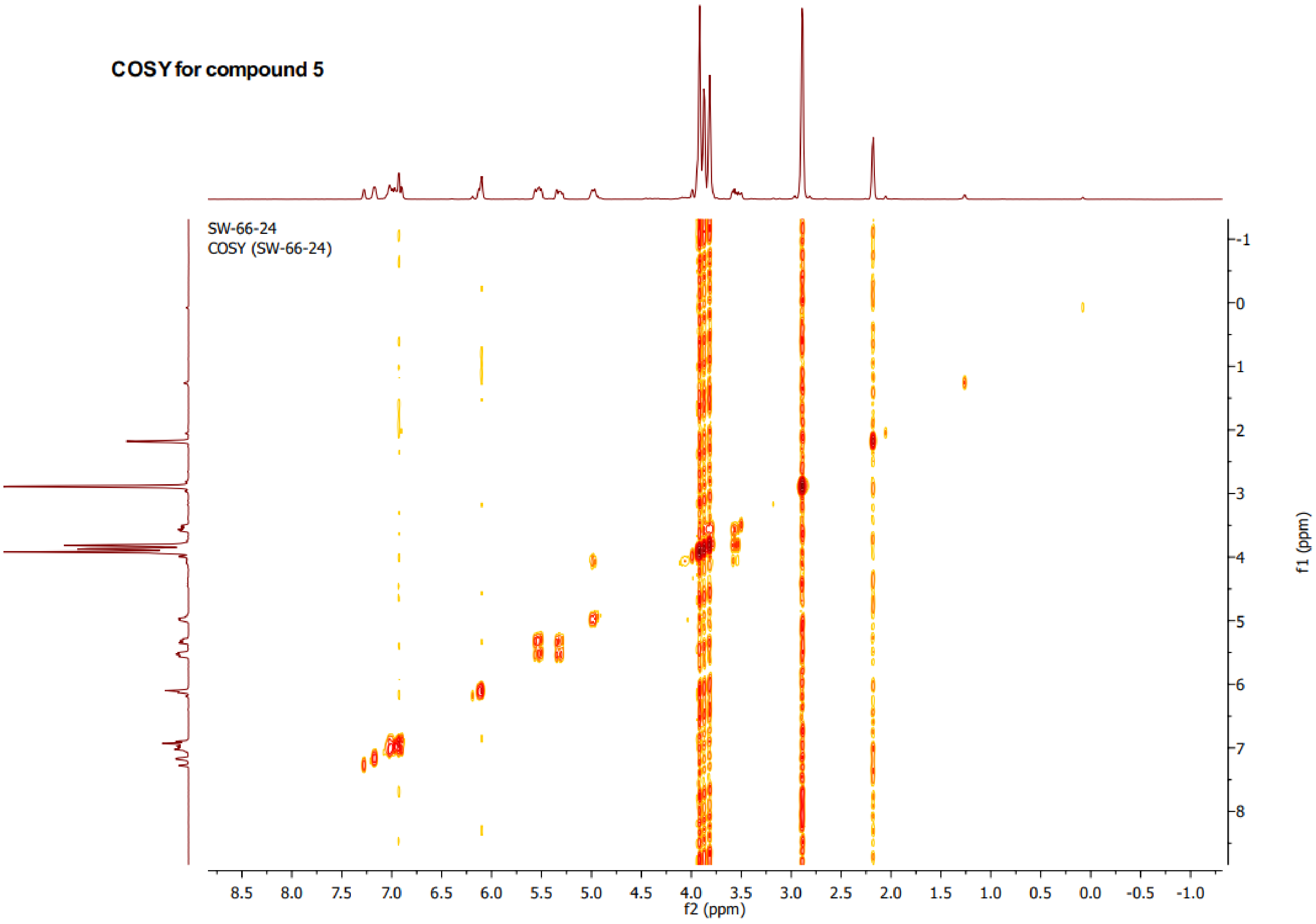

Figure S3: COSY spectrum of **5** in $CDCl_3$

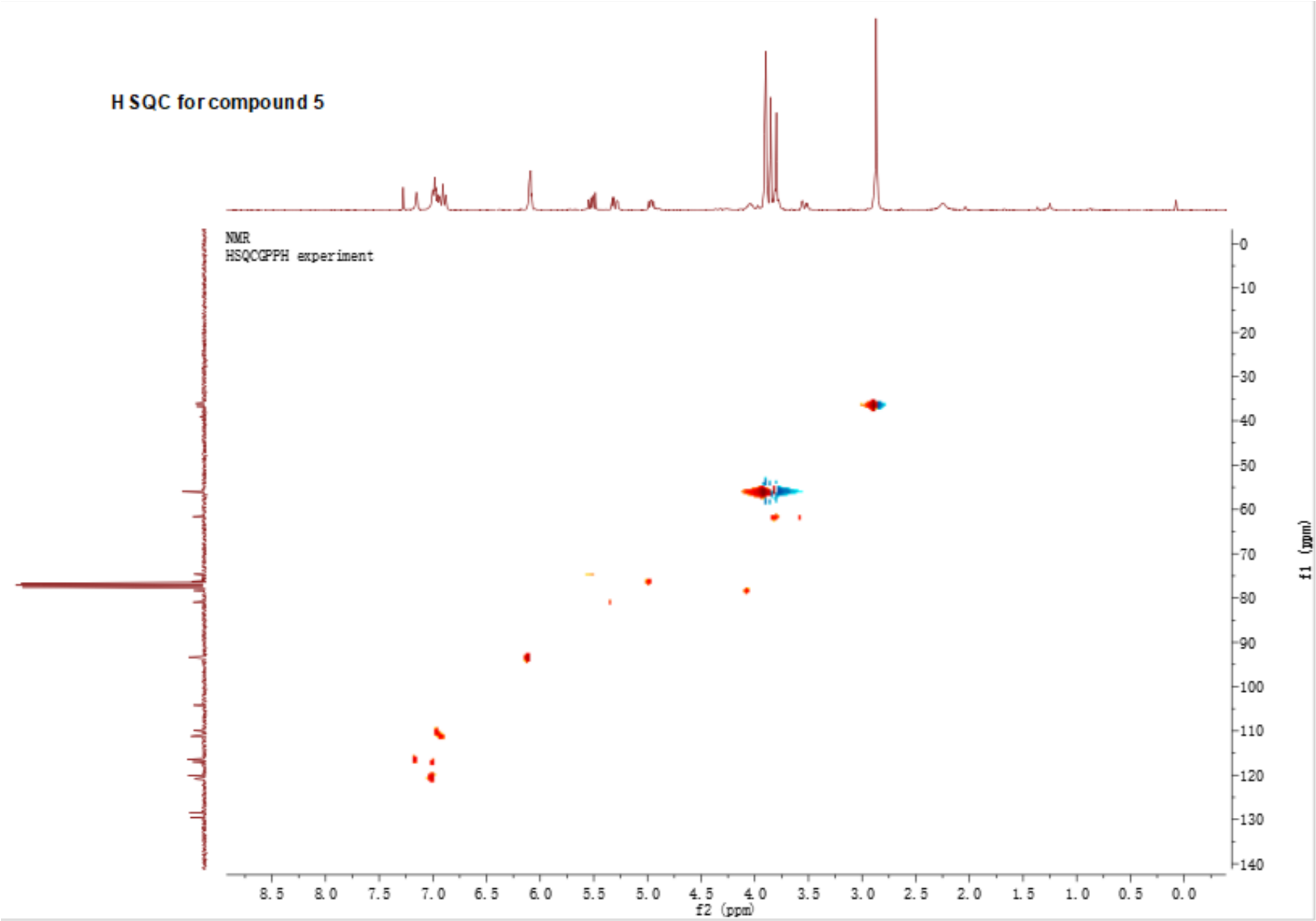

Figure S4: HSQC spectrum of **5** in $CDCl_3$

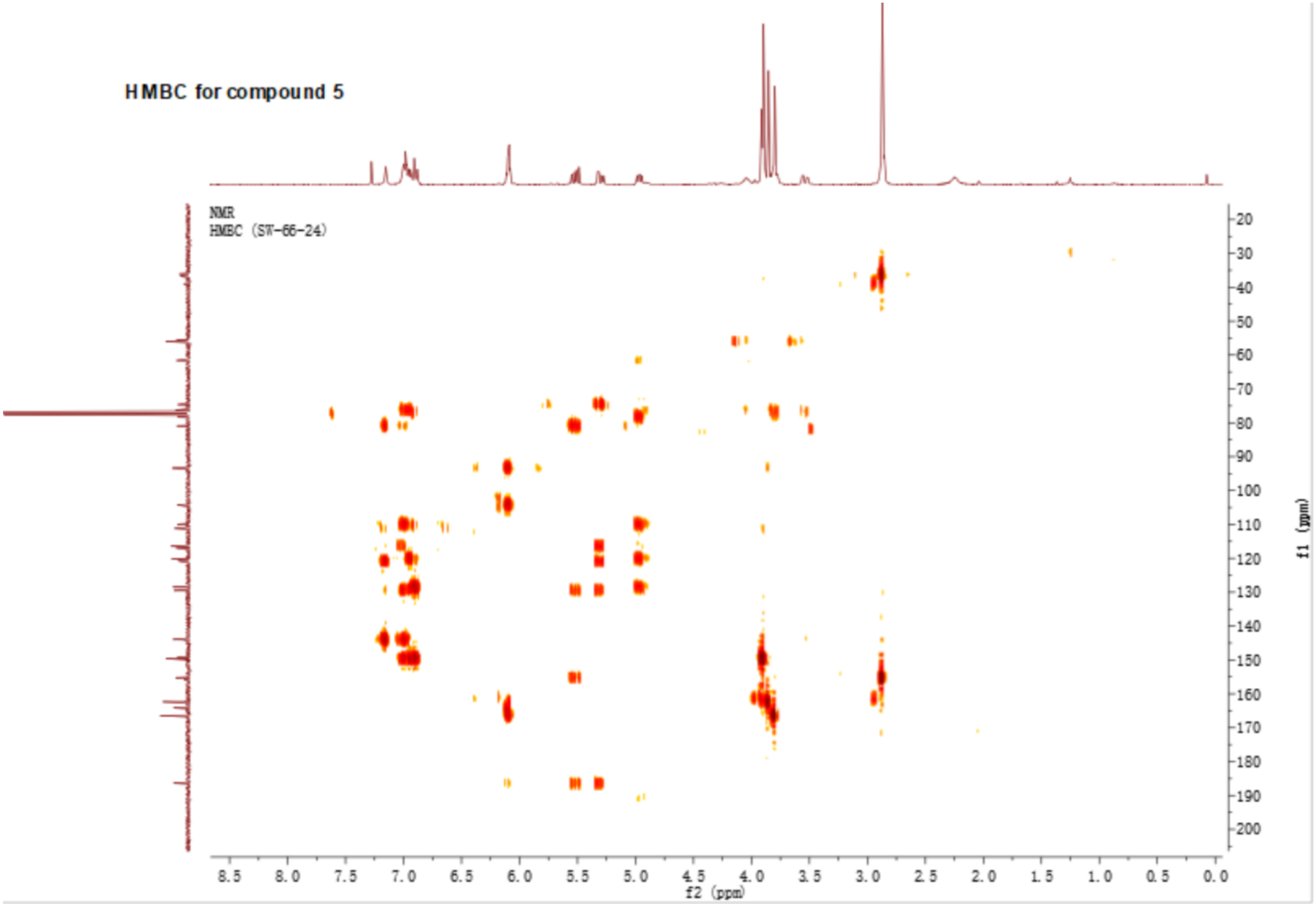

Figure S5: HMBC spectrum of **5** in $CDCl_3$

compds	Mol. Formula	Exact Mass	M+H	**Observed**	**delta**	**ppm**
QGC-68-136	C31H33NO11	595.2054	596.2132	**596.2128**	**-0.0004**	**-0.69**

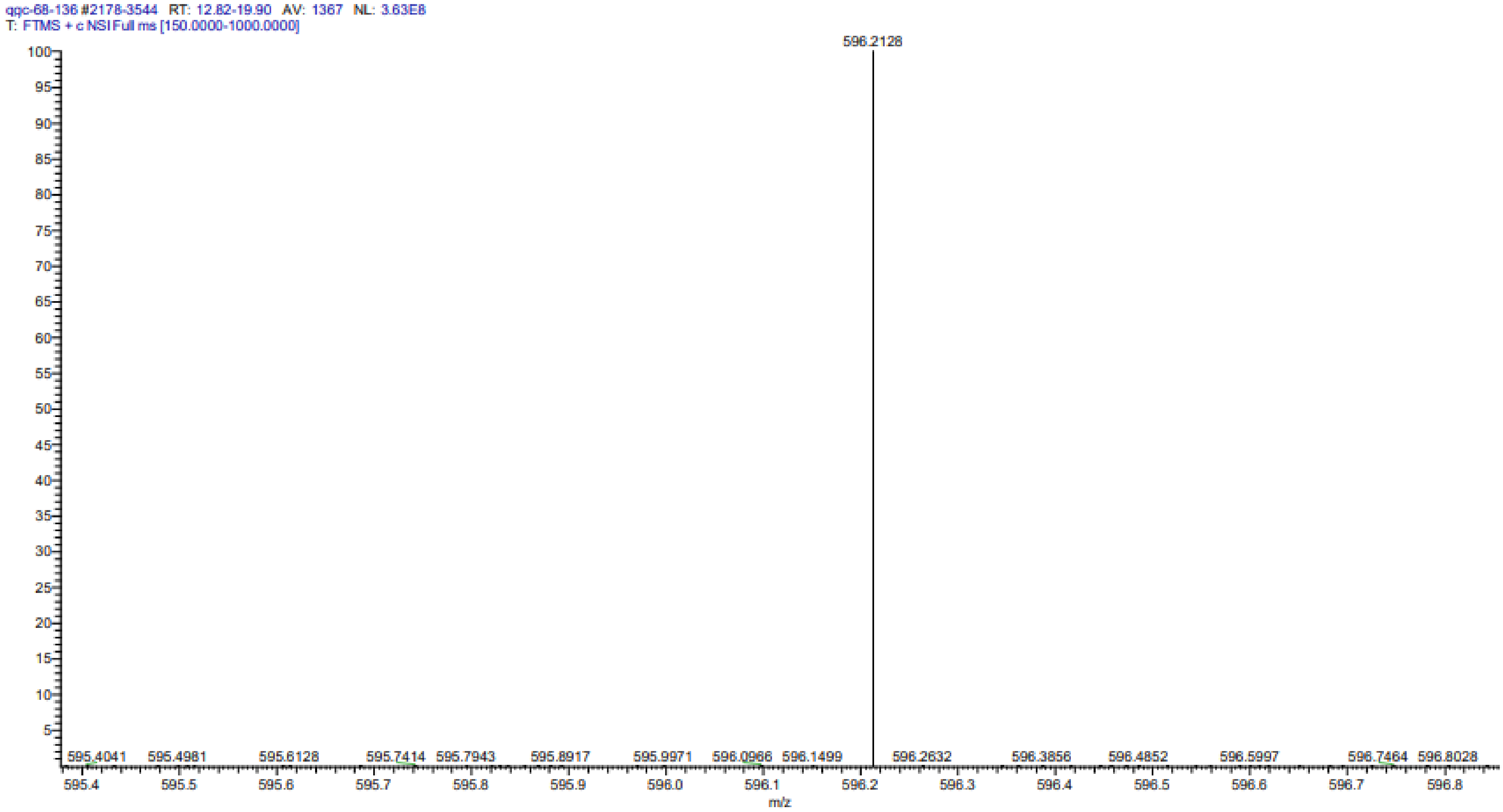

Figure S6: High resolution mass spectrum of **5**

Data File C:\HPCHEM\1\DATA\DEFAULT.D Sample Name: SW-66-24

===

```
Injection Date  : 9/17/2021 4:49:32 PM
Sample Name     : SW-66-24                    Location : Vial 1
Acq. Operator   :
Method          : C:\HPCHEM\1\METHODS\JNP2015.M
Last changed    : 9/17/2021 4:42:01 PM
                  (modified after loading)
```

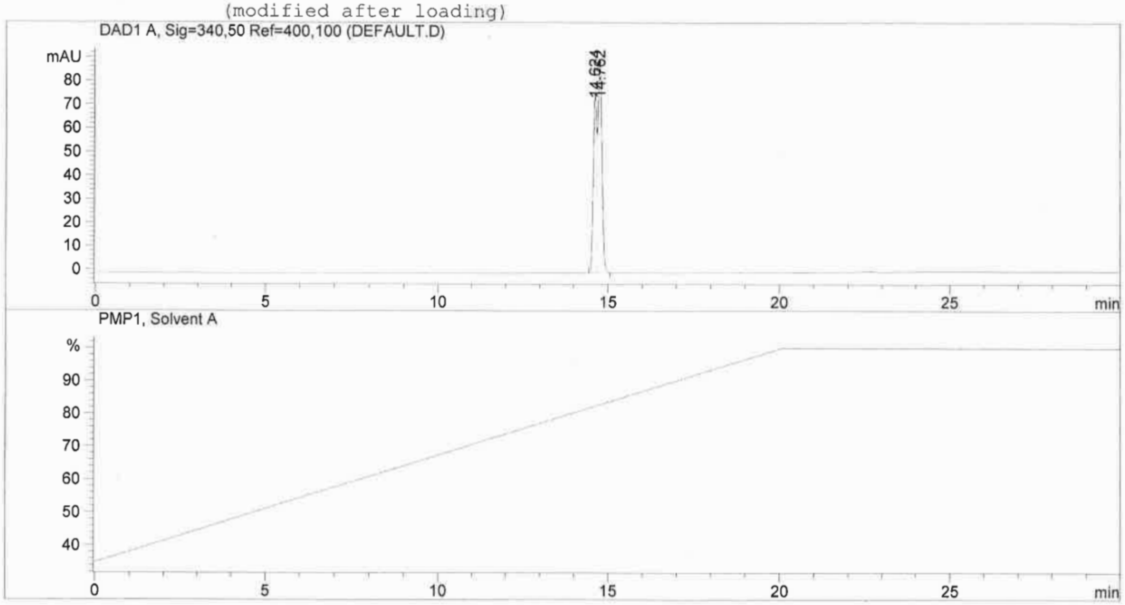

===

Area Percent Report

===

```
Sorted By       :      Signal
Multiplier      :      1.0000
Dilution        :      1.0000
```

Signal 1: DAD1 A, Sig=340,50 Ref=400,100

Peak #	RetTime [min]	Type	Width [min]	Area [mAU*s]	Height [mAU]	Area %
1	14.624	BV	0.1086	539.64001	76.22746	42.6786
2	14.762	VB	0.1216	724.78864	90.29533	57.3214
Totals :				1264.42865	166.52280	

Results obtained with enhanced integrator!

===

*** End of Report ***

Figure S7: HPLC chromatogram of **5**

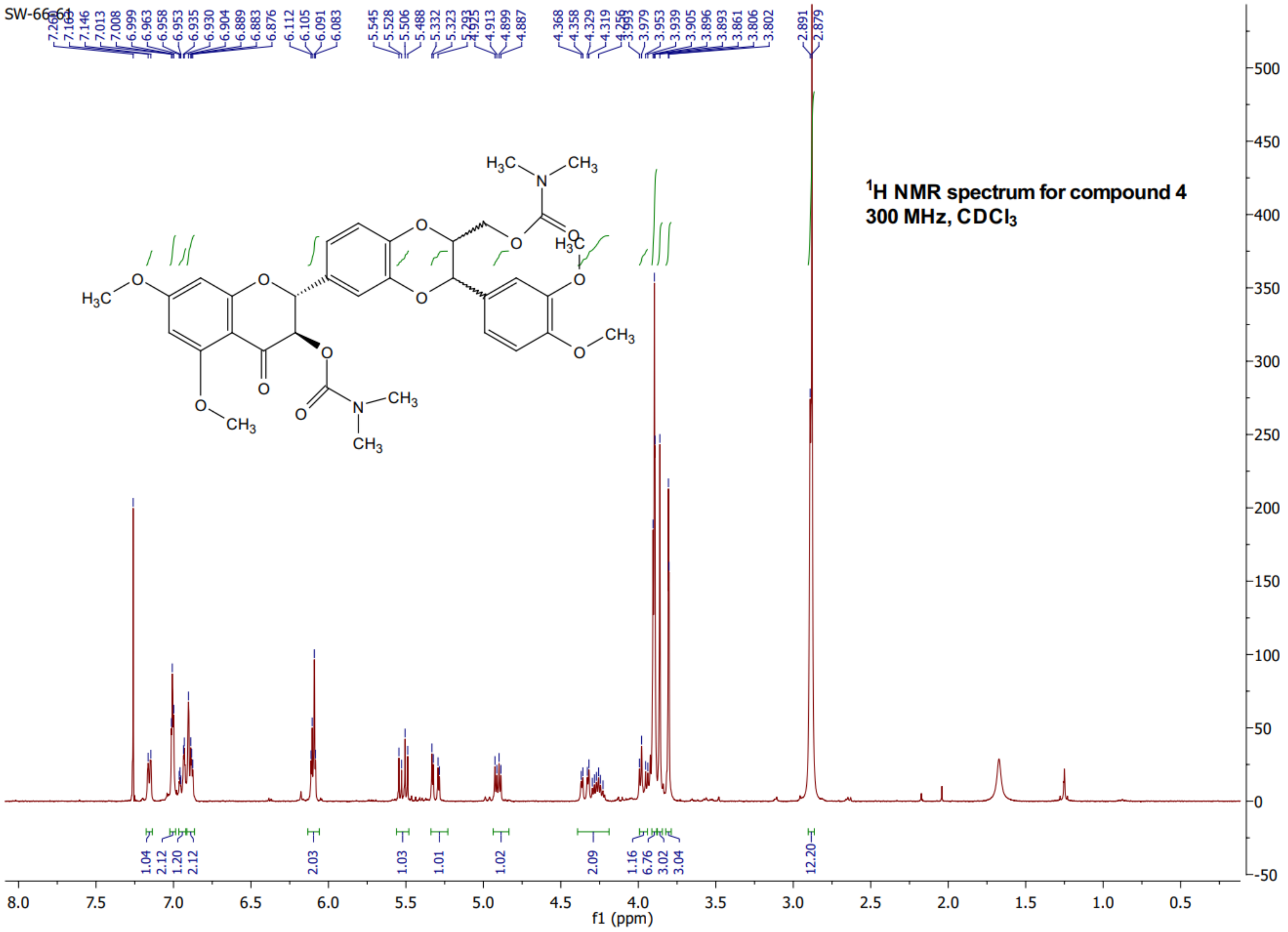

Figure S8: ^{1}H NMR spectrum of **4** in $CDCl_3$

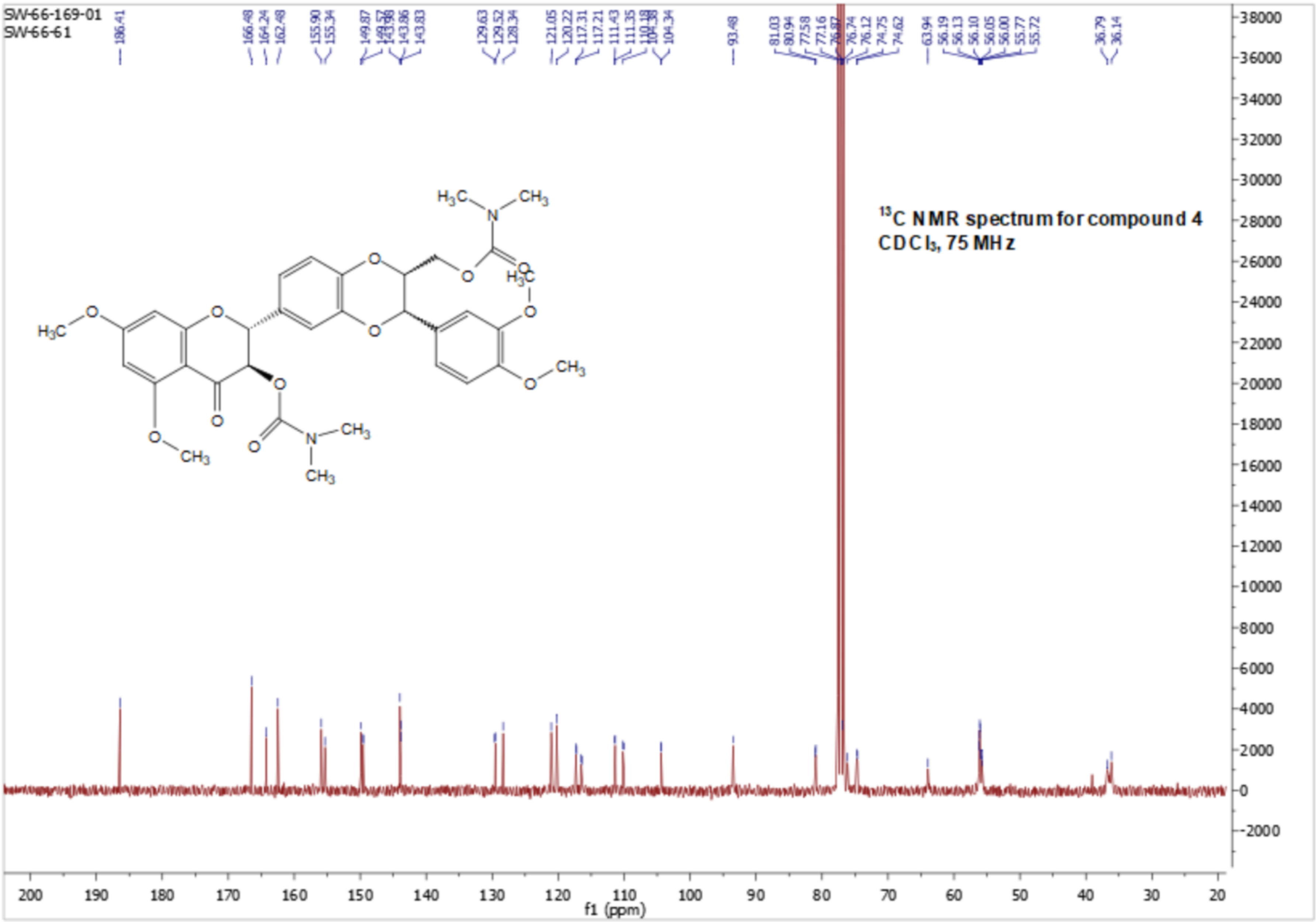

Figure S9: ^{13}C NMR spectrum of **4** in $CDCl_3$

compds	Mol. Formula	Exact Mass	M+H	**Observed**	delta	ppm
SW-66-61	C34H38N2O12	666.2425	667.2503	**667.2498**	-0.0005	-0.81

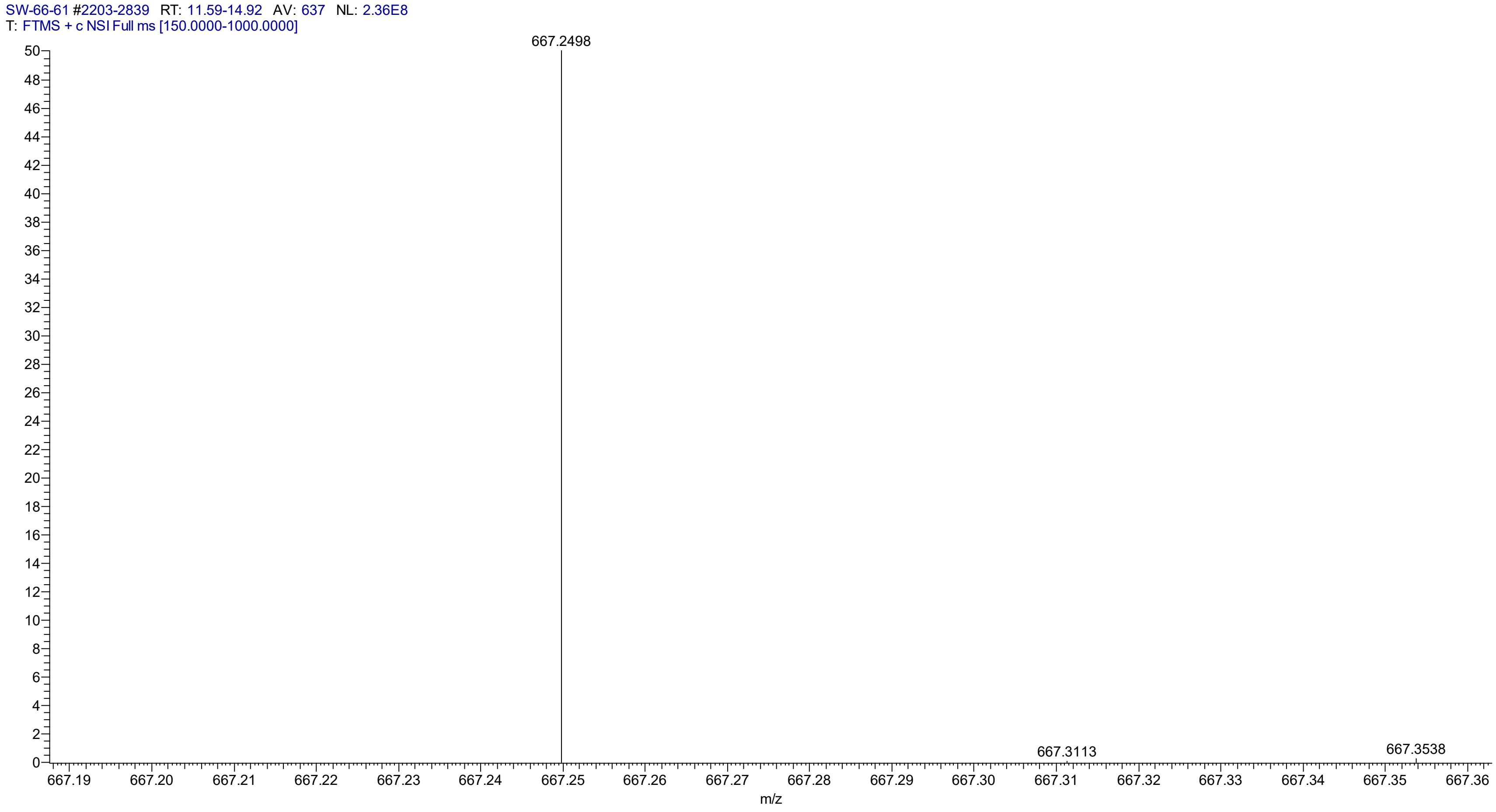

Figure S10: High resolution mass spectrum of **4**

Data File C:\HPCHEM\1\DATA\DEFAULT.D   Sample Name: SW-66-61

Injection Date : 9/16/2021 2:21:42 PM
Sample Name : SW-66-61   Location : Vial 1
Acq. Operator :
Method : C:\HPCHEM\1\METHODS\JNP2015.M
Last changed : 9/16/2021 2:14:33 PM
(modified after loading)

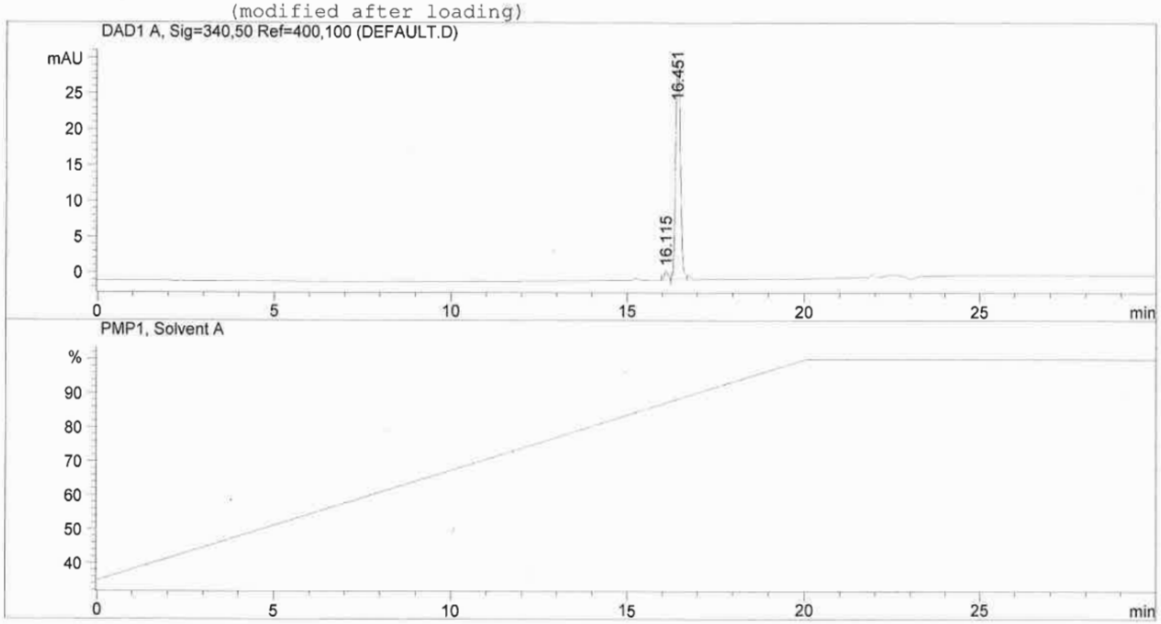

Area Percent Report

Sorted By : Signal
Multiplier : 1.0000
Dilution : 1.0000

Signal 1: DAD1 A, Sig=340,50 Ref=400,100

Peak #	RetTime [min]	Type	Width [min]	Area [mAU*s]	Height [mAU]	Area %
1	16.115	PV	0.1108	9.05094	1.27589	3.0535
2	16.451	VB	0.1501	287.36374	30.44066	96.9465

Totals : 296.41468 31.71655

Results obtained with enhanced integrator!

*** End of Report ***

Figure S11: HPLC chromatogram of **4**

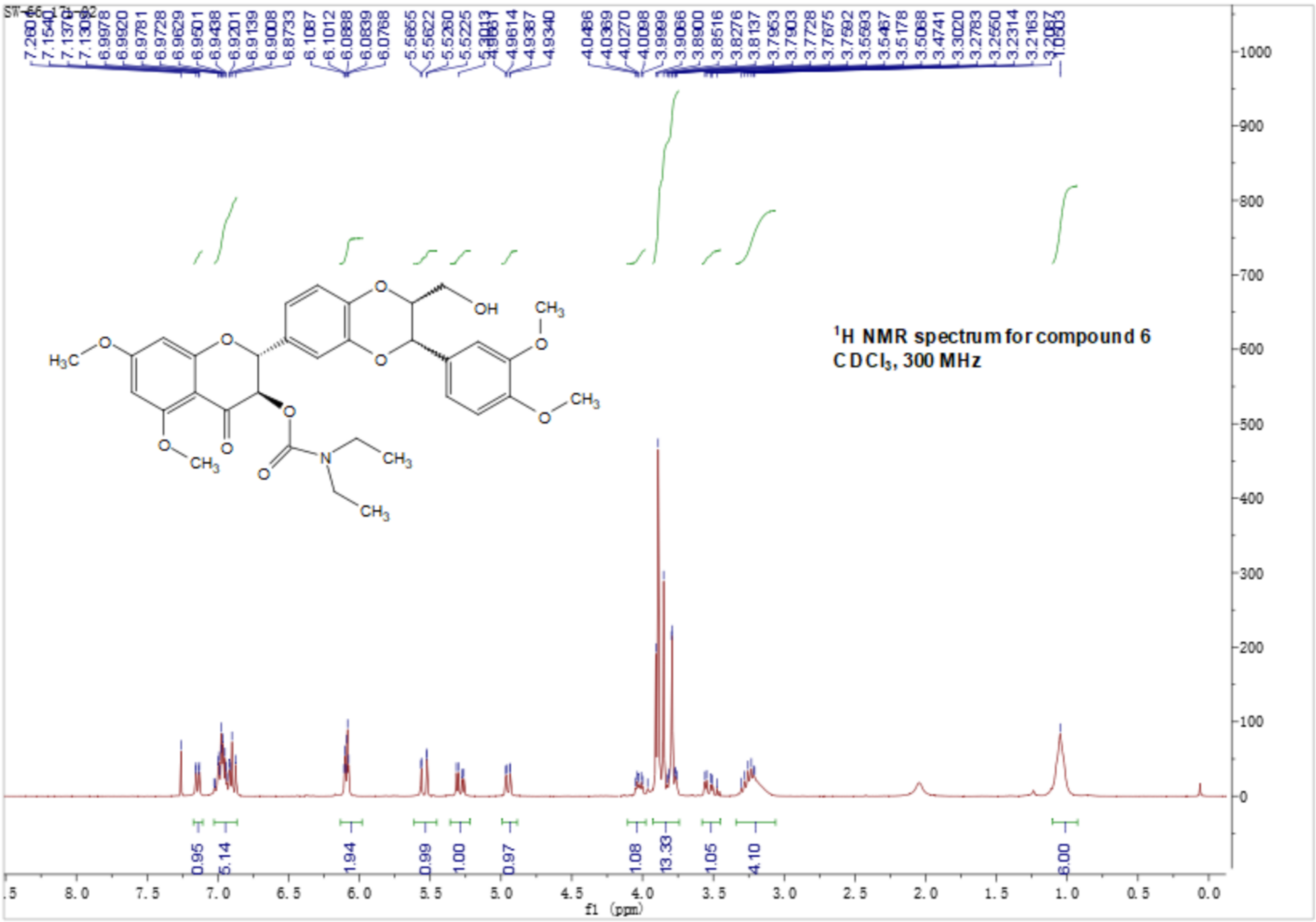

Figure S12: ¹H NMR spectrum of **6** in $CDCl_3$

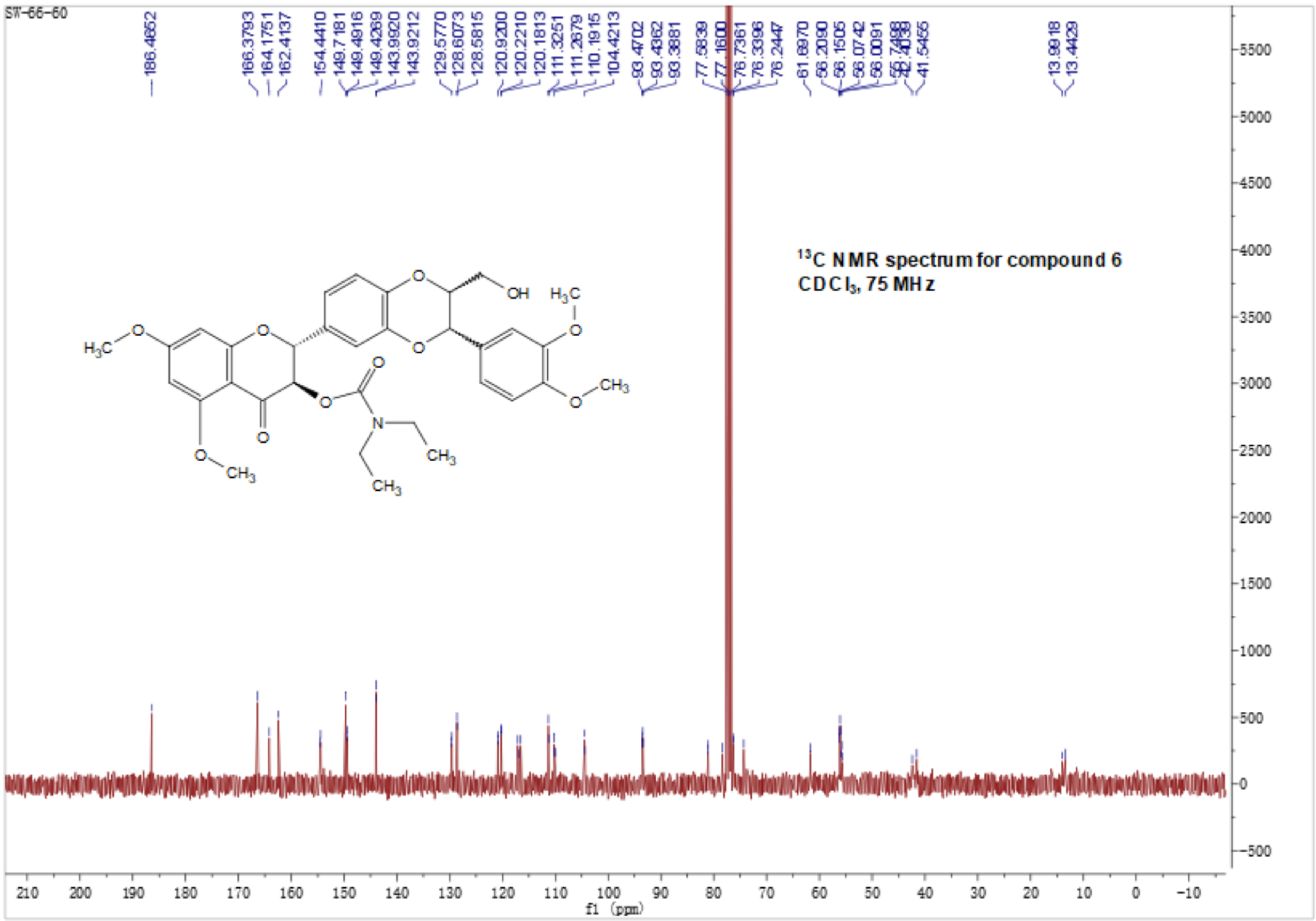

Figure S13: ^{13}C NMR spectrum of **6** in $CDCl_3$

compds	Mol. Formula	Exact Mass	M+H	**Observed**	delta	ppm
SW-66-60	C33H37NO11	623.2367	624.2445	**624.2443**	-0.0002	-0.34

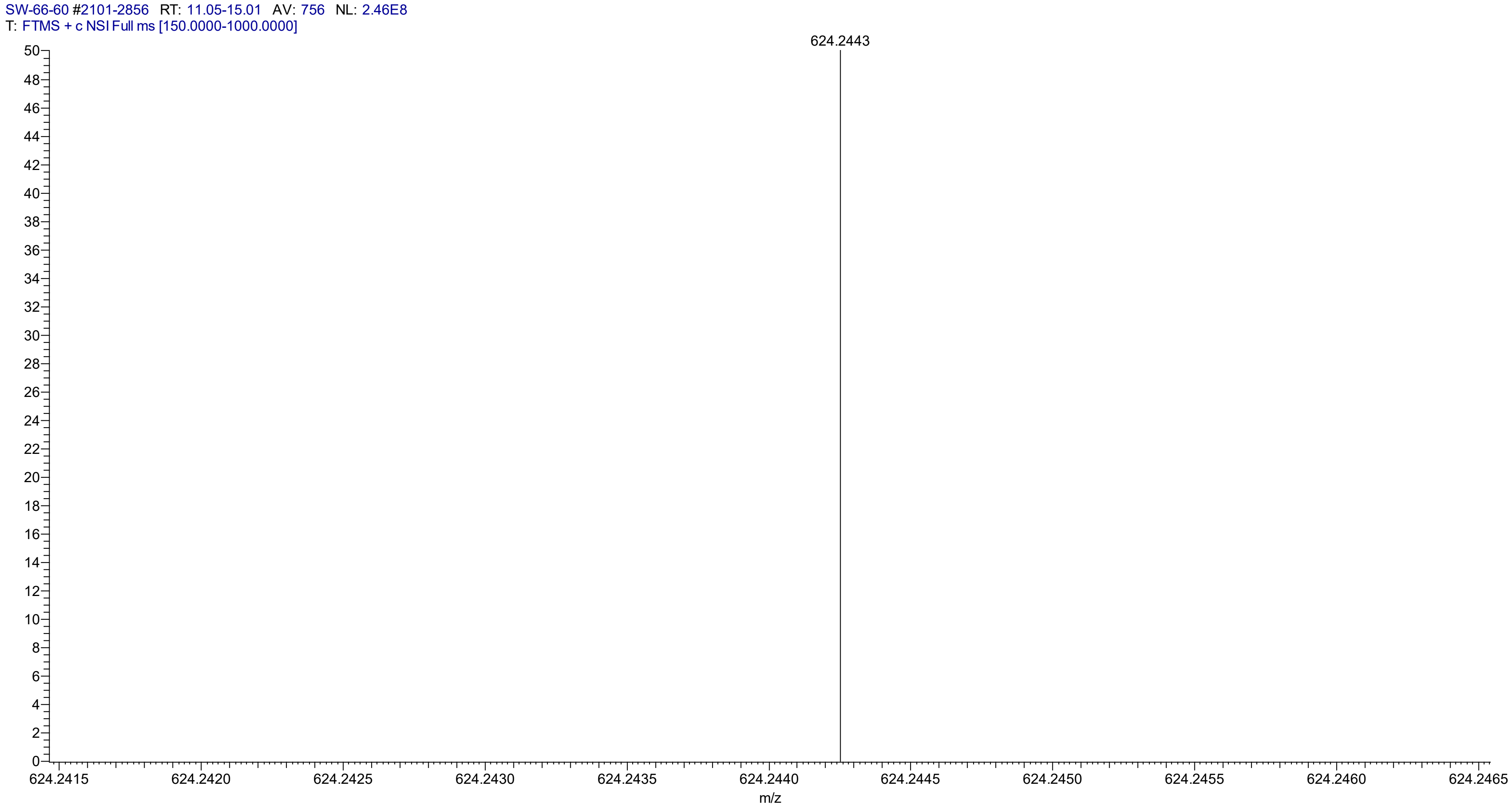

Figure S14: High resolution mass spectrum of **6**

Data File C:\HPCHEM\1\DATA\DEFAULT.D Sample Name: SW-66-60

Injection Date : 9/16/2021 3:34:17 PM
Sample Name : SW-66-60 Location : Vial 1
Acq. Operator :
Method : C:\HPCHEM\1\METHODS\JNP2015.M
Last changed : 9/16/2021 3:26:08 PM
(modified after loading)

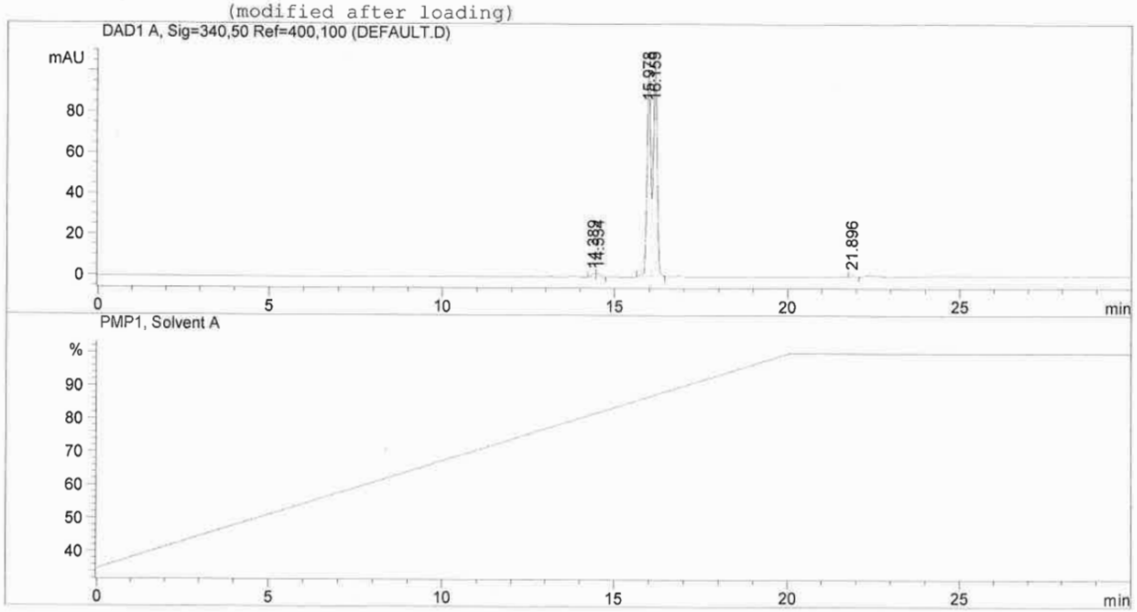

Area Percent Report

Sorted By : Signal
Multiplier : 1.0000
Dilution : 1.0000

Signal 1: DAD1 A, Sig=340,50 Ref=400,100

Peak #	RetTime [min]	Type	Width [min]	Area [mAU*s]	Height [mAU]	Area %
1	14.389	PV	0.1156	12.35465	1.64579	0.7808
2	14.534	VB	0.1193	13.75756	1.72044	0.8695
3	15.978	BV	0.1128	744.94830	100.14996	47.0812
4	16.159	VB	0.1149	802.74286	105.38542	50.7339
5	21.896	BP	0.0996	8.45884	1.34011	0.5346

Totals : 1582.26220 210.24171

Results obtained with enhanced integrator!

*** End of Report ***

Figure S15: HPLC chromatogram of **6**

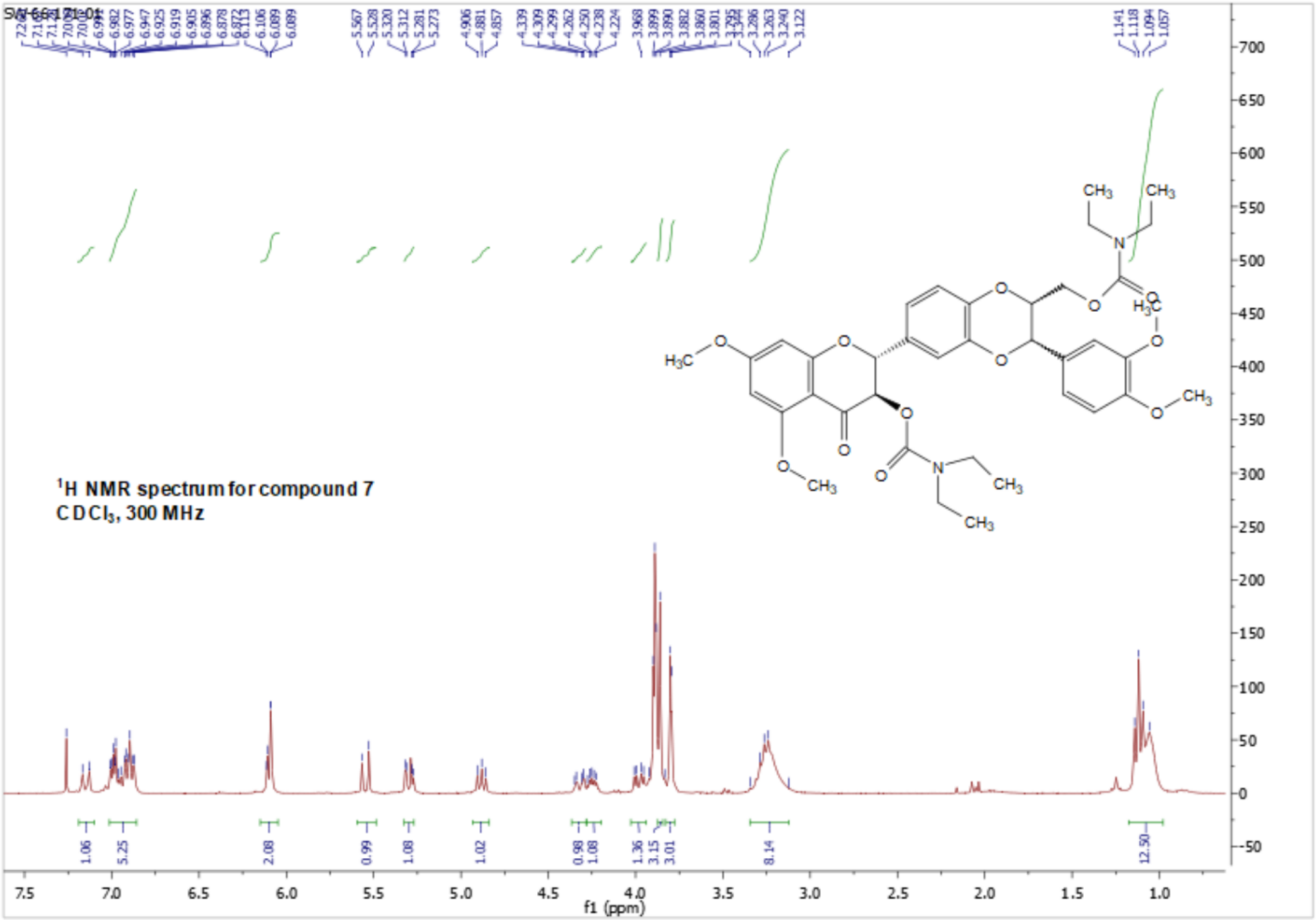

Figure S16: ^{1}H NMR spectrum of **7** in $CDCl_3$

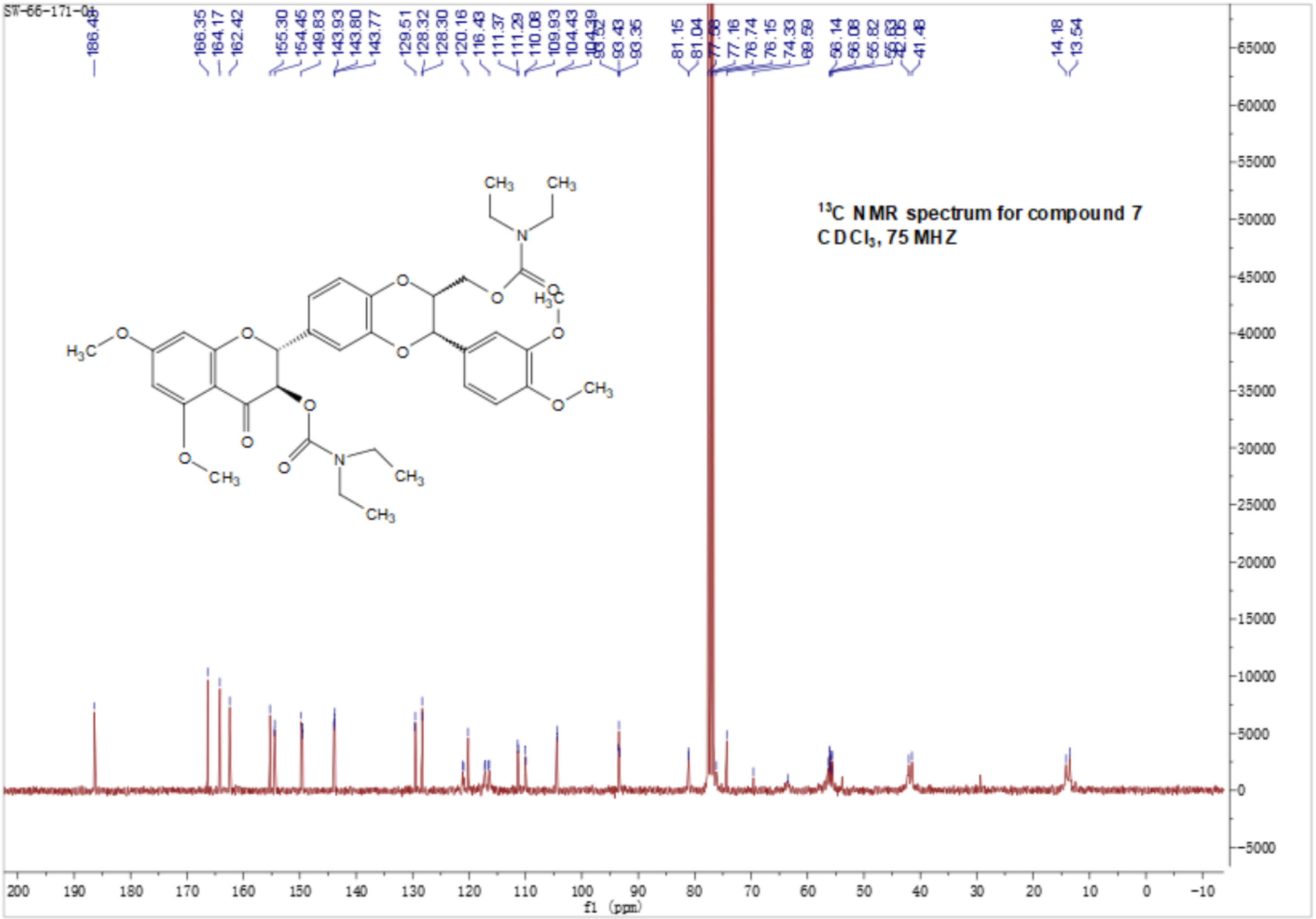

Figure S17: ^{13}C NMR spectrum of **7** in $CDCl_3$

Sample Name	Mol Fomula	MW	M+H	observed	delta	ppm
SW-66-171-01	C38H46N2O12	722.3051	723.3129	723.3129	0.0000	0.00

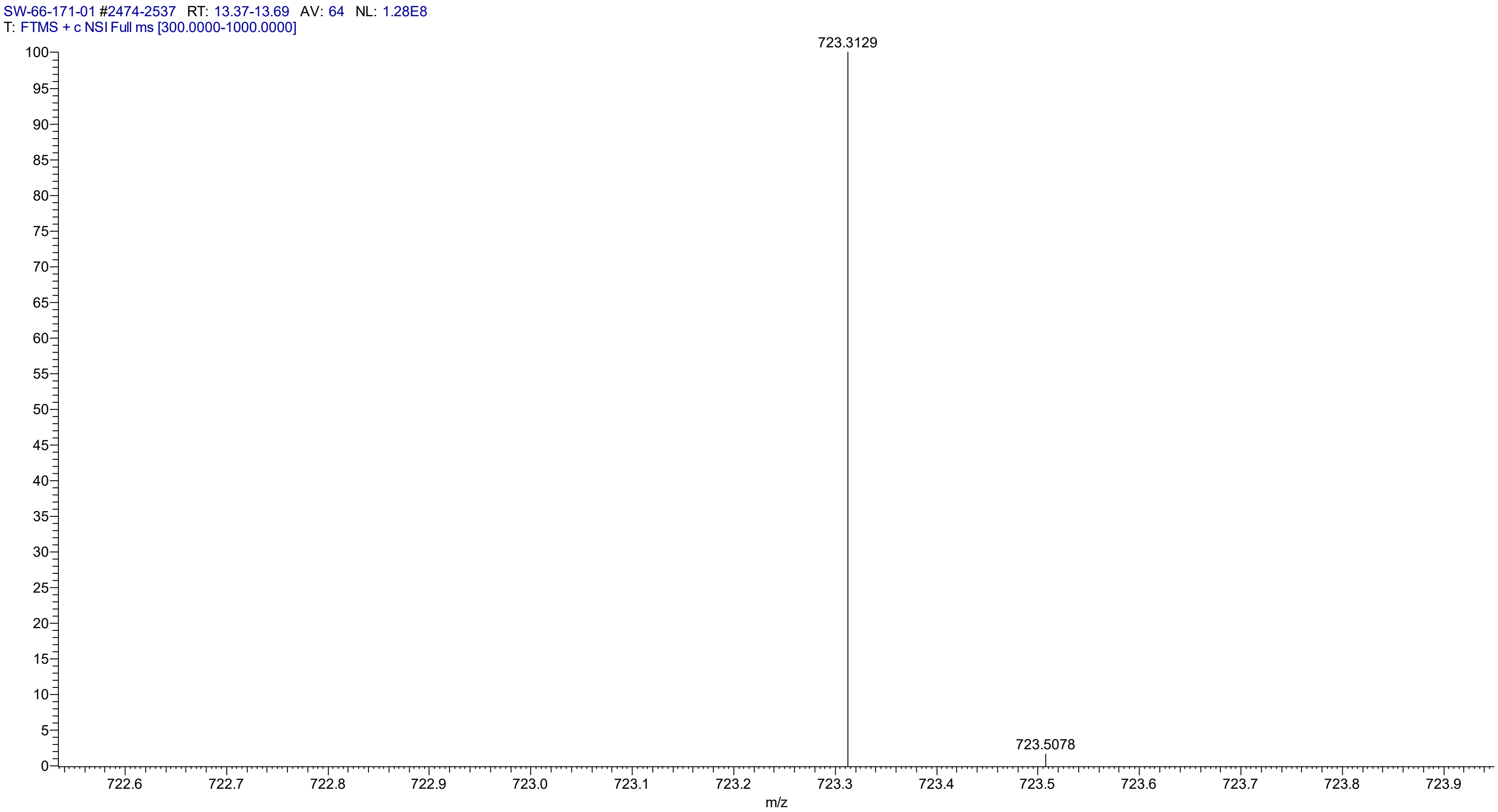

Figure S18: High resolution mass spectrum of **7**

Data File C:\HPCHEM\1\DATA\DEFAULT.D Sample Name: SW-66-171

===

Injection Date : 9/20/2021 2:44:39 PM
Sample Name : SW-66-171 Location : Vial 1
Acq. Operator :
Method : C:\HPCHEM\1\METHODS\JNP2015.M
Last changed : 9/20/2021 1:10:34 PM
(modified after loading)

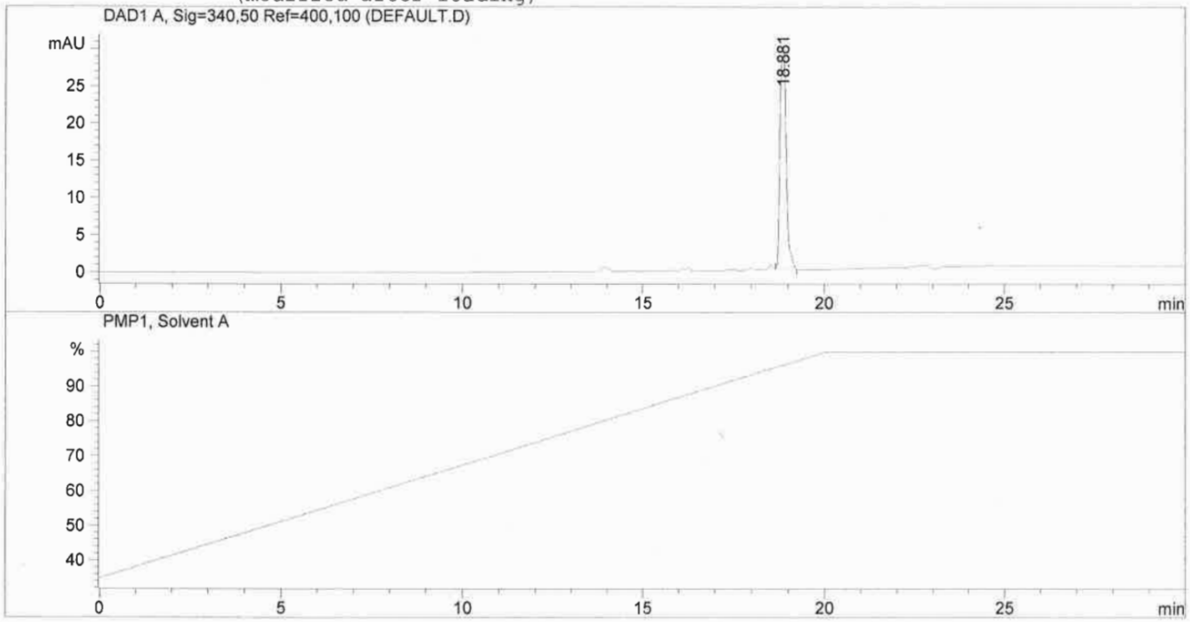

===

Area Percent Report

===

Sorted By : Signal
Multiplier : 1.0000
Dilution : 1.0000

Signal 1: DAD1 A, Sig=340,50 Ref=400,100

Peak #	RetTime [min]	Type	Width [min]	Area [mAU*s]	Height [mAU]	Area %
1	18.881	VB	0.1887	352.03210	30.04979	100.0000

Totals : 352.03210 30.04979

Results obtained with enhanced integrator!

===

*** End of Report ***

Figure S19: HPLC chromatogram of **7**

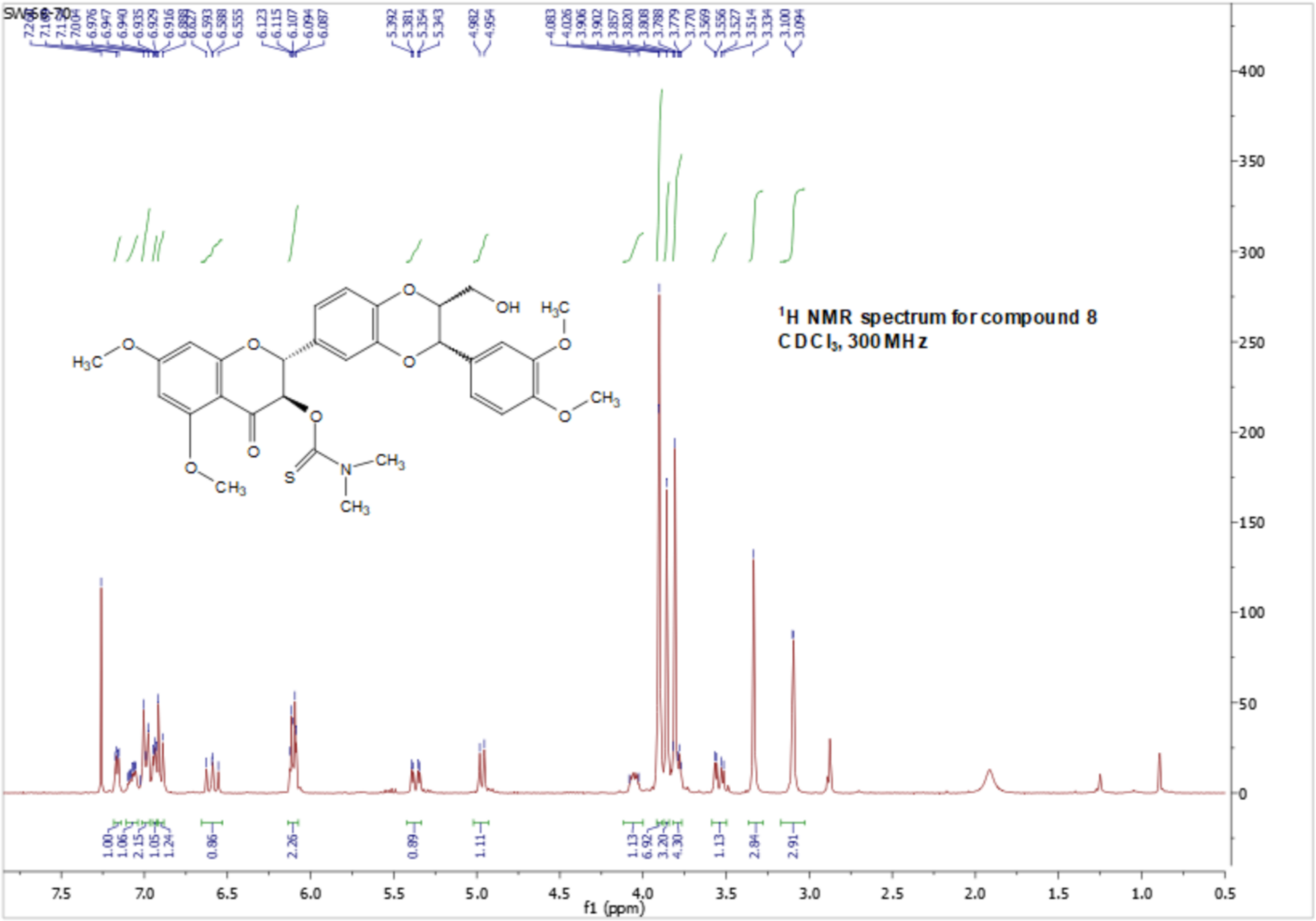

Figure S20: ^{1}H NMR spectrum of **8** in $CDCl_3$

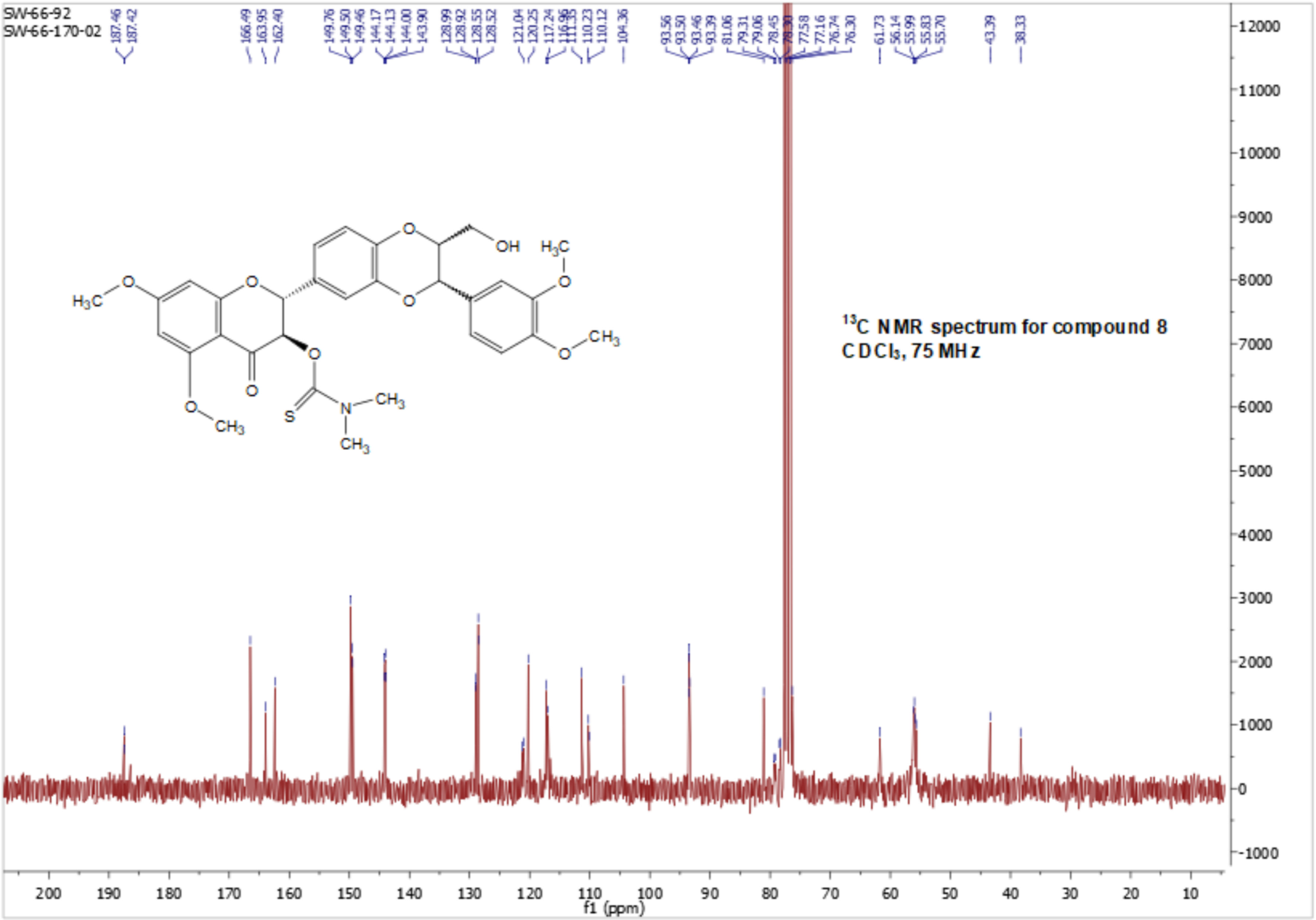

Figure S21: ^{13}C NMR spectrum of **8** in $CDCl_3$

compds	Mol. Formula	Exact Mass	M+H	**Observed**	delta	ppm
SW-66-70	C31H33NO10S	611.1826	612.1904	**612.1920**	0.0016	2.68

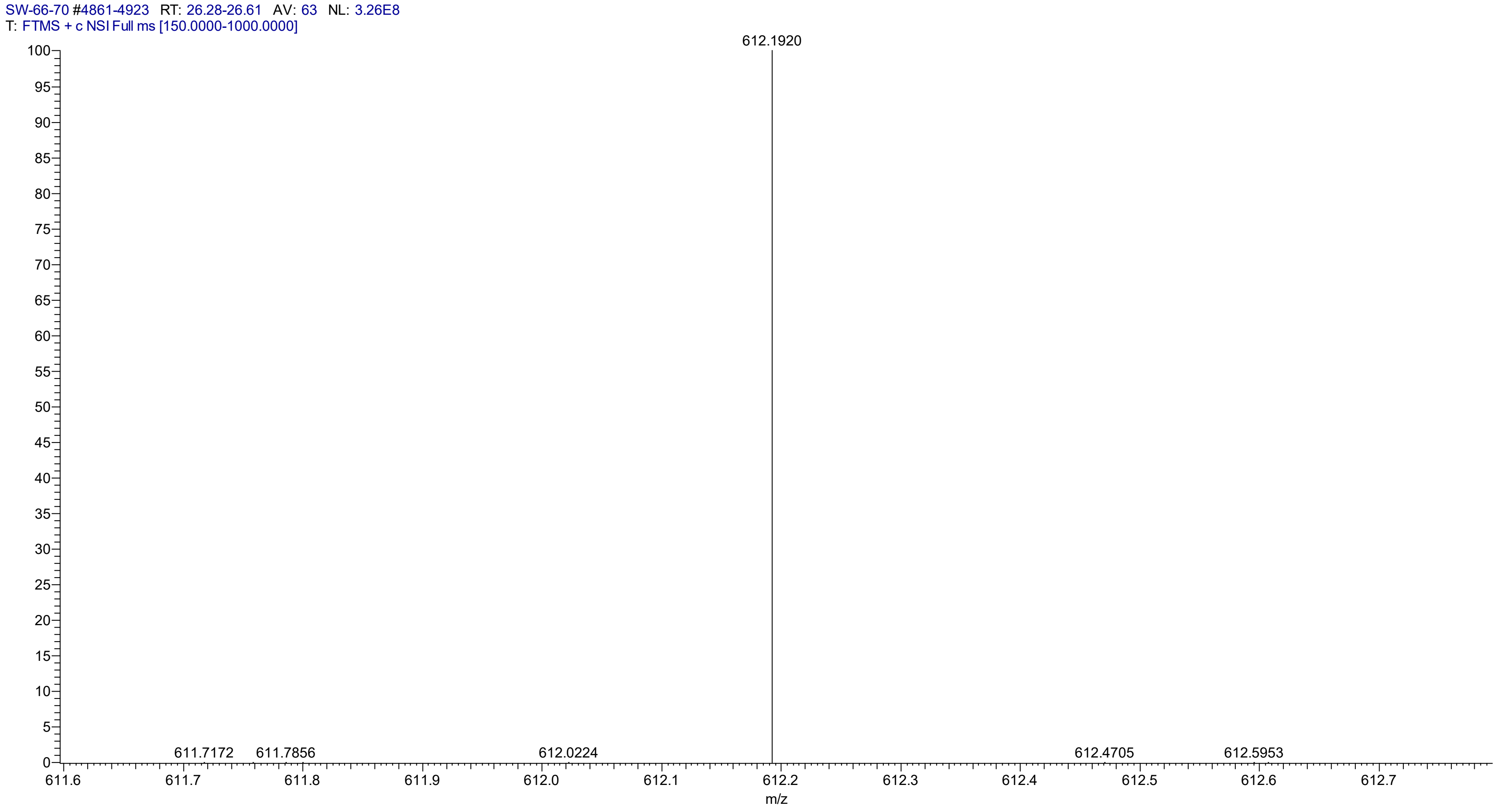

Figure S22: High resolution mass spectrum of **8**

Data File C:\HPCHEM\1\DATA\DEFAULT.D   Sample Name: SW-66-70

Injection Date : 9/17/2021 10:18:16 AM
Sample Name : SW-66-70   Location : Vial 1
Acq. Operator :
Method : C:\HPCHEM\1\METHODS\JNP2015.M
Last changed : 9/17/2021 10:10:55 AM
(modified after loading)

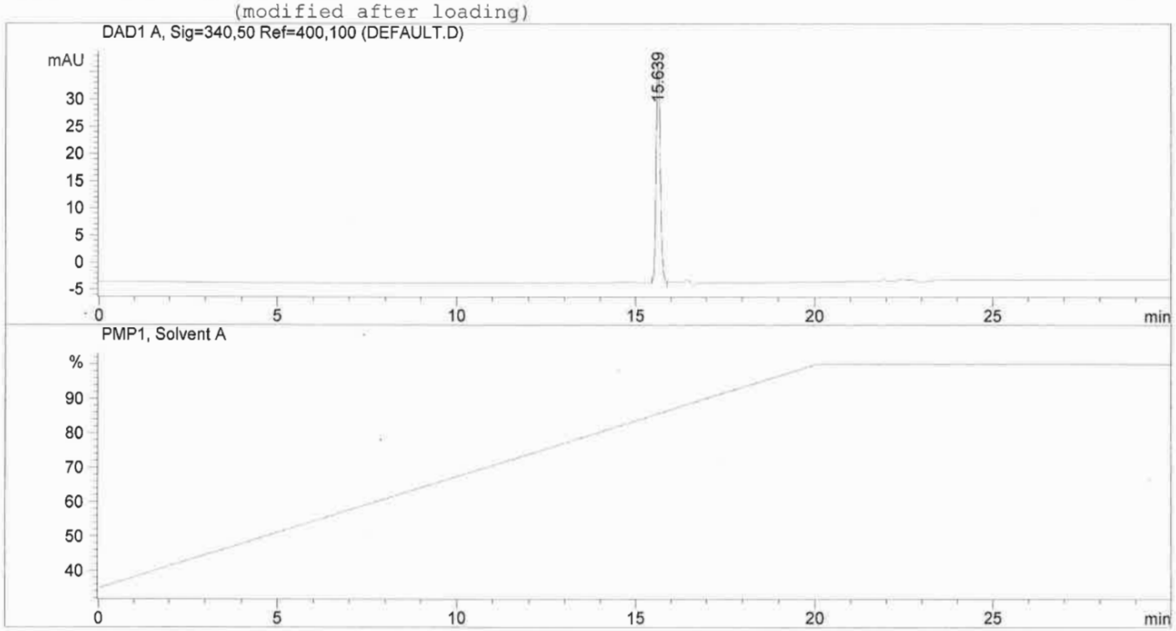

Area Percent Report

Sorted By : Signal
Multiplier : 1.0000
Dilution : 1.0000

Signal 1: DAD1 A, Sig=340,50 Ref=400,100

Peak #	RetTime [min]	Type	Width [min]	Area [mAU*s]	Height [mAU]	Area %
1	15.639	BB	0.1241	326.80460	40.52058	100.0000

Totals : 326.80460 40.52058

Results obtained with enhanced integrator!

*** End of Report ***

Figure S23: HPLC chromatogram of **8**

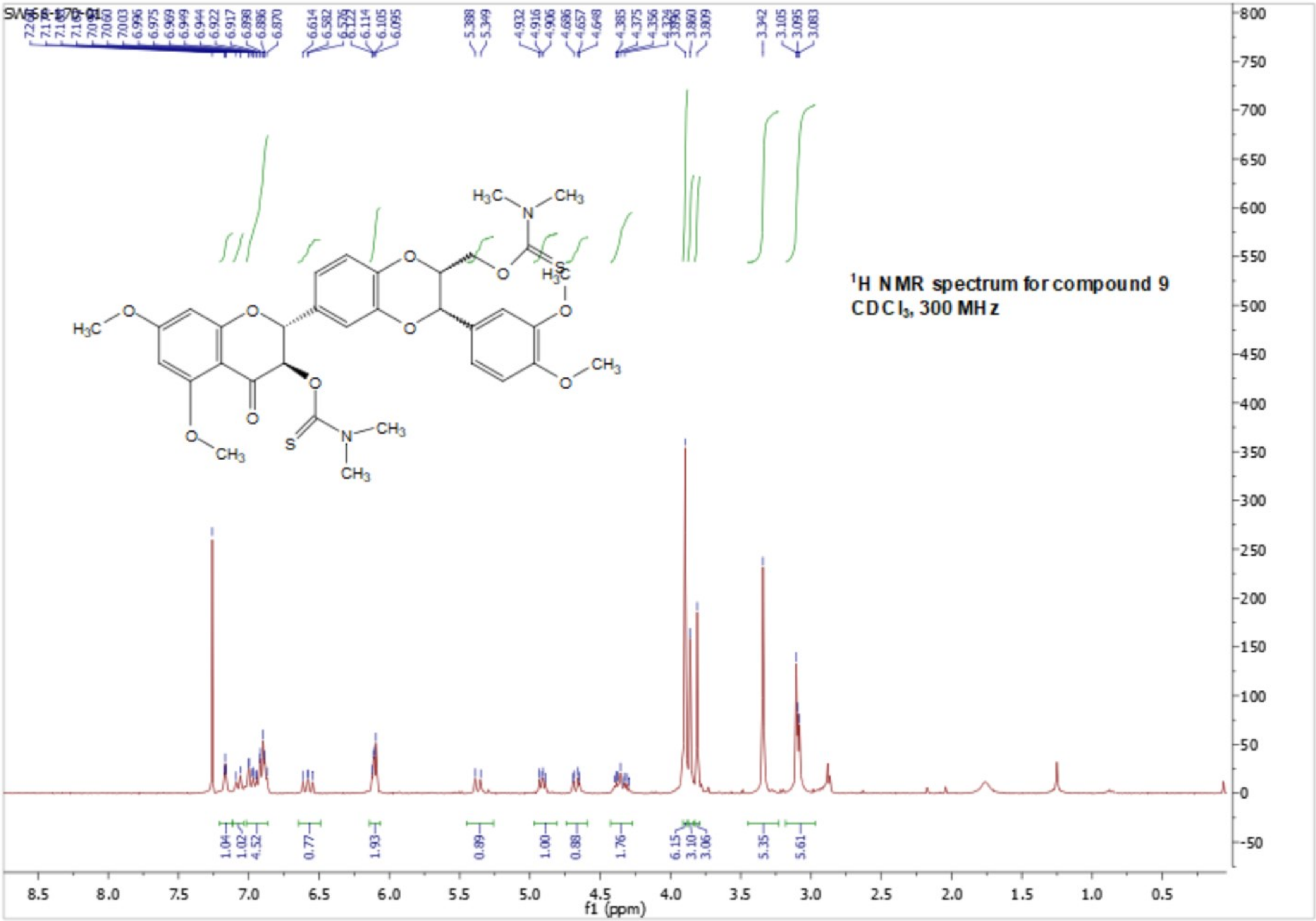

Figure S24: ^{1}H NMR spectrum of **9** in $CDCl_3$

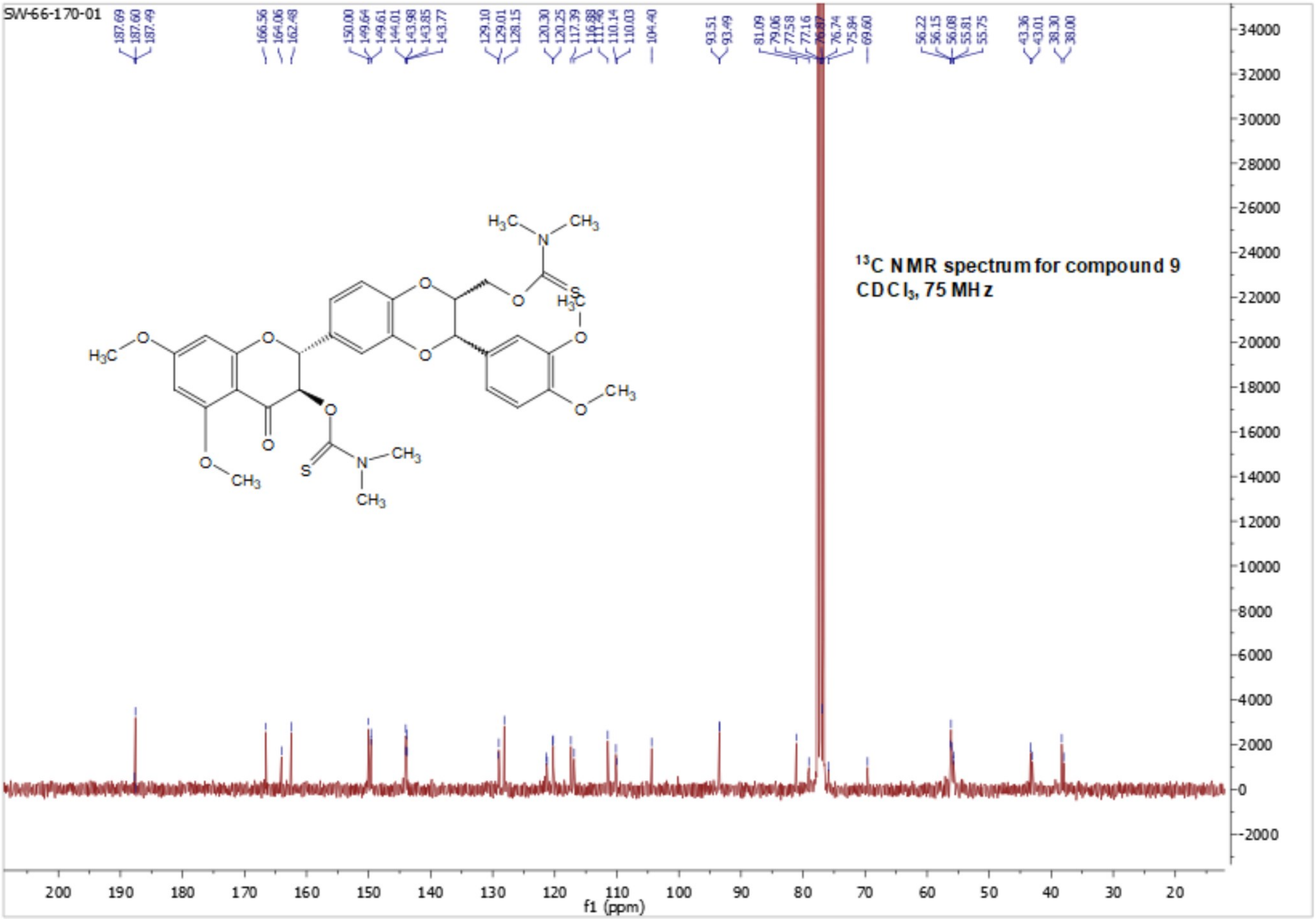

Figure S25: ^{13}C NMR spectrum of **9** in $CDCl_3$

Sample Name	Mol Fomula	MW	M+H	observed	delta	ppm
SW-66-170-01	C34H38N2O10S2	698.1968	699.2046	699.2050	0.0004	0.51

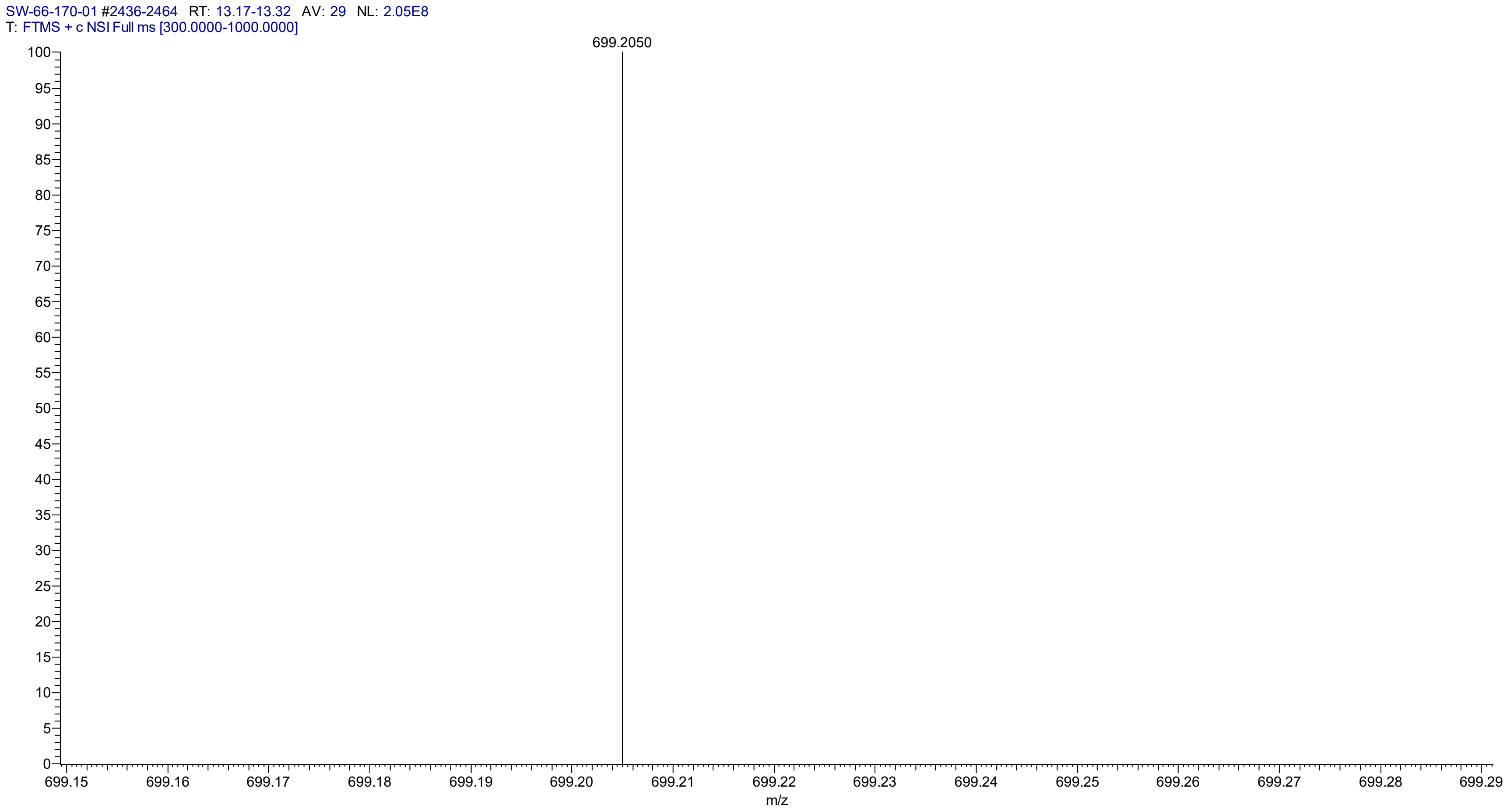

Figure S26: High resolution mass spectrum of **9**

Data File C:\HPCHEM\1\DATA\DEFAULT.D Sample Name: SW-66-170

===

Injection Date : 9/18/2021 11:40:15 AM
Sample Name : SW-66-170 Location : Vial 1
Acq. Operator :
Method : C:\HPCHEM\1\METHODS\JNP2015.M
Last changed : 9/18/2021 9:26:55 AM
(modified after loading)

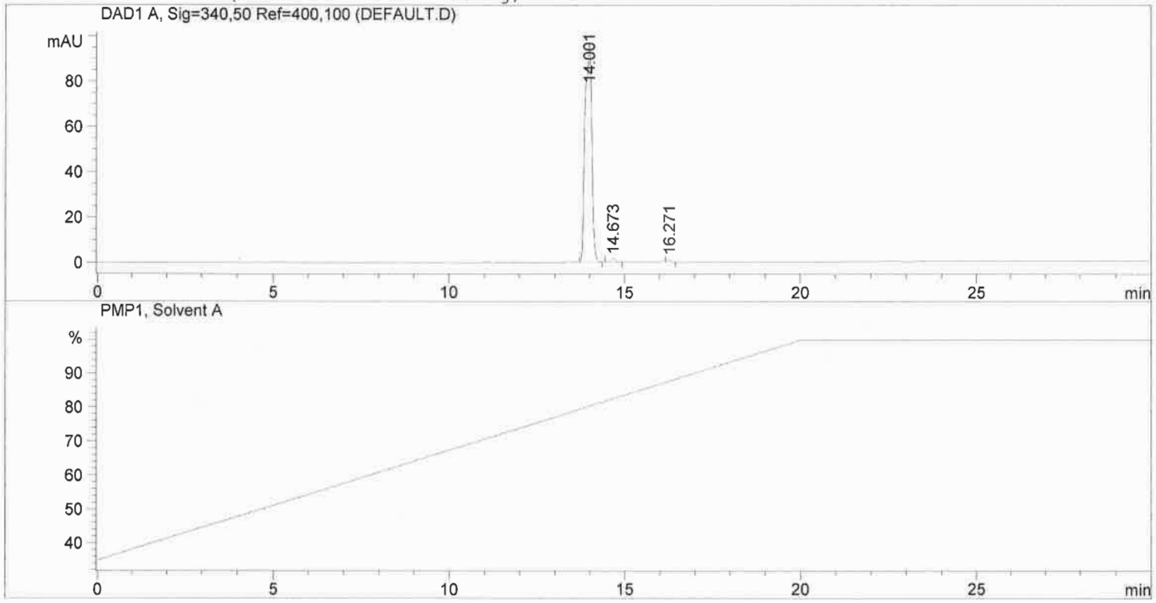

===

Area Percent Report

===

Sorted By : Signal
Multiplier : 1.0000
Dilution : 1.0000

Signal 1: DAD1 A, Sig=340,50 Ref=400,100

Peak #	RetTime [min]	Type	Width [min]	Area [mAU*s]	Height [mAU]	Area %
1	14.001	BB	0.1829	1281.49658	96.52377	97.8661
2	14.673	BP	0.1679	19.23740	1.67563	1.4691
3	16.271	VP	0.1173	8.70405	1.13770	0.6647
Totals :				1309.43803	99.33710	

Results obtained with enhanced integrator!

===

*** End of Report ***

Figure S27: HPLC chromatogram of **9**

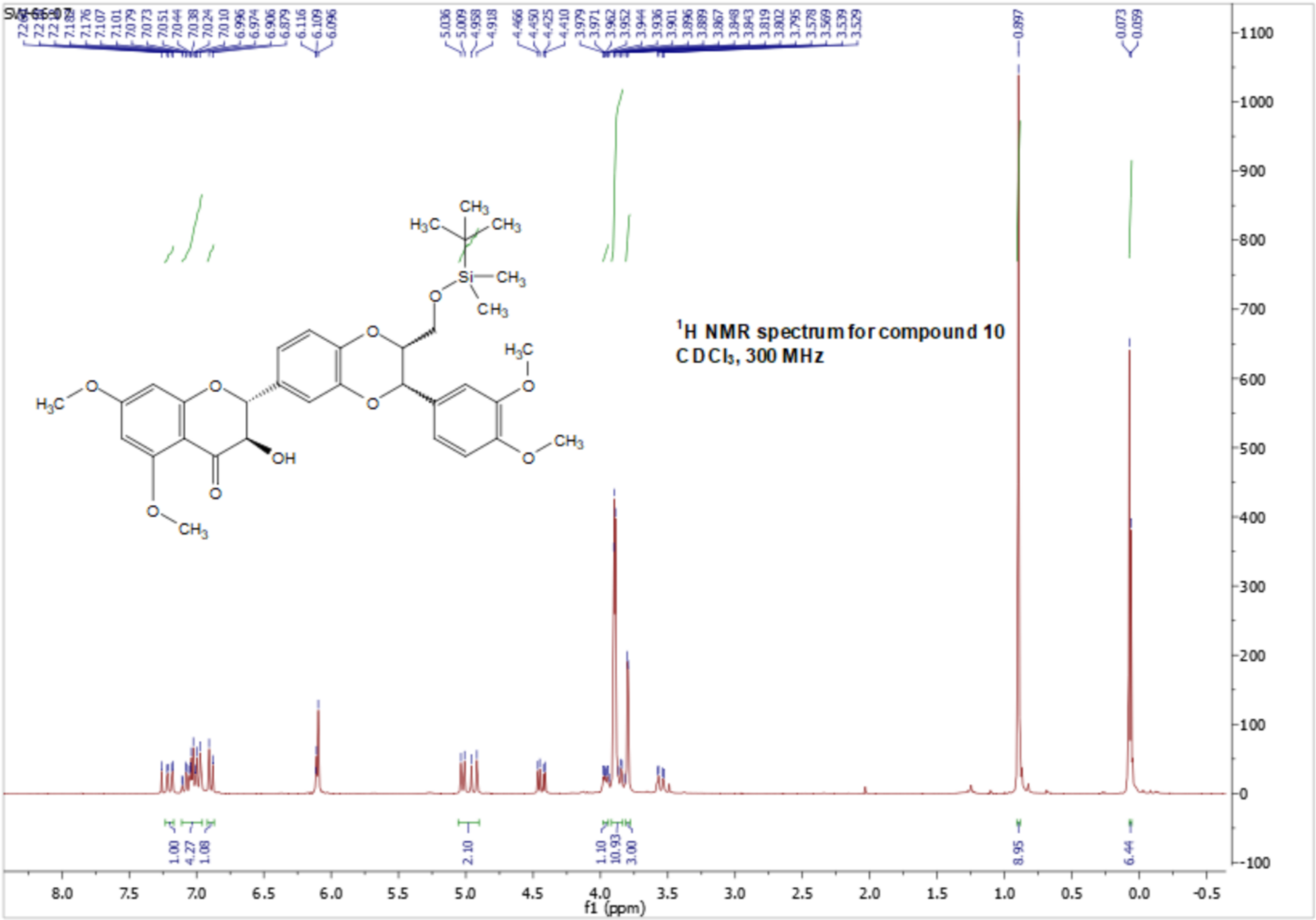

Figure S28: ^{1}H NMR spectrum of **10** in $CDCl_3$

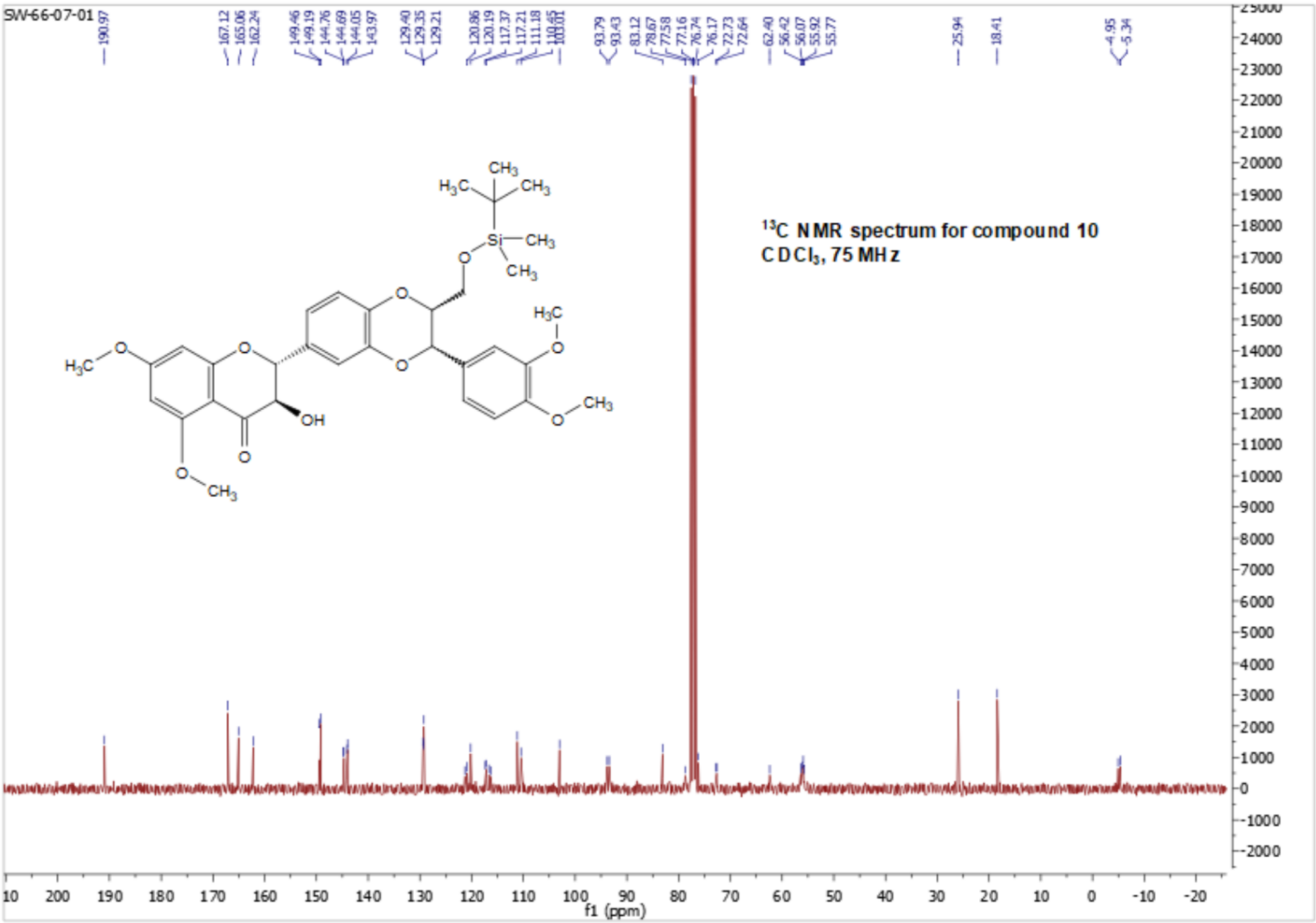

Figure S29: ^{13}C NMR spectrum of **10** in $CDCl_3$

compds	Mol. Formula	Exact Mass	M+H	**Observed**	**delta**	**ppm**
SW-66-07	C24H42O10Si	638.2548	639.2626	**639.2623**	**-0.0003**	**-0.44**

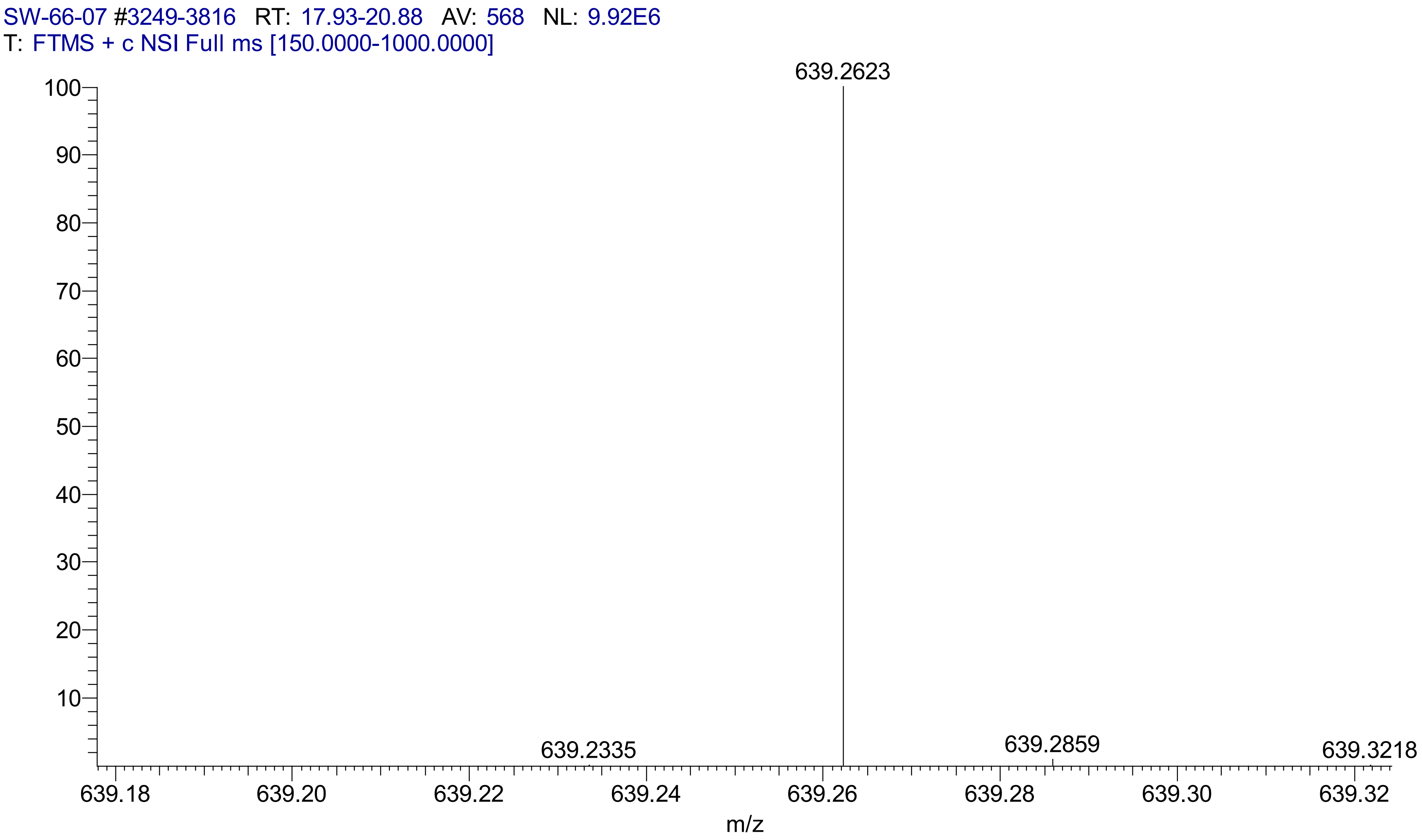

Figure S30: High resolution mass spectrum of **10**

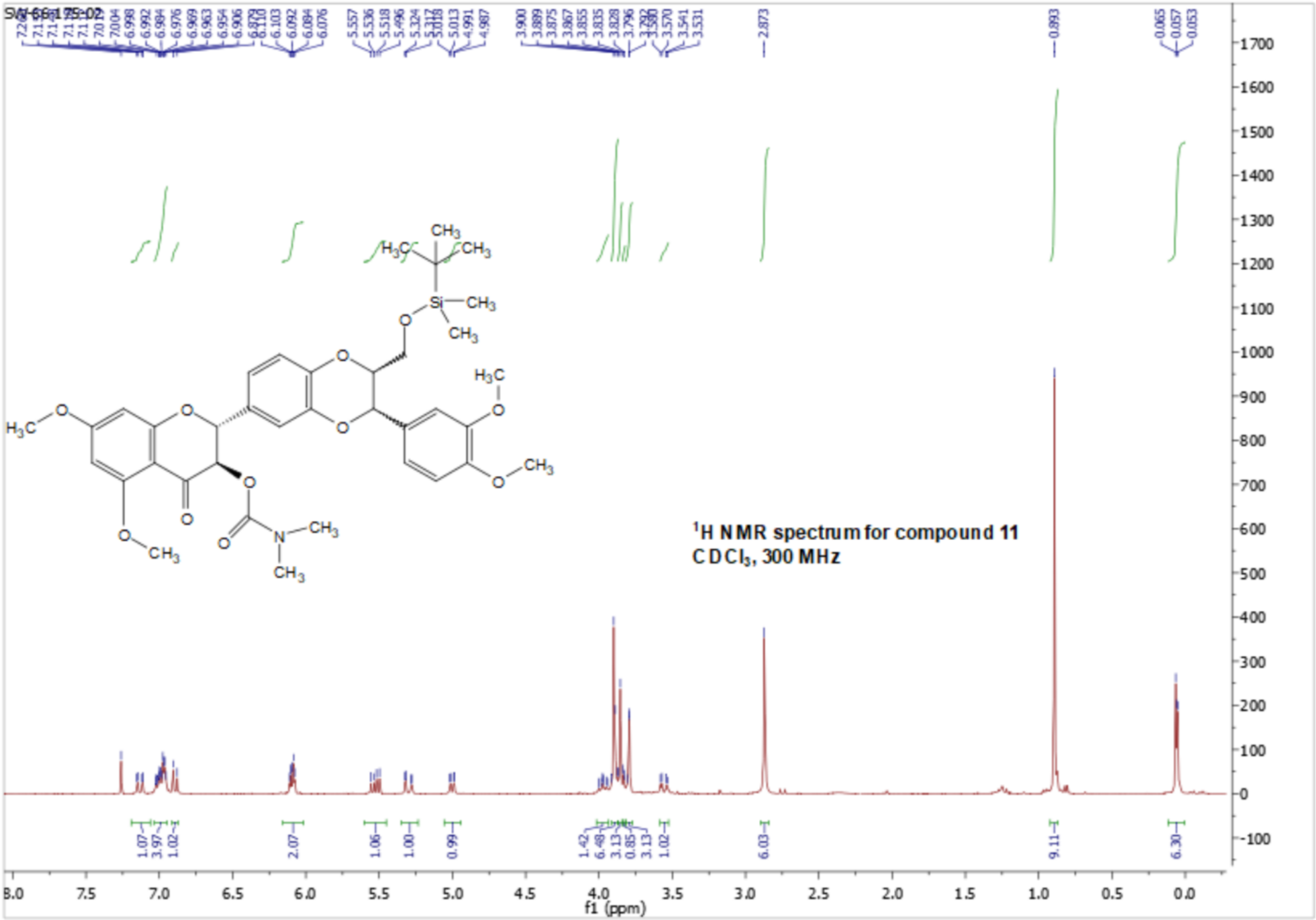

Figure S31: ^{1}H NMR spectrum of **11** in $CDCl_3$

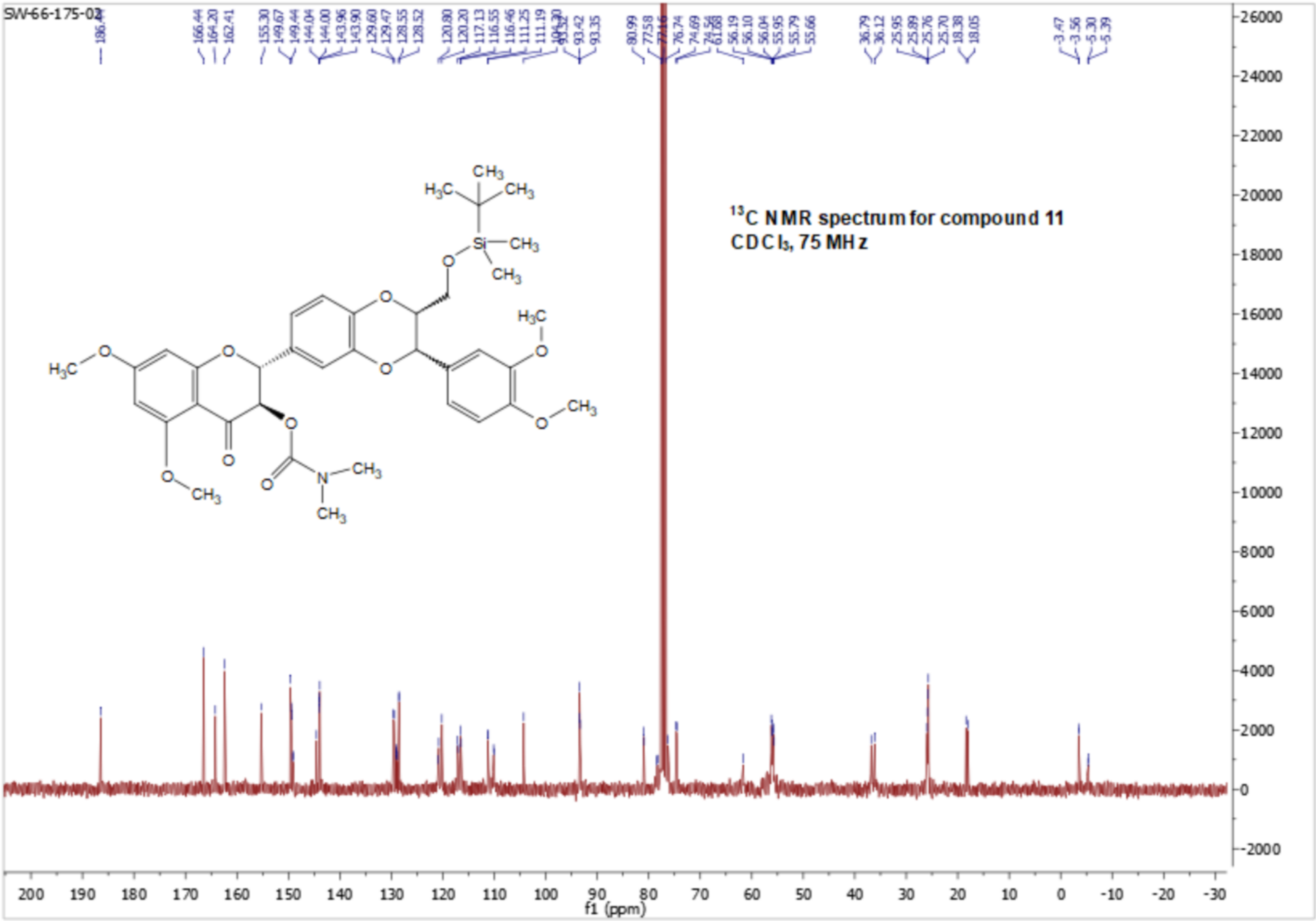

Figure S32: ^{13}C NMR spectrum of **11** in $CDCl_3$

Sample Name	Mol Fomula	MW	M+H	observed	delta	ppm
SW-66-175-02	C37H47NO11Si	709.2919	710.2997	710.3006	0.0009	1.27

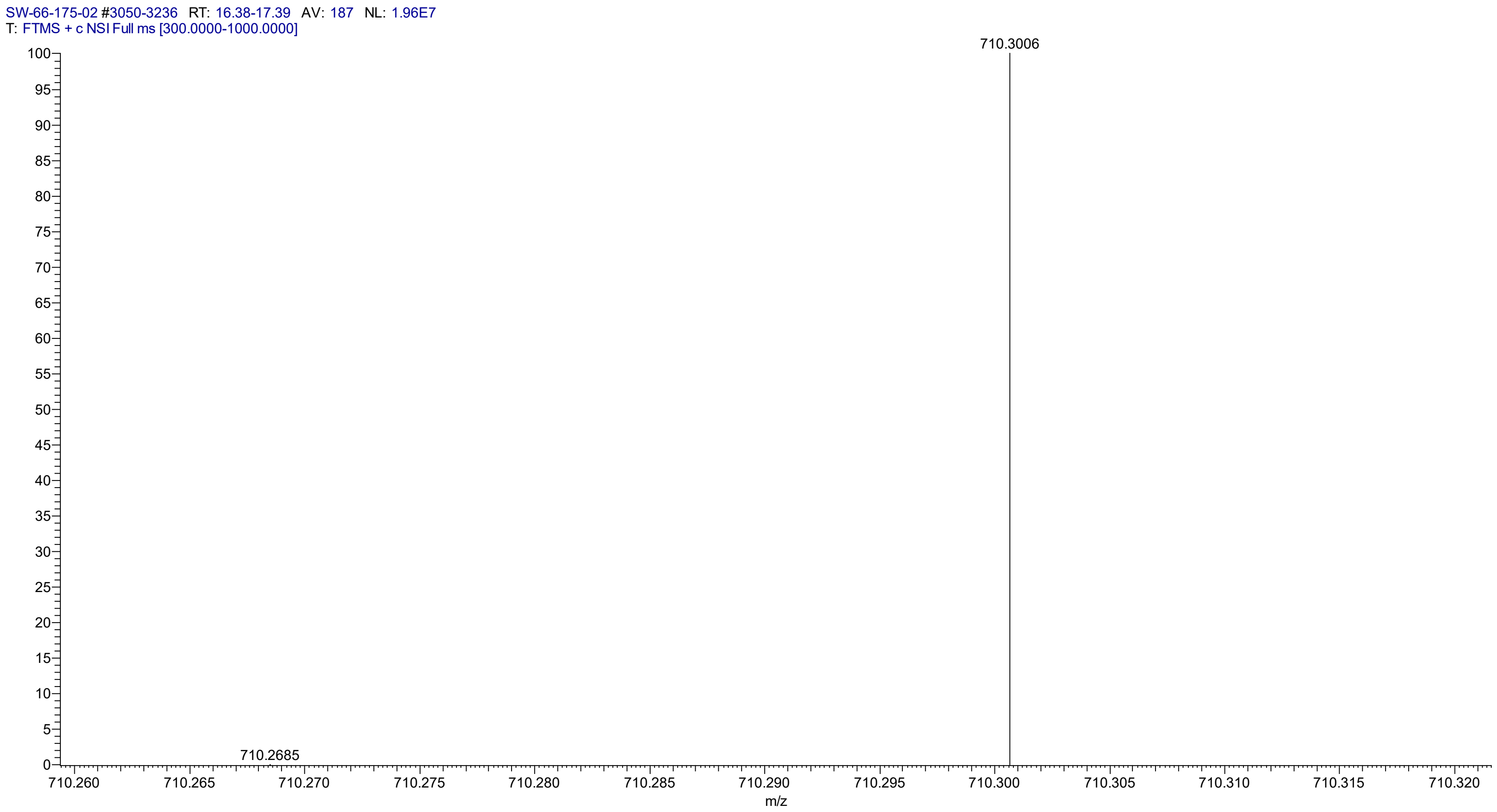

Figure S33: High resolution mass spectrum of **11**

Data File C:\HPCHEM\1\DATA\DEFAULT.D　　　　Sample Name: SW-66-175-02

Injection Date : 9/17/2021 11:10:47 AM
Sample Name : SW-66-175-02　　　　Location : Vial 1
Acq. Operator :
Method : C:\HPCHEM\1\METHODS\JNP2015.M
Last changed : 9/17/2021 10:10:55 AM
(modified after loading)

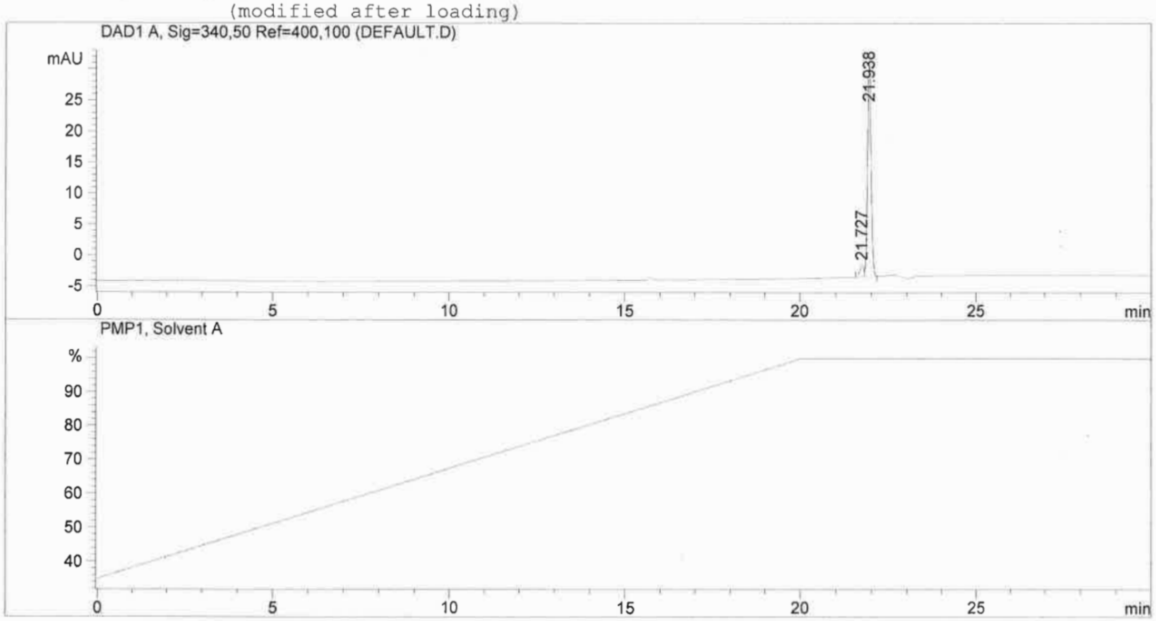

Area Percent Report

Sorted By : Signal
Multiplier : 1.0000
Dilution : 1.0000

Signal 1: DAD1 A, Sig=340,50 Ref=400,100

Peak #	RetTime [min]	Type	Width [min]	Area [mAU*s]	Height [mAU]	Area %
1	21.727	BV	0.1121	14.86666	1.96797	5.8414
2	21.938	VB	0.1067	239.63838	34.64161	94.1586

Totals : 254.50505 36.60958

Results obtained with enhanced integrator!

*** End of Report ***

Figure S33: HPLC chromatogram of **11**

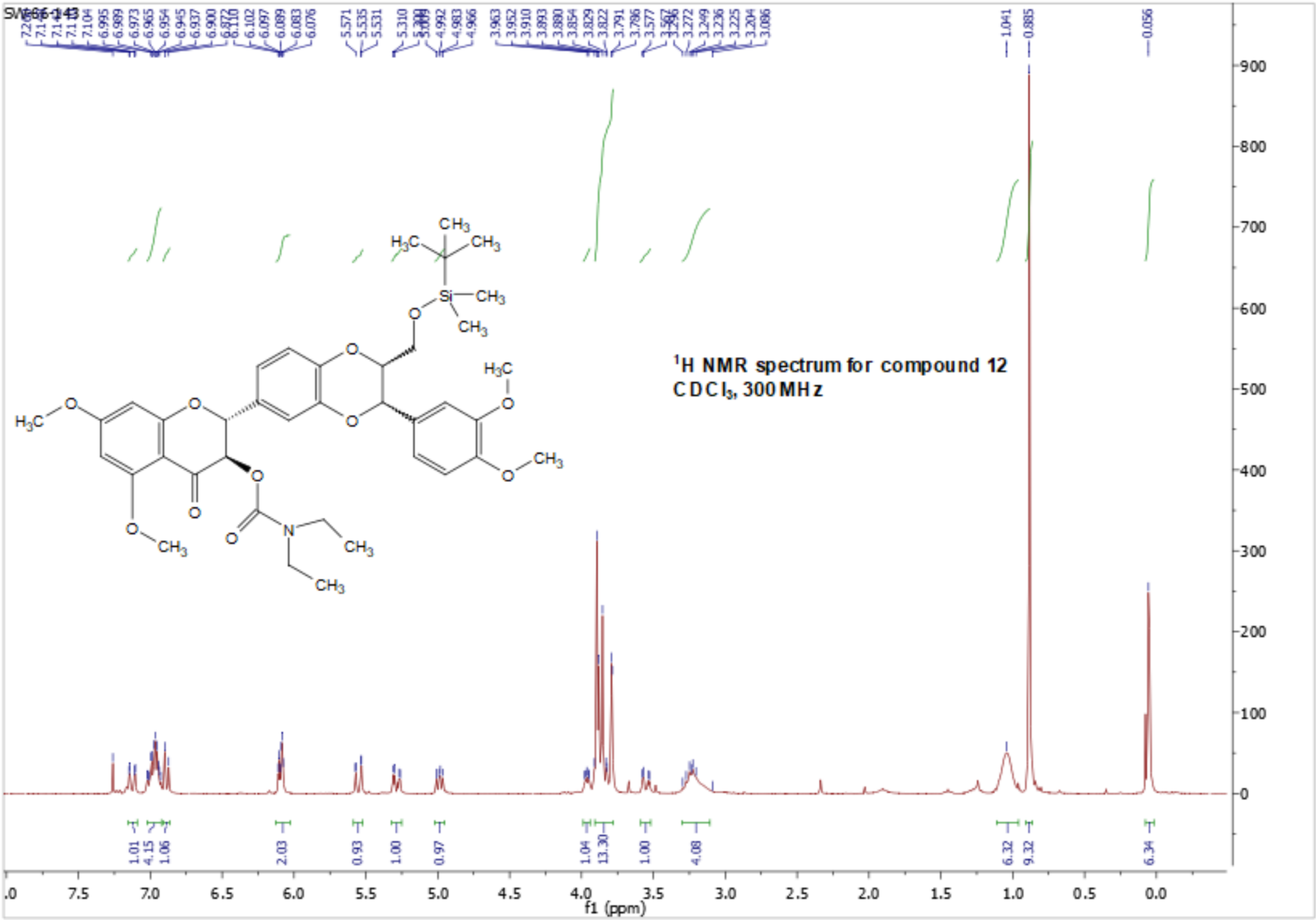

Figure S34: ^{1}H NMR spectrum of **12** in $CDCl_3$

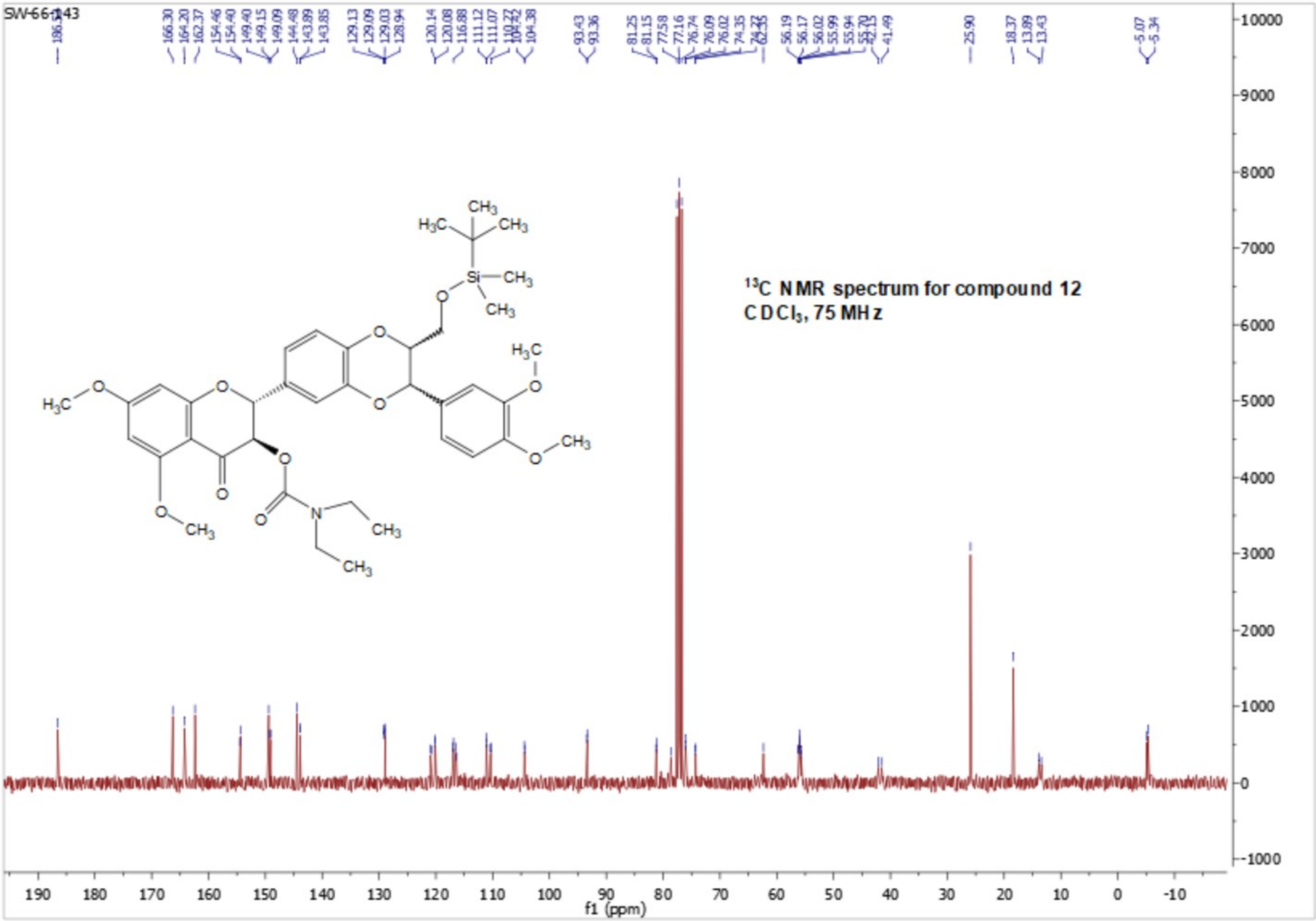

Figure S35: ^{13}C NMR spectrum of **12** in $CDCl_3$

compds	Mol. Formula	Exact Mass	M+H	**Observed**	delta	ppm
SW-66-143	C39H51NO11Si	737.3232	738.3310	**738.3329**	0.0019	2.57

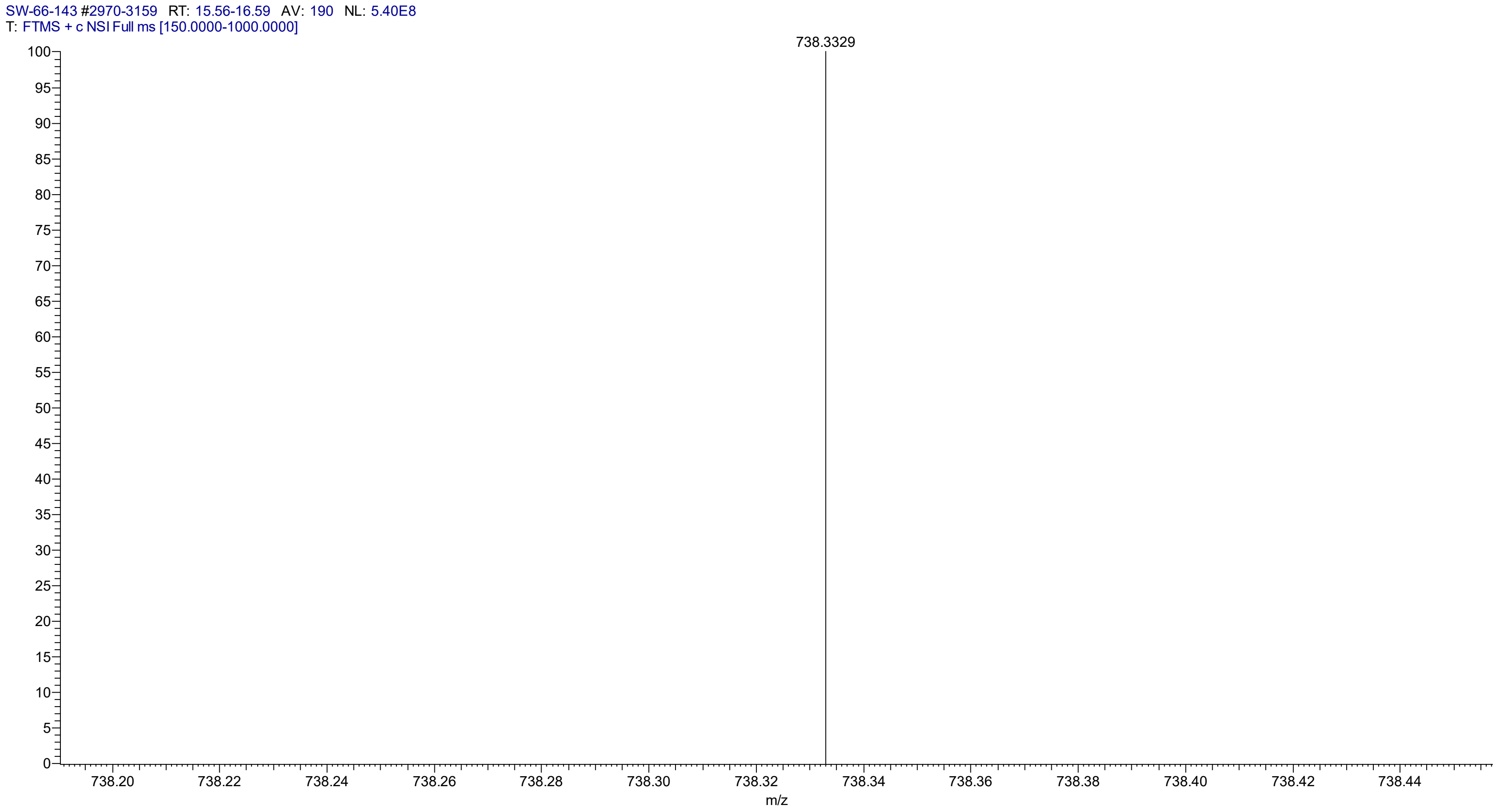

Figure S36: High resolution mass spectrum of **12**

Data File C:\HPCHEM\1\DATA\DEFAULT.D

Sample Name: SW-66-95

Injection Date : 9/13/2021 2:48:00 PM
Sample Name : SW-66-95 Location : Vial 1
Acq. Operator :
Method : C:\HPCHEM\1\METHODS\JNP2015.M
Last changed : 9/13/2021 2:42:55 PM
(modified after loading)

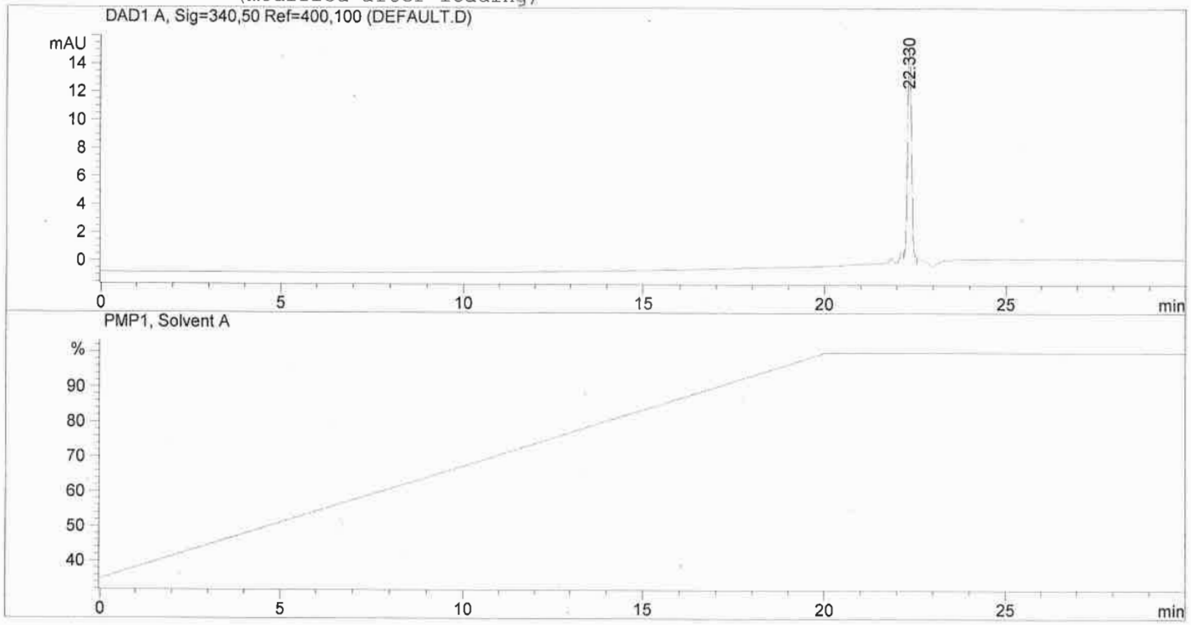

Area Percent Report

Sorted By : Signal
Multiplier : 1.0000
Dilution : 1.0000

Signal 1: DAD1 A, Sig=340,50 Ref=400,100

Peak #	RetTime [min]	Type	Width [min]	Area [mAU*s]	Height [mAU]	Area %
1	22.330	VB	0.1206	115.30704	15.17783	100.0000

Totals : 115.30704 15.17783

Results obtained with enhanced integrator!

*** End of Report ***

Figure S37: HPLC chromatogram of **12**

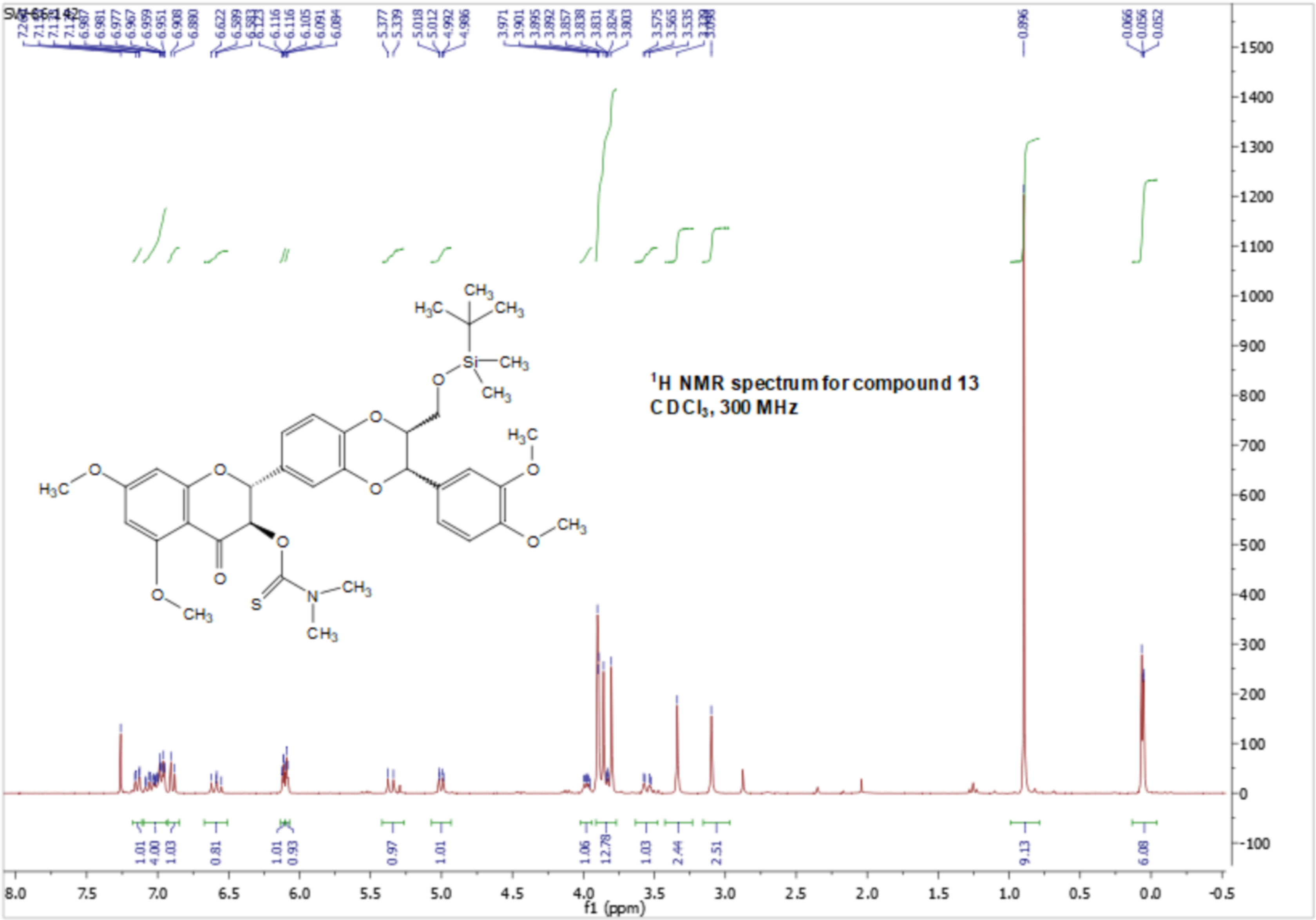

Figure S38: ^{1}H NMR spectrum of **13** in $CDCl_3$

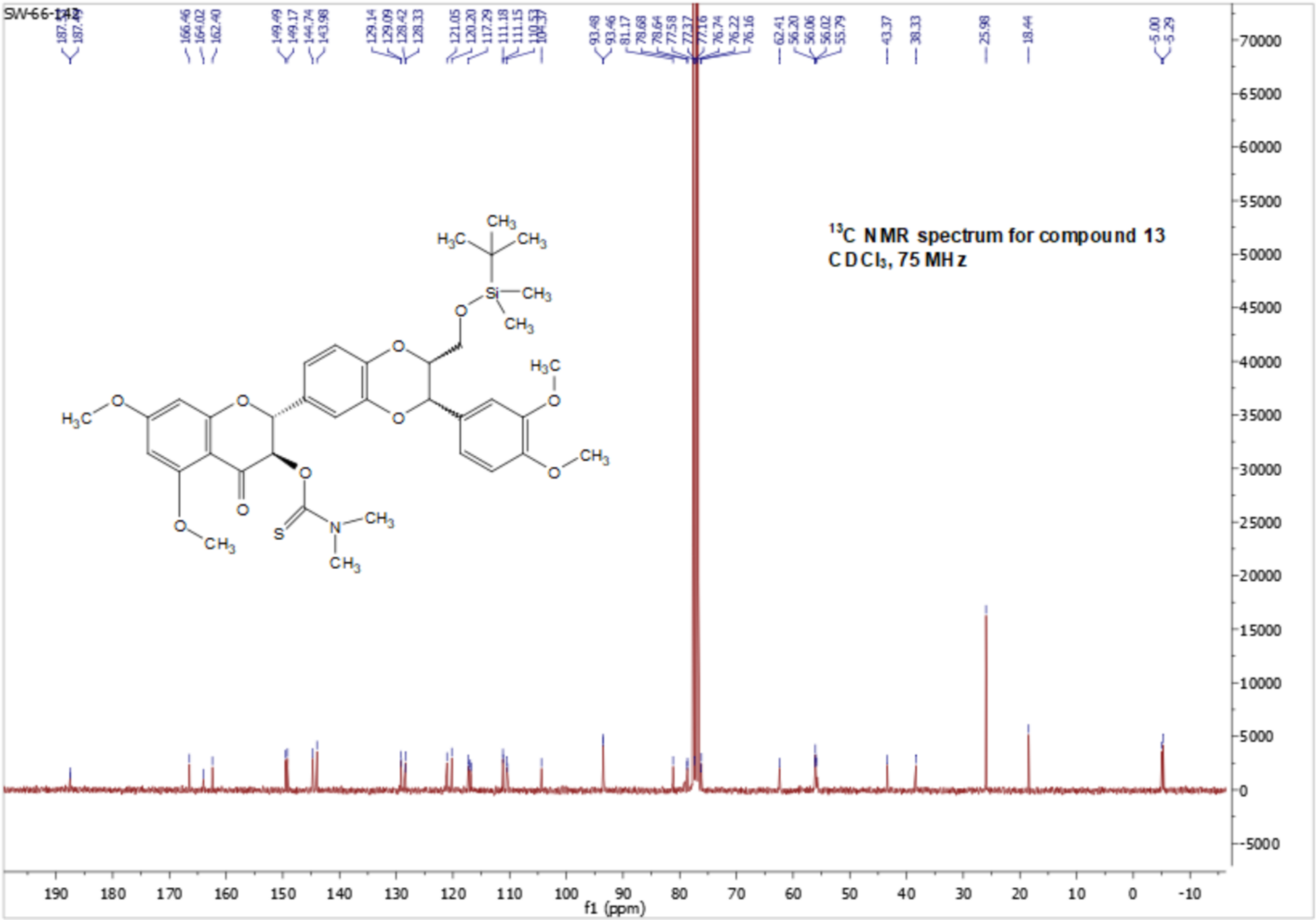

Figure S39: ^{13}C NMR spectrum of **13** in $CDCl_3$

compds	Mol. Formula	Exact Mass	M+H	**Observed**	delta	ppm
SW-66-142	C37H47NO10SSi	725.2691	726.2769	**726.2796**	0.0027	3.79

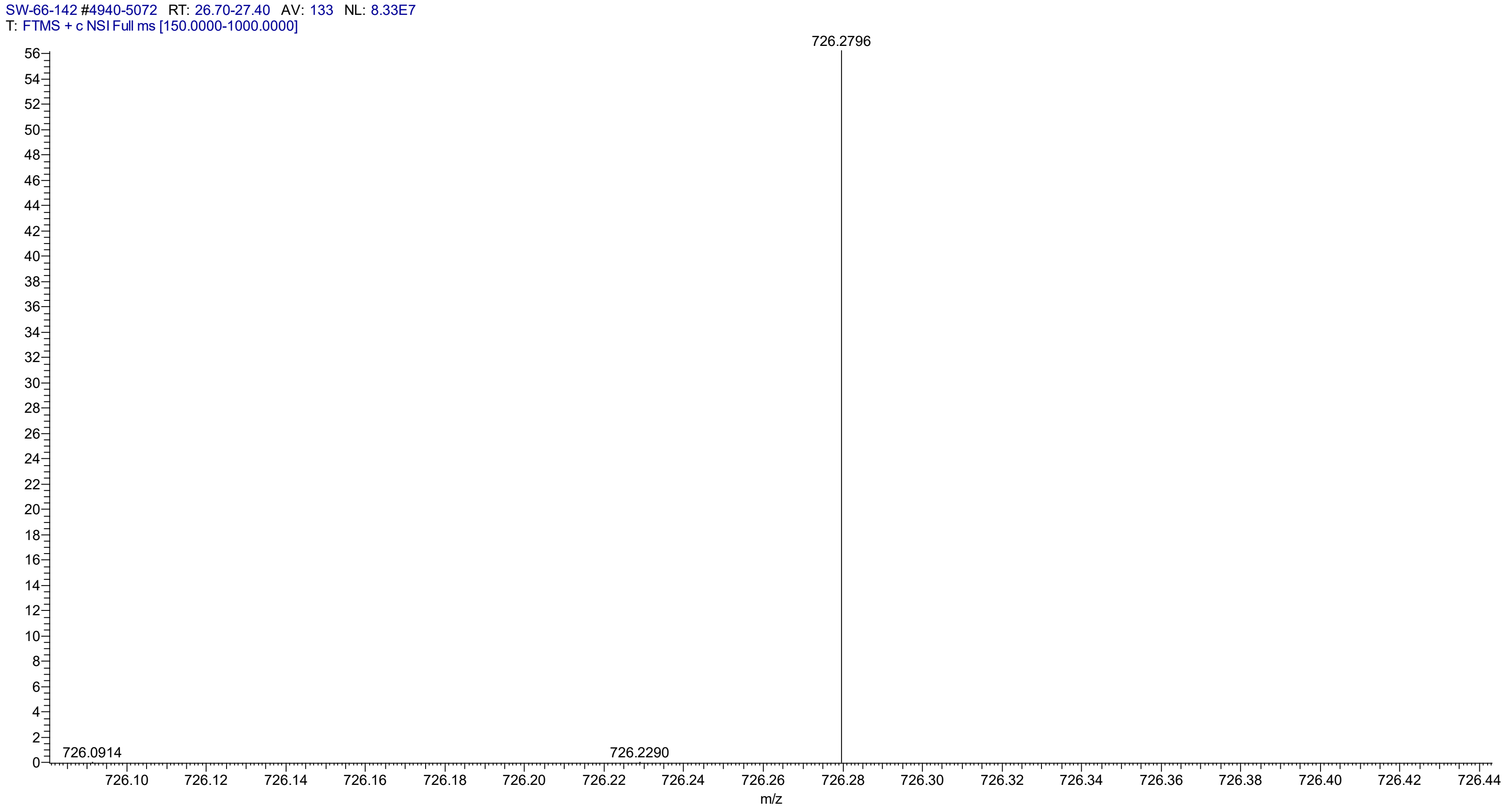

Figure S40: High resolution mass spectrum of **13**

Data File C:\HPCHEM\1\DATA\DEFAULT.D Sample Name: SW-66-94

```
=====================================================================
Injection Date  : 9/13/2021 11:55:21 AM
Sample Name     : SW-66-94                      Location : Vial 1
Acq. Operator   :
Method          : C:\HPCHEM\1\METHODS\JNP2015.M
Last changed    : 9/13/2021 11:53:45 AM
                  (modified after loading)
```

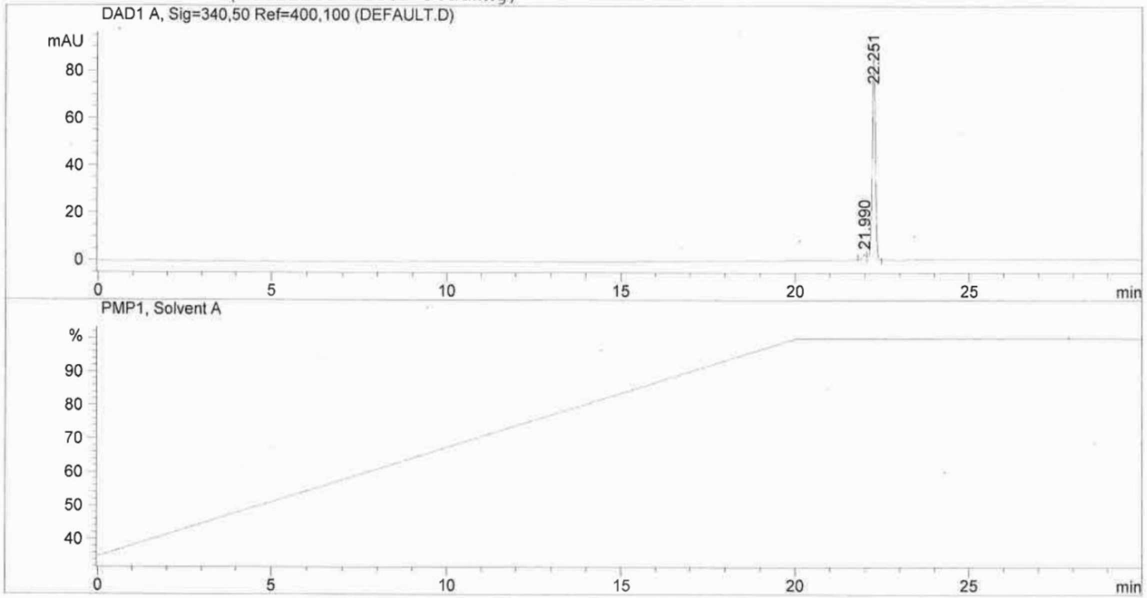


```
=====================================================================
                      Area Percent Report
=====================================================================

Sorted By             :      Signal
Multiplier            :      1.0000
Dilution              :      1.0000

Signal 1: DAD1 A, Sig=340,50 Ref=400,100
```

Peak #	RetTime [min]	Type	Width [min]	Area [mAU*s]	Height [mAU]	Area %
1	21.990	BV	0.1080	19.44175	2.57780	3.3204
2	22.251	VB	0.0966	566.07684	91.02409	96.6796
Totals :				585.51859	93.60189	

```
Results obtained with enhanced integrator!
=====================================================================
                      *** End of Report ***
```

Figure S41: HPLC chromatogram of **13**

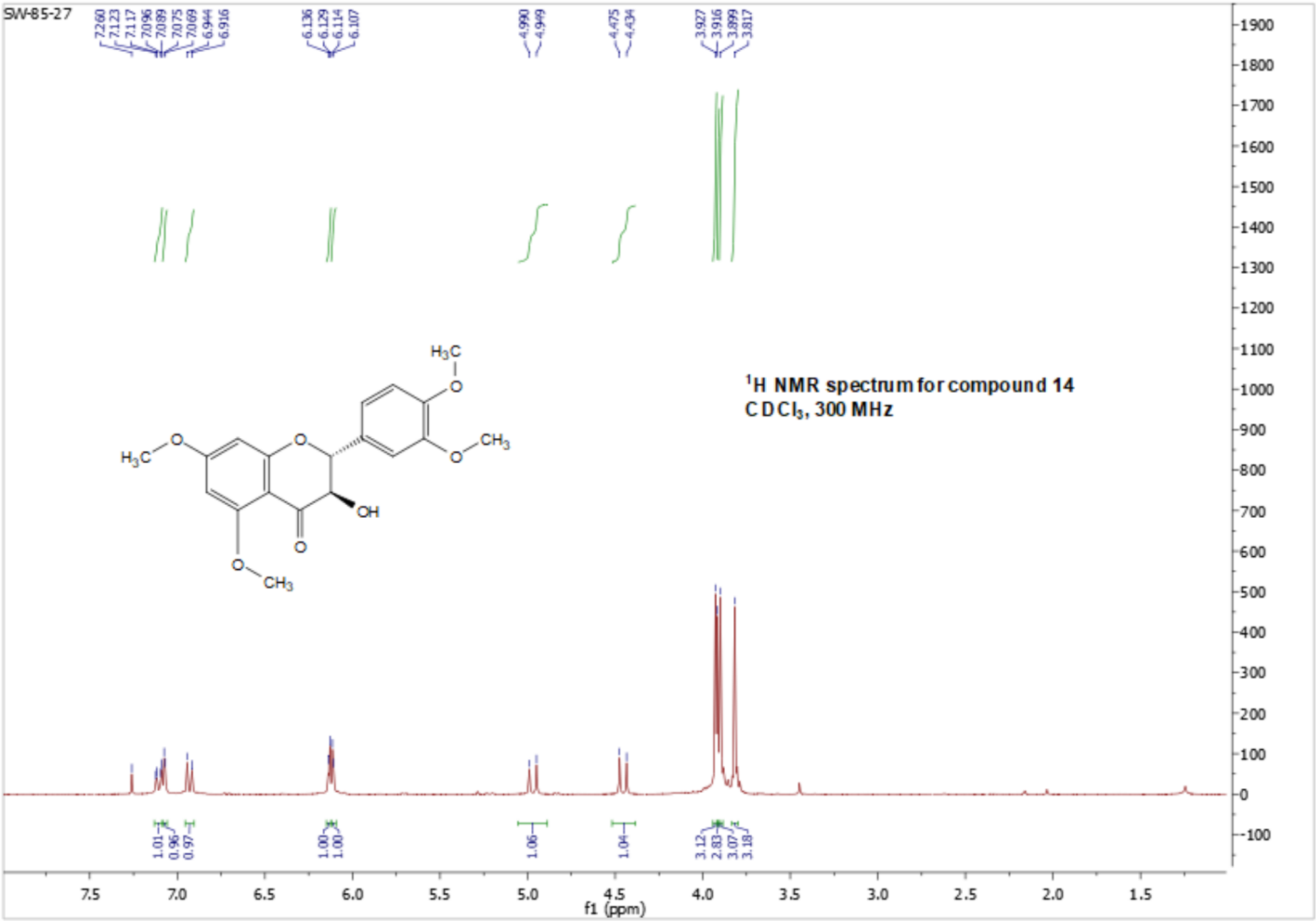

Figure S42: ^{1}H NMR spectrum of **14** in $CDCl_3$

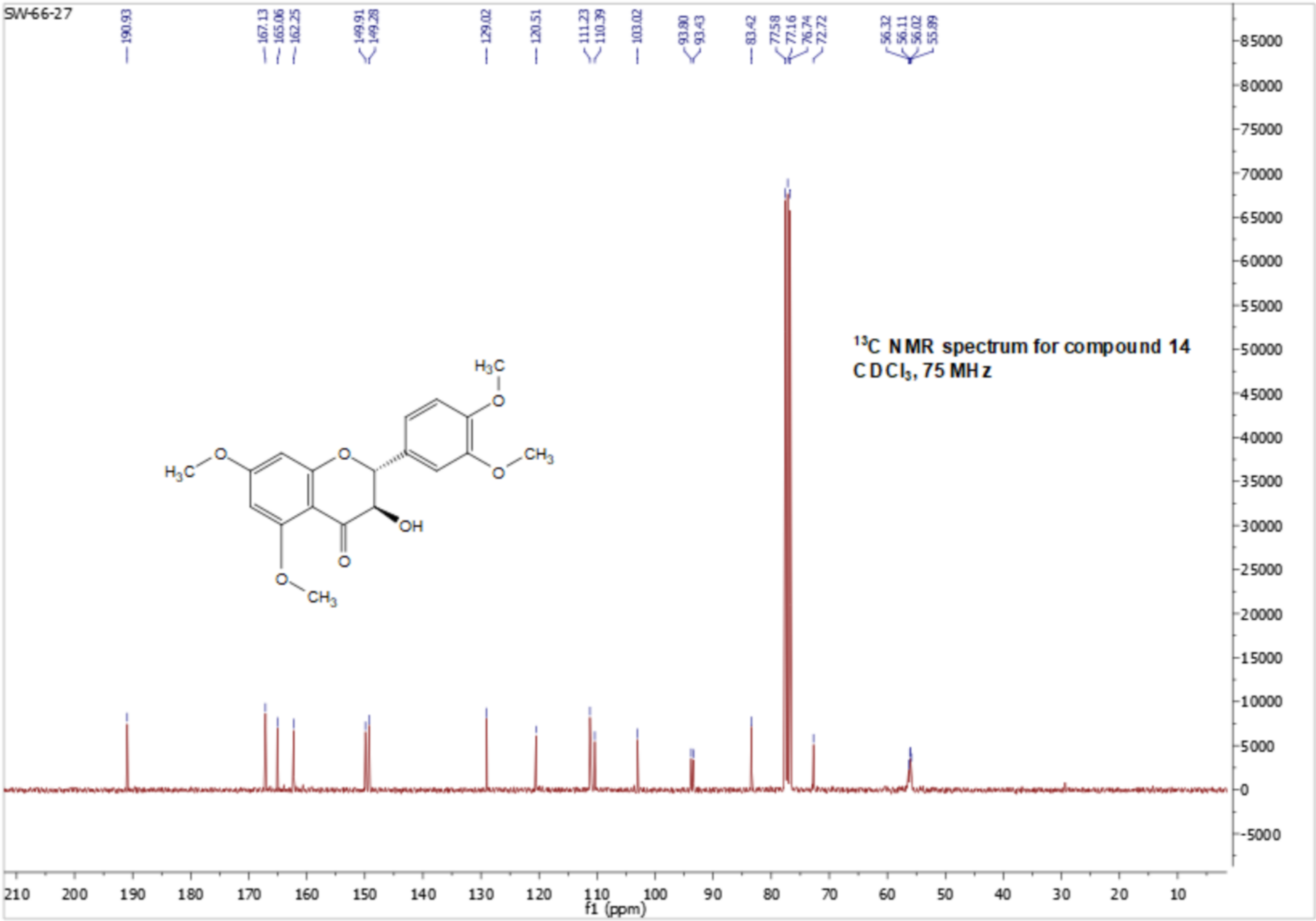

Figure S43: ^{13}C NMR spectrum of **14** in CDCl$_3$

Sample Name	Mol Fomula	MW	M+H	observed	delta	ppm
**SW-85-27**	**C19H20O7**	**360.1209**	**361.1287**	**361.1281**	**-0.0006**	**-1.77**

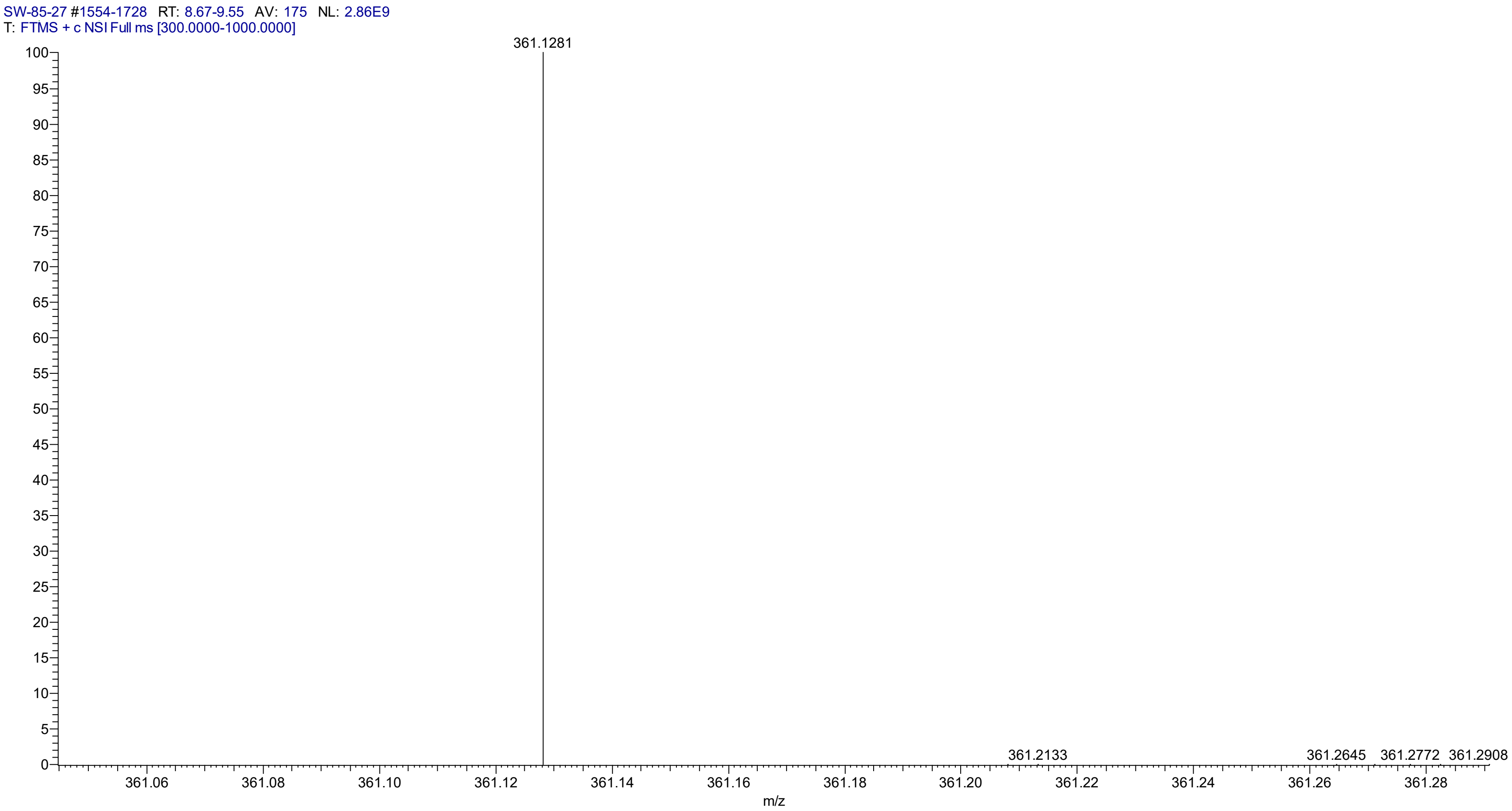

Figure S44: High resolution mass spectrum of **14**

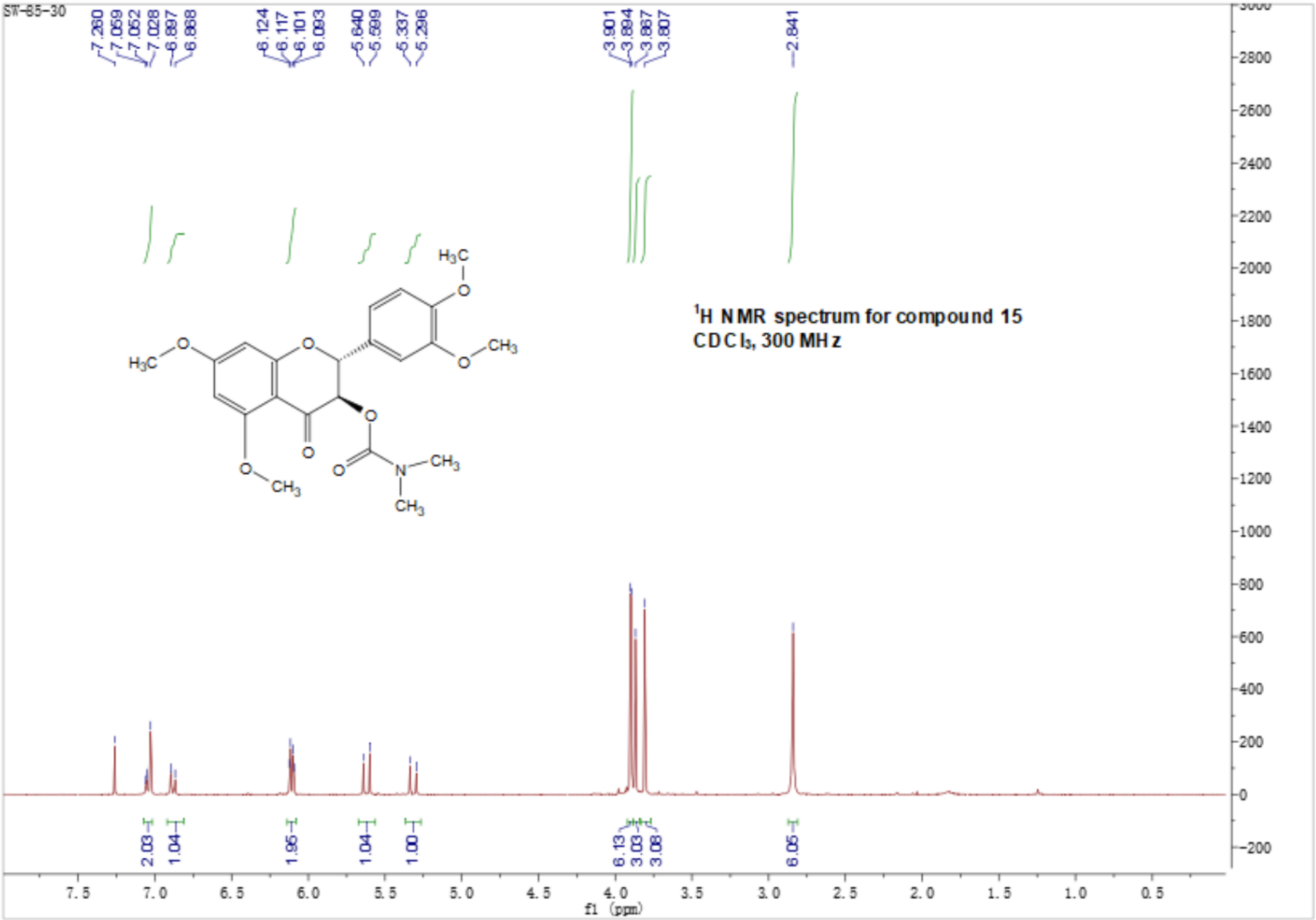

Figure S45: ^{1}H NMR spectrum of **15** in $CDCl_3$

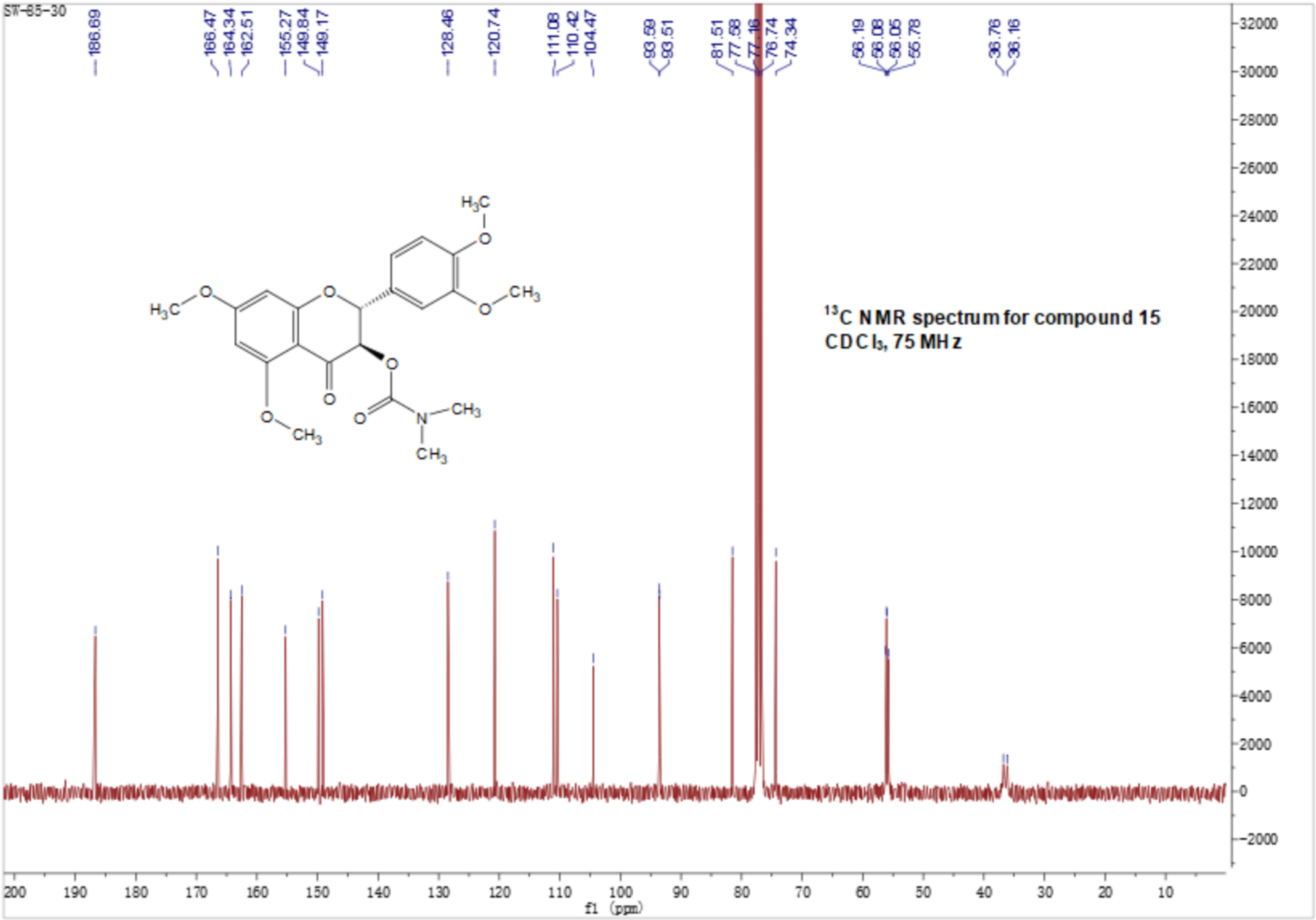

Figure S46: ^{13}C NMR spectrum of **15** in $CDCl_3$

Sample Name	Mol Fomula	MW	M+H	observed	delta	ppm
SW-85-30	C22H25NO8	431.1581	432.1659	432.1657	-0.0002	-0.37

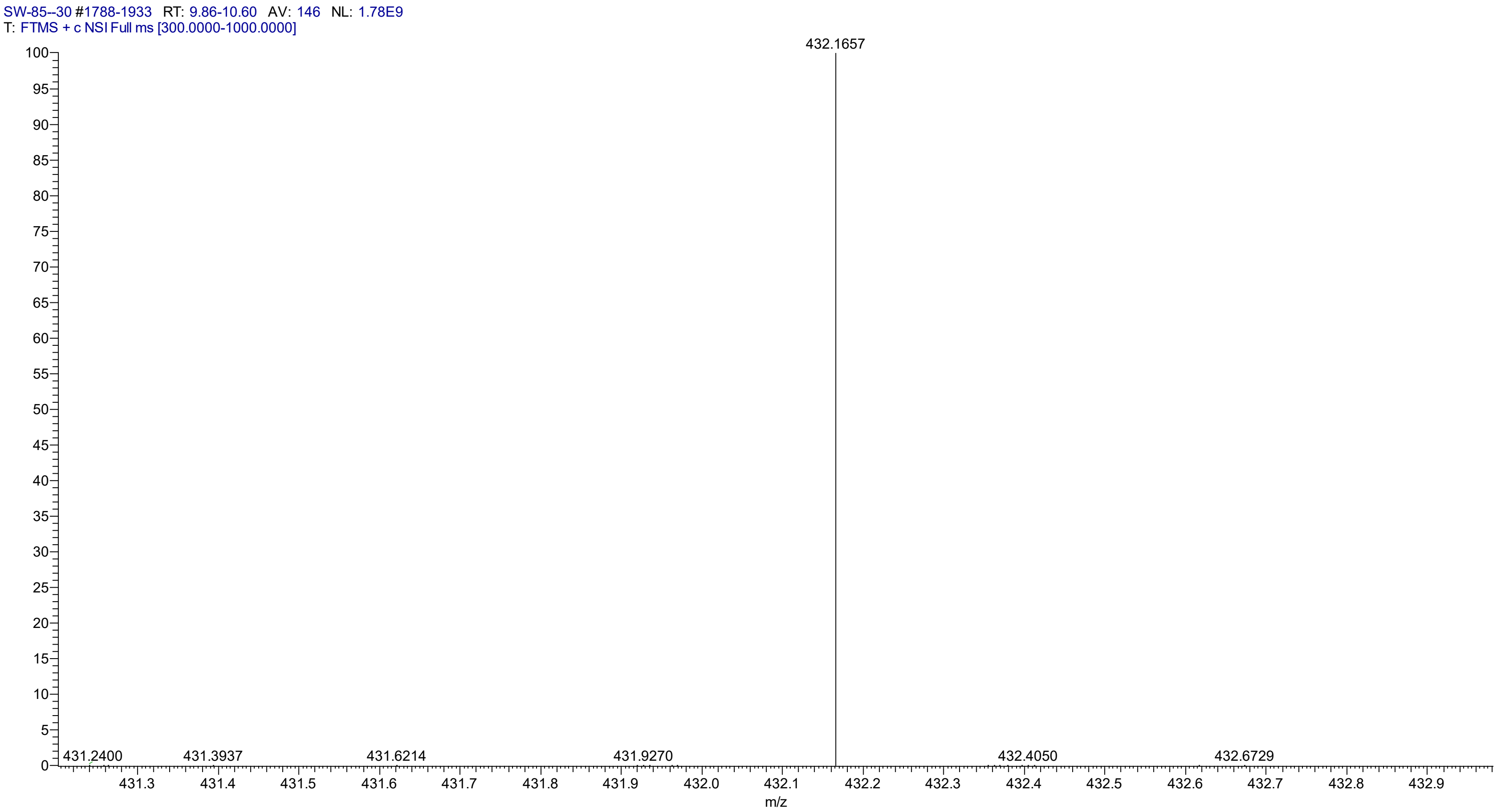

Figure S47: High resolution mass spectrum of **15**

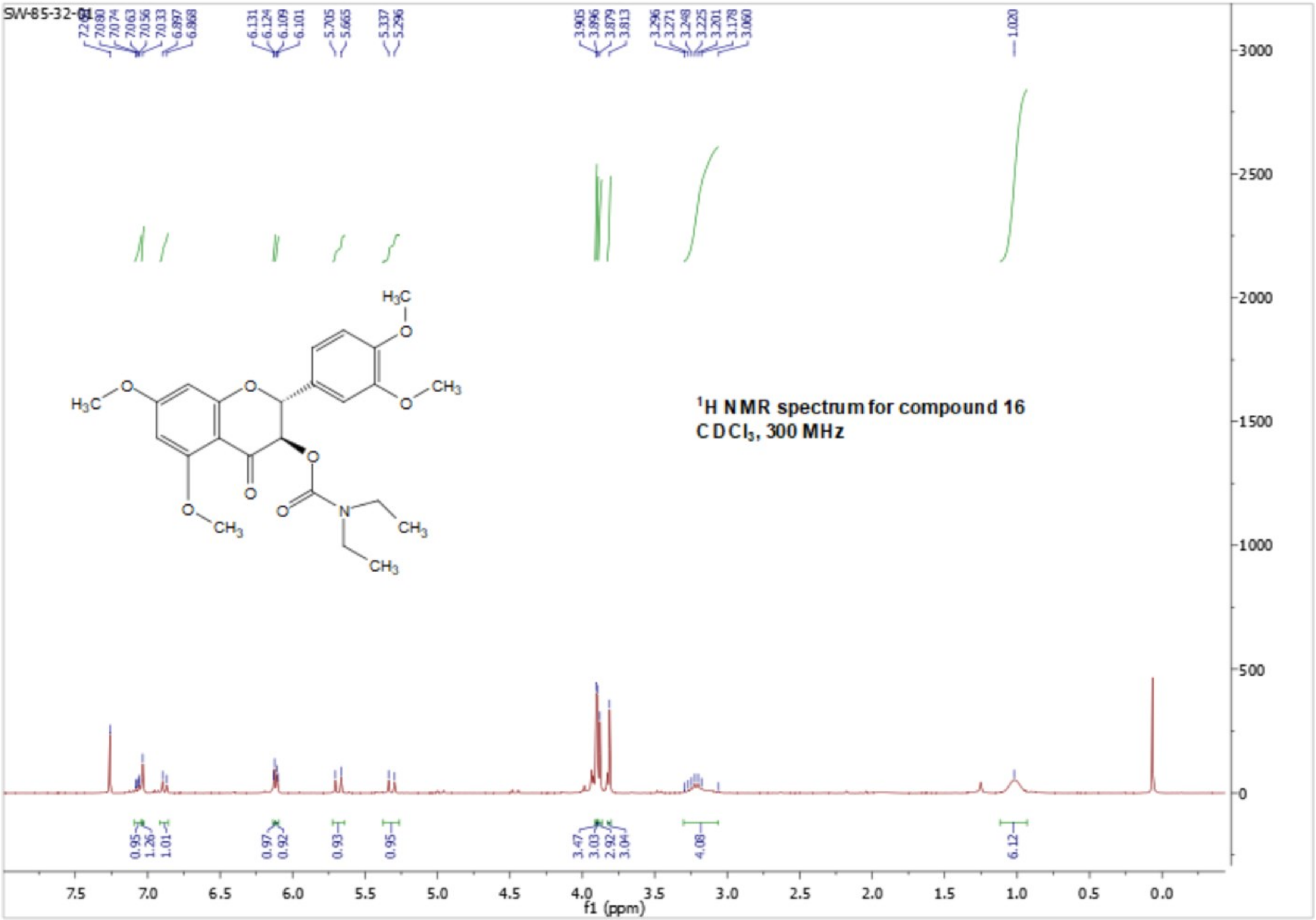

Figure S48: ^{1}H NMR spectrum of **16** in $CDCl_3$

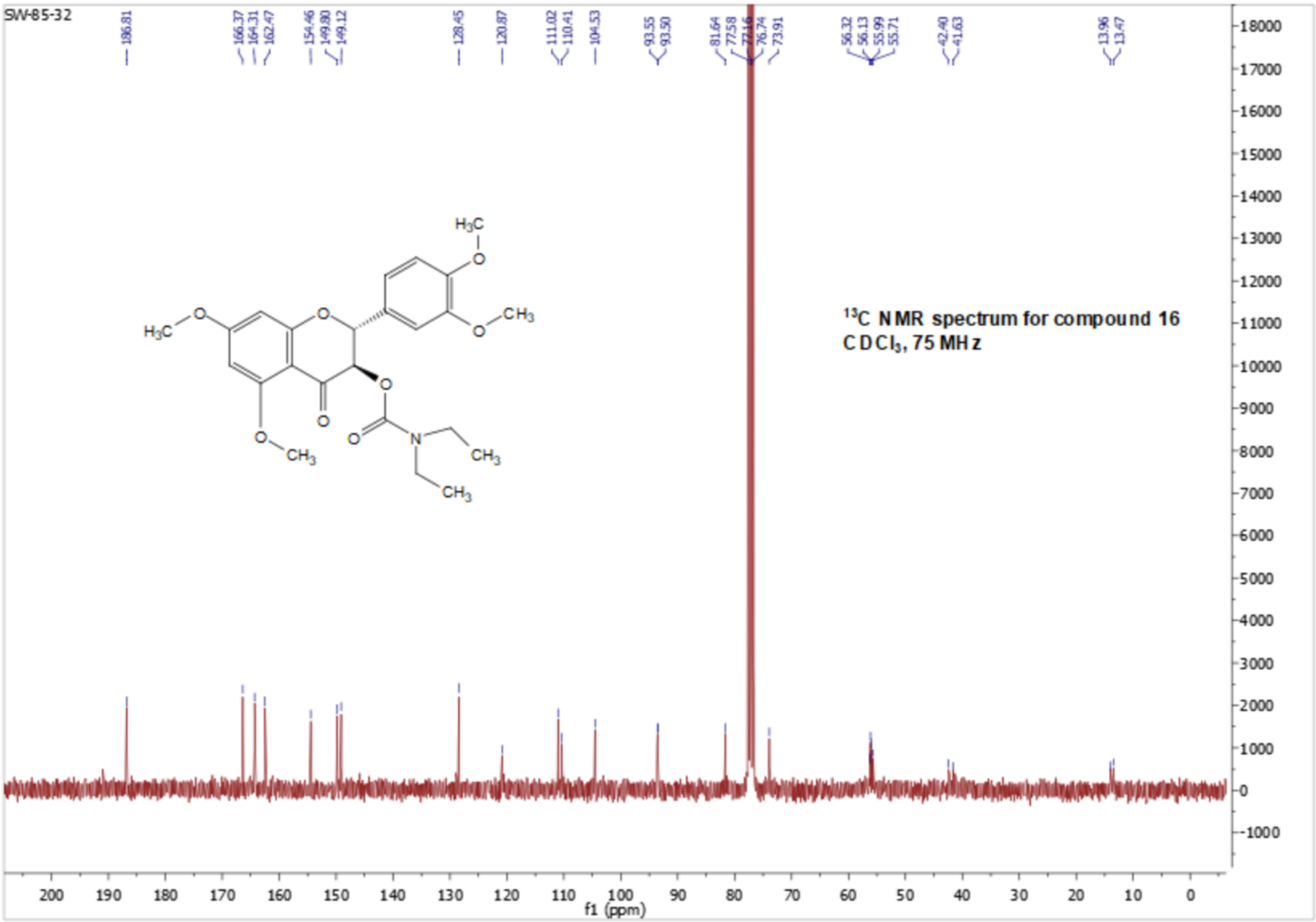

Figure S49: ^{13}C NMR spectrum of **16** in $CDCl_3$

Sample Name	Mol Fomula	MW	M+H	observed	delta	ppm
SW-85-32	C24H29NO8	459.1894	460.1972	460.1970	-0.0002	-0.35

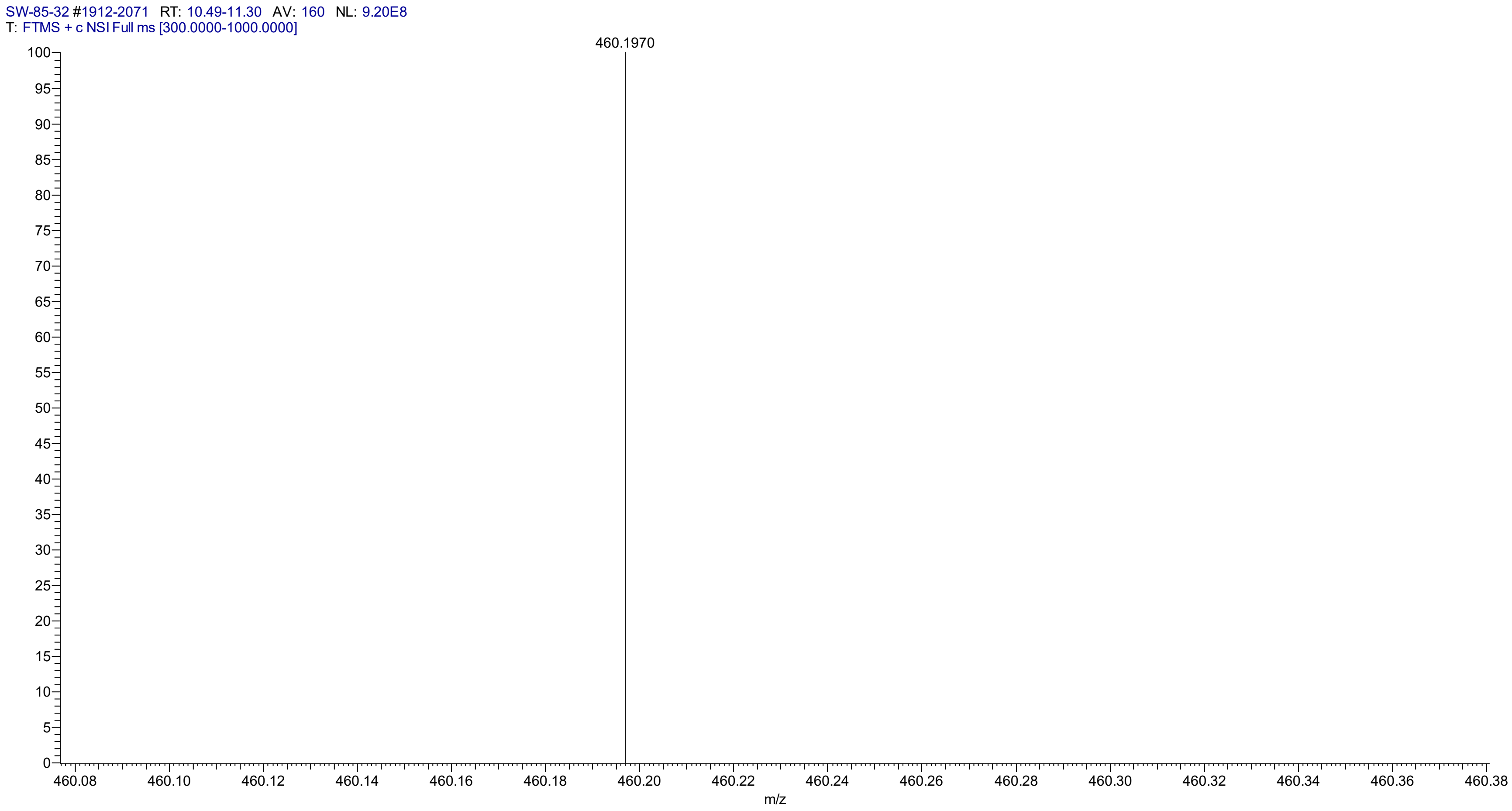

Figure S50: High resolution mass spectrum of **16**

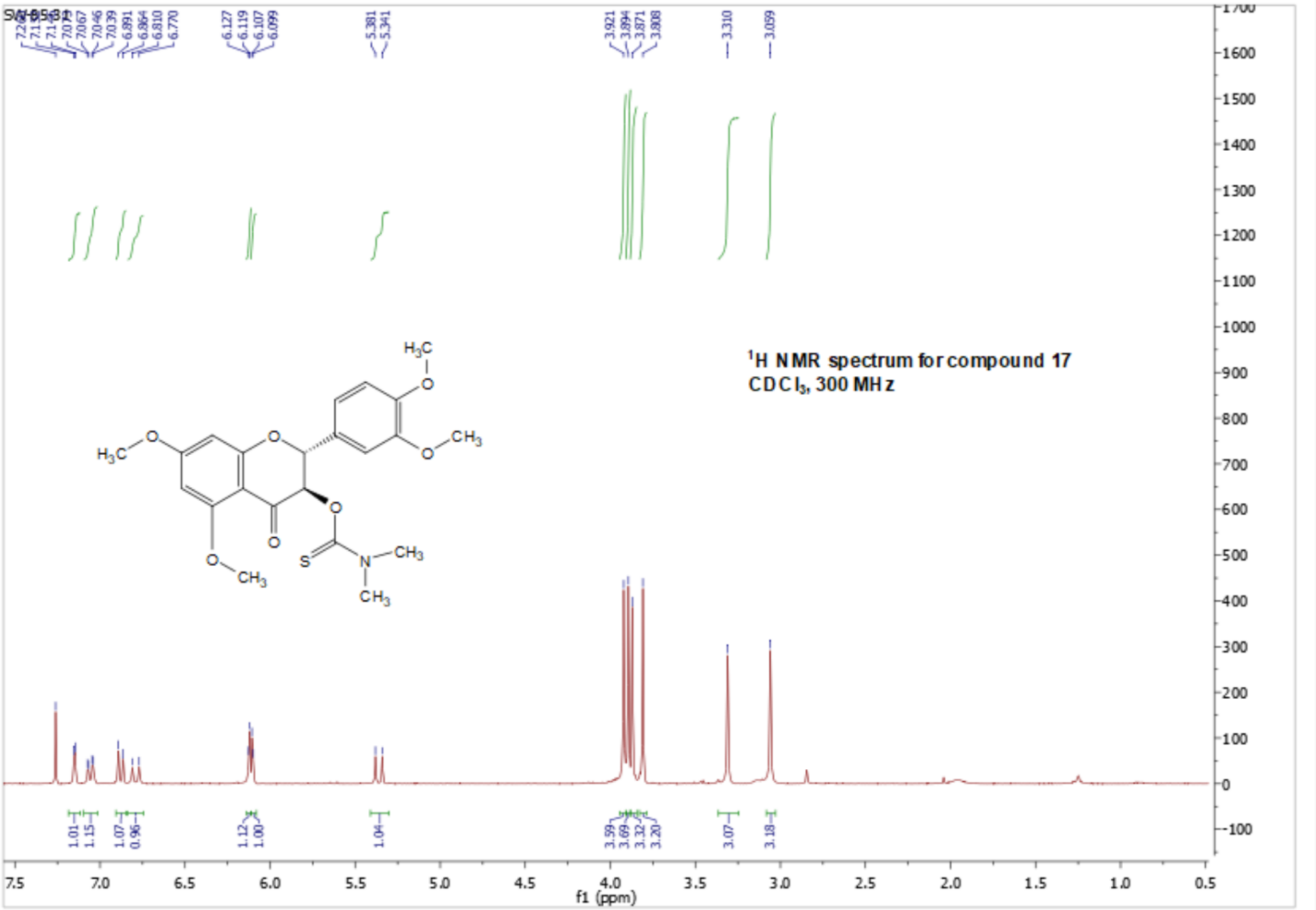

Figure S51: ^{1}H NMR spectrum of **17** in $CDCl_3$

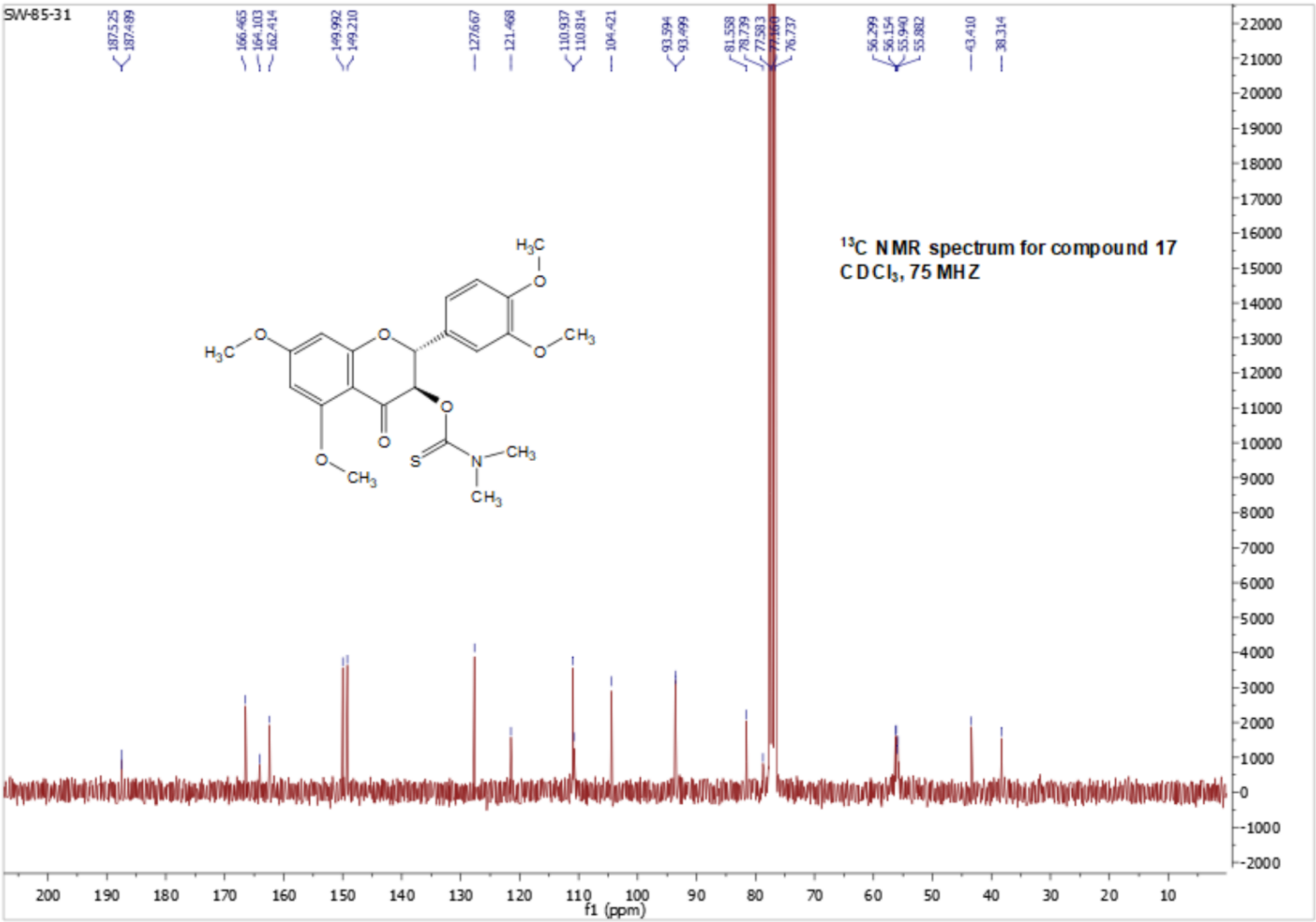

Figure S52: ^{13}C NMR spectrum of **17** in $CDCl_3$

Sample Name	Mol Fomula	MW	M+H	observed	delta	ppm
SW-85-31	C22H25NO7S	447.1352	448.1430	448.1431	0.0001	0.20

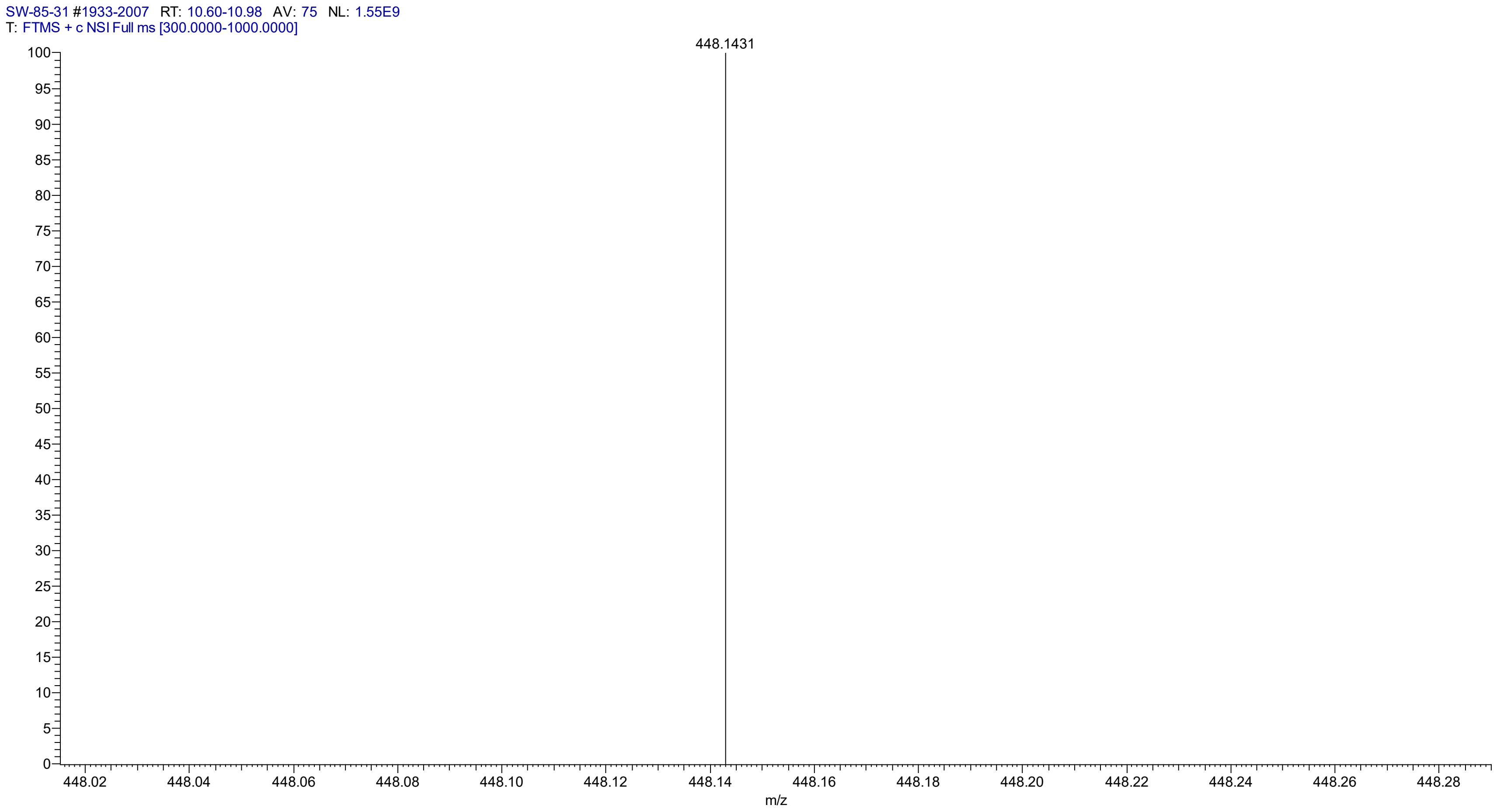

Figure S53: High resolution mass spectrum of **17**

Figure S54: The concentration-effect curves that were used to calculate the IC50 values from the WST-1 cell proliferation assay

1. The curves that we used to calculate the IC_{50} value for compound **3** against PC-3

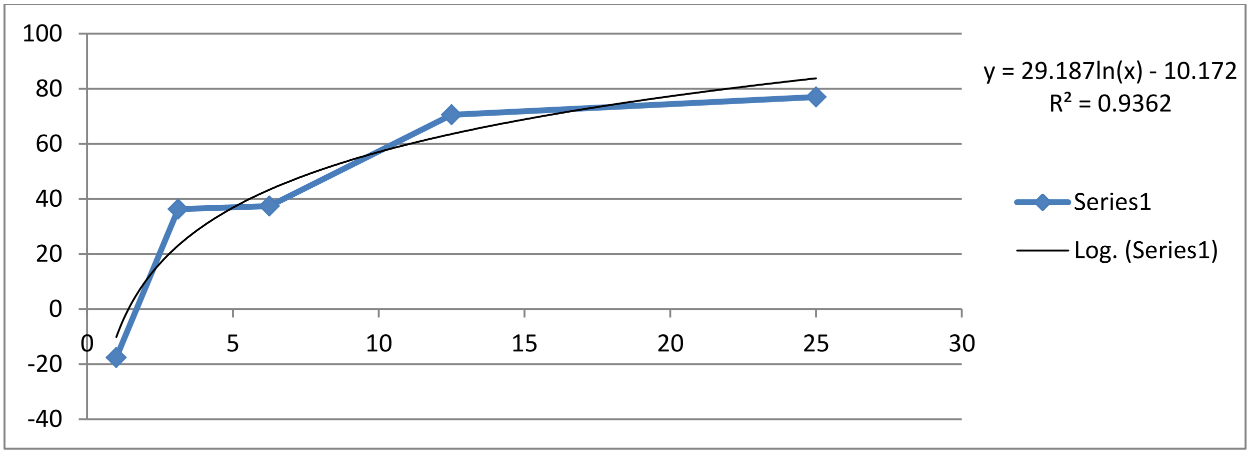

X–axis = concentration (µM) of compound **3**
Y–axis = % Cell Proliferation Inhibition
IC_{50} = 7.858 µM

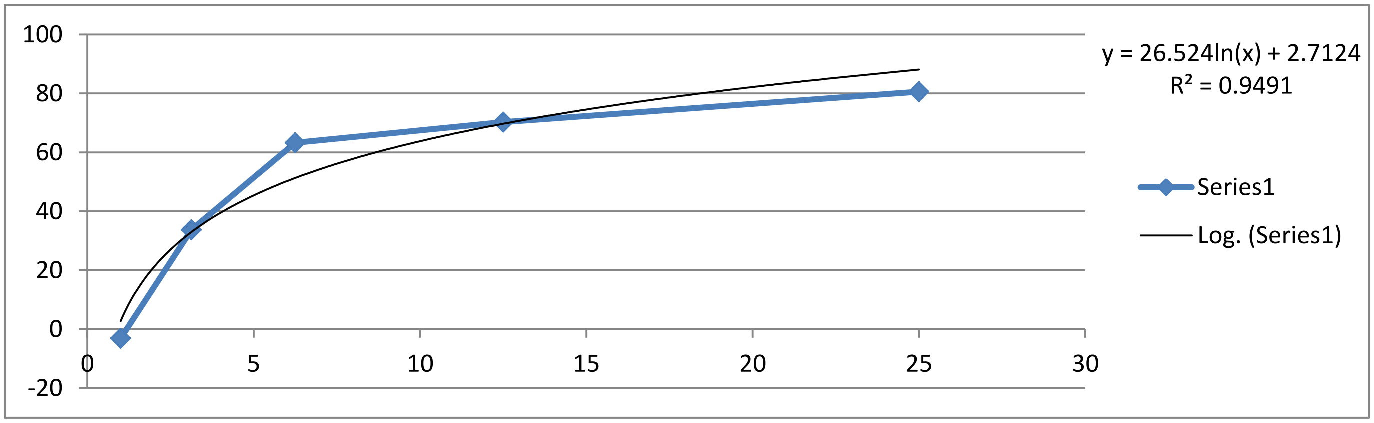

X–axis = concentration (µM) of compound **3**
Y–axis = % Cell Proliferation Inhibition
IC_{50} = 5.946 µM

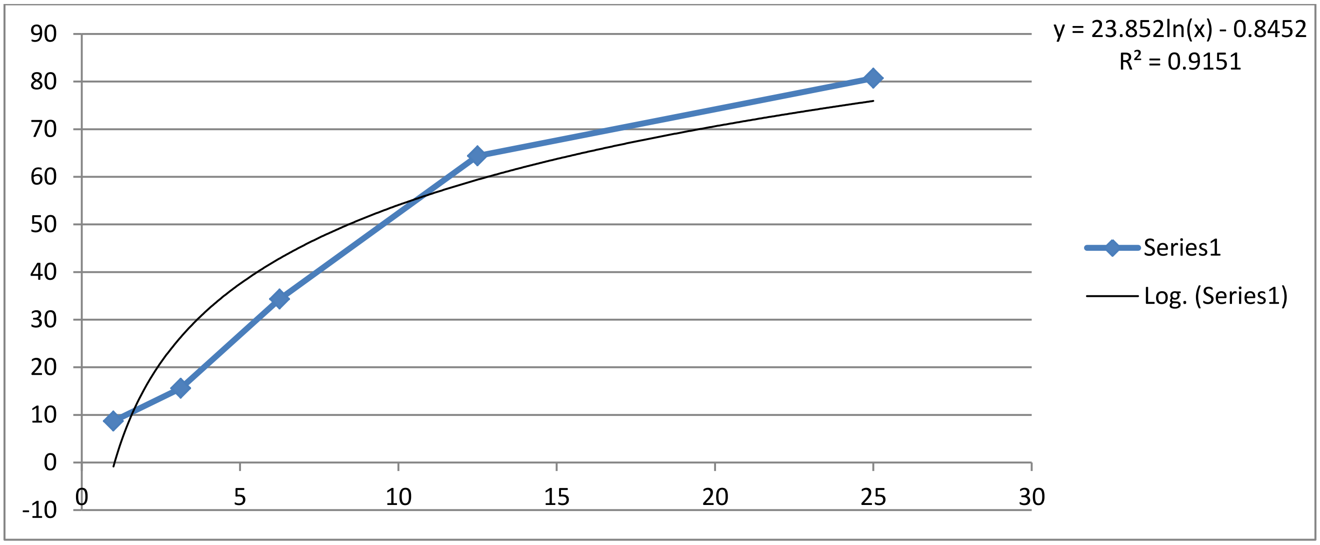

X–axis = concentration (μM) of compound **3**
Y–axis = % Cell Proliferation Inhibition
IC_{50} = 8.429 μM

2. The curves that we used to calculate the IC_{50} value for compound **3** against DU145

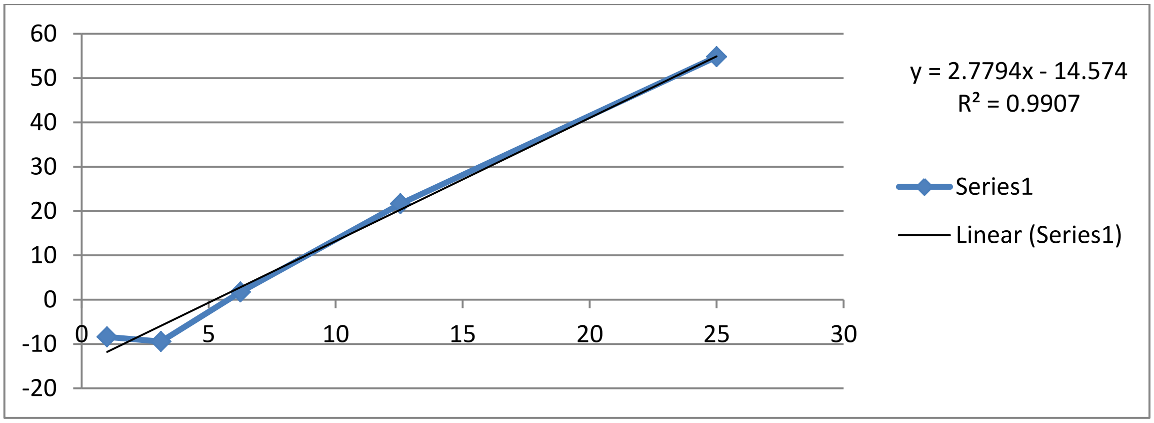

X–axis = concentration (μM) of compound **3**
Y–axis = % Cell Proliferation Inhibition
IC_{50} = 23.233 μM

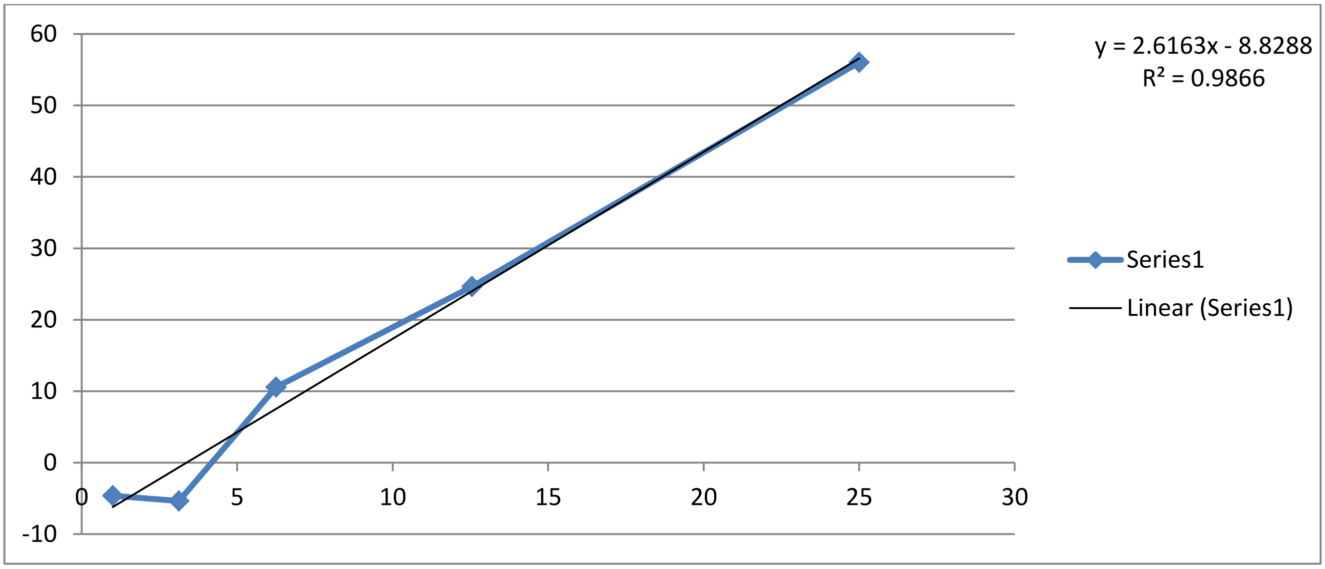

X–axis = concentration (μM) of compound **3**
Y–axis = % Cell Proliferation Inhibition
IC_{50} = 22.485 μM

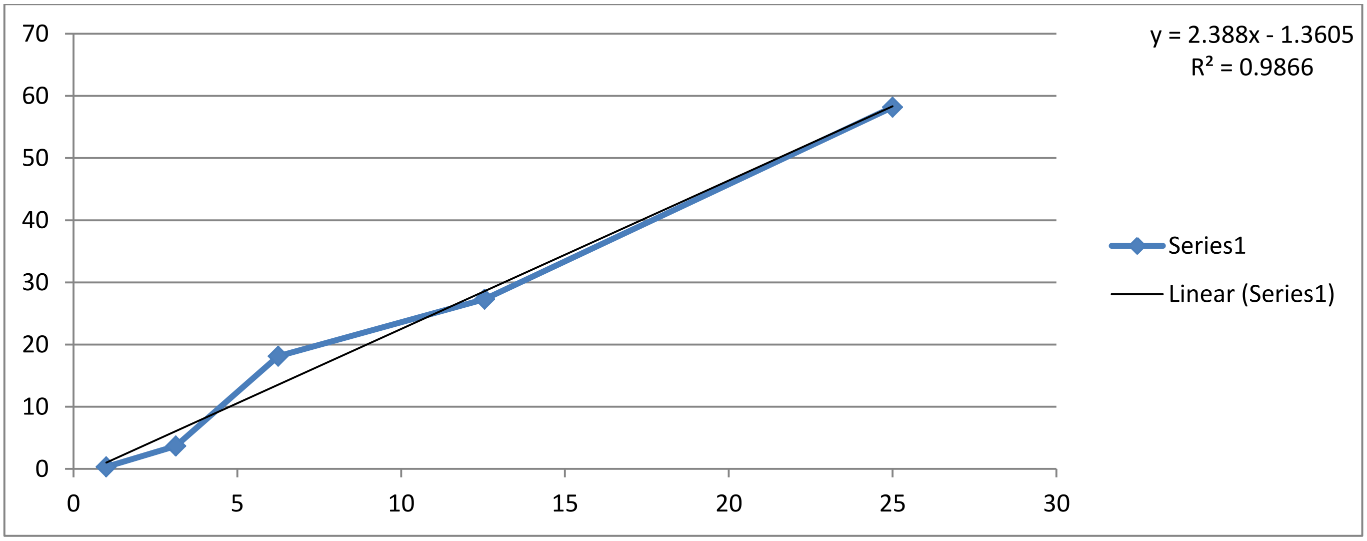

X–axis = concentration (μM) of compound **3**
Y–axis = % Cell Proliferation Inhibition
IC_{50} = 21.508 μM

3. The curves that we used to calculate the IC_{50} value for compound **3** against LNCaP

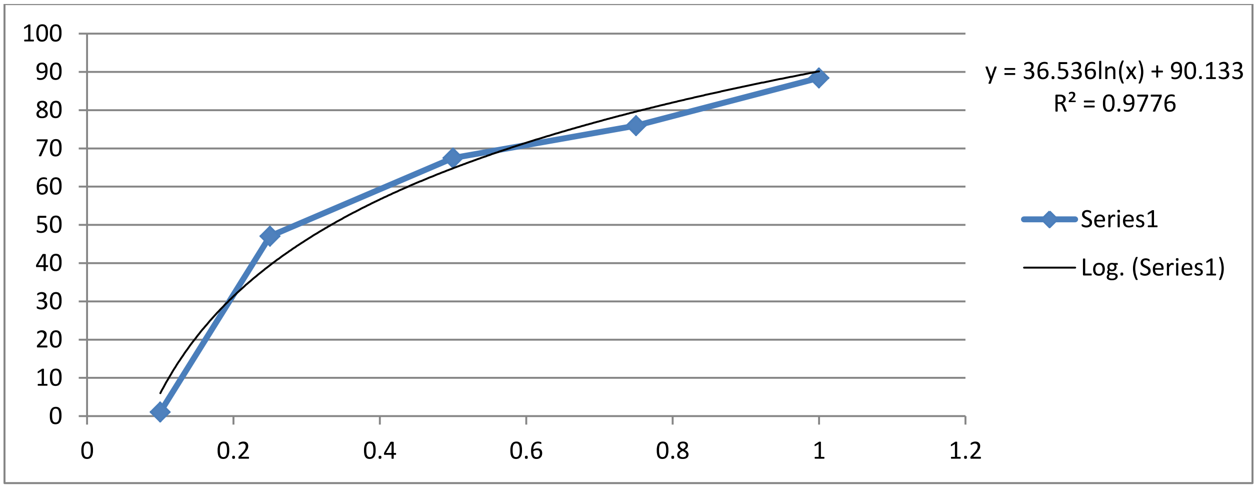

X–axis = concentration (μM) of compound **3**
Y–axis = % Cell Proliferation Inhibition
IC_{50} = 0.333 μM

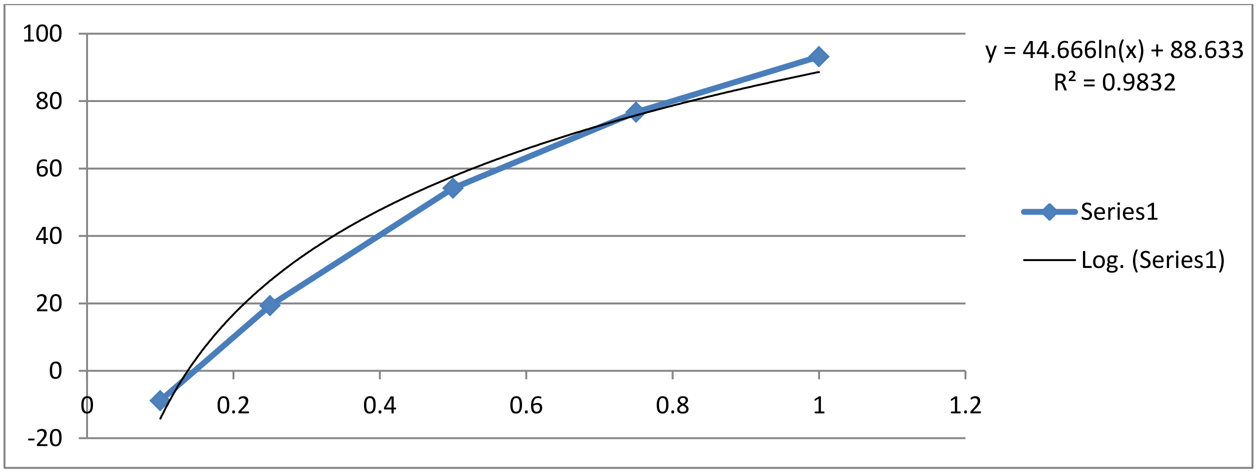

X–axis = concentration (μM) of compound **3**
Y–axis = % Cell Proliferation Inhibition
IC_{50} = 0.421 μM

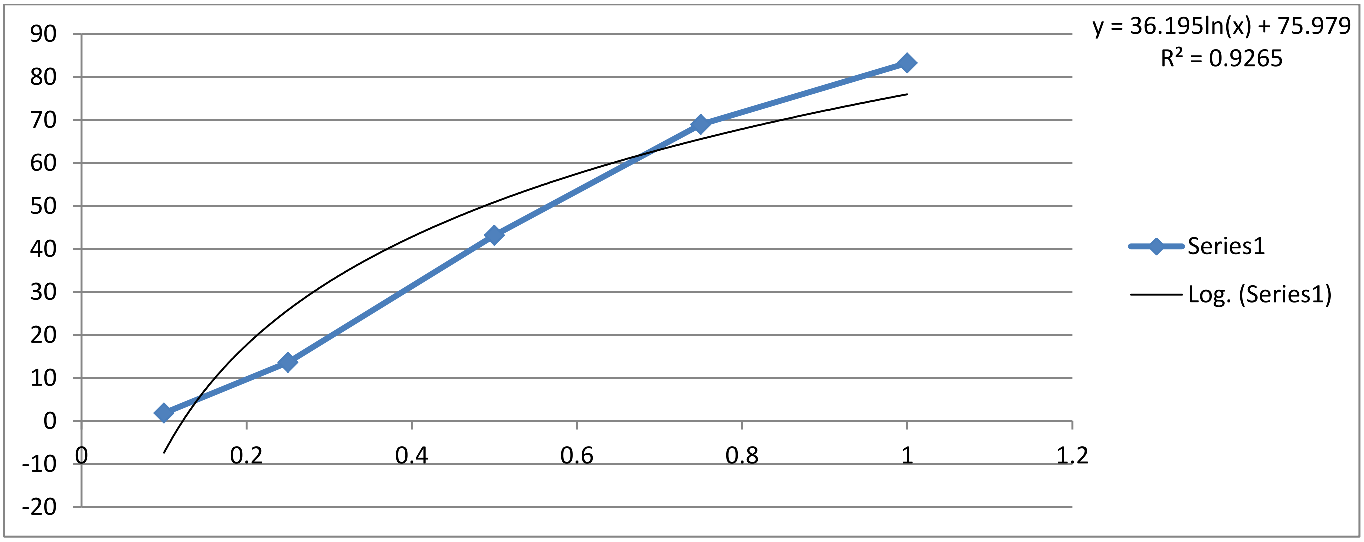

X–axis = concentration (μM) of compound **3**
Y–axis = % Cell Proliferation Inhibition
IC_{50} = 0.488 μM

4. The curves that we used to calculate the IC_{50} value for compound **4** against PC-3

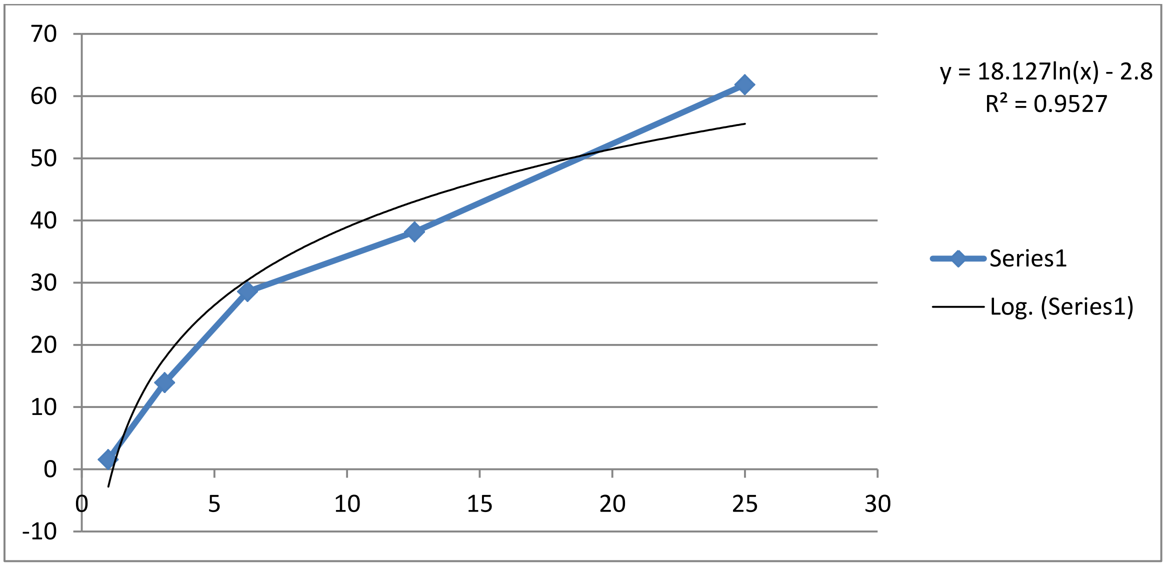

X–axis = concentration (μM) of compound **4**
Y–axis = % Cell Proliferation Inhibition
IC_{50} = 18.408 μM

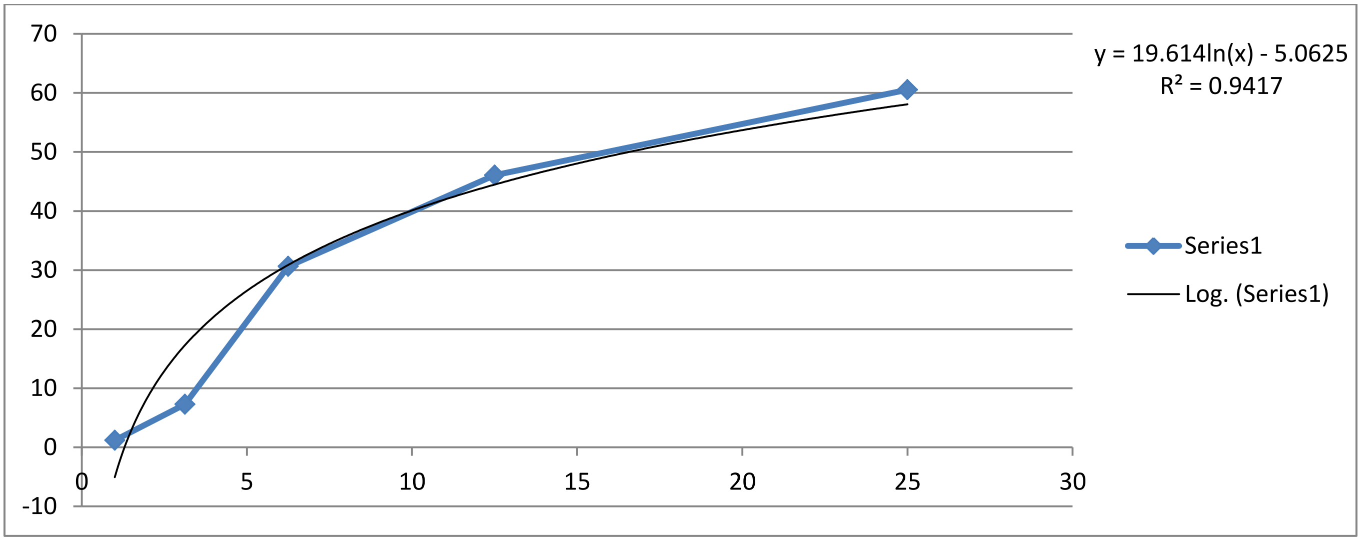

X–axis = concentration (μM) of compound **4**
Y–axis = % Cell Proliferation Inhibition
IC_{50} = 16.565 μM

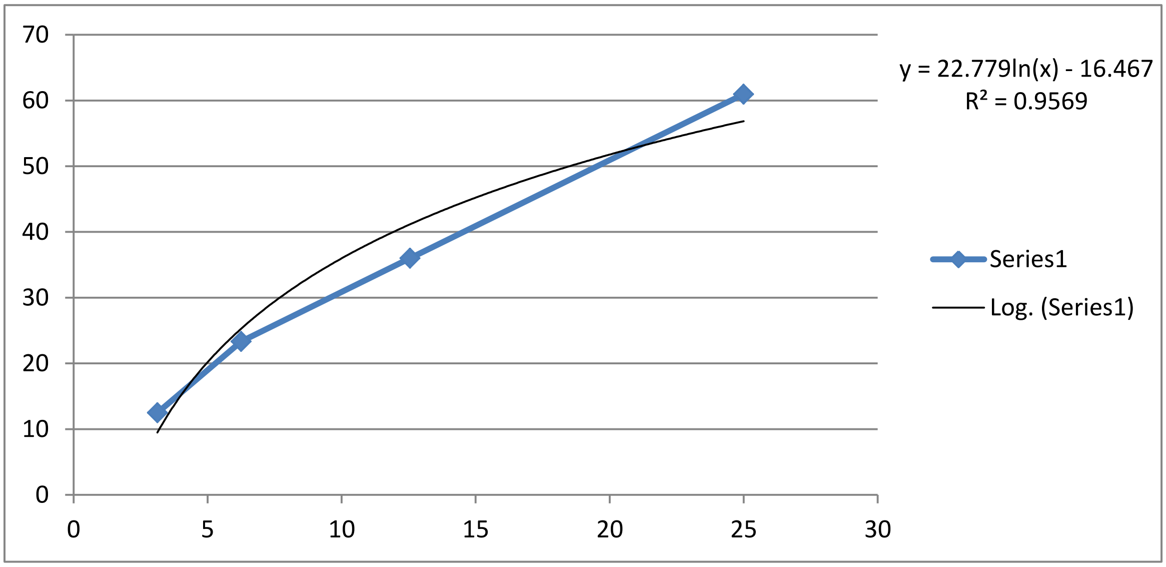

X–axis = concentration (μM) of compound **4**
Y–axis = % Cell Proliferation Inhibition
IC_{50} = 18.5025 μM

5. The curves that we used to calculate the IC_{50} value for compound **4** against DU145

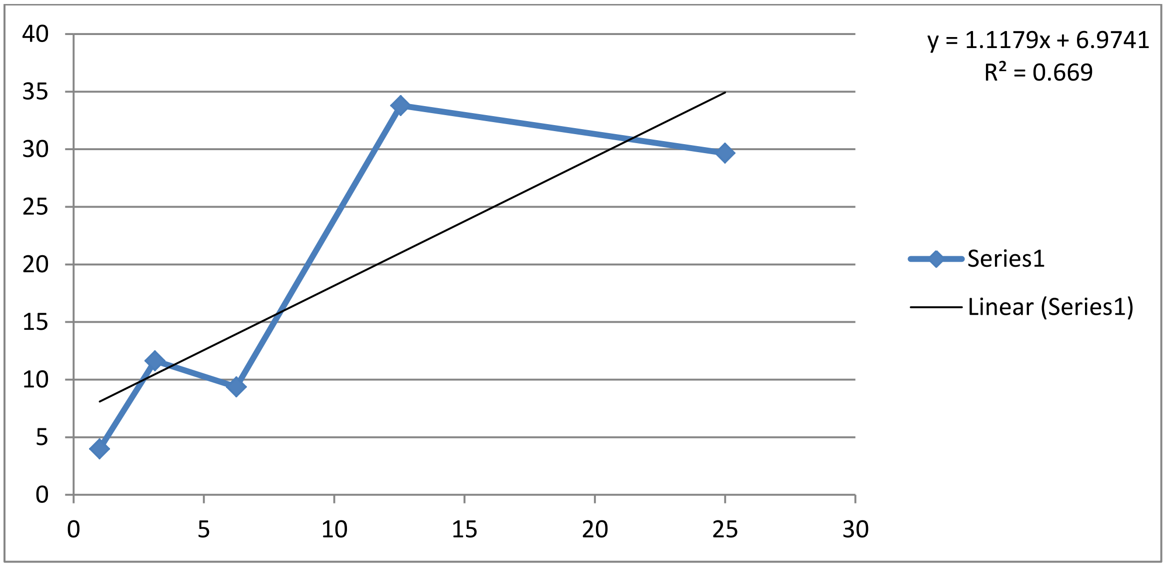

X–axis = concentration (μM) of compound **4**
Y–axis = % Cell Proliferation Inhibition
IC_{50} > 25 μM

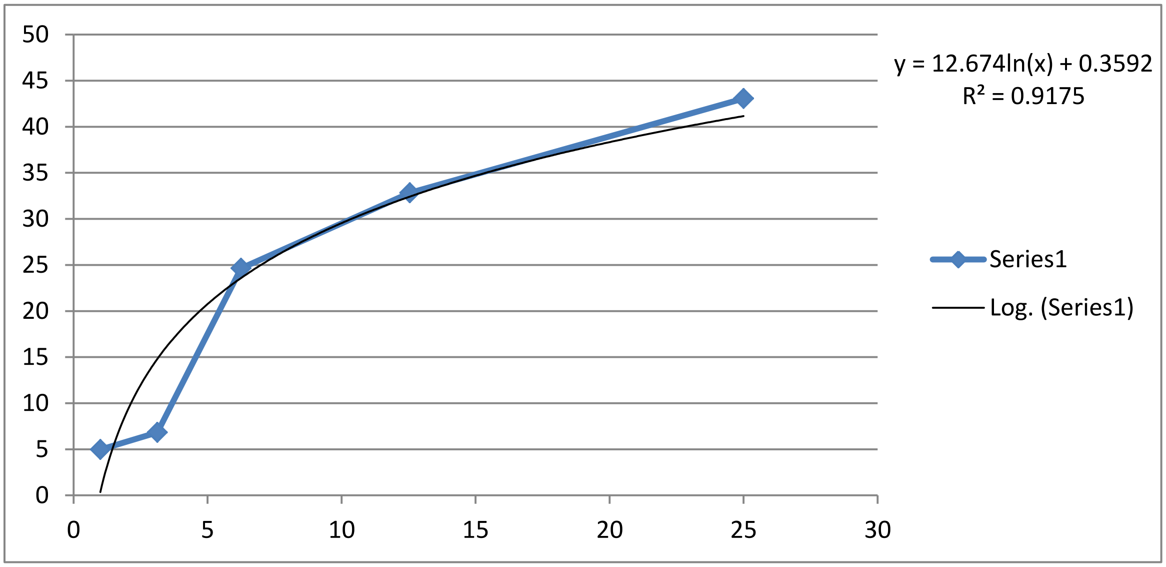

X–axis = concentration (μM) of compound **4**
Y–axis = % Cell Proliferation Inhibition
IC_{50} > 25 μM

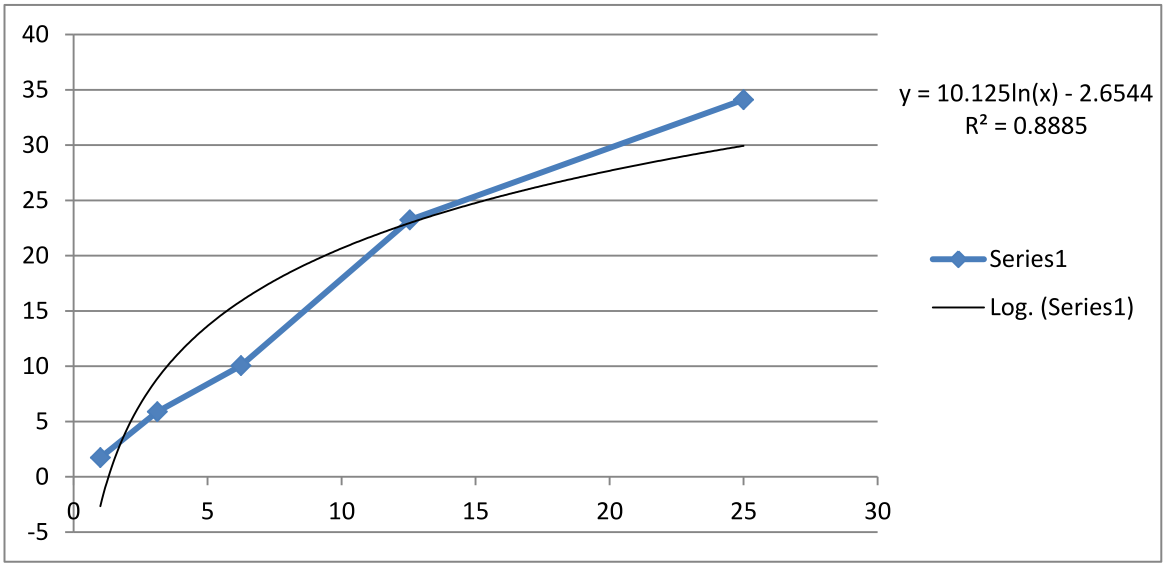

X–axis = concentration (μM) of compound **4**
Y–axis = % Cell Proliferation Inhibition
IC_{50} > 25 μM

6. The curves that we used to calculate the IC_{50} value for compound **4** against LNCaP

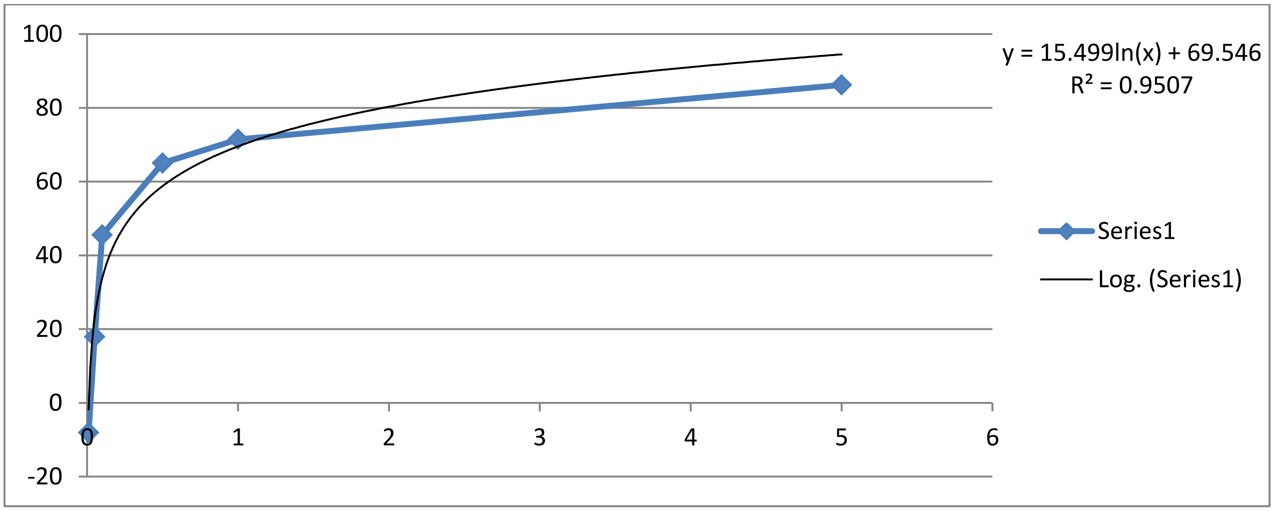

X–axis = concentration (μM) of compound **4**
Y–axis = % Cell Proliferation Inhibition
IC_{50} = 0.283 μM

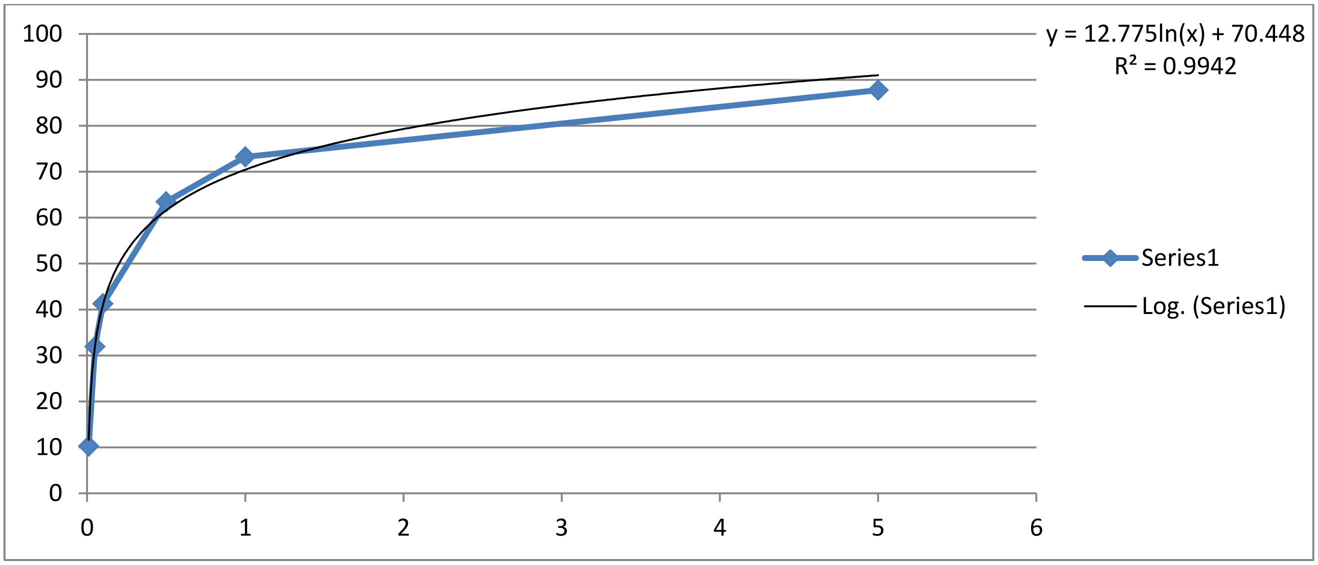

X–axis = concentration (μM) of compound **4**
Y–axis = % Cell Proliferation Inhibition
IC_{50} = 0.202 μM

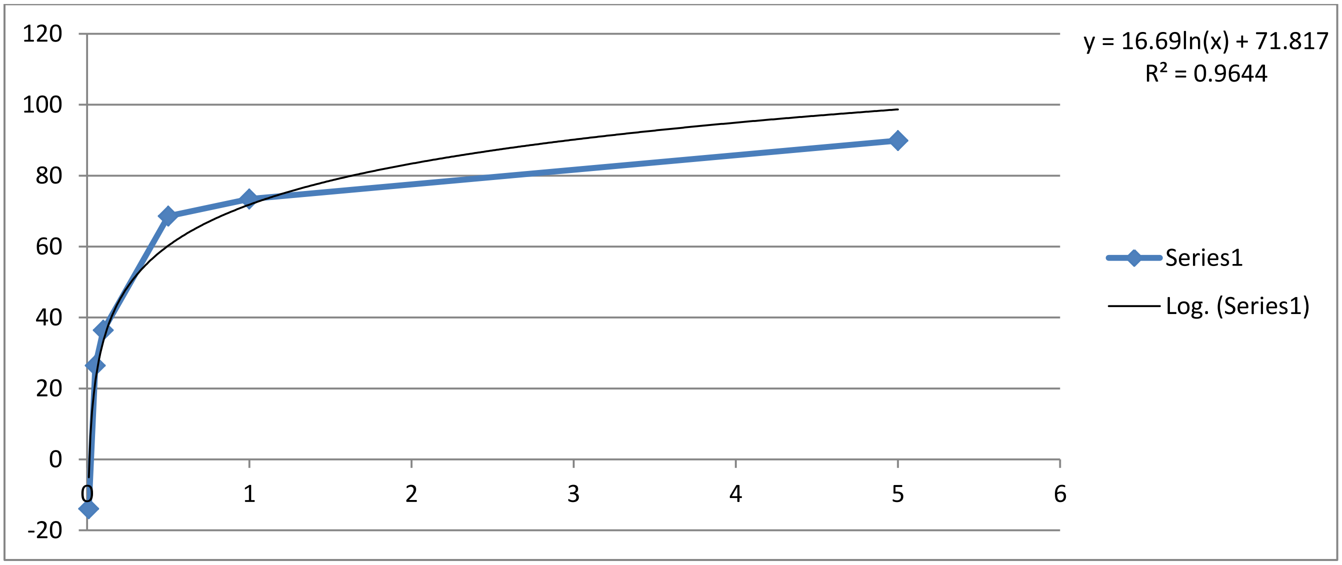

X–axis = concentration (μM) of compound **4**
Y–axis = % Cell Proliferation Inhibition
IC_{50} = 0.271 μM

7. The curves that we used to calculate the IC_{50} value for compound **5** against PC-3

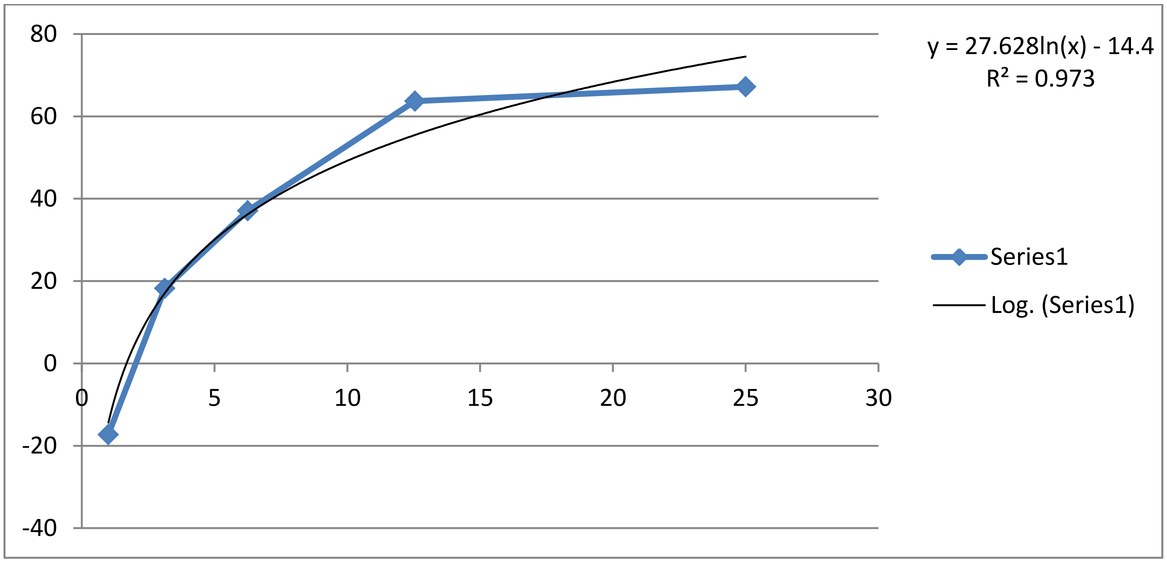

X–axis = concentration (µM) of compound **5**
Y–axis = % Cell Proliferation Inhibition
IC_{50} = 10.288 µM

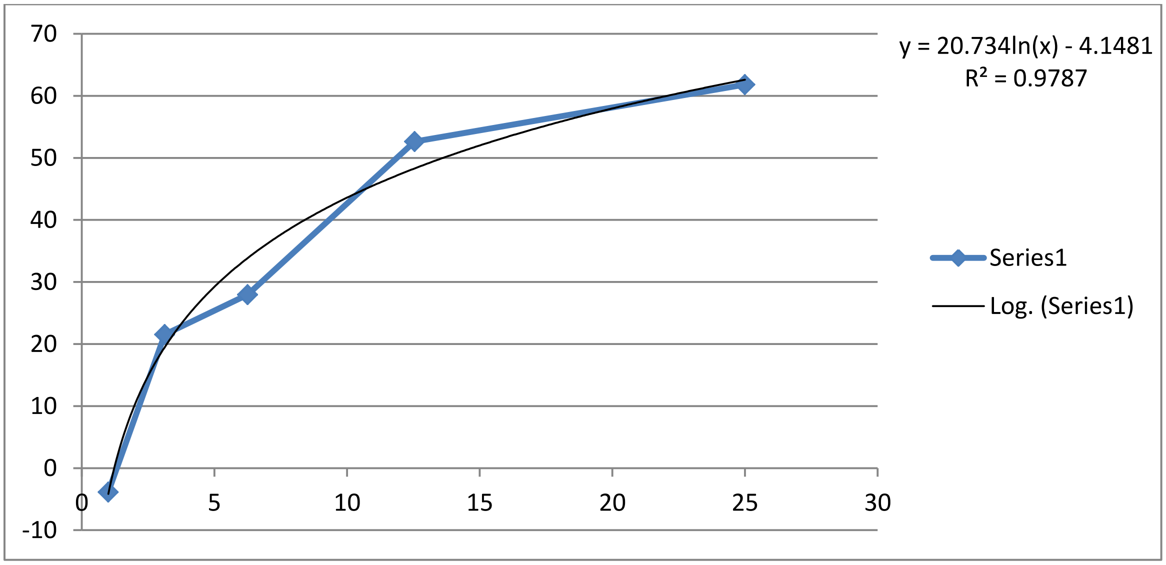

X–axis = concentration (µM) of compound **5**
Y–axis = % Cell Proliferation Inhibition
IC_{50} = 13.620 µM

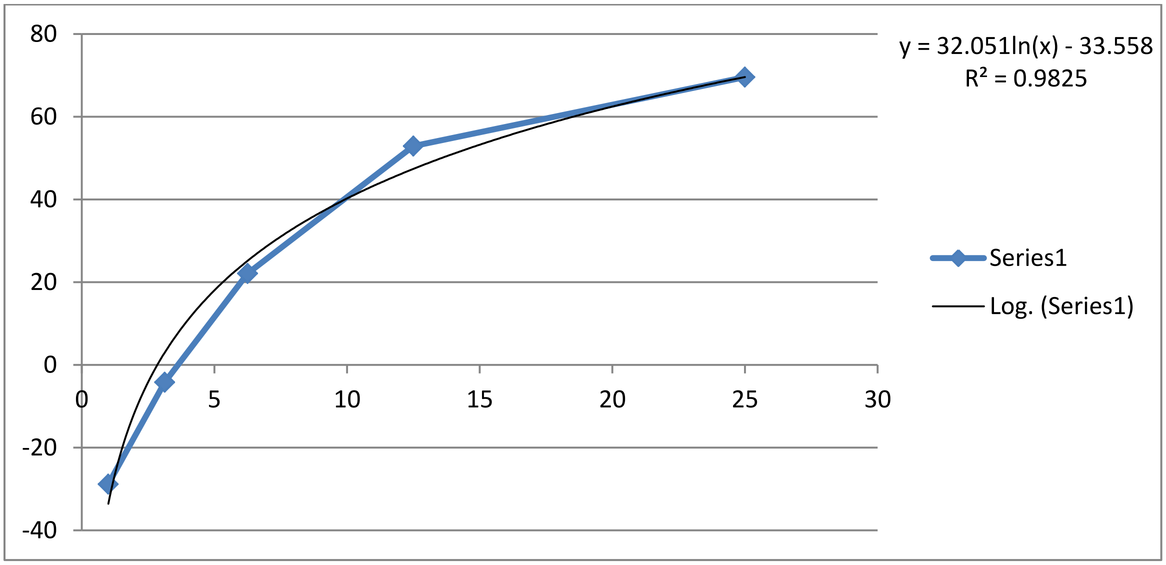

X–axis = concentration (µM) of compound **5**
Y–axis = % Cell Proliferation Inhibition
IC_{50} = 13.575 µM

8. The curves that we used to calculate the IC_{50} value for compound **5** against DU145

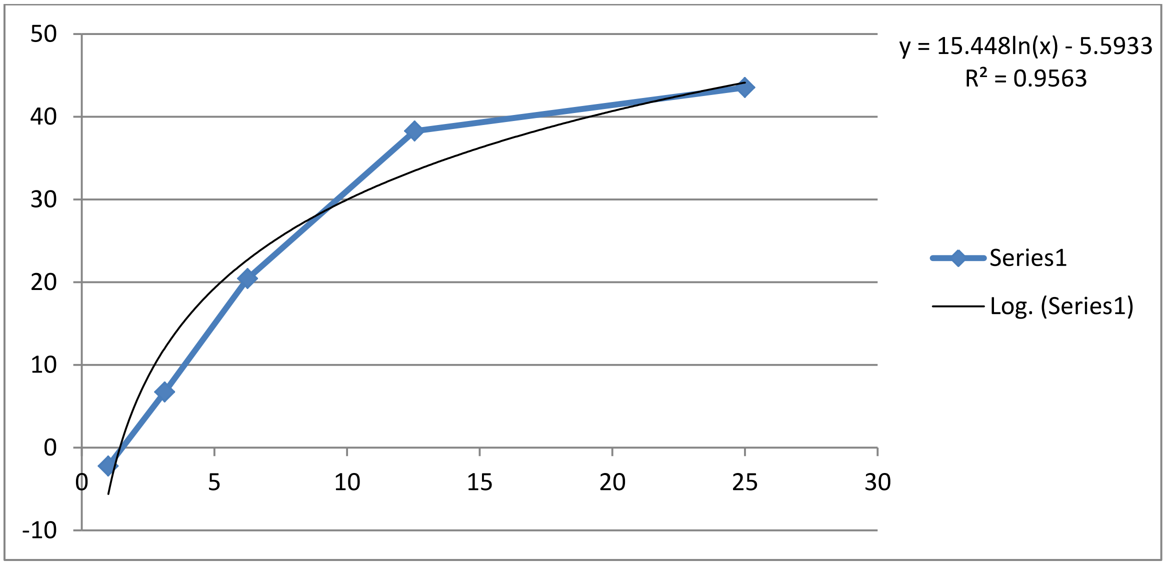

X–axis = concentration (µM) of compound **5**
Y–axis = % Cell Proliferation Inhibition
IC_{50} > 25 µM

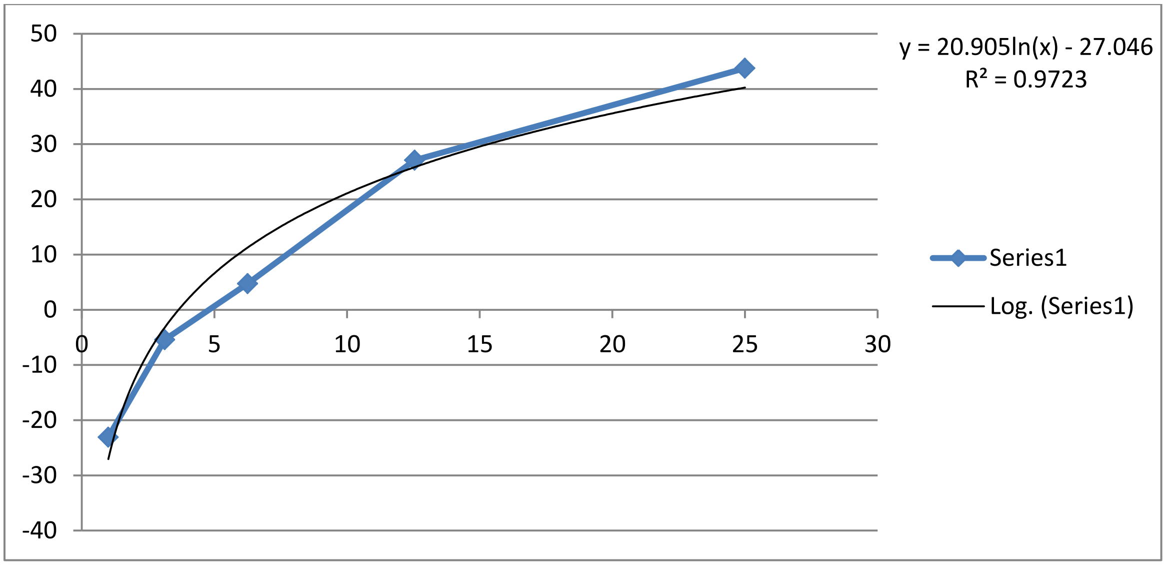

X–axis = concentration (μM) of compound **5**
Y–axis = % Cell Proliferation Inhibition
$IC_{50} > 25$ μM

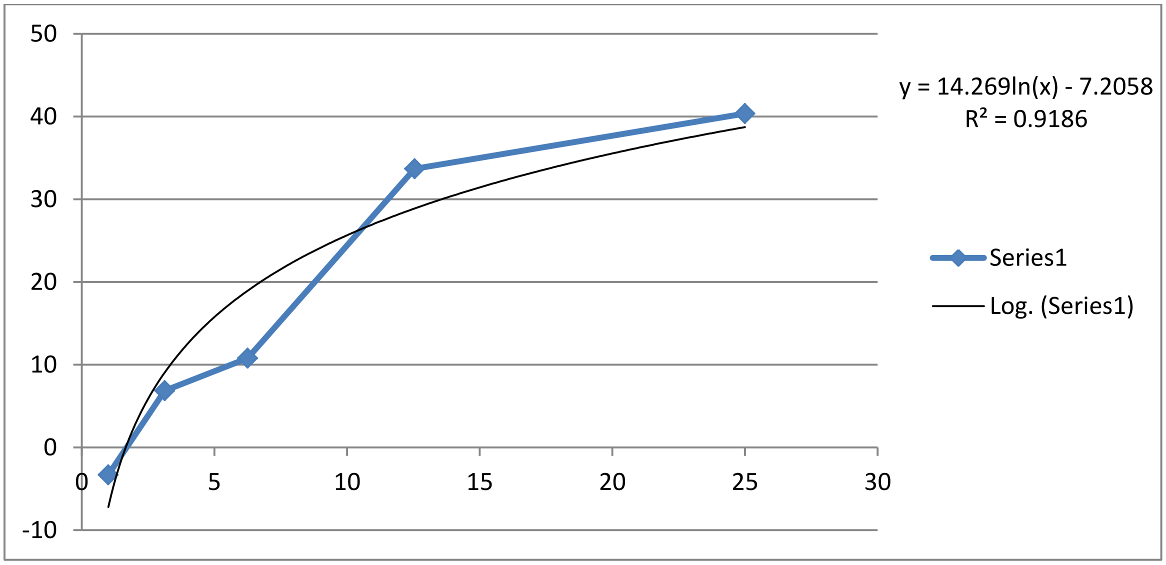

X–axis = concentration (μM) of compound **5**
Y–axis = % Cell Proliferation Inhibition
$IC_{50} > 25$ μM

9. The curves that we used to calculate the IC_{50} value for compound **5** against LNCaP

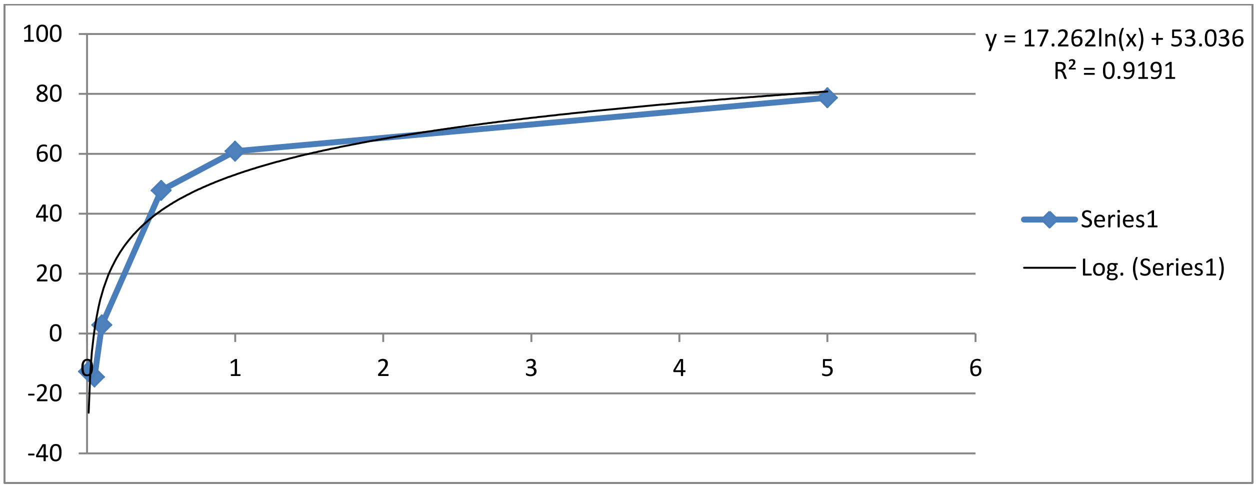

X–axis = concentration (µM) of compound **5**
Y–axis = % Cell Proliferation Inhibition
IC_{50} = 0.839 µM

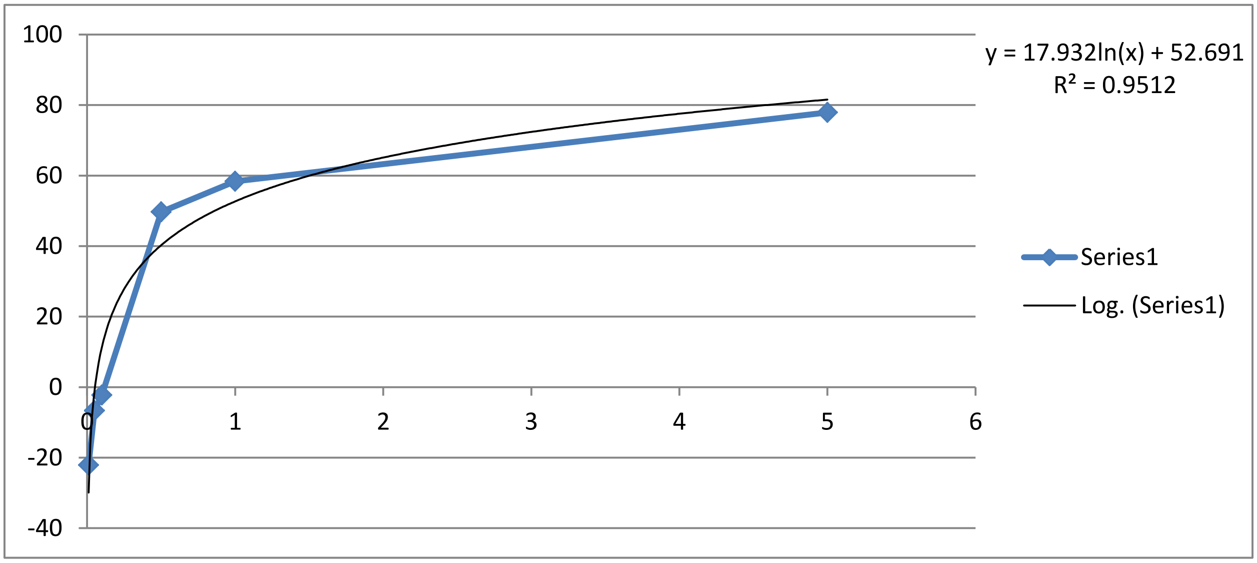

X–axis = concentration (µM) of compound **5**
Y–axis = % Cell Proliferation Inhibition
IC_{50} = 0.861 µM

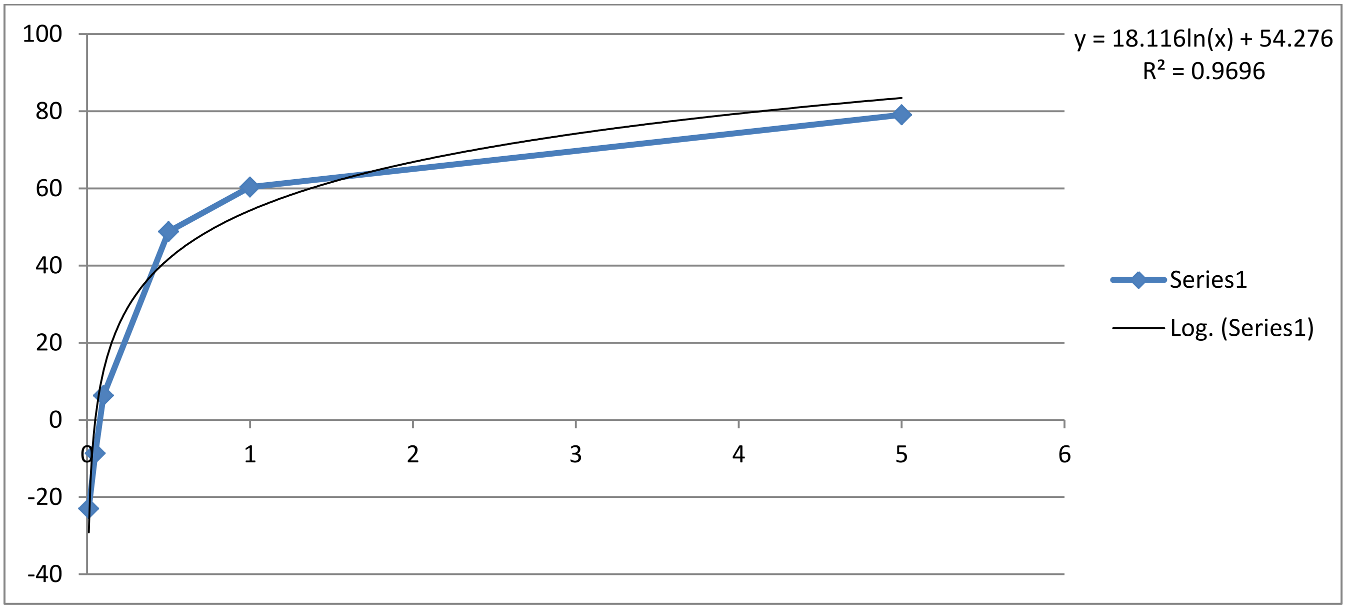

X–axis = concentration (μM) of compound **5**
Y–axis = % Cell Proliferation Inhibition
IC_{50} = 0.790 μM

10. The curves that we used to calculate the IC_{50} value for compound **6** against PC-3

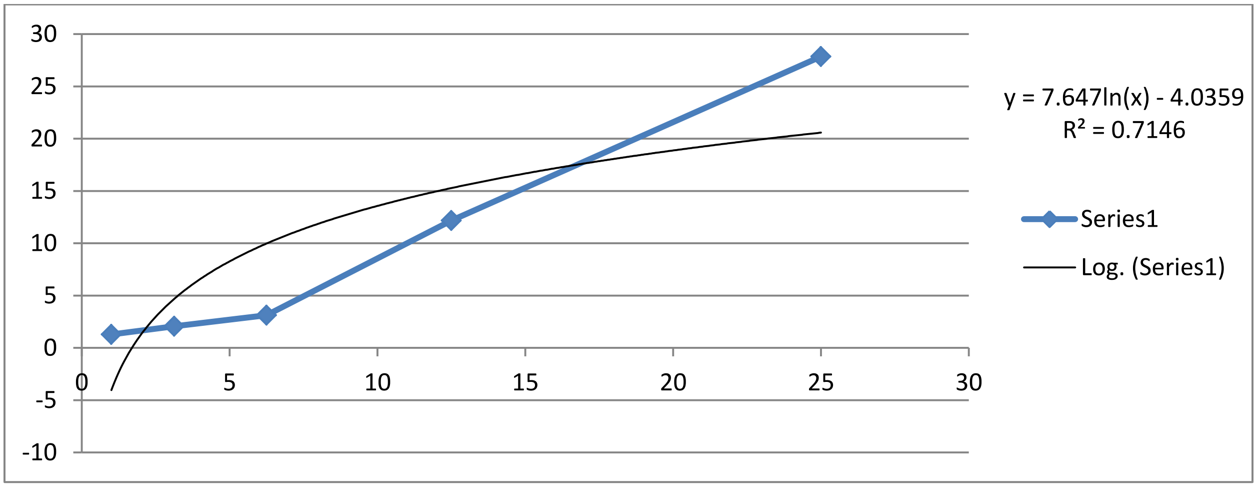

X–axis = concentration (μM) of compound **6**
Y–axis = % Cell Proliferation Inhibition
IC_{50} > 25 μM

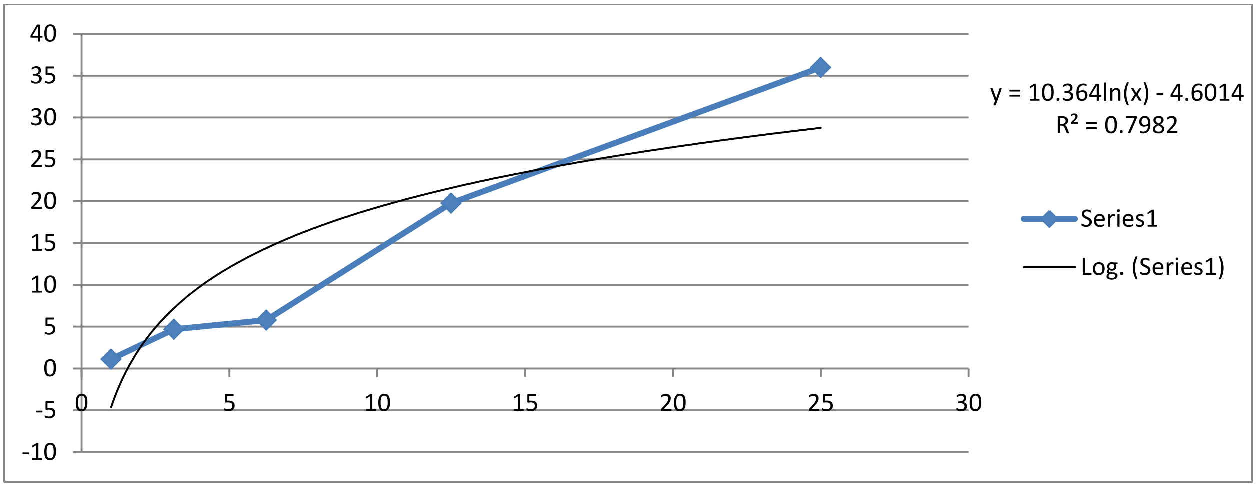

X–axis = concentration (μM) of compound **6**
Y–axis = % Cell Proliferation Inhibition
$IC_{50} > 25$ μM

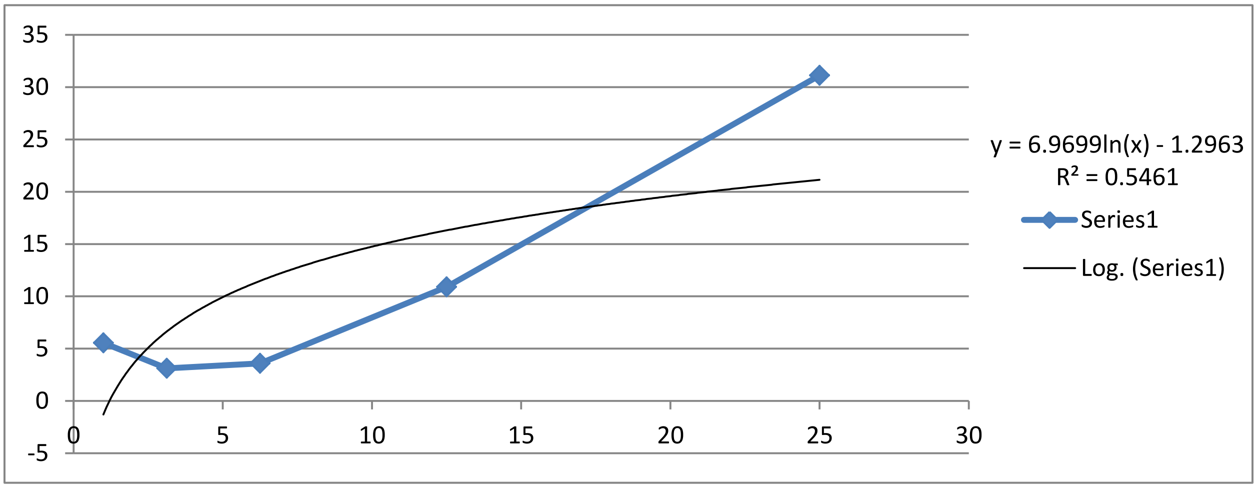

X–axis = concentration (μM) of compound **6**
Y–axis = % Cell Proliferation Inhibition
$IC_{50} > 25$ μM

11. The curves that we used to calculate the IC_{50} value for compound **6** against DU145

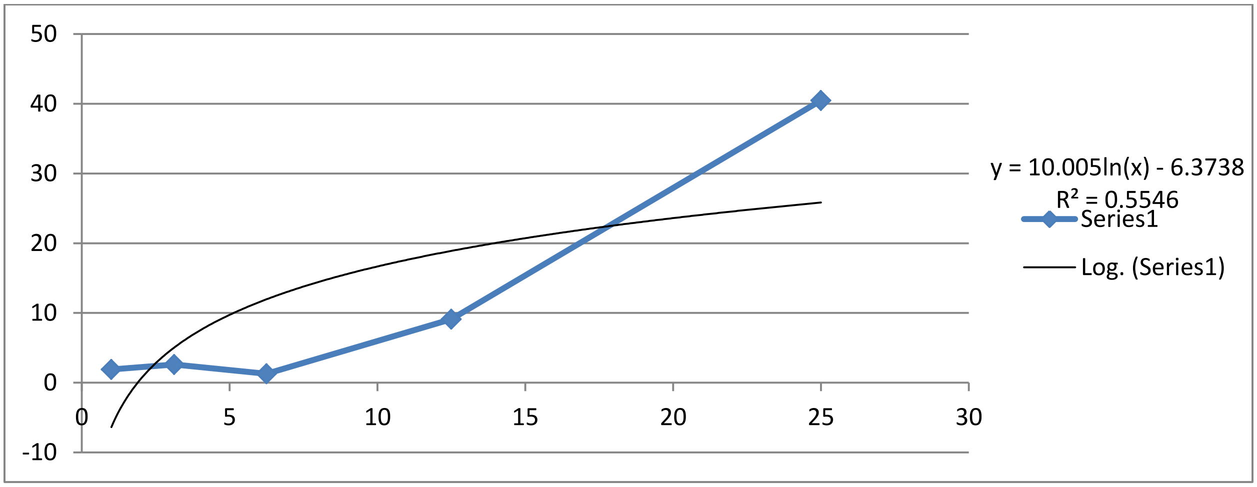

X–axis = concentration (μM) of compound **6**
Y–axis = % Cell Proliferation Inhibition
$IC_{50} > 25$ μM

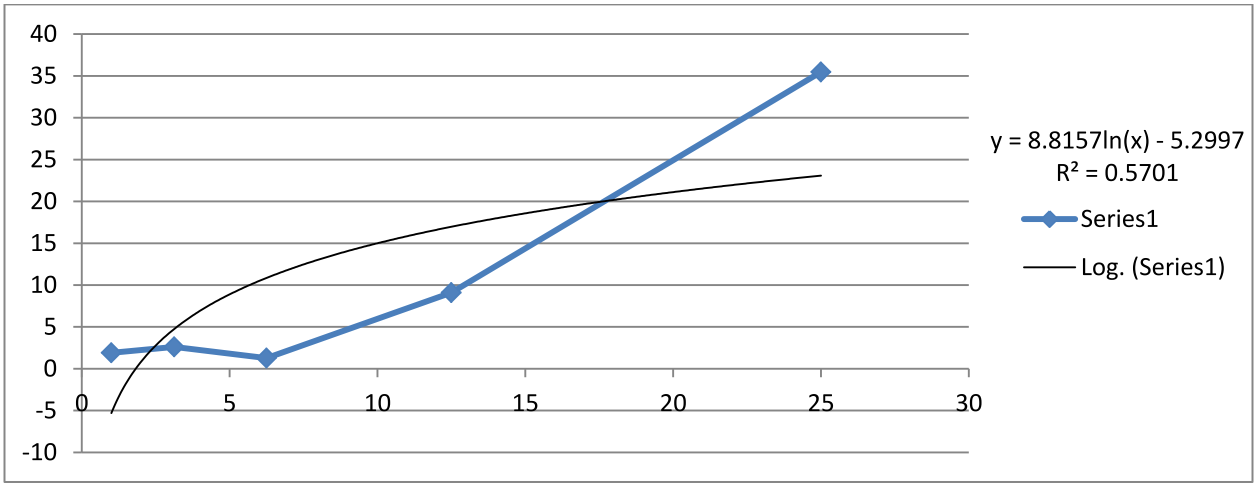

X–axis = concentration (μM) of compound **6**
Y–axis = % Cell Proliferation Inhibition
$IC_{50} > 25$ μM

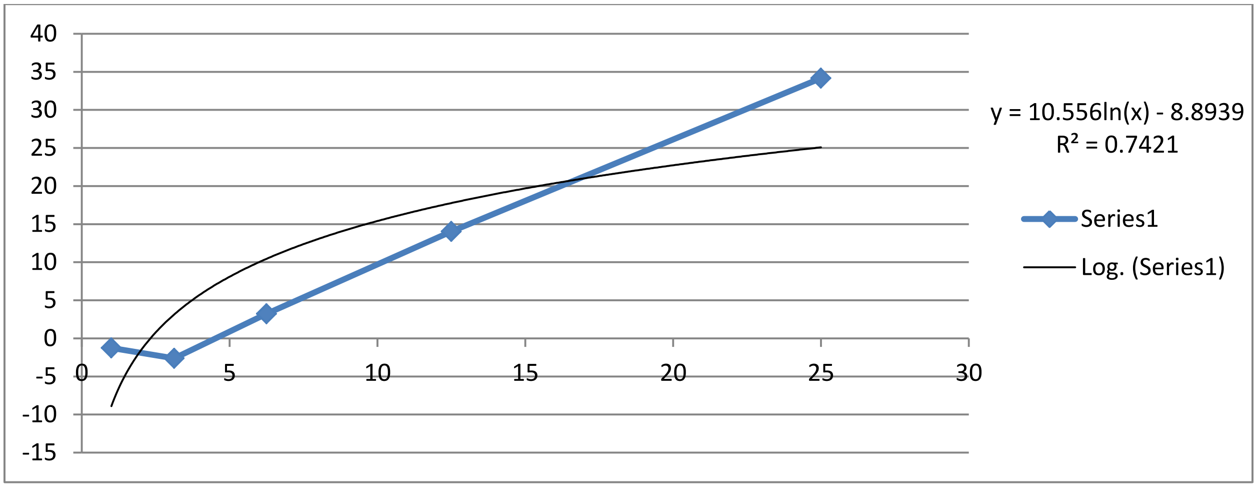

X–axis = concentration (µM) of compound **6**
Y–axis = % Cell Proliferation Inhibition
$IC_{50} > 25$ µM

12. The curves that we used to calculate the IC_{50} value for compound **6** against LNCaP

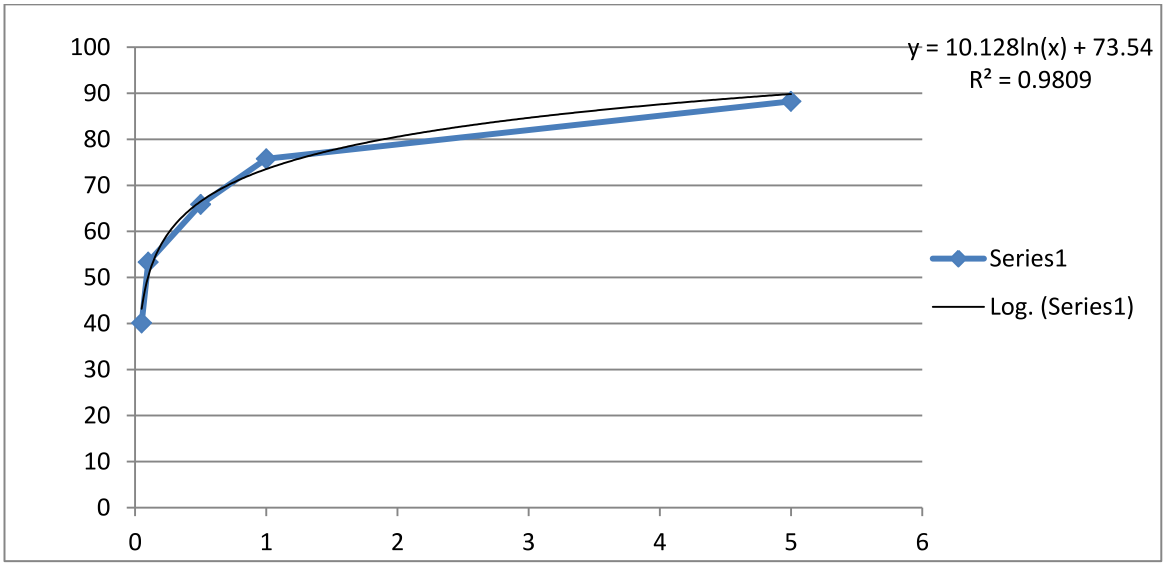

X–axis = concentration (µM) of compound **6**
Y–axis = % Cell Proliferation Inhibition
$IC_{50} = 0.0979$ µM

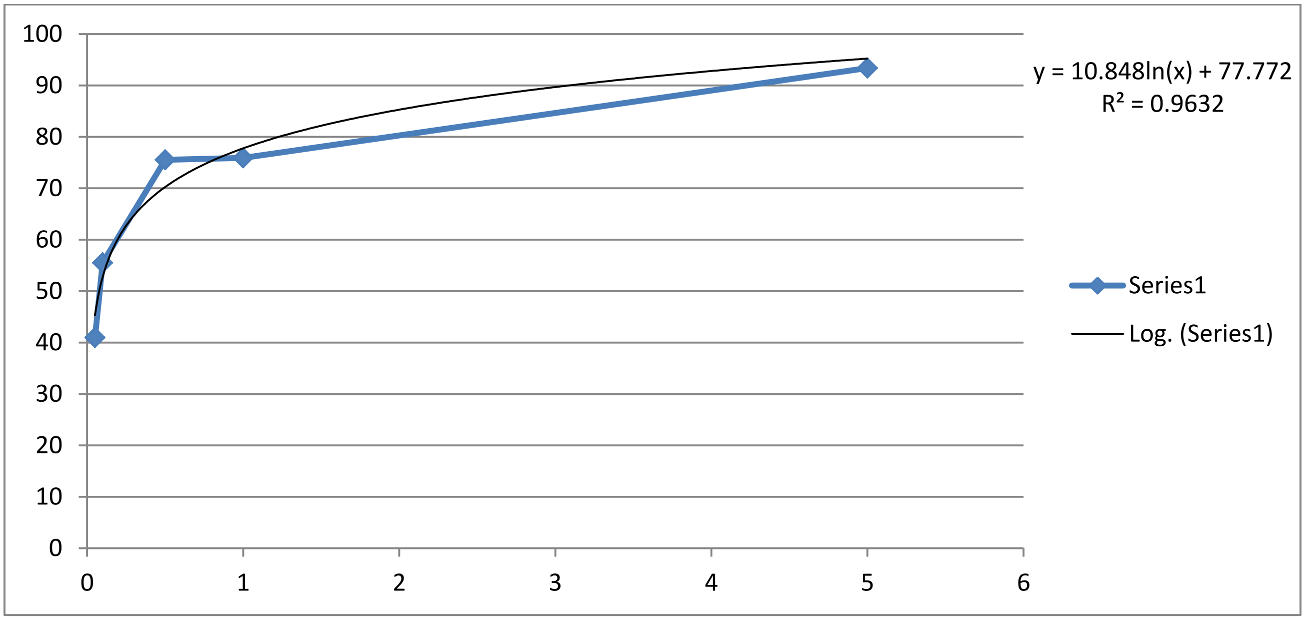

X–axis = concentration (μM) of compound **6**
Y–axis = % Cell Proliferation Inhibition
IC_{50} = 0.0773 μM

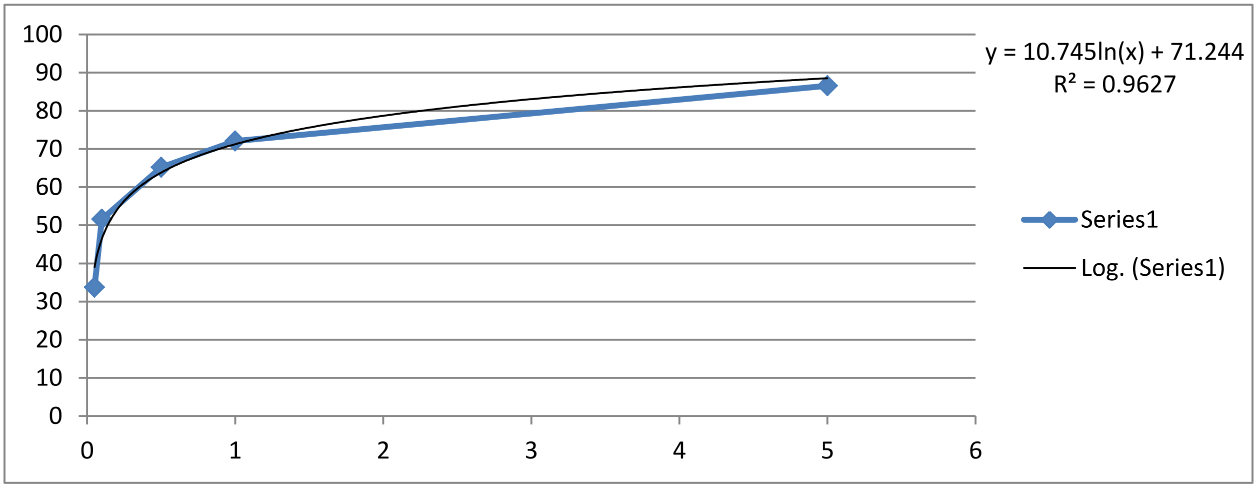

X–axis = concentration (μM) of compound **6**
Y–axis = % Cell Proliferation Inhibition
IC_{50} = 0.1385 μM

13. The curves that we used to calculate the IC_{50} value for compound **7** against PC-3

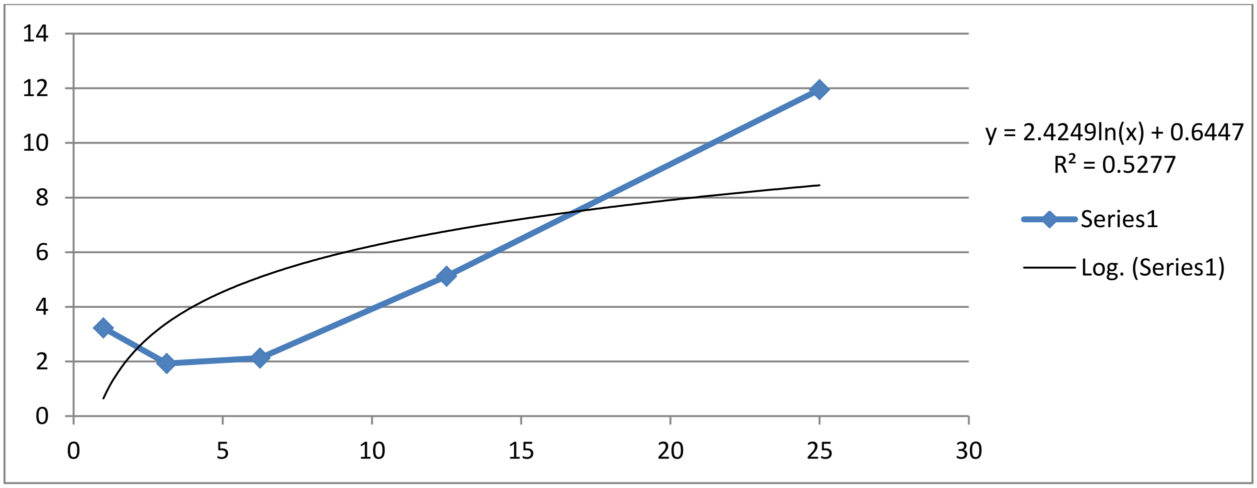

X-axis = concentration (μM) of compound **7**
Y-axis = % Cell Proliferation Inhibition
$IC_{50} > 25$ μM

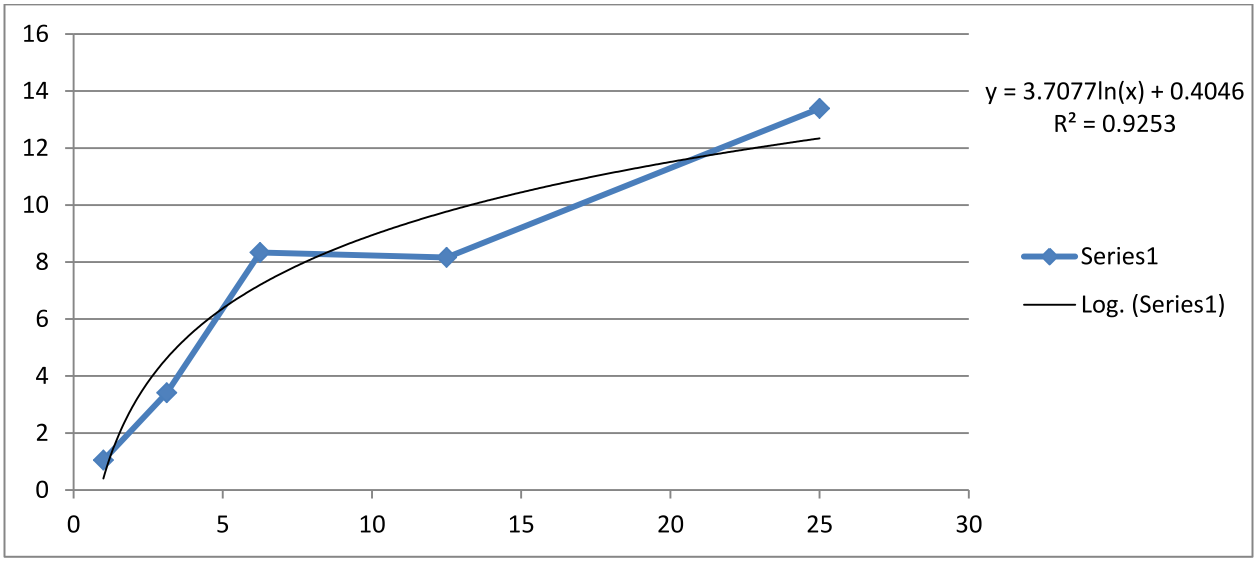

X–axis = concentration (μM) of compound **7**
Y–axis = % Cell Proliferation Inhibition
$IC_{50} > 25$ μM

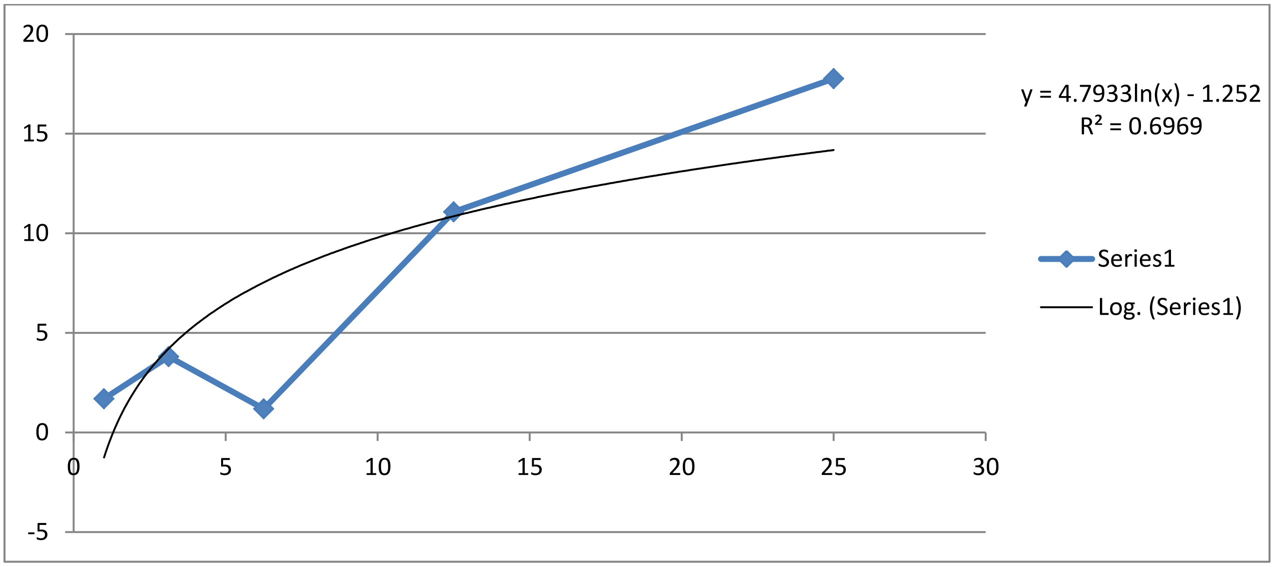

X–axis = concentration (μM) of compound **7**
Y–axis = % Cell Proliferation Inhibition
IC_{50} > 25 μM

14. The curves that we used to calculate the IC_{50} value for compound **7** against DU145

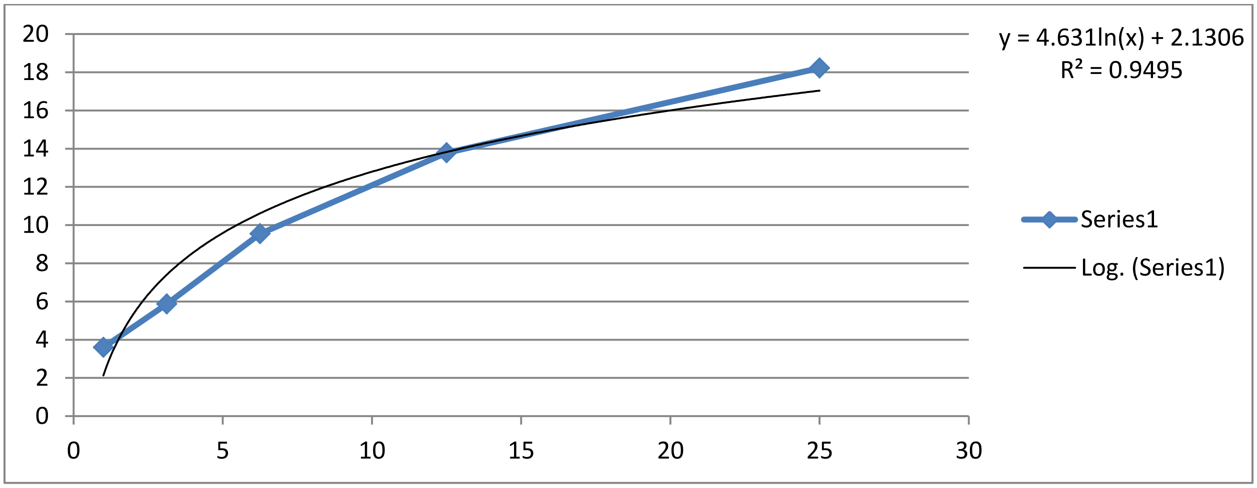

X–axis = concentration (μM) of compound **7**
Y–axis = % Cell Proliferation Inhibition
IC_{50} > 25 μM

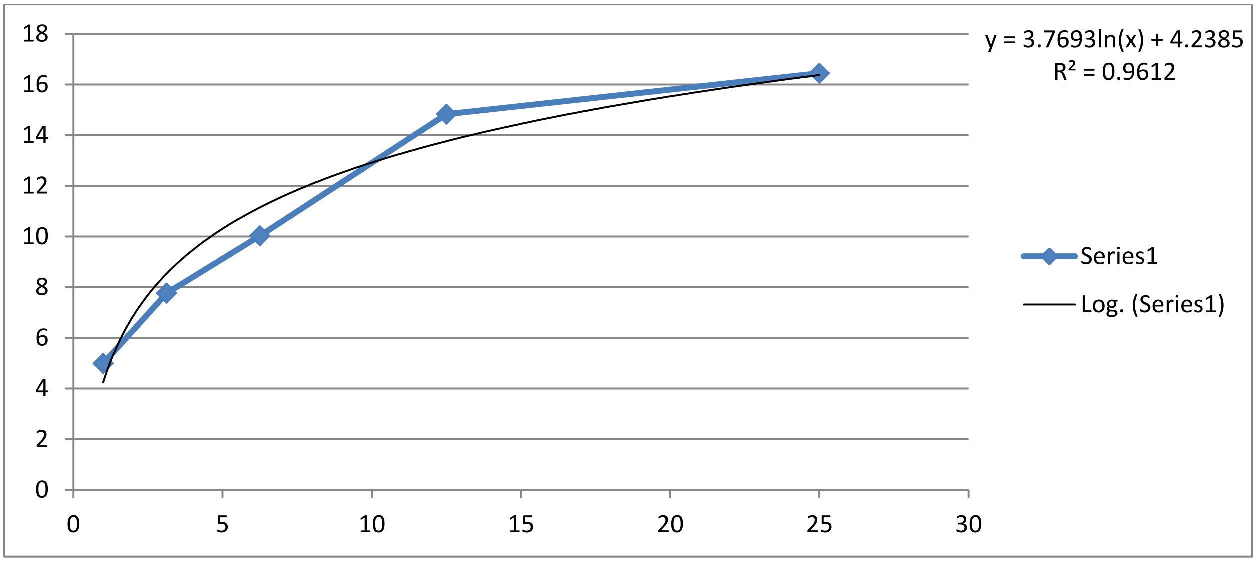

X–axis = concentration (μM) of compound **7**
Y–axis = % Cell Proliferation Inhibition
$IC_{50} > 25$ μM

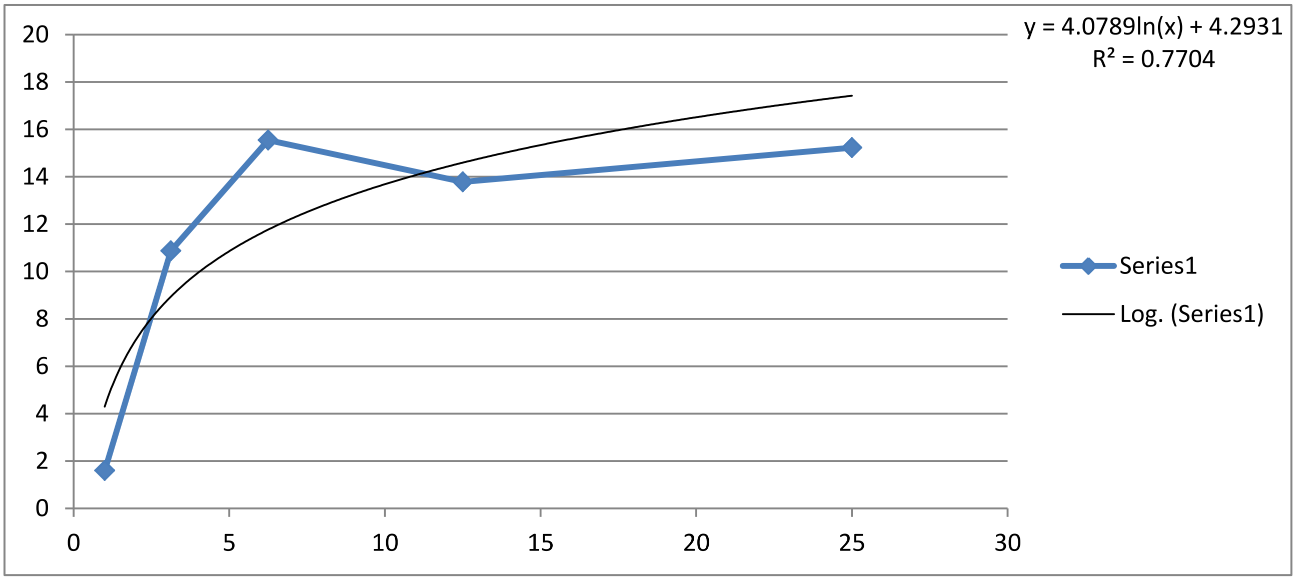

X–axis = concentration (μM) of compound **7**
Y–axis = % Cell Proliferation Inhibition
$IC_{50} > 25$ μM

15. The curves that we used to calculate the IC_{50} value for compound **7** against LNCaP

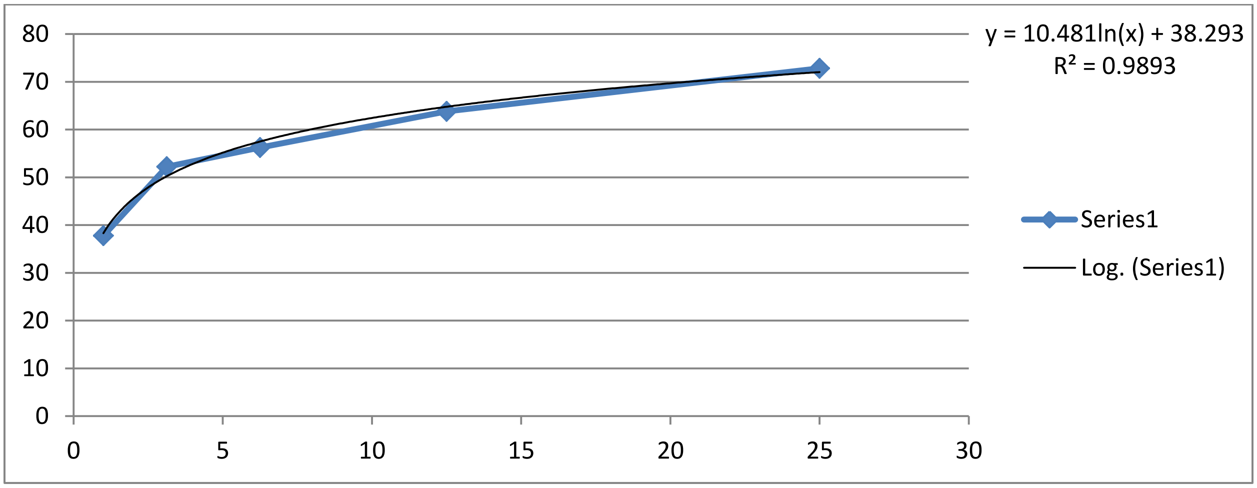

X–axis = concentration (μM) of compound **7**
Y–axis = % Cell Proliferation Inhibition
IC_{50} = 3.055 μM

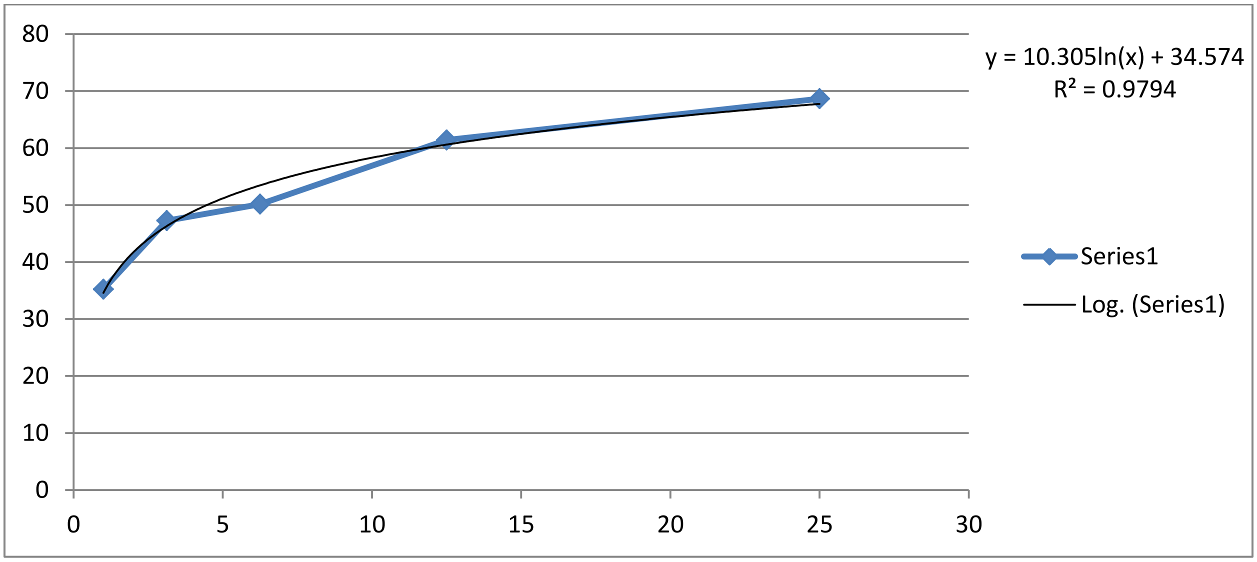

X–axis = concentration (μM) of compound **7**
Y–axis = % Cell Proliferation Inhibition
IC_{50} = 4.468 μM

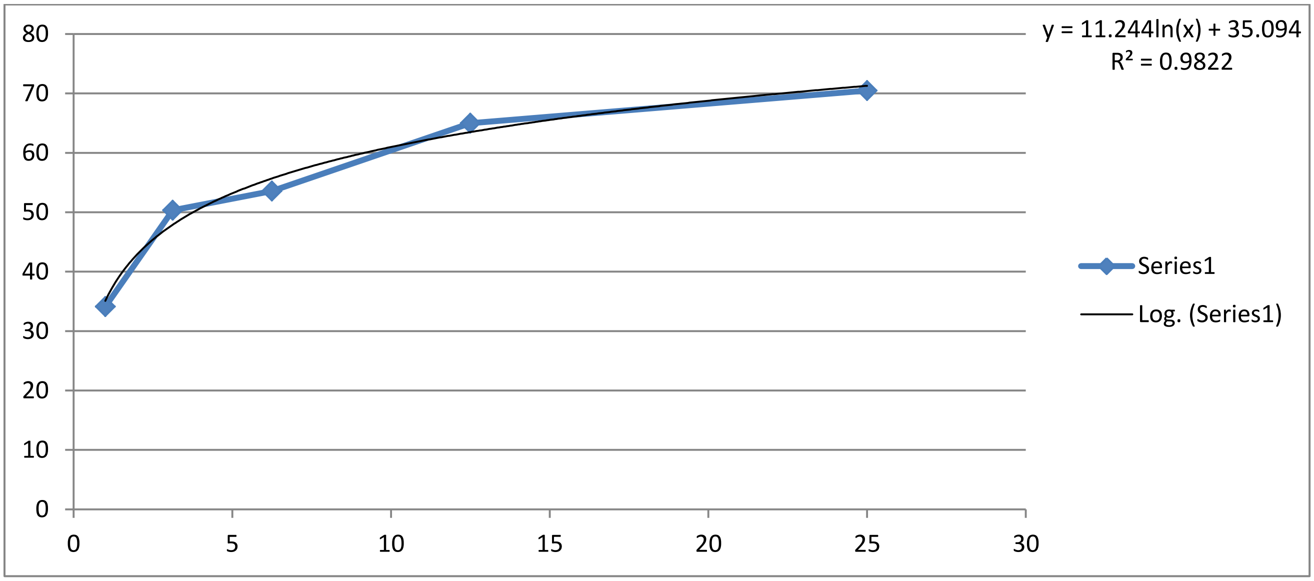

X–axis = concentration (μM) of compound **7**
Y–axis = % Cell Proliferation Inhibition
IC_{50} = 3.765 μM

16. The curves that we used to calculate the IC_{50} value for compound **8** against PC-3

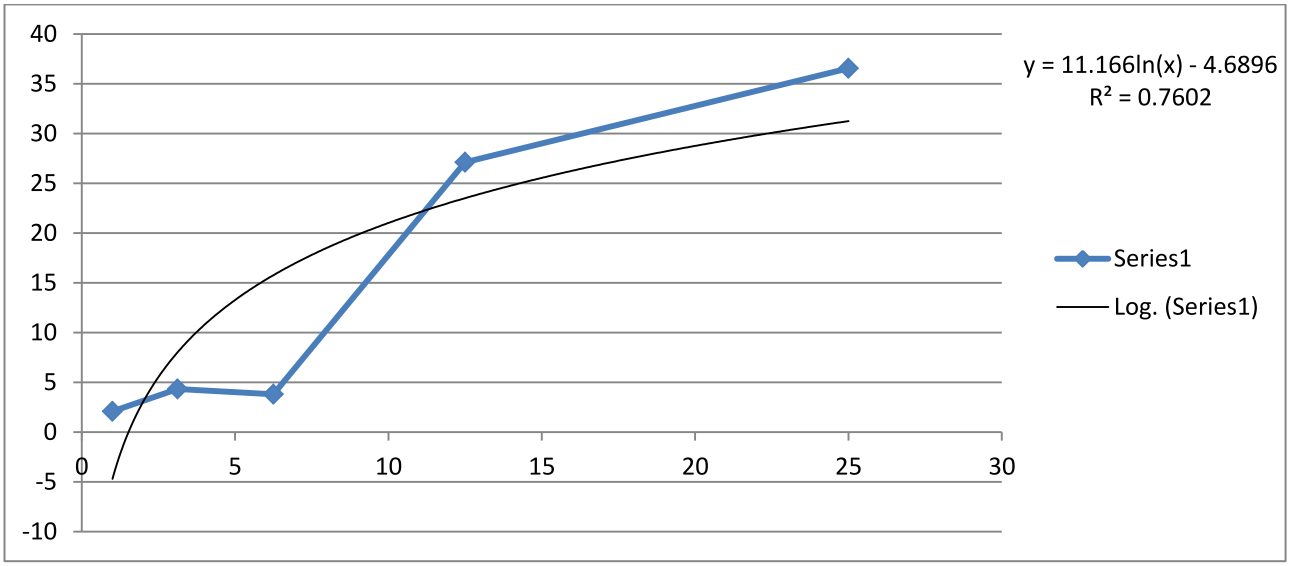

X–axis = concentration (μM) of compound **8**
Y–axis = % Cell Proliferation Inhibition
IC_{50} > 25 μM

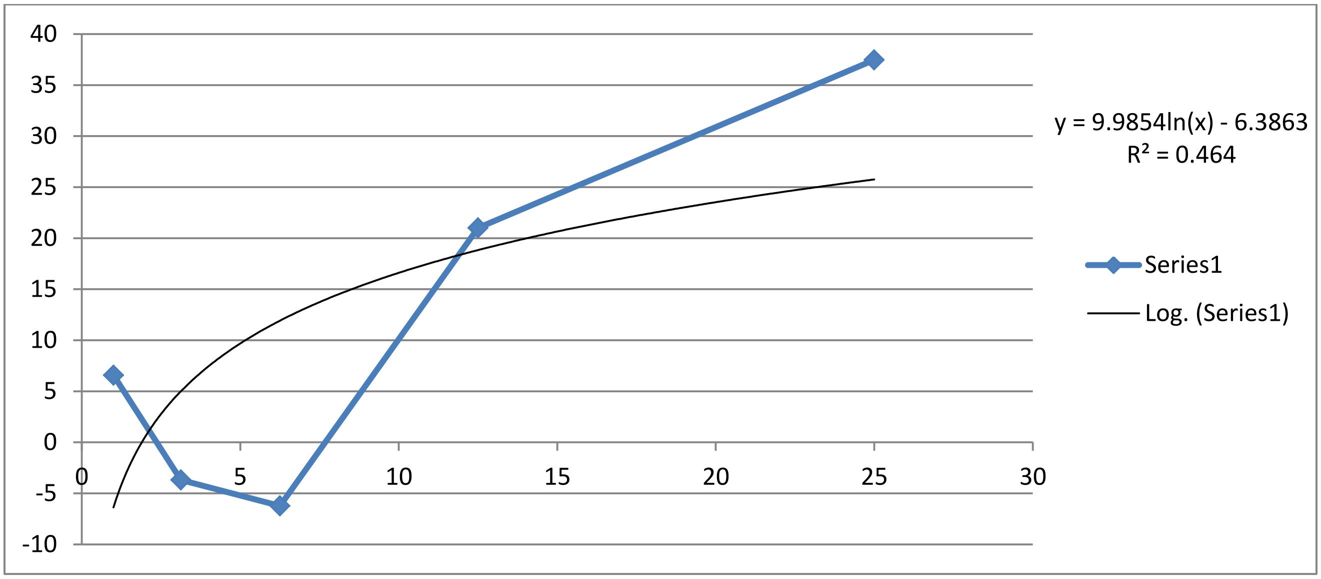

X–axis = concentration (μM) of compound **8**
Y–axis = % Cell Proliferation Inhibition
$IC_{50} > 25$ μM

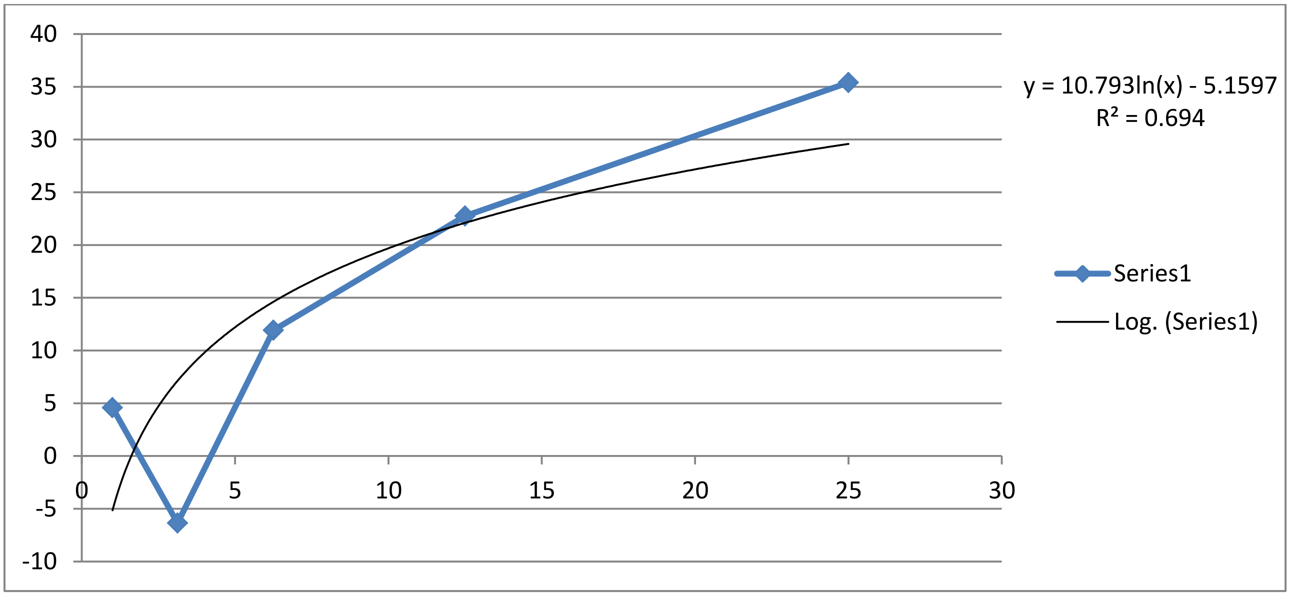

X–axis = concentration (μM) of compound **8**
Y–axis = % Cell Proliferation Inhibition
$IC_{50} > 25$ μM

17. The curves that we used to calculate the IC_{50} value for compound **8** against DU145

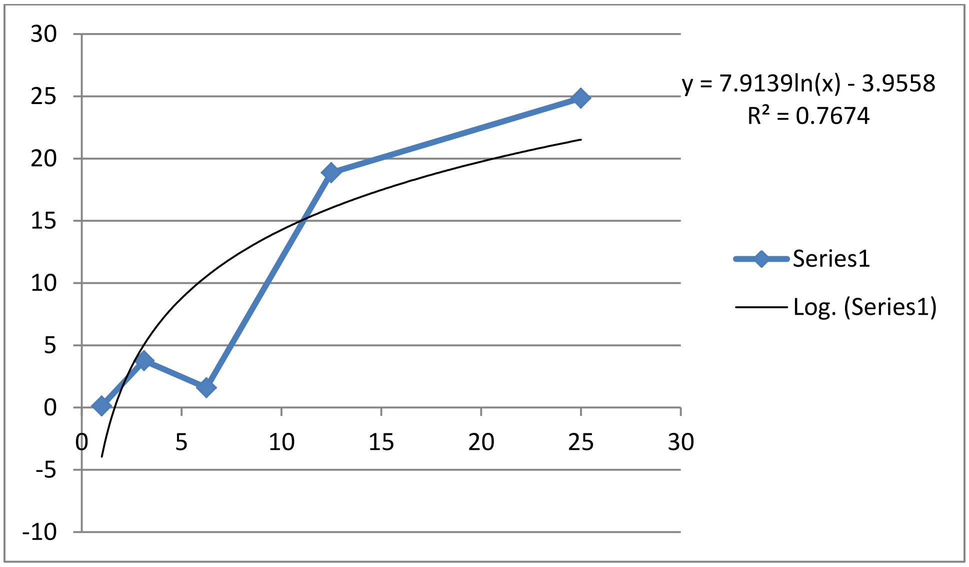

X–axis = concentration (µM) of compound **8**
Y–axis = % Cell Proliferation Inhibition
$IC_{50} > 25$ µM

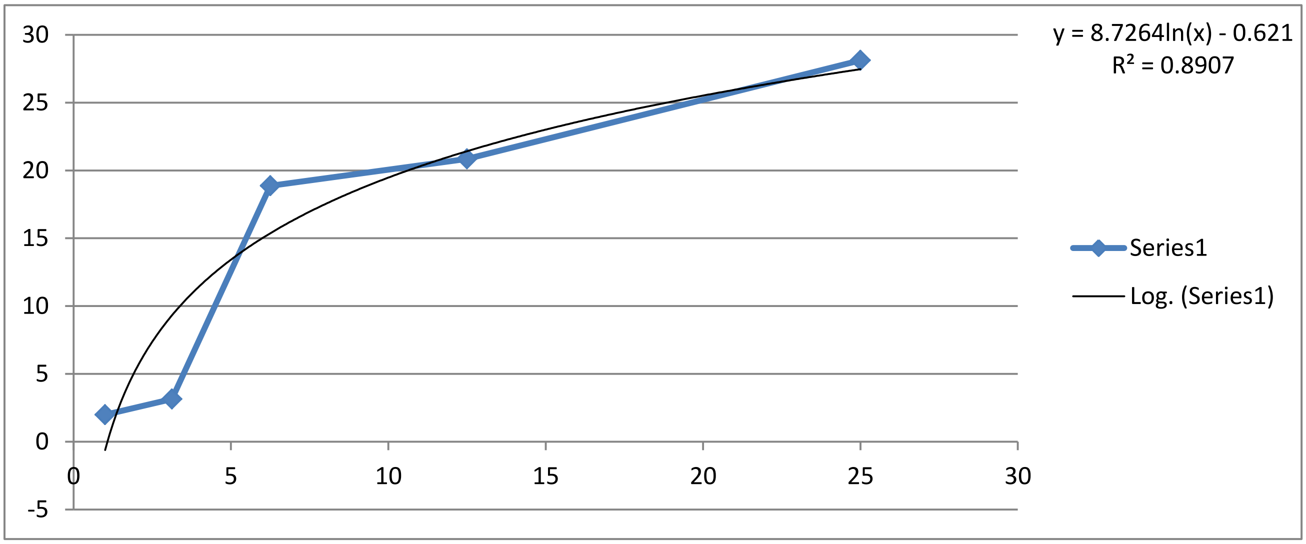

X–axis = concentration (µM) of compound **8**
Y–axis = % Cell Proliferation Inhibition
$IC_{50} > 25$ µM

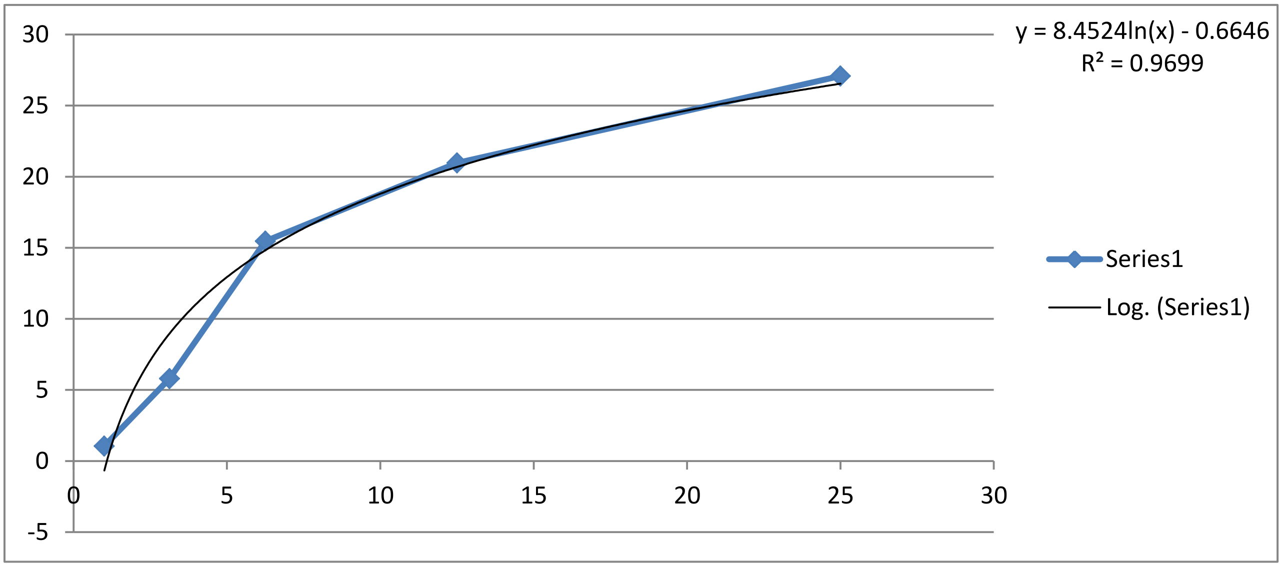

X–axis = concentration (μM) of compound **8**
Y–axis = % Cell Proliferation Inhibition
$IC_{50} > 25$ μM

18. The curves that we used to calculate the IC_{50} value for compound **8** against LNCaP

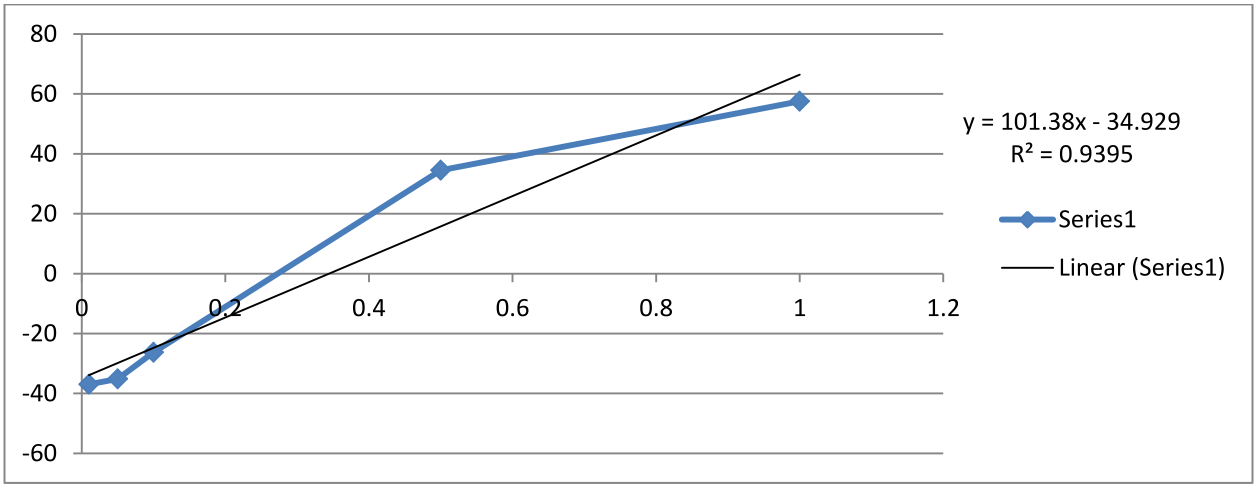

X–axis = concentration (μM) of compound **8**
Y–axis = % Cell Proliferation Inhibition
$IC_{50} = 0.838$ μM

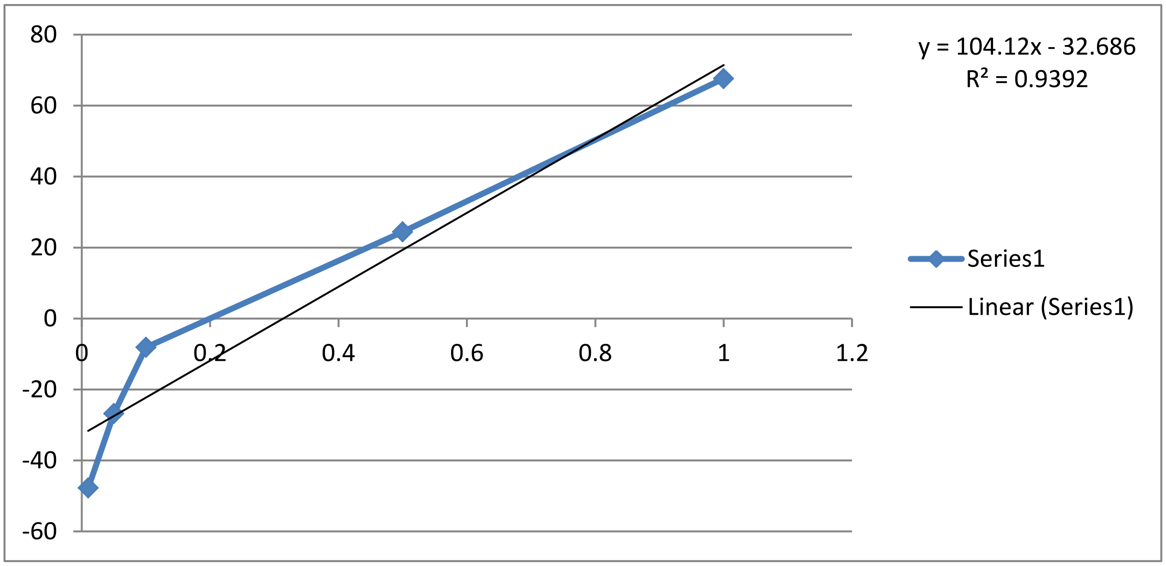

X–axis = concentration (μM) of compound **8**
Y–axis = % Cell Proliferation Inhibition
IC_{50} = 0.794 μM

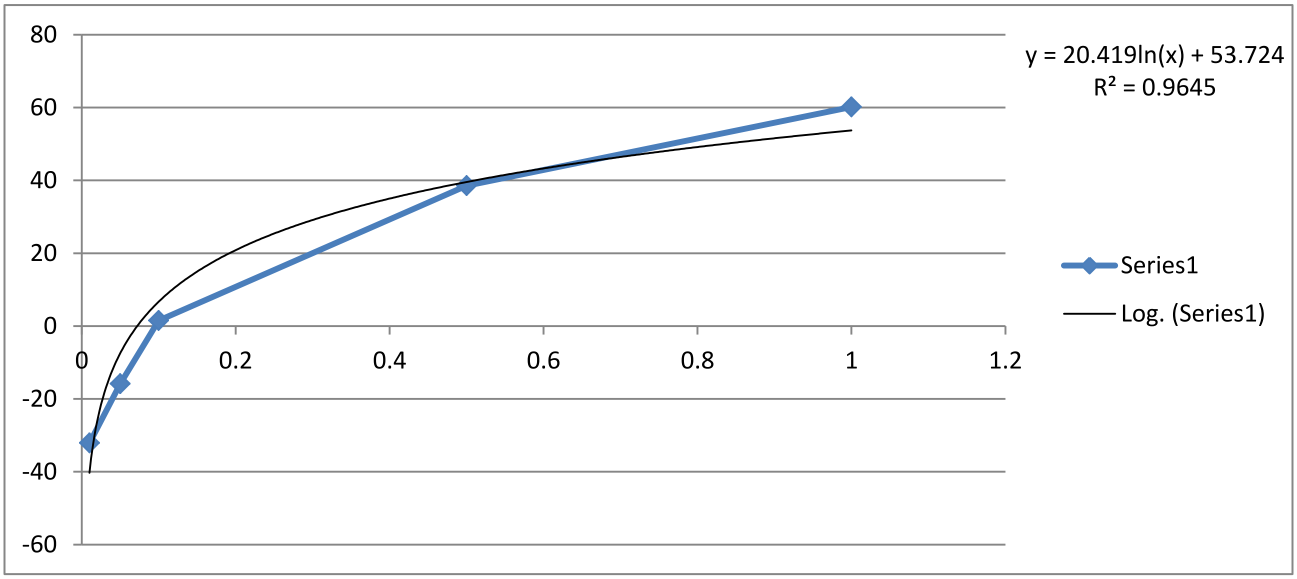

X–axis = concentration (μM) of compound **8**
Y–axis = % Cell Proliferation Inhibition
IC_{50} = 0.833 μM

19. The curves that we used to calculate the IC_{50} value for compound **9** against PC-3

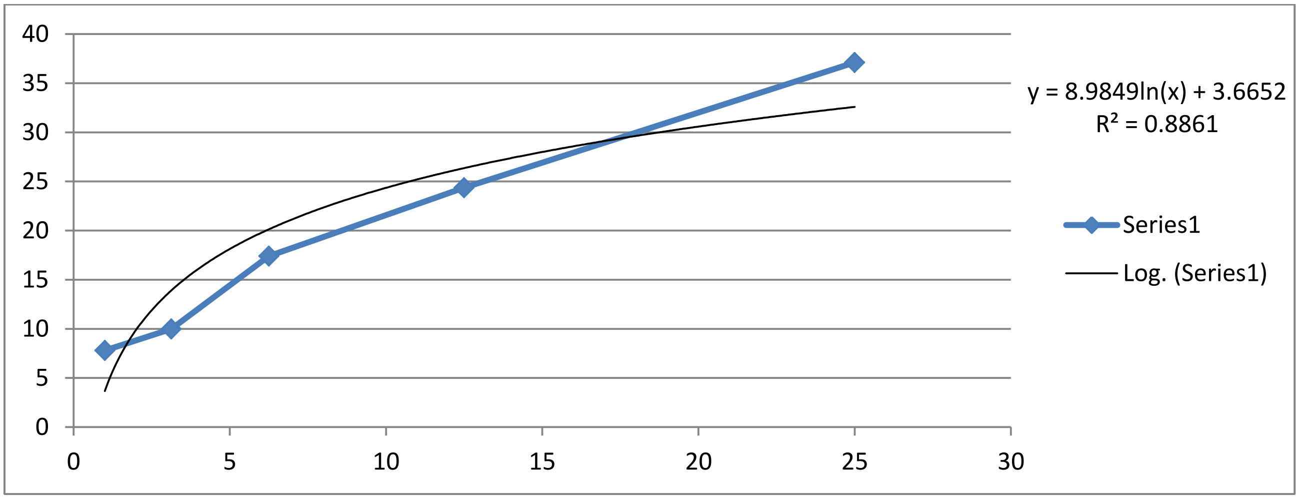

X–axis = concentration (µM) of compound **9**
Y–axis = % Cell Proliferation Inhibition
$IC_{50} > 25$ µM

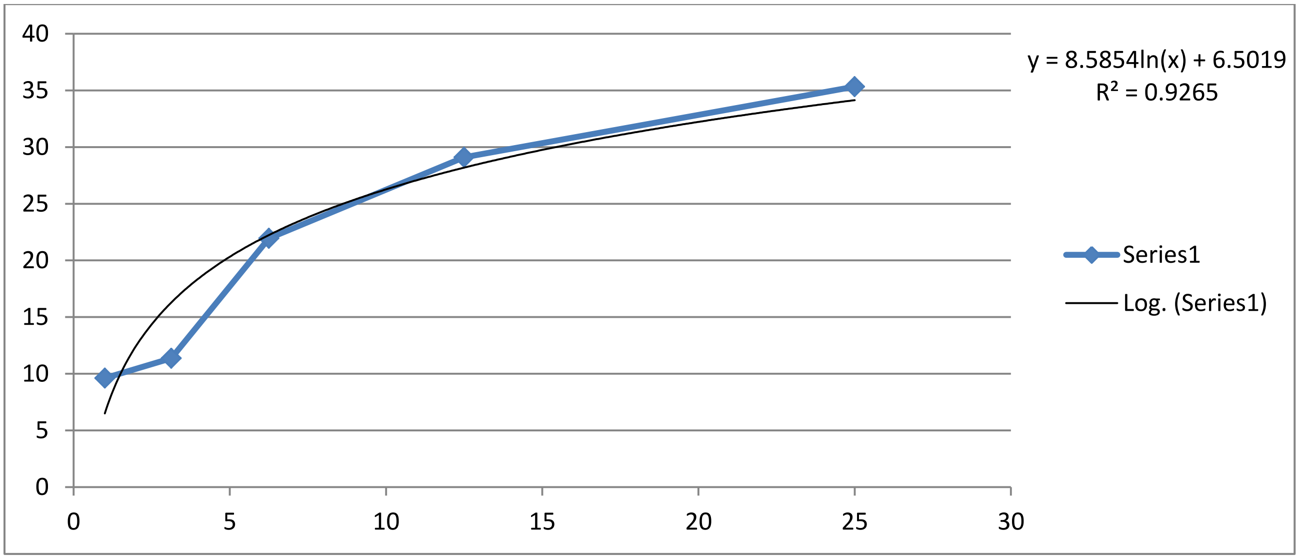

X–axis = concentration (µM) of compound **9**
Y–axis = % Cell Proliferation Inhibition
$IC_{50} > 25$ µM

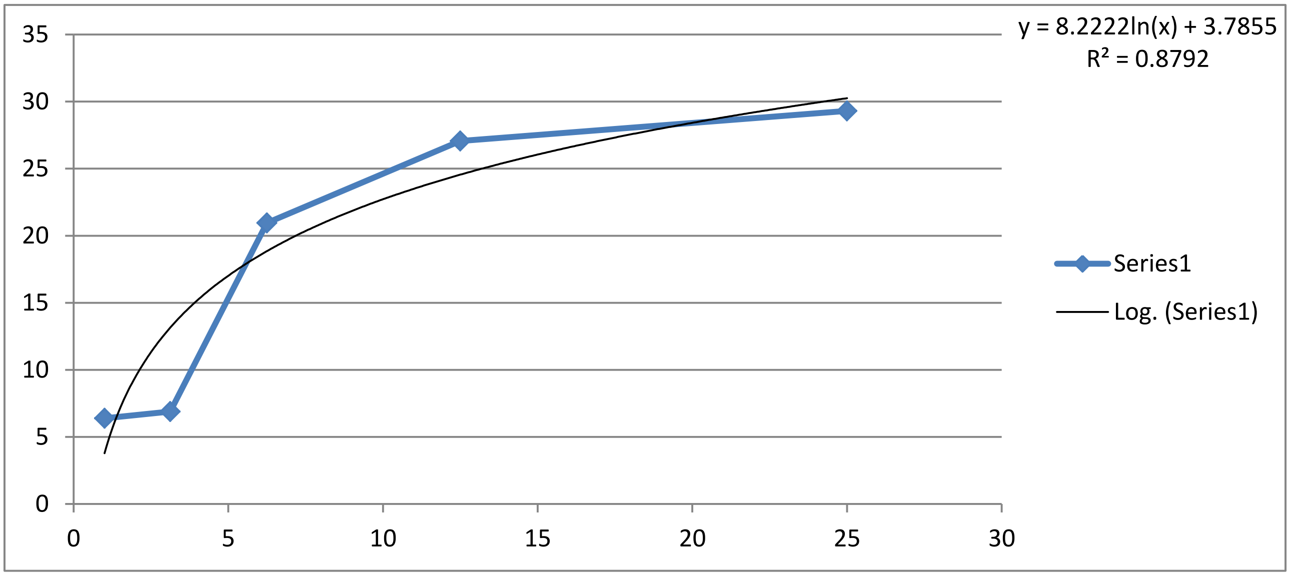

X–axis = concentration (μM) of compound **9**
Y–axis = % Cell Proliferation Inhibition
$IC_{50} > 25$ μM

20. The curves that we used to calculate the IC_{50} value for compound **9** against DU145

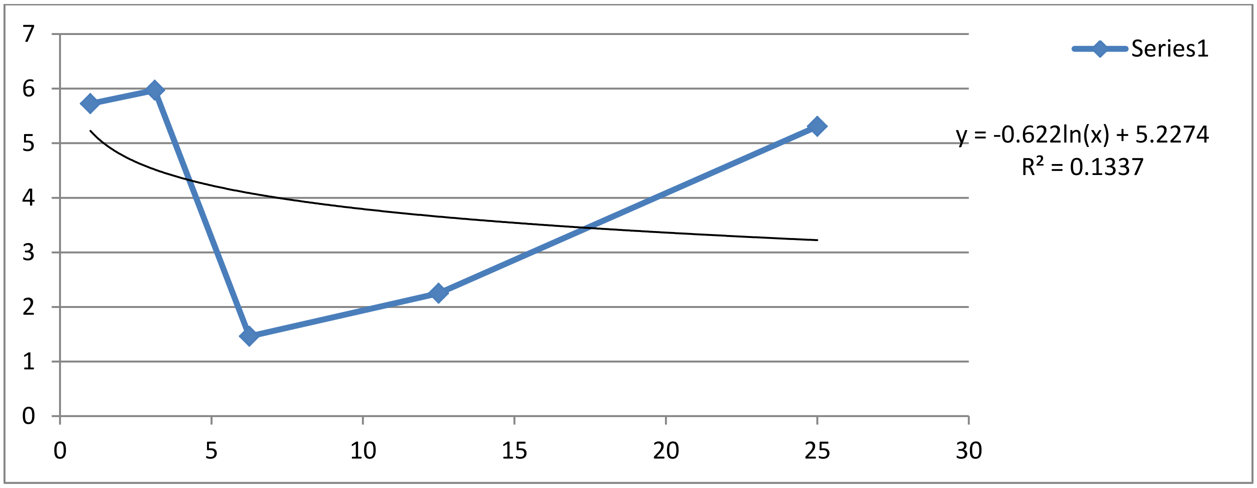

X–axis = concentration (μM) of compound **9**
Y–axis = % Cell Proliferation Inhibition
$IC_{50} > 25$ μM

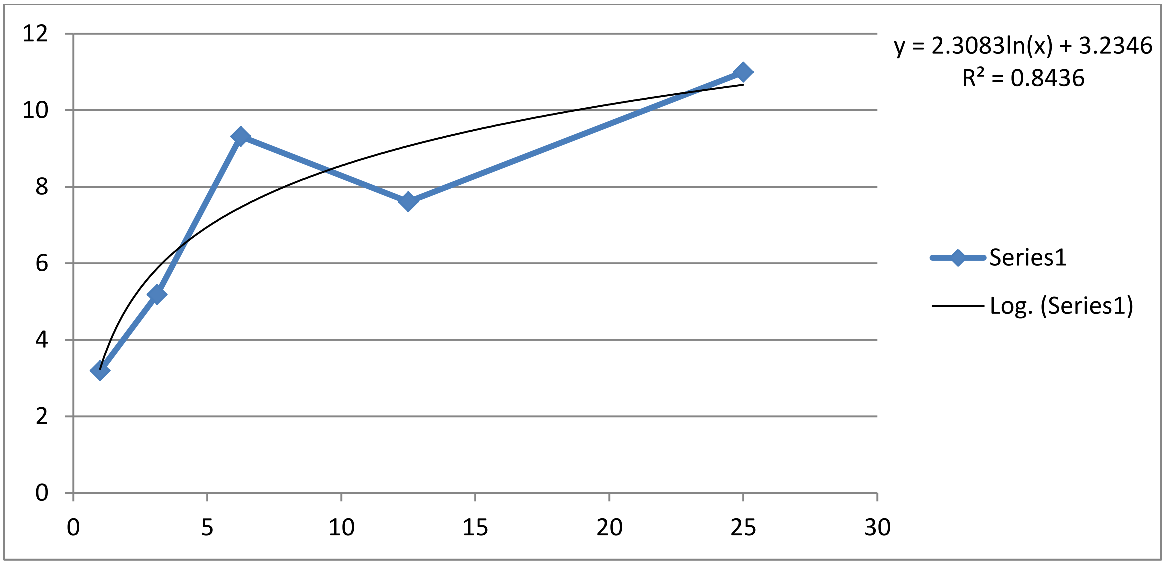

X–axis = concentration (μM) of compound **9**
Y–axis = % Cell Proliferation Inhibition
IC_{50} > 25 μM

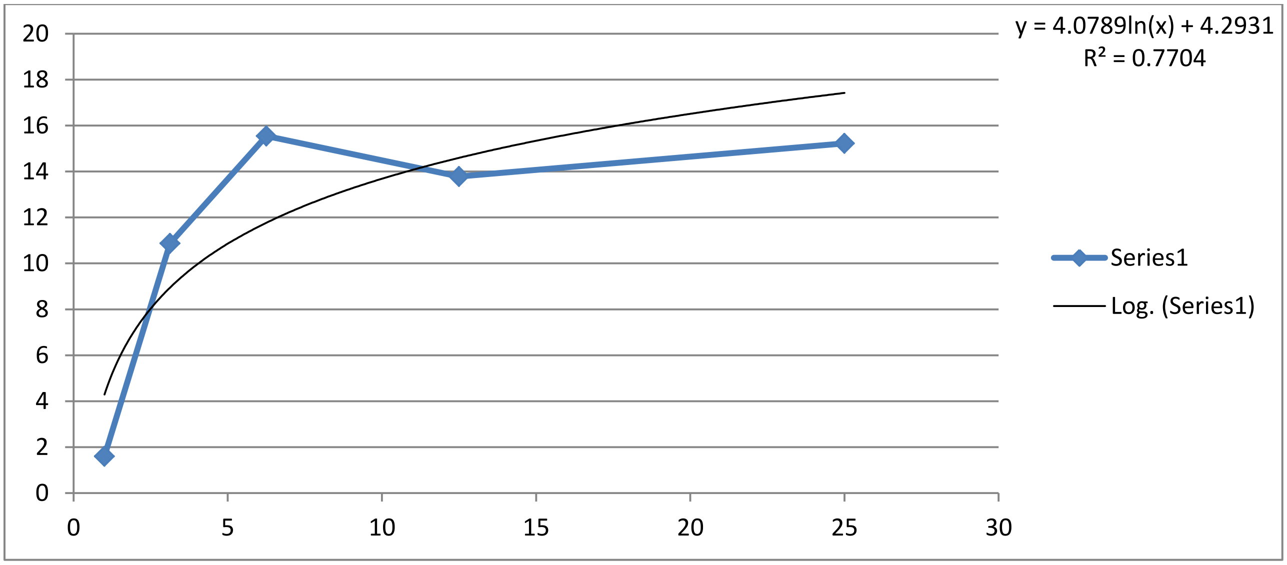

X–axis = concentration (μM) of compound **9**
Y–axis = % Cell Proliferation Inhibition
IC_{50} > 25 μM

21. The curves that we used to calculate the IC_{50} value for compound **9** against LNCaP

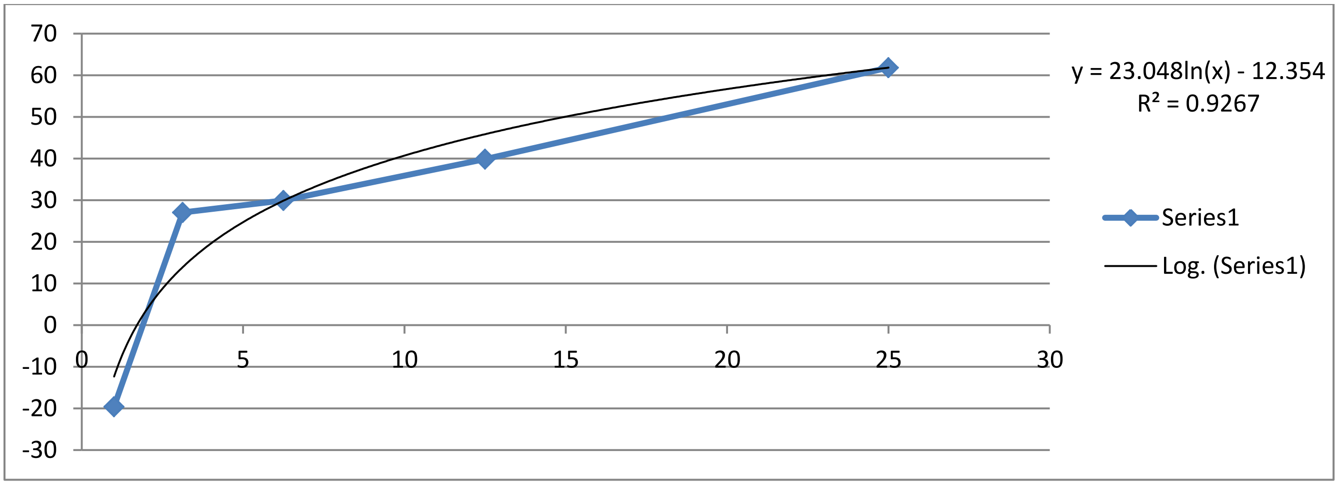

X–axis = concentration (μM) of compound **9**
Y–axis = % Cell Proliferation Inhibition
IC_{50} = 14.960 μM

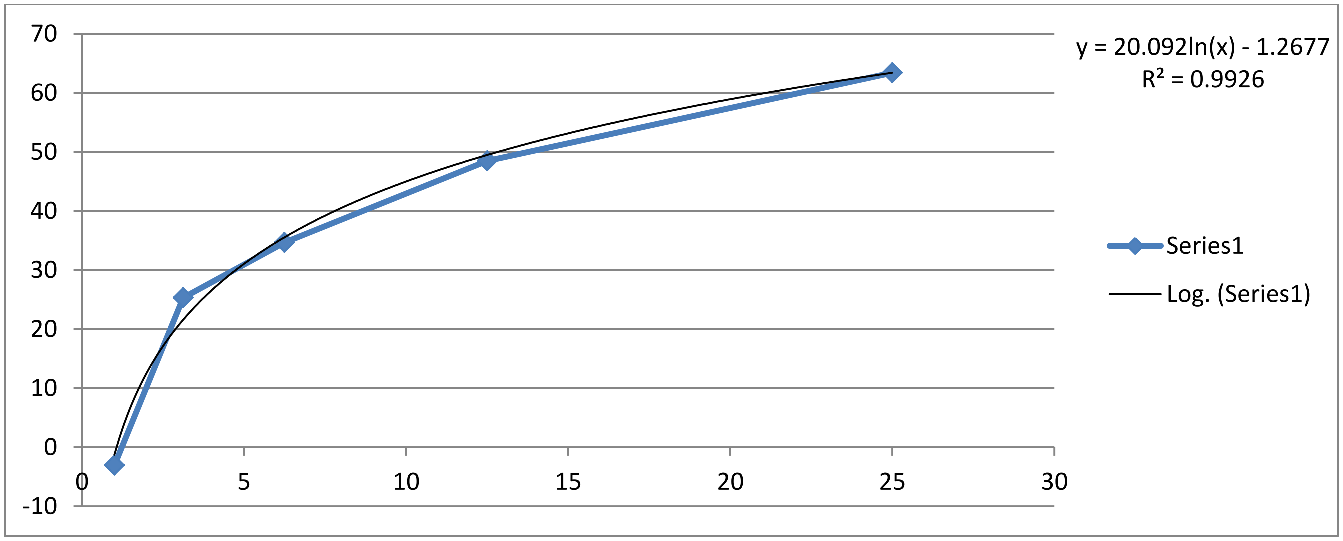

X–axis = concentration (μM) of compound **9**
Y–axis = % Cell Proliferation Inhibition
IC_{50} = 12.828 μM

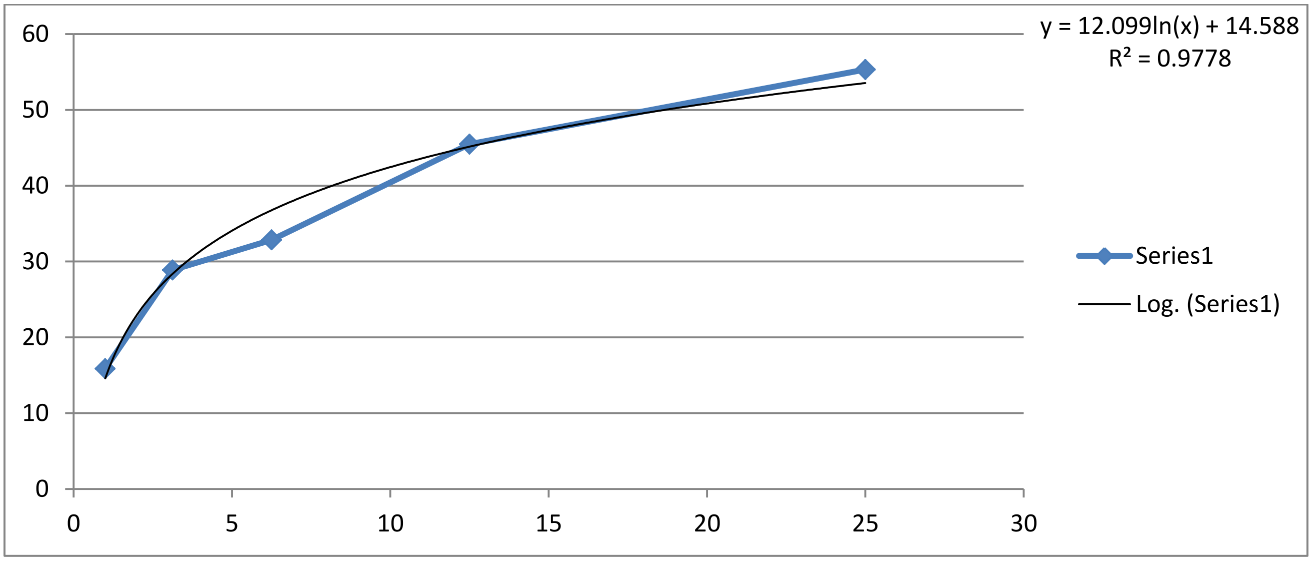

X–axis = concentration (µM) of compound **9**
Y–axis = % Cell Proliferation Inhibition
IC_{50} = 18.669 µM

22. The curves that we used to calculate the IC_{50} value for compound **11** against PC-3

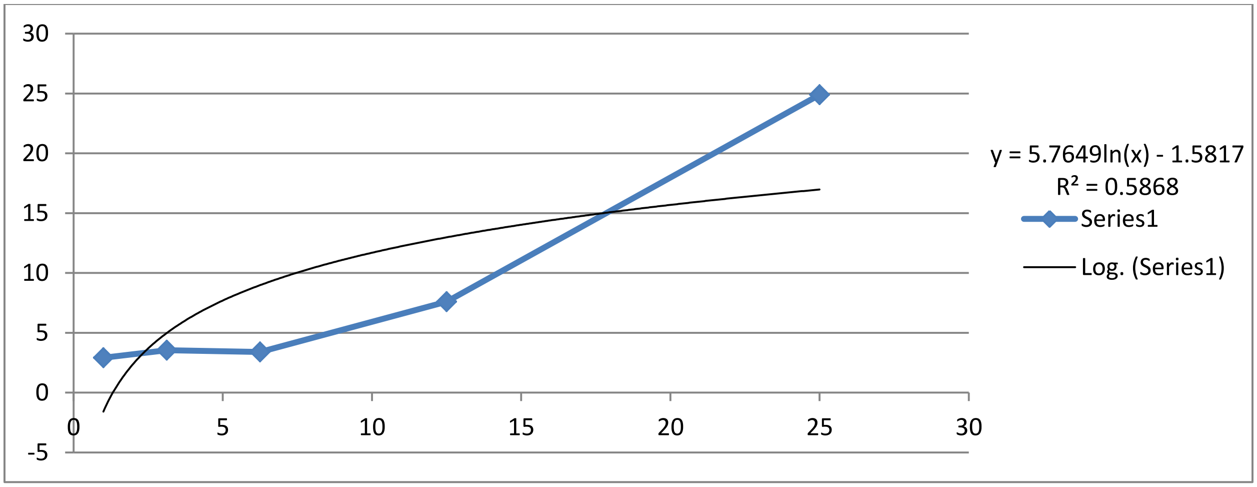

X–axis = concentration (µM) of compound **11**
Y–axis = % Cell Proliferation Inhibition
IC_{50} > 25 µM

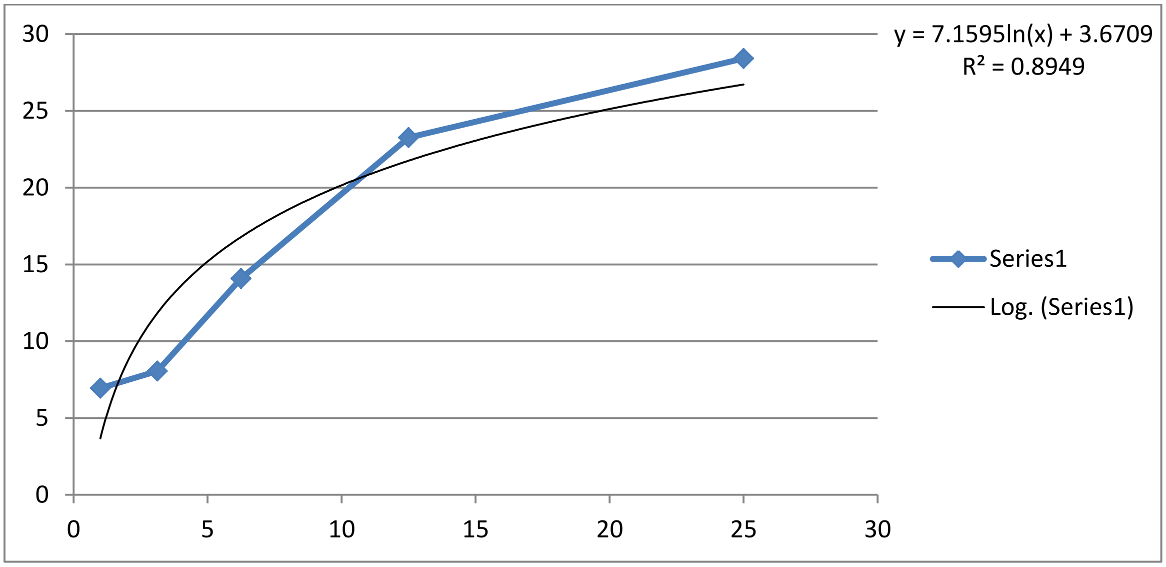

X–axis = concentration (µM) of compound **11**
Y–axis = % Cell Proliferation Inhibition
$IC_{50} > 25$ µM

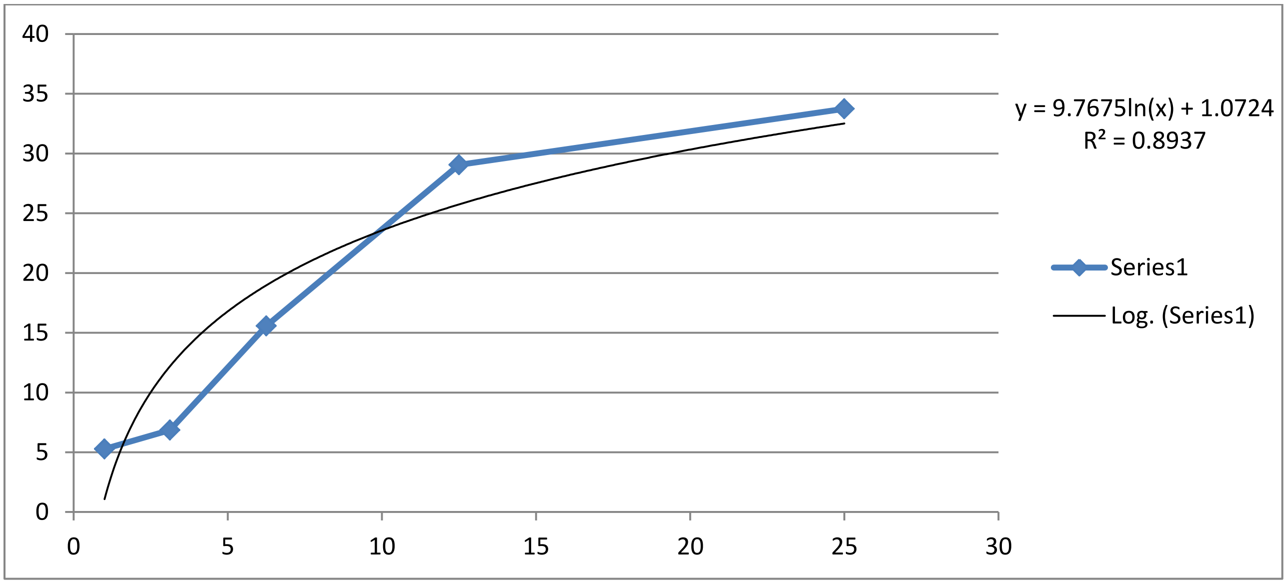

X–axis = concentration (µM) of compound **11**
Y–axis = % Cell Proliferation Inhibition
$IC_{50} > 25$ µM

23. The curves that we used to calculate the IC_{50} value for compound **11** against DU145

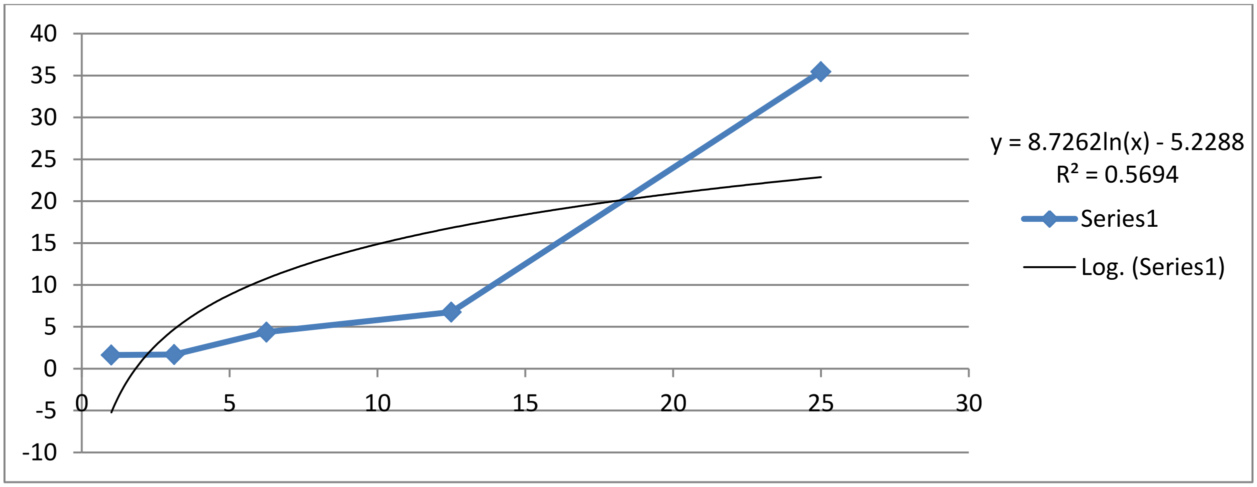

X–axis = concentration (µM) of compound **11**
Y–axis = % Cell Proliferation Inhibition
IC_{50} > 25 µM

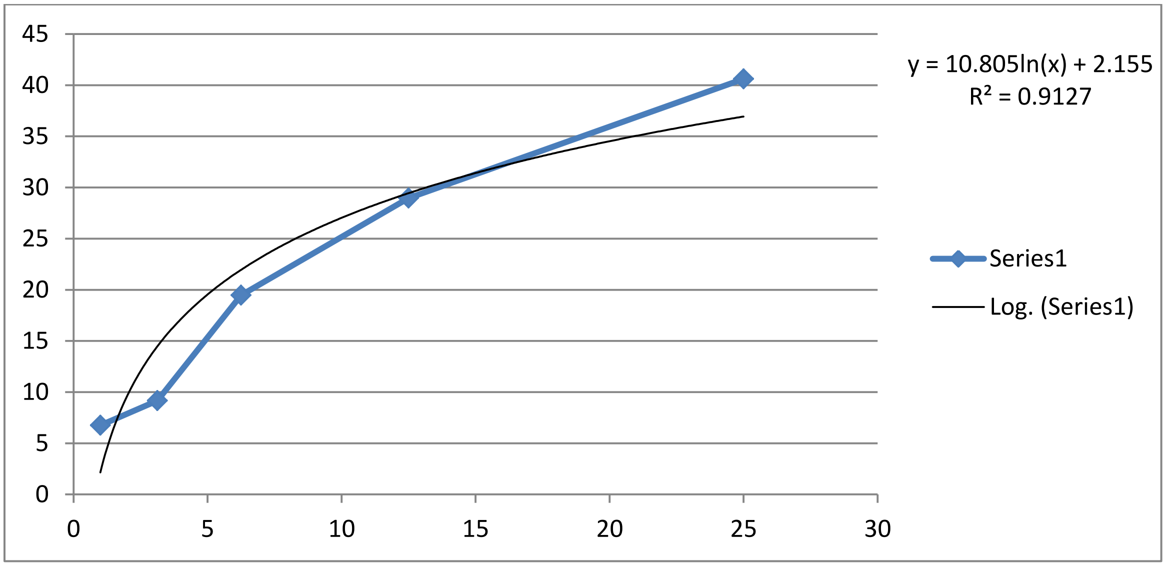

X–axis = concentration (µM) of compound **11**
Y–axis = % Cell Proliferation Inhibition
IC_{50} > 25 µM

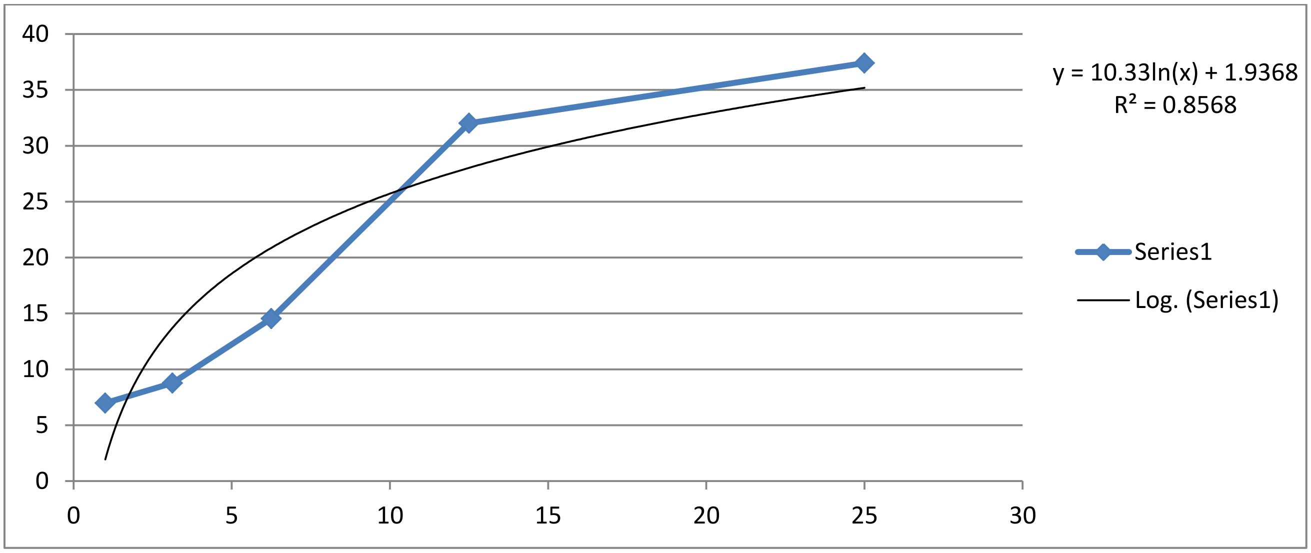

X–axis = concentration (μM) of compound **11**
Y–axis = % Cell Proliferation Inhibition
$IC_{50} > 25$ μM

24. The curves that we used to calculate the IC_{50} value for compound **11** against LNCaP

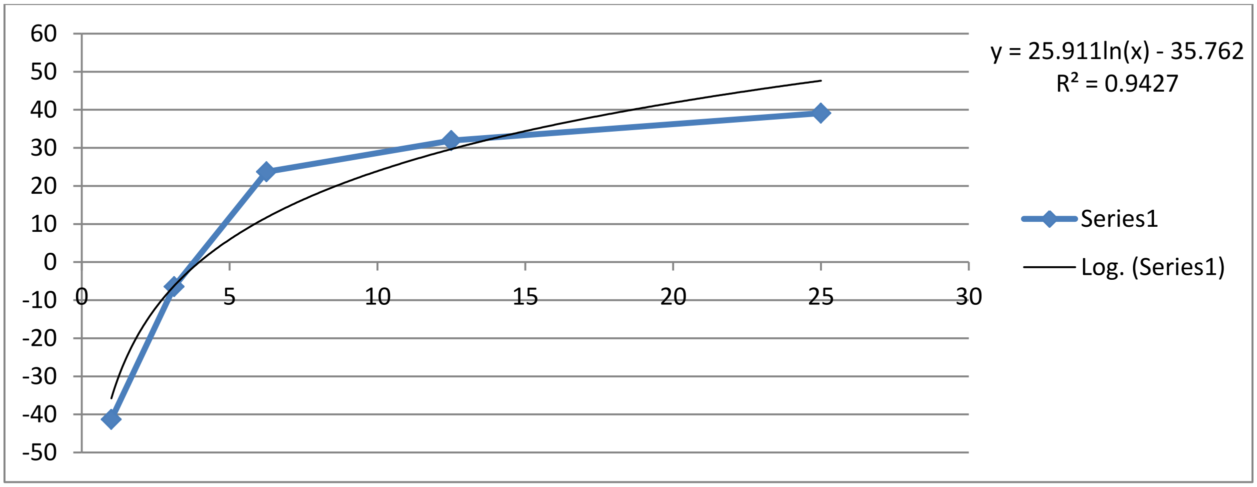

X–axis = concentration (μM) of compound **11**
Y–axis = % Cell Proliferation Inhibition
$IC_{50} = 27.381$ μM

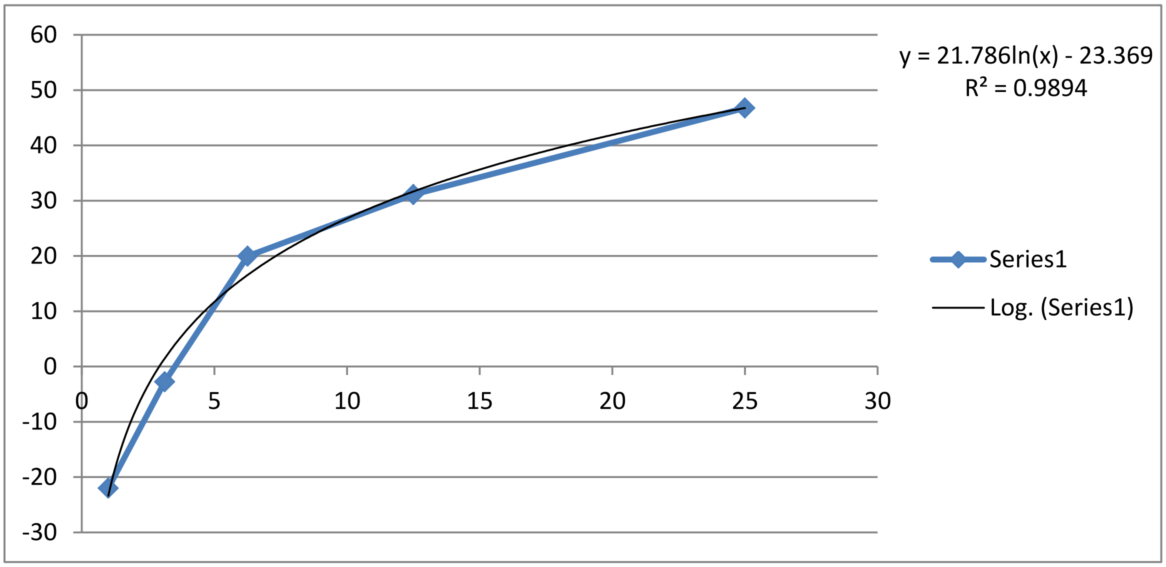

X–axis = concentration (µM) of compound **11**
Y–axis = % Cell Proliferation Inhibition
IC_{50} = 29.012 µM

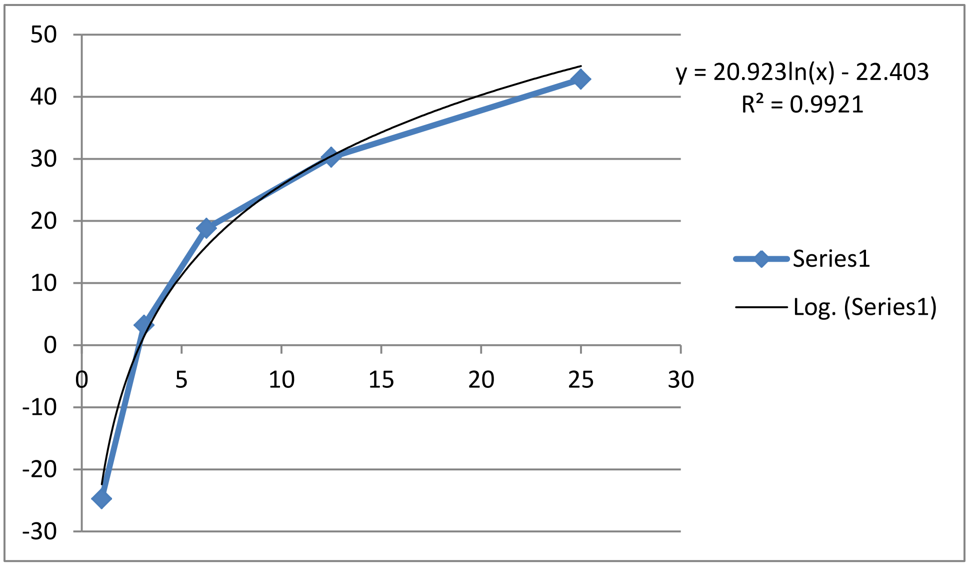

X–axis = concentration (µM) of compound **11**
Y–axis = % Cell Proliferation Inhibition
IC_{50} = 31.831 µM

25. The curves that we used to calculate the IC_{50} value for compound **12** against PC-3

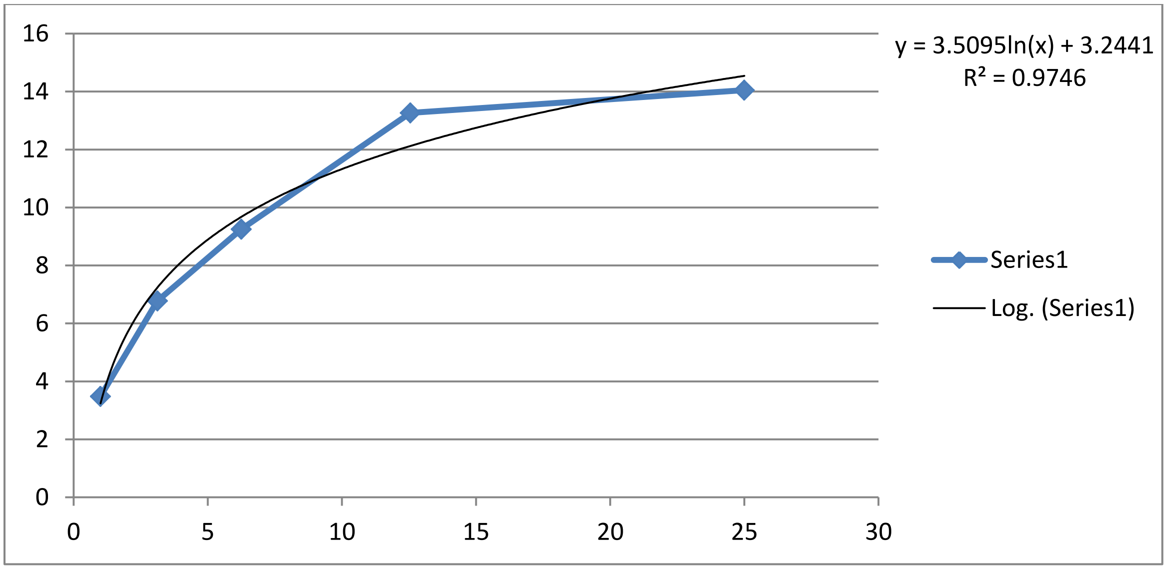

X–axis = concentration (µM) of compound **12**
Y–axis = % Cell Proliferation Inhibition
$IC_{50} > 25$ µM

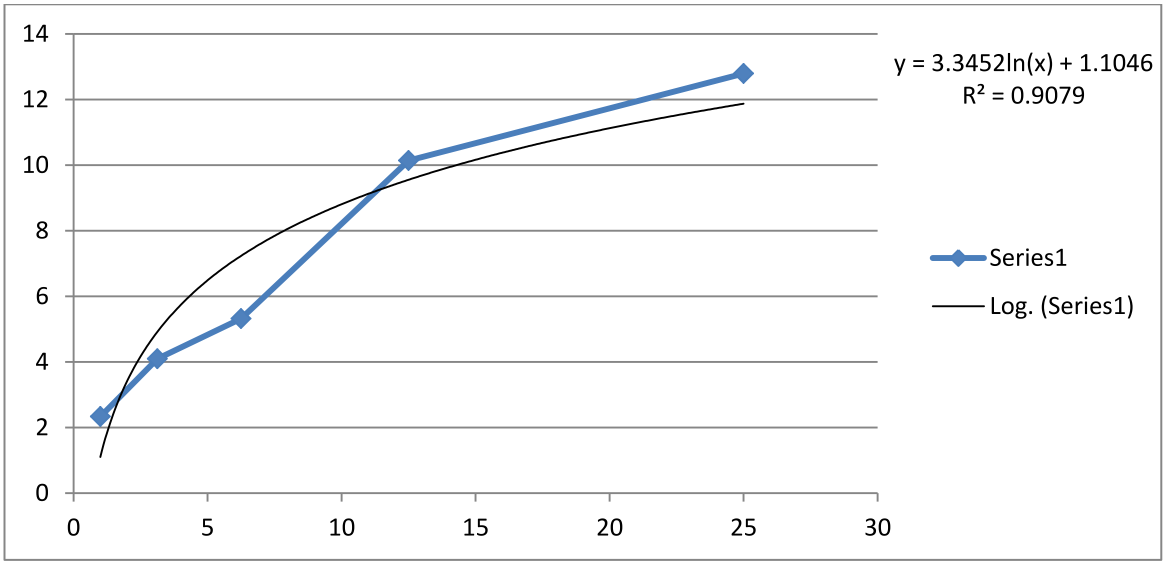

X–axis = concentration (µM) of compound **12**
Y–axis = % Cell Proliferation Inhibition
$IC_{50} > 25$ µM

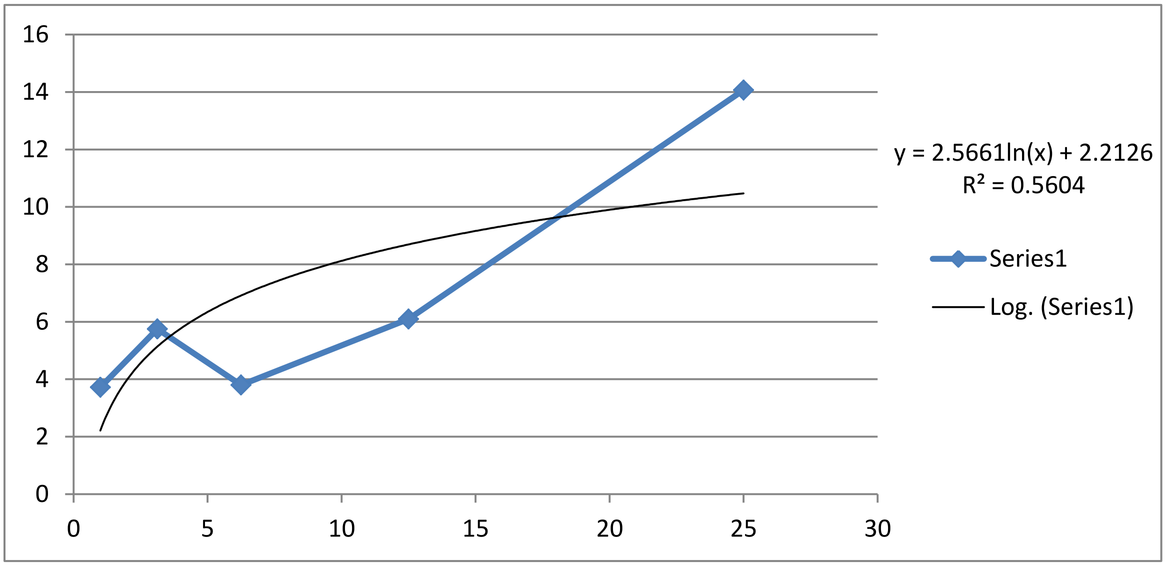

X–axis = concentration (µM) of compound **12**
Y–axis = % Cell Proliferation Inhibition
IC_{50} > 25 µM

26. The curves that we used to calculate the IC_{50} value for compound **12** against DU145

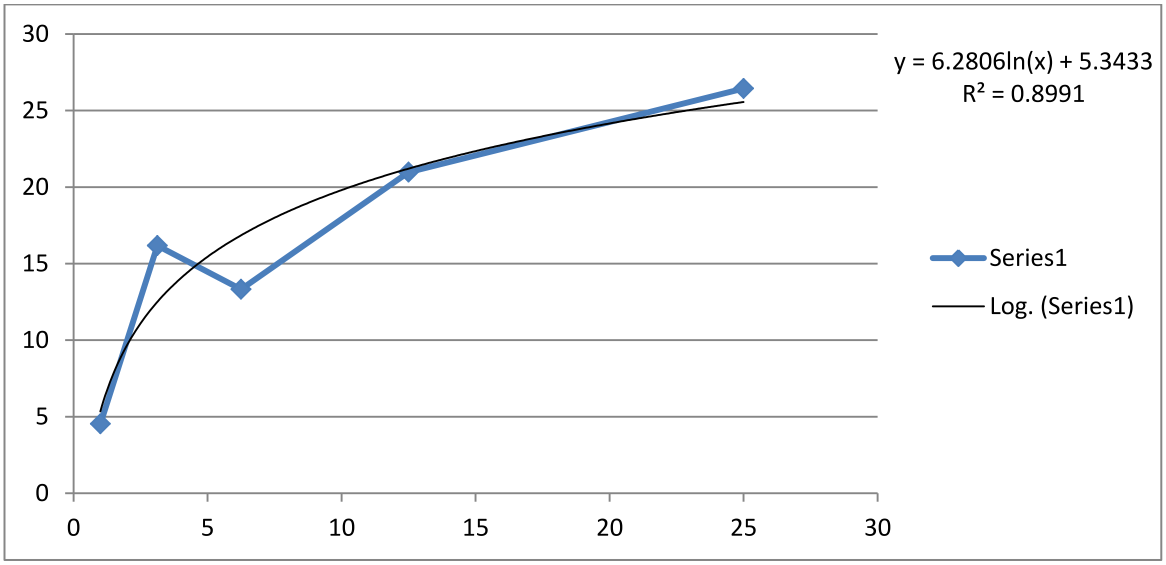

X–axis = concentration (µM) of compound **12**
Y–axis = % Cell Proliferation Inhibition
IC_{50} > 25 µM

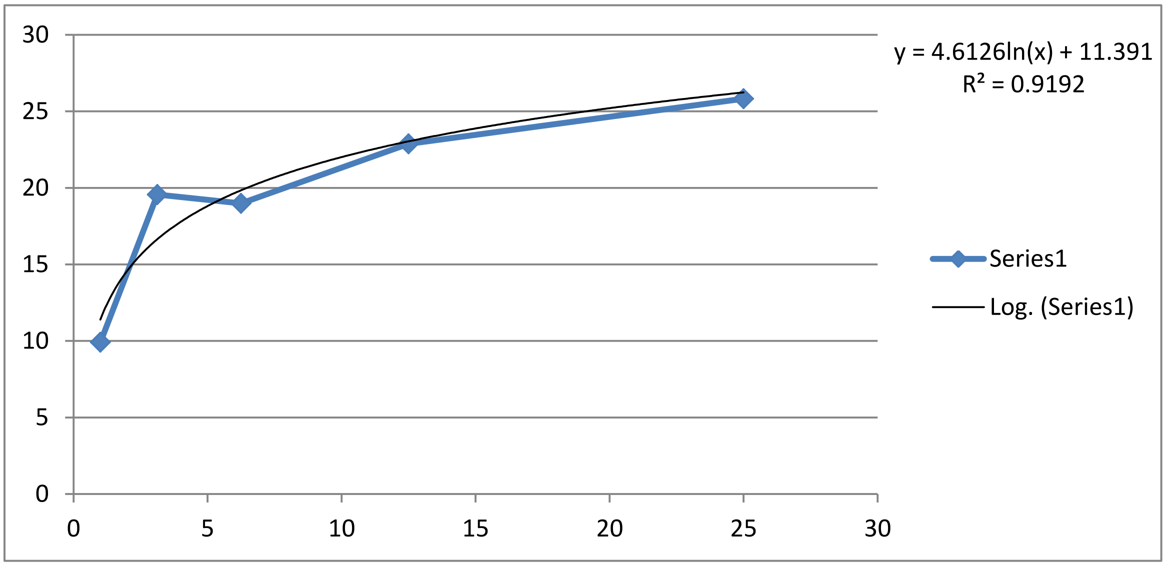

X–axis = concentration (μM) of compound **12**
Y–axis = % Cell Proliferation Inhibition
$IC_{50} > 25$ μM

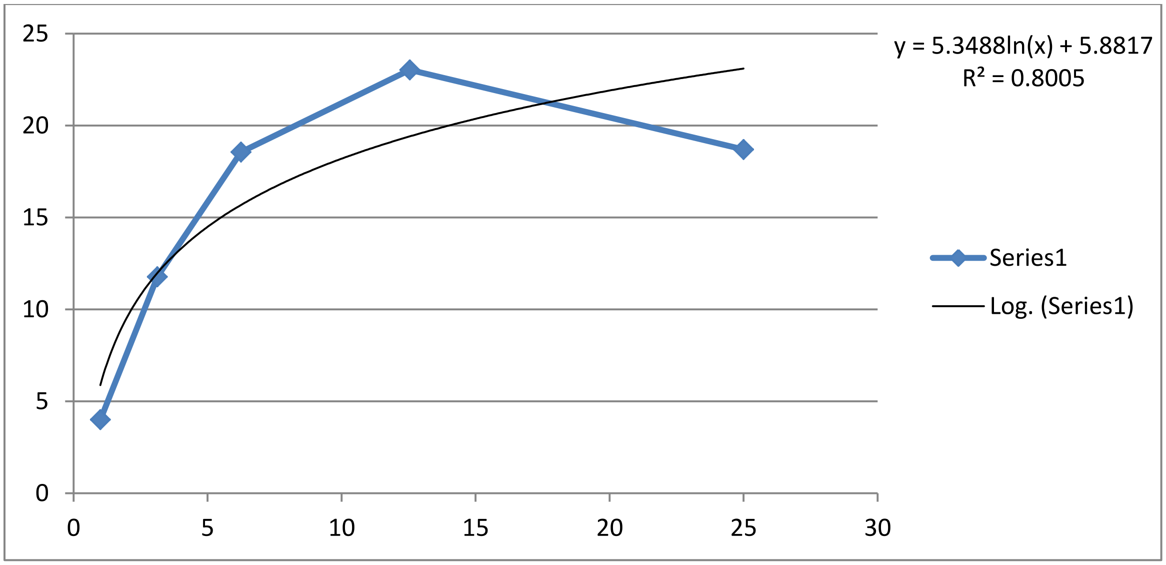

X–axis = concentration (μM) of compound **12**
Y–axis = % Cell Proliferation Inhibition
$IC_{50} > 25$ μM

27. The curves that we used to calculate the IC_{50} value for compound **12** against LNCaP

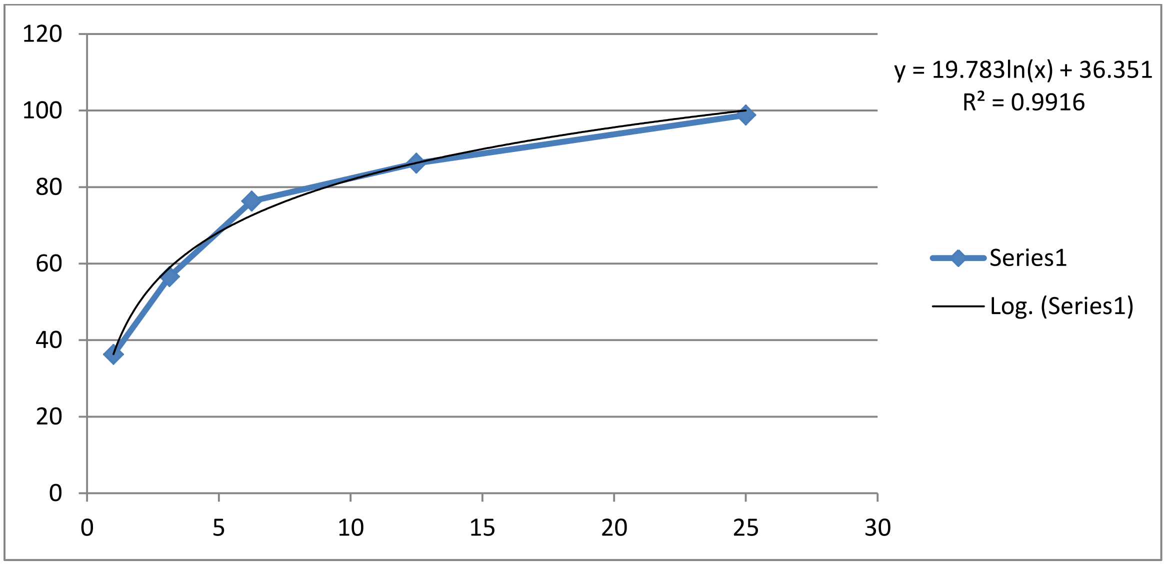

X–axis = concentration (μM) of compound **12**
Y–axis = % Cell Proliferation Inhibition
IC_{50} = 1.994 μM

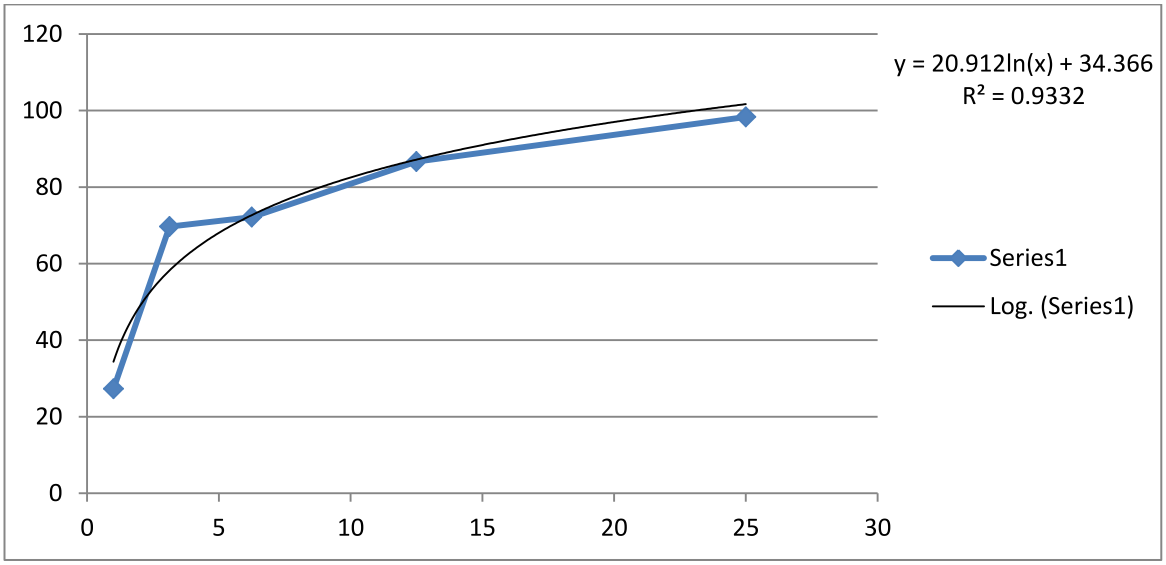

X–axis = concentration (μM) of compound **12**
Y–axis = % Cell Proliferation Inhibition
IC_{50} = 2.112 μM

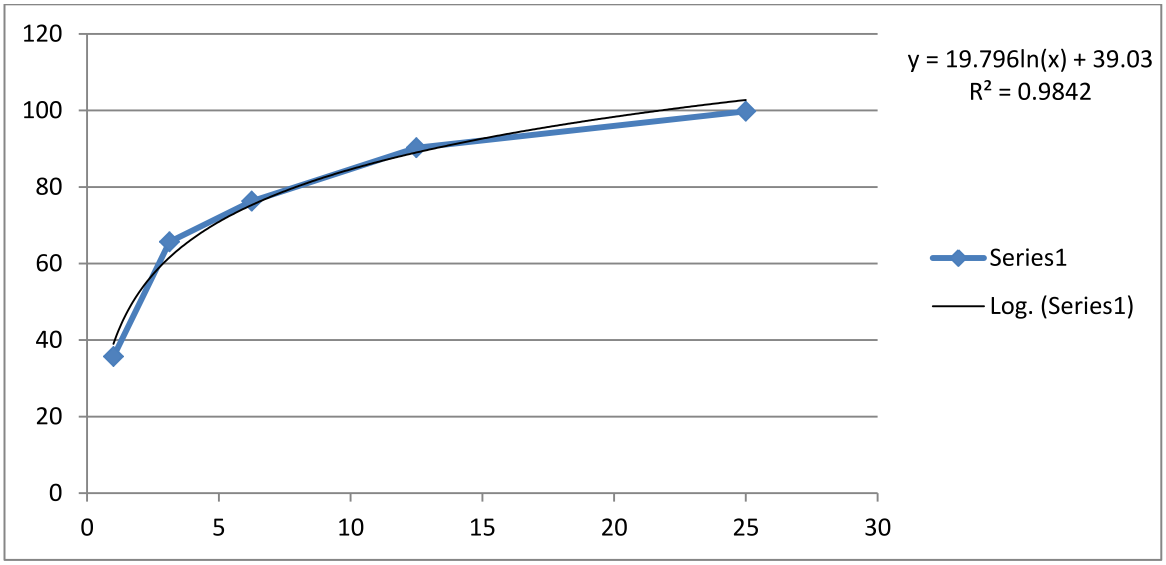

X–axis = concentration (µM) of compound **12**
Y–axis = % Cell Proliferation Inhibition
IC_{50} = 1.740 µM

28. The curves that we used to calculate the IC_{50} value for compound **13** against PC-3

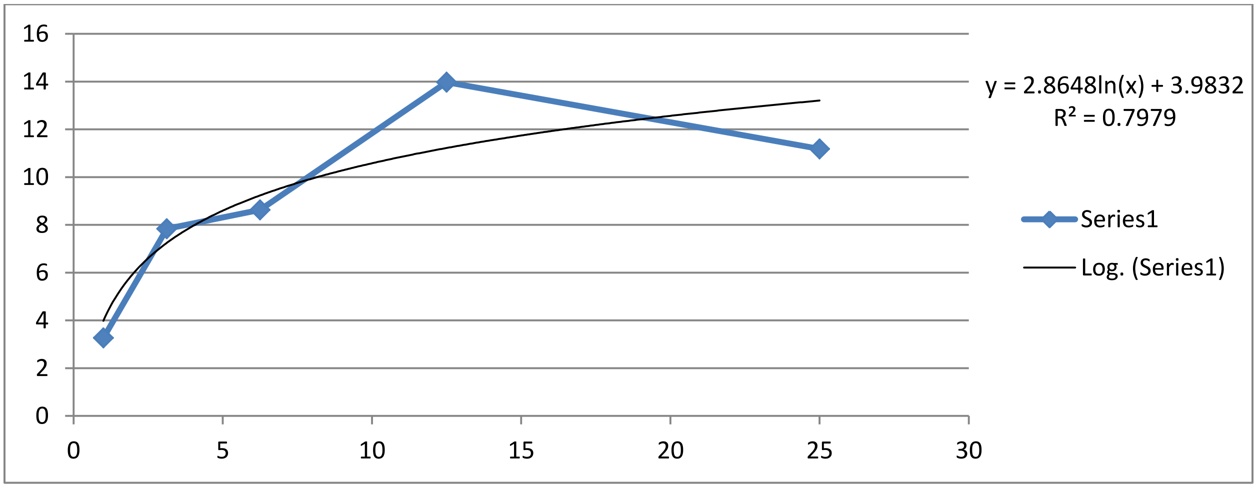

X–axis = concentration (µM) of compound **13**
Y–axis = % Cell Proliferation Inhibition
IC_{50} > 25 µM

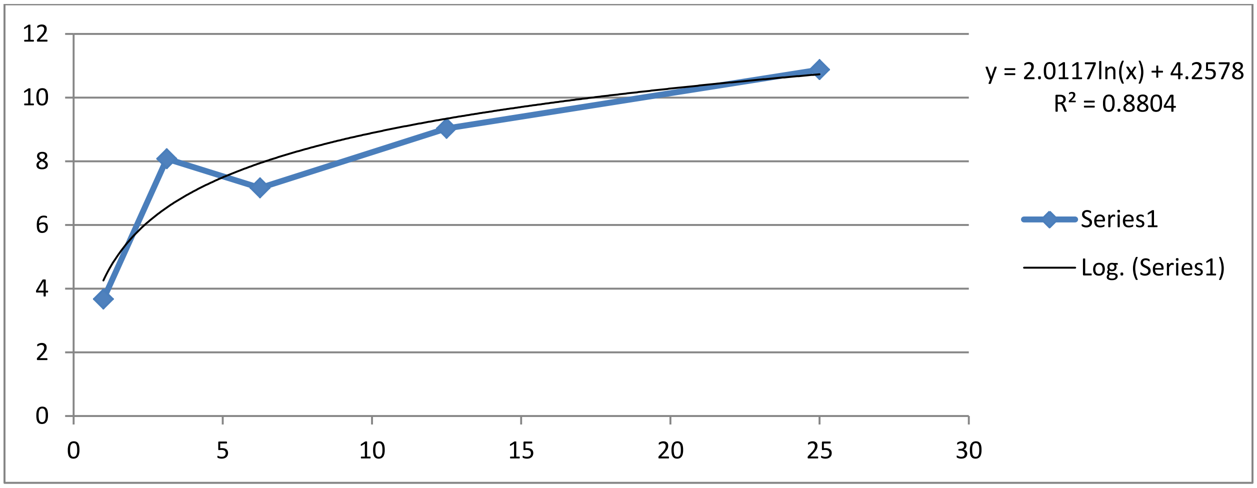

X–axis = concentration (μM) of compound **13**
Y–axis = % Cell Proliferation Inhibition
$IC_{50} > 25$ μM

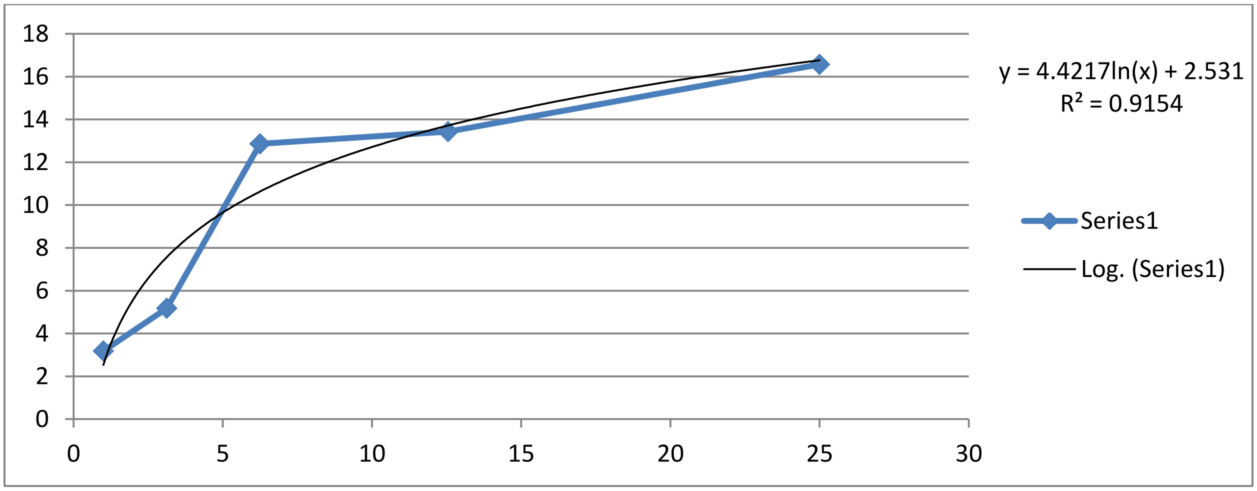

X–axis = concentration (μM) of compound **13**
Y–axis = % Cell Proliferation Inhibition
$IC_{50} > 25$ μM

29. The curves that we used to calculate the IC_{50} value for compound **13** against DU145

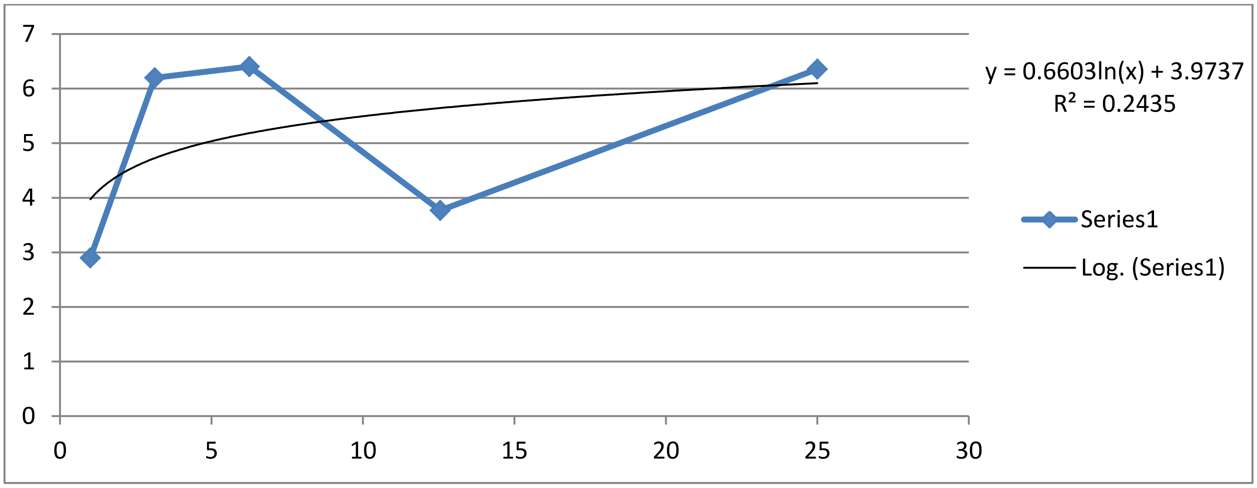

X–axis = concentration (μM) of compound **13**
Y–axis = % Cell Proliferation Inhibition
$IC_{50} > 25$ μM

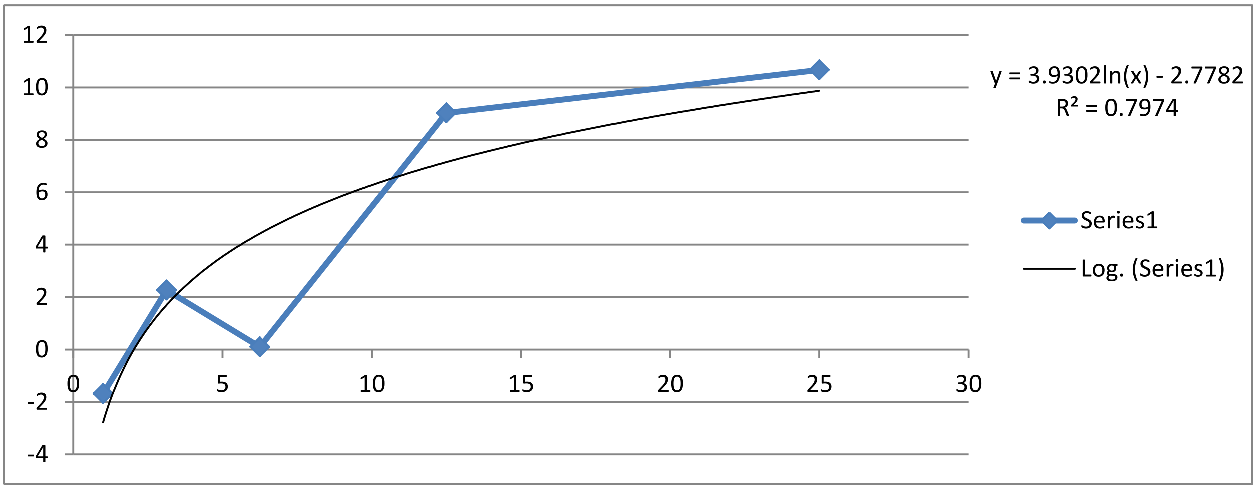

X–axis = concentration (μM) of compound **13**
Y–axis = % Cell Proliferation Inhibition
$IC_{50} > 25$ μM

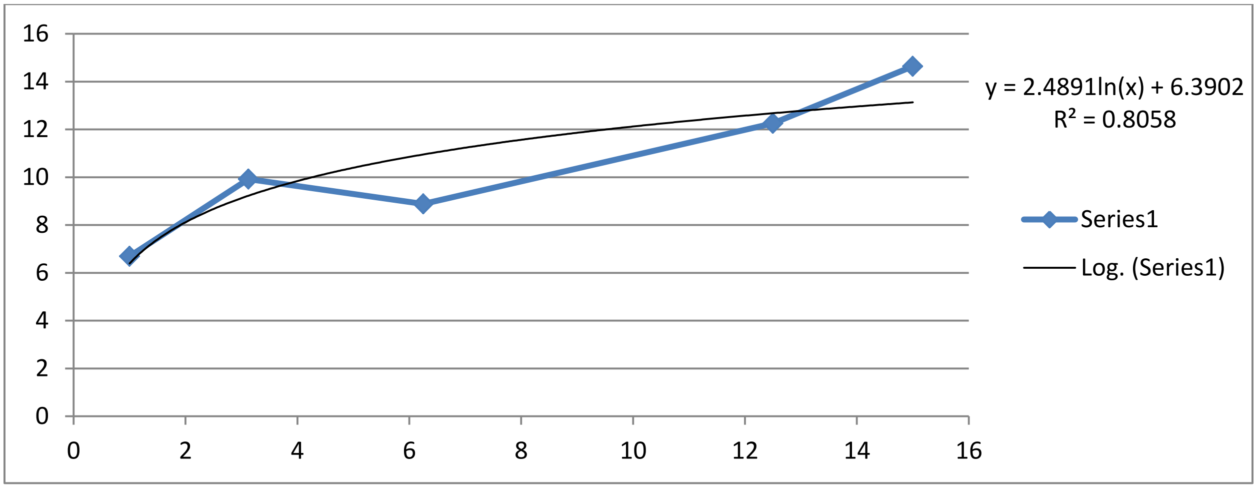

X–axis = concentration (μM) of compound **13**
Y–axis = % Cell Proliferation Inhibition
$IC_{50} > 25$ μM

30. The curves that we used to calculate the IC_{50} value for compound **13** against LNCaP

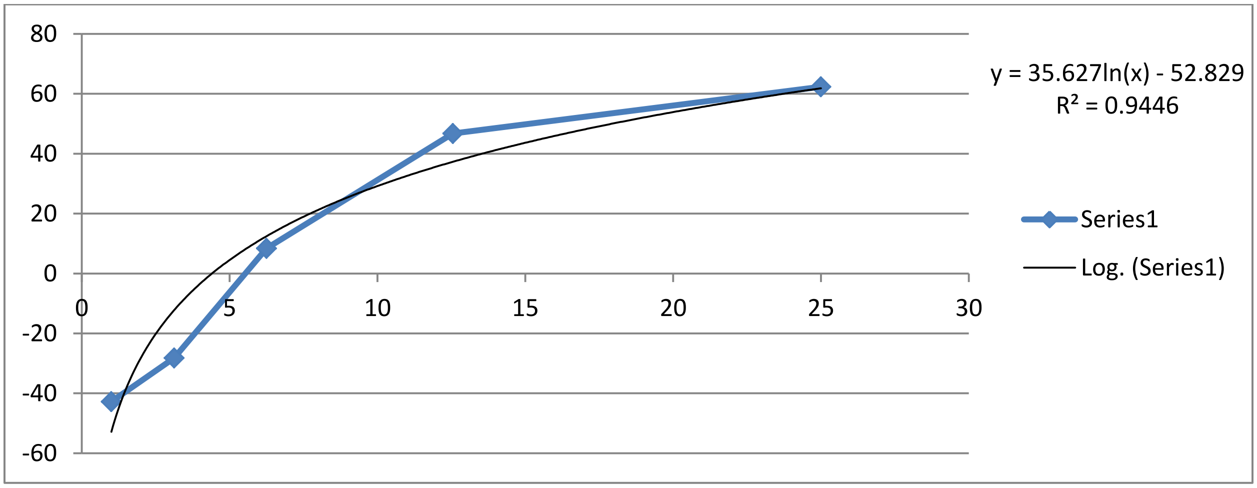

X–axis = concentration (μM) of compound **13**
Y–axis = % Cell Proliferation Inhibition
$IC_{50} = 17.926$ μM

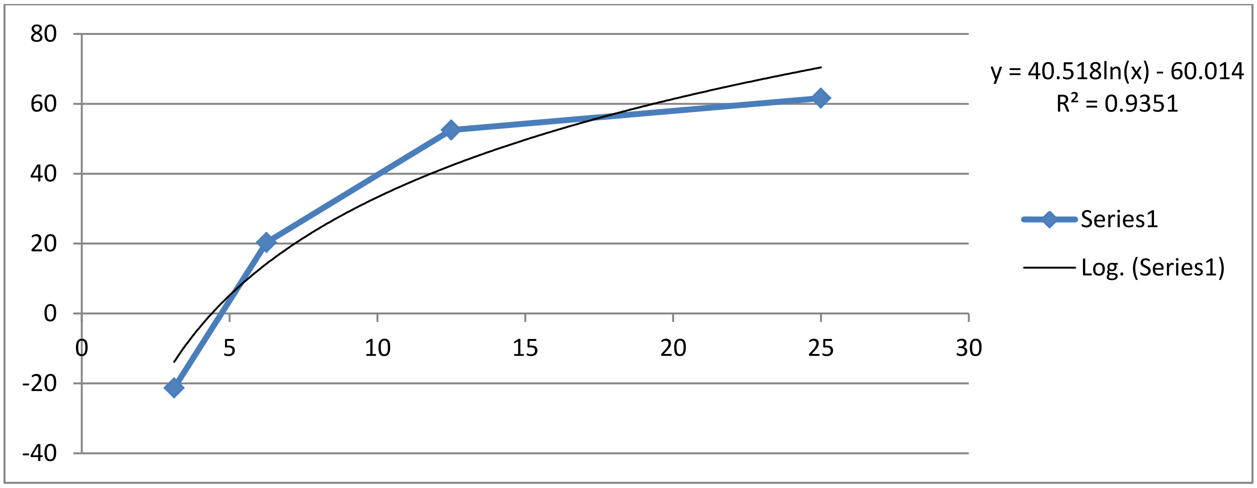

X–axis = concentration (μM) of compound **13**
Y–axis = % Cell Proliferation Inhibition
IC_{50} = 15.123 μM

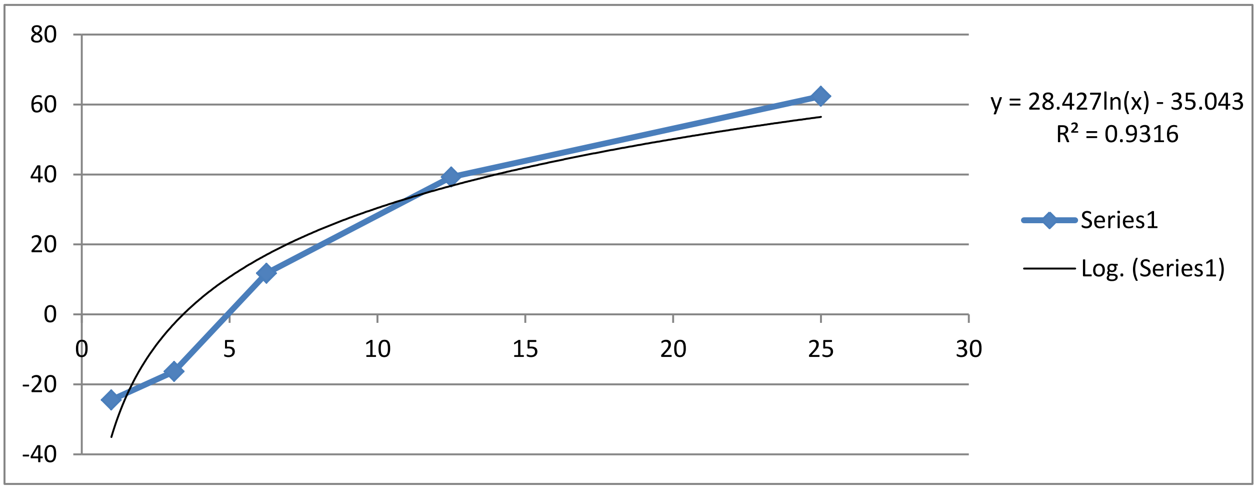

X–axis = concentration (μM) of compound **13**
Y–axis = % Cell Proliferation Inhibition
IC_{50} = 19.918 μM
